# Integrating very high resolution environmental proxies in genotype–environment association studies

**DOI:** 10.1111/eva.13737

**Published:** 2024-06-28

**Authors:** Annie S. Guillaume, Kevin Leempoel, Aude Rogivue, Felix Gugerli, Christian Parisod, Stéphane Joost

**Affiliations:** ^1^ Geospatial Molecular Epidemiology Group (GEOME), Laboratory for Biological Geochemistry (LGB), Ecole Polytechnique Fédérale de Lausanne (EPFL) Lausanne Switzerland; ^2^ Royal Botanic Gardens, Kew Richmond, Surrey UK; ^3^ WSL Swiss Federal Research Institute Birmensdorf Switzerland; ^4^ Department of Biology University of Fribourg Fribourg Switzerland

**Keywords:** digital elevation models, landscape genomics, local adaptation, multiscale analysis, spatial scale, topographic variables

## Abstract

Landscape genomic analyses associating genetic variation with environmental variables are powerful tools for studying molecular signatures of species' local adaptation and for detecting candidate genes under selection. The development of landscape genomics over the past decade has been spurred by improvements in resolutions of genomic and environmental datasets, allegedly increasing the power to identify putative genes underlying local adaptation in non‐model organisms. Although these associations have been successfully applied to numerous species across a diverse array of taxa, the spatial scale of environmental predictor variables has been largely overlooked, potentially limiting conclusions to be reached with these methods. To address this knowledge gap, we systematically evaluated performances of genotype–environment association (GEA) models using predictor variables at multiple spatial resolutions. Specifically, we used multivariate redundancy analyses to associate whole‐genome sequence data from the plant *Arabis alpina* L. collected across four neighboring valleys in the western Swiss Alps, with very high‐resolution topographic variables derived from digital elevation models of grain sizes between 0.5 m and 16 m. These comparisons highlight the sensitivity of landscape genomic models to spatial resolution, where the optimal grain sizes were specific to variable type, terrain characteristics, and study extent. To assist in selecting variables at appropriate spatial resolutions, we demonstrate a practical approach to produce, select, and integrate multiscale variables into GEA models. After generalizing fine‐grained variables to multiple spatial resolutions, a forward selection procedure is applied to retain only the most relevant variables for a particular context. Depending on the spatial resolution, the relevance for topographic variables in GEA studies calls for integrating multiple spatial scales into landscape genomic models. By carefully considering spatial resolutions, candidate genes under selection by a more realistic range of pressures can be detected for downstream analyses, with important applied implications for experimental research and conservation management of natural populations.

## 1 INTRODUCTION

Rapidly changing climatic conditions emphasise an urgent need to understand the capacity of organisms to adapt to novel environments. By studying local adaptation, where populations display increased fitness in their local environment (Kawecki & Ebert, 2004), researchers and conservation practitioners can gain insights into levels of genetic variance both within and among populations to understand their adaptive potential to future conditions (Hoffmann & Sgró, 2011; Whitlock, 2015). Technological and analytical advances over the last decade have unlocked the identification of genomic regions putatively involved in adaptation (Hoban et al., 2016), forming the foundation for experimental testing of adaptive phenotypic responses to heterogeneous environments (Lasky et al., 2023; Savolainen et al., 2013). Although genome scans of genetic differentiation and the detection of outlier loci can identify candidate genomic regions under selection (Lotterhos & Whitlock, 2014), environmental variables must be integrated into analyses to identify ecological factors potentially driving the adaptive process (i.e., selective agents) and predict how organisms might respond to future environmental conditions (Hoban et al., 2016; Lasky et al., 2023; Rellstab et al., 2016).

Genotype–environment association (GEA) methods of landscape genomics are an exploratory bottom‐up approach to study genomic imprints of local adaptation and identify candidate genomic regions under selection. Used to correlate genetic variation segregating in populations with the environmental conditions that they experience (Rellstab et al., 2015), these methods successfully identified putative genes involved in local adaptation across various plant and animal species (e.g., Bogaerts‐Márquez et al., 2021; Selmoni et al., 2021; Todesco et al., 2020). Among the many methods available to perform GEAs (Forester et al., 2018), multivariate redundancy analyses (RDAs) represent promising approaches to detect signatures of selection (Capblancq & Forester, 2021; Forester et al., 2016). RDA models work by maximizing the explained responses of all input loci simultaneously with regards to multiple environmental variables (Legendre & Legendre, 1998). Because of this, they have recently gained traction in landscape genomic analyses as they provide more ecologically relevant models of adaptation than traditional univariate GEA methods (Lasky et al., 2023), including the popular latent factor mixed models (LFMM; Frichot et al., 2013) or the SamBada approach (Duruz et al., 2019; Stucki et al., 2017). Additionally, simulations highlight their robustness to demography and sampling designs (Forester et al., 2018) while maintaining higher power and lower false discovery rates than traditional univariate methods (Capblancq et al., 2018). Though improvements in resolutions of genomic and environmental datasets have spurred the development of these GEA methods (Dauphin et al., 2023), the impact of using high‐resolution environmental variables on the detection of putative genes under selection is still largely speculative and unknown. To avoid ambiguity and facilitate discussion (Anderson et al., 2010), here we define *spatial resolution* as the grain size of environmental variables, *extent* as the boundary size of the study site, and *level* as the difference between individual study sites (local level) and their agglomeration (regional level).

The issue of spatial scale in ecology is not new (Levin, 1992), yet a current limitation of evolutionary ecology methods, including GEAs, is that the full implications of spatial scale on the reliability, accuracy and interpretation of model results remains unknown (Dungan et al., 2002). Patterns observed in nature are the result of processes occurring across a continuum of nested spatial scales, where modelling requires a biologically arbitrary selection of variables at distinct spatial resolutions (Fitzpatrick & Keller, 2015). Despite awareness of this sensitivity, employing the finest spatial resolution available, often with little to no justification, remains the main paradigm (Moudrý et al., 2023). While “finer‐is‐better” may be acceptable for variables ≥90 m (Chauvier et al., 2022; Cushman & Landguth, 2010), the accessibility of very high‐resolution terrain variables at resolutions ≤1 m (e.g., Kasser et al., 2019) calls for spatial resolutions to be more thoroughly assessed prior to analyses. For instance, species distribution models of an alpine plant were optimized when using resolutions between 2 and 16 m (Guillaume et al., 2021), despite expectations that the finest resolutions ≤0.5 m would seem most relevant across such rugged terrain.

Topographic variables derived from digital elevation models (DEMs) have gained popularity in evolutionary ecology modelling to complement climatic variables when describing spatial patterns of plant diversity (Irl et al., 2015; Scherrer & Körner, 2011) and have been successfully implemented in species distribution modelling (e.g., Gottfried et al., 1998; Guillaume et al., 2021) and landscape genomic analyses (e.g., Leempoel et al., 2018). A plethora of topographic variables can be derived, which are classified into: (i) primary terrain attributes calculated directly from DEMs (including slope, curvature, and aspect as northness and eastness); and (ii) secondary terrain attributes derived from the primary attributes to describe a given pattern as a function of a process (e.g., vector ruggedness measure, soil wetness indices, and solar radiation; Wilson & Gallant, 2000). These secondary terrain attribute variables have been specifically developed to represent measured environmental characteristics, including the air humidity, soil moisture, air temperature (e.g., Leempoel et al., 2015), as well as biophysical processes including erosion, water flow, and solar radiation (e.g., Moore et al., 1991). Care must be taken when selecting the spatial resolution of these variables (Anderson et al., 2010), as the same variable type that is generalized to different spatial resolutions can describe different terrain processes (Keitt & Urban, 2005). Fine resolutions can add high amounts of unnecessary detail and noise (Kalbermatten et al., 2012), whereas coarse resolutions may generalise over important topographic structures and thus miss relevant ecological patterns (Pain, 2005). The spatial resolution of a variable therefore may change its relevance in different ecological contexts (Chauvier et al., 2022; Leempoel et al., 2015).

The optimal spatial resolution for ecological modelling depends on species’ biology (e.g., Anderson et al., 2010; Loke & Chisholm, 2022), study region extent (e.g, Anderson et al., 2010), terrain topography (e.g., Guillaume et al., 2021), and variable type (e.g., Chauvier et al., 2022; Leempoel et al., 2015), making it challenging to generalise the selection of the most appropriate spatial resolution for use in evolutionary ecology models (Woodcock & Strahler, 1987). One option is to systematically compare the performance of models built with predictor variables at various spatial resolutions, where different resolution variables can be produced using a multiscale approach (Woodcock & Strahler, 1987). For DEM‐derived variables, multiscale can be achieved using a Laplace‐gradient wavelet transformation to first generalise a fine‐resolution DEM to coarser resolutions, extracting the most prominent nested topographic patterns at each step (Kalbermatten et al., 2012) before deriving relevant terrain variables (Leempoel et al., 2015). A draw‐back to this systematic method is that it can be time consuming and impractical to investigate each combination of variable type and spatial resolution, particularly when investigating multiple study sites with different terrain characteristics. An alternative is to obtain the full range of desired variables at multiple resolutions before implementing a selection procedure to retain only the variables at resolutions that are most relevant to the data. In species distribution modelling this has been achieved by retaining the variables at resolutions that best discriminate presence data from random background points (e.g., Guillaume et al., 2021; Rochat et al., 2021) or using an automatic collinear variable selection algorithm (Adde et al., 2023); in landscape genomics by using a stepwise forward selection procedure (Blanchet et al., 2008) to select the variables that maximise genomic variance explained (e.g., Capblancq & Forester, 2021). However, the relevance of such approaches in GEA models are yet to be investigated.

Here, we implement an approach to obtain environmental proxies at multiple resolutions for conducting GEA analyses, looking to assess environmental variable effects on genomes at different spatial resolutions. Specifically, multivariate RDA models are used to combine very‐high resolution genomic and environmental datasets, with the aims of (i) systematically assessing the sensitivity of GEA models to topographic environmental variables at multiple spatial resolutions, and (ii) investigating a method to select multiscale variables for use in GEA models. This work falls in the context of a landscape genomics study on the perennial herb *Arabis alpina* L., sampled across four alpine valleys in the western Swiss Alps. As species distribution modelling predictions in this system were optimized when using variables ranging from 2 to 16 m (Guillaume et al., 2021), depending on site characteristics and variable type, it is hypothesized that DEM‐derived variables of 2–16 m spatial resolutions will be most relevant for detecting signatures of selection in this system. We highlight the importance of carefully considering and justifying the spatial resolution of predictor variables used in evolutionary ecology models and demonstrate a practical method to produce, select, and integrate multiscale variables into GEA models. By carefully considering spatial resolutions, candidate genes under selection can be more accurately detected for downstream analyses, with important applied implications for experimental research and conservation management of natural populations.

## 2 METHODS

### 2.1 Study species and sample sites

*Arabis alpina* (Brassicaceae) is an arctic‐alpine perennial plant that is becoming a widely used model organism to study ecological genomics (reviewed in Wötzel et al., 2022). In the Alps, individuals occur mostly in high‐alpine areas with rugged terrain, typically characterized by calcareous scree slopes and unstable rocky structures (Buehler et al., 2012). *Arabis alpina* has a relatively small genome of approximately 375 Mbp (Jiao et al., 2017; Willing et al., 2015). The present study uses publicly available data from Rogivue et al. (2019a) containing information on 304 geo‐referenced *A. alpina* individuals located across four valleys in the western Swiss Pre‐Alps: Essets (ESS; *N* = 70), Martinets (MAR; *N* = 96), Para (PAR; *N* = 69) and Pierredar (PIE; *N* = 69) (Table 1; Figure S1). For each site, individuals were sampled in 10 plots of 6–10 individuals, with at least 1 m between individuals and a maximum of 10‐30 m between individuals of a plot (for schema, see figure 1 in Rogivue et al., 2023). Individuals were geo‐referenced to an accuracy of ±2 cm with a Differential Global Positioning System (DGPS) receiver.

**TABLE 1:** Location and genomic information (number of SNPs used in analyses) for the four study sites (ESS, MAR, PAR, and PIE) separately (local; A–D) and grouped (regional; E).

	(A) ESS	(B) MAR	(C) PAR	(D) PIE	(E) Regional
Latitude	46°16′2″N	46°12′37″N	46°23′23″N	46°19′13″N	
Longitude	7°9′52″E	7°5′12″E	7°9′6″E	7°11′35″E	
Overall orientation	N	NE	NNE	NW	N
Target study site area (km^2^)	0.7	0.56	0.51	0.43	2.2
Sampled plant individuals	70	96	69	69	304
*Genomic data*					
Total SNPs	220,214	113,900	287,261	160,322	291,396
Intergenic SNPs	183,647	94,904	240,404	133,888	243,622
Intergenic SNPS: LD pruned	6957	5991	7555	5705	11,813
Intragenic SNPs	36,567	18,996	46,857	26,434	47,774
Intragenic SNPs: high‐impact	13,945	7813	17,537	10,757	22,806
Genes with high‐impact SNPs	6331	3831	7832	5196	9632

Although these four populations have a shared post‐glacial ancestry (Rogivue et al., 2018), investigations of genome‐wide single‐nucleotide polymorphism (SNP) data revealed restricted historical gene flow between populations, with pairwise *F*_ST_ values between 0.09 and 0.18 (Rogivue et al., 2019a). Additionally, low observed heterozygosity (*H*_O_ between 0.05 and 0.17) corresponded to significantly high inbreeding coefficients (*F*_IS_ between 0.18 and 0.28) coupled with high levels of selfing in this region (Zeitler et al., 2023). Given predominant selfing requiring insect pollinators and limited dispersal capacities of small wind‐dispersed seeds between mountain valleys, gene flow is expected to occur over short distances in this species (<1 km; Ansell et al., 2008; Buehler et al., 2012). The distribution and orientation of the valleys (>7 km between each north‐facing valley) support the four sites as independent replicates, with a common genomic background and comparable genetic variation, for studying local adaptation.

We investigated the impacts of spatial scale on the detection of local adaptation at the local level by assessing each of the four valleys independently (local: ESS, MAR, PAR, and PIE), as well as at the regional level by grouping the four valleys together (regional). A schema providing a workflow of the methods is given in Figure S1.

### 2.2 Environmental data

The four valleys were characterized using topographic variables derived from DEMs at multiple spatial resolutions, following the methods of Guillaume et al. (2021). Raw LiDAR (light detection and ranging) point clouds produced by the Direction of Land Registry and of Geoinformation (DCG), Canton of Vaud, Switzerland, were obtained from the ASIT Vaud website (www.asitvd.ch; accessed 12.9.2019) and selected to cover the target study site extents and surrounding mountain topography. Point clouds were processed using CloudCompare GLP software (version 2.10.2, 2020; retrieved from http://www.cloudcompare.org/) to produce DEMs with a 0.5 m grain size geo‐referenced in the Swiss reference system (MN95: CH1903+/LV95).

To obtain multiscale variables, the base DEMs at 0.5 m were generalized to 1, 2, 4, 8, and 16 m resolutions using a Gaussian pyramid algorithm in MATLAB (version R2019a, 2019; retrieved from https://www.mathworks.com) with the *impyramid* function, following the work of Kalbermatten et al. (2012). These spatial resolutions were chosen following a multiscale species distribution model study using *A. alpina* occurrence data at MAR and PAR, where topographic variables were most important at resolutions of 2–16 m (Guillaume et al., 2021).

The same nine variable types relating to terrain morphometry, hydrology, solar radiation, and climate were derived from each of the DEMs at the six spatial resolutions, using SAGA GIS (v7.5.0; Conrad et al., 2015). The nine DEM‐derived variable types used here include the primary terrain attributes of slope (SLO), horizontal curvature (HCU), and aspect in the form of eastness (EAST – sine of aspect) and northness (NORTH—cosine of aspect), as well as the secondary terrain attributes of vector ruggedness measure (VRM), the SAGA wetness index (SWI), the sky view factor (SVF), total irradiance in June (TI6), and the wind exposure index (WEX). Study sites were characterized across the site extents with mean and standard deviation of the nine variable types at the middle resolution of 2 m. Mean terrain ruggedness (assessed as VRM) was used to classify the sites into homogeneous and heterogeneous terrain sites (Loke & Chisholm, 2022). Detailed descriptions and parameters used to produce the variable types can be found in Table S1.

The geographic coordinates of the 304 sampled plants were used to extract corresponding environmental values from each of the 54 DEM‐derived variables (nine variable types at six spatial resolutions), as well as elevation at 0.5 m, using the *extract* function of the raster R package (v.3.5.15; Hijmans, 2022). Upon generalization of variables toward coarser resolutions, an average of four samples occurred in the same grid at the 16 m resolution (up to maximum of 10 individuals). While this pseudo‐replication could be handled by iteratively running GEA analyses with randomly removed individuals, we retained all samples to maintain both GEA model power (Selmoni et al., 2020) and consistent sample sizes for comparisons between models (Guillaume et al., 2021; Guisan et al., 2007).

Environmental variable values were standardized (i.e., centered and scaled) for each local and regional analysis to remove biases arising from differences in variable units (Legendre & Legendre, 1998). Due to the large number of variables produced upon multiscale generalization, independence amongst the variable types was assessed at the finest resolution of 0.5 m using a Spearman rank correlation with a threshold of |*r*_*s*_| ≥ 0.8 (Dormann et al., 2013) for each local site to minimise the number of variable types for multiscale generalization. Principal component analysis (PCA) based on the 0.5 m DEM‐derived variables was performed for each local site to verify that plots were sampled across a range of conditions using the *prcomp* function in the stats R package (v.4.1.2; R Core Team, 2021).

To investigate how to best select variables at appropriate spatial resolutions for use in GEA models, we systematically tested eight sets of DEM‐derived variables for each site and level (Table 2). The first six Variable Sets (shortened to “VS”) were based on the nine variable types at one of the following spatial resolutions: 0.5, 1, 2, 4, 8, or 16 m, along with elevation at 0.5 m, such that each of these single resolution variable sets (“VS*‐single*”) had 10 predictor variables. These will be referred to as “VS*‐0.5m*”, “VS*‐1m*”, etc. The seventh variable set (“VS*‐all*”) included all nine variable types at all six spatial resolutions, plus elevation at 0.5 m, giving a total of 55 predictor variables. As this produced many correlated predictor variables, the eighth variable set (“VS*‐fwd*”) was created from the VS*‐all* model. To produce VS*‐fwd*, a stepwise forward model selection procedure was implemented to maximise the genetic variance of the intragenic SNP dataset (described in Section 2.3 below) explained by the DEM‐derived variables (Blanchet et al., 2008). After ensuring significance of the VS*‐all* global RDA model, the forward selection procedure began with an empty null model. The null model was complexified by adding one explanatory variable at a time, where the model stopped either when: (i) the permutation‐based significance test *p* < 0.01 threshold was reached (across 1000 permutations), or (ii) the model's adjusted*‐R* ^2^ began to decline (following Capblancq & Forester, 2021). The resulting VS*‐fwd* variable sets were site‐specific, with 7–14 predictor variables at the local sites, and 31 predictor variables at the regional level. Forward selection was performed with the *ordiR2step* function of the vegan R package (v.2.5.7, Oksanen et al., 2020).

**TABLE 2:** Description of the eight Variable Sets (shortened to “VS”) produced to investigate the effect of spatial resolution in GEA models. Nine topographic environmental variables (Table S2) were produced at six spatial resolutions (0.5, 1, 2, 4, 8, and 16 m).

	Variable set	Description	Variables per set
i–vi	VS*‐single* (e.g. VS*‐0.5m*)	Each variable at one single spatial resolution, plus elevation at 0.5 m	10
vii	VS*‐all*	All variables at all spatial resolutions, plus elevation at 0.5 m	55
viii	VS*‐fwd*	Site‐specific stepwise forward selected variables based on the VS*‐all* model	7–14 (local); 31 (regional)

### 2.3 Genomic data

The publicly available “non‐TE SNP dataset” containing whole‐genome sequenced SNP variants outside of identified transposable element (TE) sequences was obtained from Rogivue et al. (2019b), with complete sampling and data processing details described in Rogivue et al. (2019a). Briefly, SNP filtering was done at the local and regional levels independently for a minor allele frequency (MAF) of <0.025 and a 10% missingness threshold. Missing genotypes were independently imputed at each local and regional level using the *snmf* and *impute* functions in the LEA R package (v.3.6.0, Frichot & François, 2015), where the *K* latent factors retained for imputation (ESS: *K* = 2; MAR: *K* = 3; PAR: *K* = 6; PIE: *K* = 2; Regional: *K* = 6) were based on the lowest genomic inflation factor values.

The annotation based on the reference genome v5.1 (Jiao et al., 2017) was used to further divide the imputed SNP dataset into two categories, following Capblancq and Forester (2021): (i) intergenic SNPs lying outside of coding regions that are treated as putatively neutral, and (ii) intragenic SNPs within coding regions that are more likely directly influenced by natural selection. They were used in analyses assessing neutral and adaptive processes, respectively, where the intergenic SNPs were first pruned for linkage disequilibrium (LD; threshold = 0.2) using the *snpgdsLDpruning* function of the SNPrelate R package (v.1.28.0; Zheng et al., 2012). It is noted that intergenic SNPs may be involved in selective processes due to LD, with whole‐genome LD decay estimated at *r* ^2^ < 0.1 within 30.98 kb for non‐TE SNPs (Rogivue et al., 2019a). However, as the present study does not look to provide exhaustive evaluations of SNPs under selection, only the intragenic SNP set was used as the response variable in downstream analyses. This was done to simplify model comparisons, where preliminary analyses found that the VS*‐fwd* models explained the most variation for the intragenic SNP dataset compared with intergenic or whole genome (Table S2). Intragenic SNP variants were further annotated using SnpEff (Cingolani et al., 2012) to identify high‐impact SNPs as those with a direct impact on gene functionality (i.e., within‐gene variants involved in non‐synonymous mutations including missense variants, splice acceptor and intron variants, starts lost, stops gained, and splice region variants). All analyses were performed at the individual genotype level, with genomic data coded as the count of the alternative allele for each locus.

### 2.4 Genotype–environment associations

To identify candidate loci involved in local adaptation at the local and regional levels, GEAs using multivariate RDAs were performed following Capblancq and Forester (2021). A partial RDA was used to understand the partitioning of intragenic SNP variation into neutral (assessed as population structure and spatial geographic structure) and adaptive (assessed with environmental variables) processes at each local and regional level (full methods in Appendix S2). A full RDA was then performed for the GEA analyses, with intragenic SNPs coded to individual genotypes in the response matrix and the DEM‐derived variable sets (Table 2) in the explanatory variable matrix. Strong population structure was corrected in the regional analysis using the first three principal components (PC) of the LD‐pruned intergenic SNP PCA (Appendix S2) as conditioning variables in a partial RDA. The weak population structure detected at MAR was highly correlated with elevation (*r*_*S*_ = −0.88), such that it was not corrected for. This decision to not correct for population structure follows simulations indicating reduced RDA model power and inflated FDR due to altered mapping of quantitative trait mutations into the ordination space when an environmental gradient is correlated with structure (Forester et al., 2018; Lotterhos, 2023). For each local and regional level, the effect of variable sets on the detection of candidate SNPs under selection was investigated, resulting in 40 GEA models in total.

Outlier SNPs were identified based on RDA loadings, following methods outlined in Capblancq et al. (2018). Scree tests were used to select only the first *K‐*constrained RDA axes that most frequently explained a majority of the genetic variance associated with the predictor variables. The same number of *K* axes were retained for each site to ensure compatibility amongst variable sets, given a minimum of two *K* axes. The custom *rdadapt* function from Capblancq et al. (2018) was used to evaluate the significance of each SNP based on its extremeness of its Mahalanobis distance value compared to the distribution of the other SNPs in the RDA space of *K* retained axes. The Mahalanobis distances were computed using the *covRob* function of the ROBUST R package (v.0.7.0; Wang et al., 2022), corrected for inflation factor (François et al., 2016) and distributed along a chi‐squared distribution with *K* degrees of freedom to assign a *p*‐value to each SNP (Luu et al., 2017). A stringent Bonferroni correction was applied to identify outlier loci, using the threshold of *p*‐value <0.01/the number of tests (i.e. the number of SNPs in each RDA model). The lists of SNPs detected as outliers by each RDA model were compared between variable sets using upset plots made with the UpSetR R package (v.1.4.0; Gehlenborg, 2019). Provided the putative functional relevance, we focus on the high‐impact SNPs detected as outliers.

RDA biplots for the *K* retained axes were used to associate outlier SNPs with DEM‐derived variables. For each outlier SNP, its projection onto each variable vector in the *K* retained axes was used to assign the associated predictor variable as the one with the largest absolute scalar value. The proportion of loci detected as outliers from the investigated high‐impact SNP set (p_S_; based on Ahrens et al., 2018) were calculated to allow for comparisons between sites and to identify whether a particular model resulted in a higher relative frequency of outlier SNPs compared to other models. The number of associations between high‐impact outlier SNPs and predictor variables that occurred within each gene were tallied. Genes with multiple SNP–variable associations were noted as “multiple”.

### 2.5 Gene ontology enrichment analyses

Gene ontology (GO) terms associated with genes assessed the putative function(s) of candidate high‐impact SNPs detected by the DEM‐derived variables in each model, following Primmer et al. (2013). Using the high‐quality *A. alpina* reference genome annotation, GO enrichment analyses were performed with the topGO R package (v.2.46.0; Alexa & Rahnenführer, 2021), assessing overrepresented GO terms among genes associated with each DEM‐derived variable. The significance of enriched GO terms was determined using Fisher's exact tests using the default *weight01* algorithm and ranking by *p*‐value to only retain significant GO terms (*p* < 0.05) associated with at least five genes.

## 3 RESULTS

### 3.1 Topographic variables and terrain characteristics

DEM‐derived topographic variables were successfully used to characterise four alpine study sites. Independence amongst the nine DEM‐derived variable types (Table S1) was confirmed for the finest grain size of 0.5 m to avoid redundancy in downstream analyses, where only TI6–SLO at MAR (*r*_*s*_ = −0.92) and EAST–NORTH at PIE (*r*_*s*_ = 0.85) had Spearman correlations above the |*r*_*s*_| ≥ 0.8 threshold (Figure S2). Positive correlations observed amongst variables of the same type generalized to six spatial resolutions (0.5, 1, 2, 4, 8, 16 m) were strongest when grain size was similar, and weakened with increased differences amongst grain size (Figure S3). Elevation at 0.5 m, included to represent temperature and biotic factors, was uncorrelated with the DEM‐derived variables across all sites, except for VRM 4 m (*r*_*s*_ = −0.8) at ESS. In contrast, elevation was strongly negatively correlated with latitude (Y‐coordinate), as expected for predominantly north‐orientated alpine valleys.

PCAs (Figure S4) confirmed that individuals sampled at the four sites were exposed to similar ranges of environmental conditions, allowing them to be treated as replicate systems potentially undergoing independent local adaptation within sites. The characteristics of the sites based on the 2 m derived variables assisted the interpretations of GEA results (Table S3). The less complex, more homogeneous terrain sites of ESS and PIE were characterized by more gentle slopes with low rugosity, wetter soils and higher irradiance, while the more complex, heterogeneous terrain sites of MAR and PAR were characterized by steeper and more rugged slopes, with drier soils, moderate irradiance and more wind exposure.

### 3.2 Variable selection: Stepwise forward model

The selection order of DEM‐derived variables for forward selection (VS*‐fwd*; Table 2) hints at their relative contributions in driving genomic variation, where in some cases the same variable type was selected at multiple spatial resolutions (Table 3). Here, terrain complexity and variable type had the greatest influence on variable ranking. Variables at coarser resolutions (4, 8, 16 m) were predominately selected at homogeneous terrain sites (ESS and PIE), while variables at finer resolutions down to 1 m grain size were also selected at heterogeneous terrain sites (MAR and PAR) (Table 3; Table S4). Notably, only elevation was selected at the finest resolution of 0.5 m for local sites, where it was always one of the first variables selected. Primary terrain variables (i.e., SLO, HCU, EAST, NORTH) were selected at coarse spatial resolutions (8 m, 16 m) at the homogeneous sites and at mid to coarse resolutions (2–16 m) at the heterogeneous sites. Secondary terrain variables were overall selected at finer resolutions: variables representing solar radiation and sky view (TI6, SVF) were selected toward the mid‐resolutions between 4 and 16 m, and variables representing rugosity (VRM), soil wetness (SWI) and wind exposure (WEX) were selected at finer resolutions between 1 and 16 m. All spatial resolutions were represented among the 31 selected variables at the regional level, with similar trends as with the local sites: primary variables were selected between 2 and 16 m; TI6, SVF were selected between 2 and 16 m; and VRM, SWI and WEX were selected between 0.5 and 16 m.

**TABLE 3:** Forward selected DEM‐derived variables that explained the most variance in the intragenic SNP dataset of individuals sampled at the four sites (ESS, MAR, PAR, PIE) separately (local; A–D) and grouped (regional; E). Numbers indicate the order that variables were selected, where blank cells indicate that the variable was not selected. Detailed results are provided in Table S4.

Res.	(A) ESS						(B) MAR						(C) PAR						(D) PIE						(E) Regional					
	0.5	1	2	4	8	16	0.5	1	2	4	8	16	0.5	1	2	4	8	16	0.5	1	2	4	8	16	0.5	1	2	4	8	16
Elev	1						1						2						1						1					
SLO					5							10			7		4							4			26	28		15
EAST					13	9					11	9																	10	3
NORTH					7				5						1									2					27	13
HCU						14						12												8						
VRM			4	3								4												5		31	29	11	21	20
SWI				12	11				6															3	19			5		14
SVF						2										5		6						9			22	17	23	6
TI6					8					13		3												7			4	30	24	12
WEX						10		8	7			2		3								10	6		8	7	16	2	9	18

### 3.3 RDA model evaluation

Multivariate RDAs identified candidate loci strongly associated with environmental variables at the local and regional levels, despite neutral processes of population and geographic structures explaining approximately a third of explained genomic variation at local sites (see Appendix S2 for full results; Figures S5–S8, Tables S4 and S5). The *K* constrained RDA axes retained were selected using Scree plots (ESS: *K* = 3, MAR: *K* = 2, PAR: *K* = 2, PIE: *K* = 3, and regional: *K* = 2; Figure S9; Table S7). Outlier loci were identified as those with *p*‐values below the stringent Bonferroni thresholds (ESS: 2.73 × 10^−7^, MAR: 5.26 × 10^−7^, PAR: 2.13 × 10^−7^, PIE: 3.78 × 10^−7^, and regional: 2.09 × 10^−7^), after adjusting locus‐wise *p*‐value distributions using genomic inflation factors (Table S7; Figure S10). Downstream analyses focused on loci annotated as high‐impact variants (i.e., SNPs that have a direct impact on gene functionality via non‐synonymous mutations that change amino acids and are thus more likely influenced by selection), which accounted for 40%–65% of outlier SNPs at the local levels and 50% at the regional level (Table S7).

**FIGURE 1:**
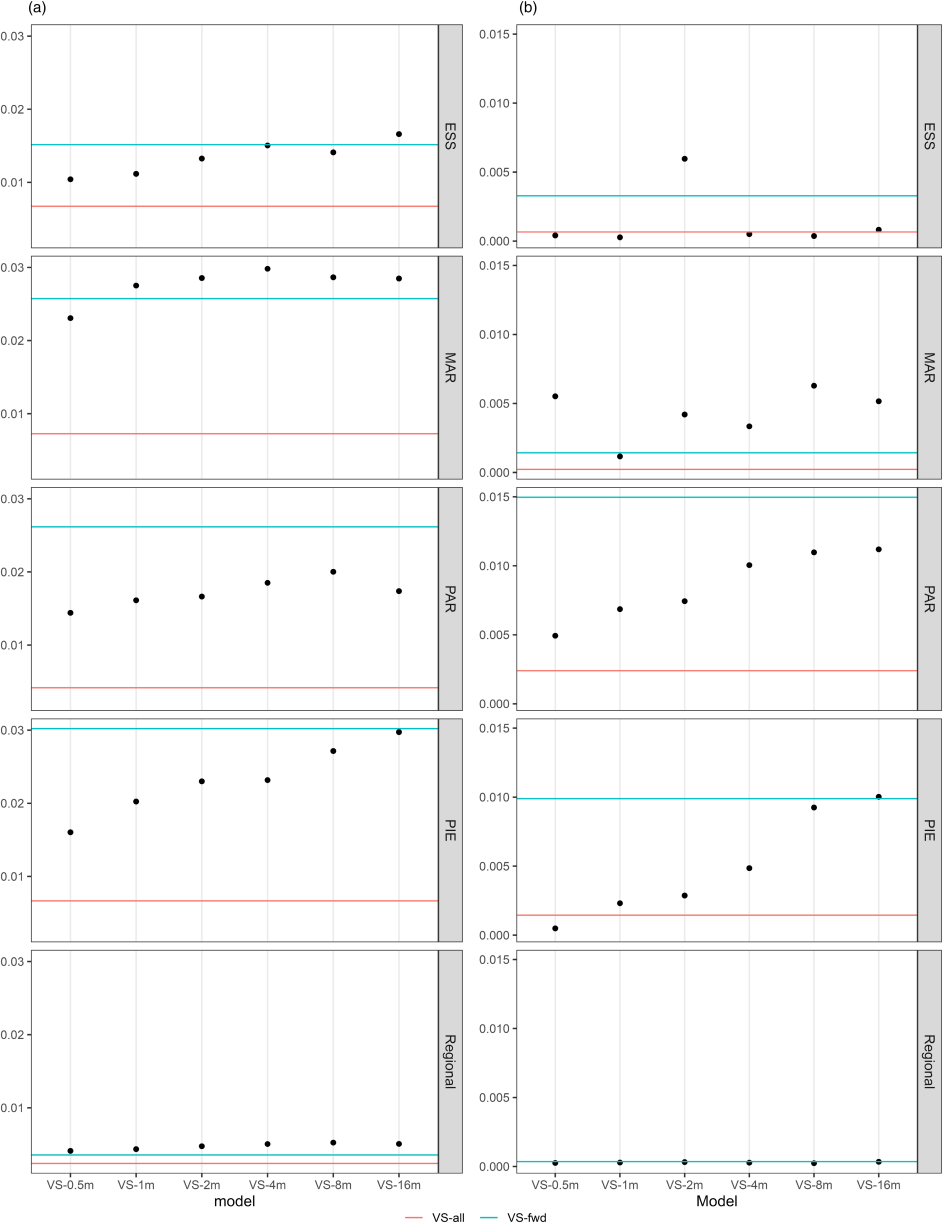
Assessment of RDA model performance based on two metrics: (a) RDA model's adjusted‐*R* ^2^ values, and (b) the proportion of high‐impact SNPs that were detected as outliers by RDA models (ps), where values on the *y*‐axis for (a) have been corrected for the number of input variables (see Table 2). Raw values can be found in Figure S11. For each local (ESS, MAR, PAR, PIE) and regional analysis, model performance metrics along the *x*‐axis are VS*‐single* models built with all variables at the same spatial resolution (0.5, 1, 2, 4, 8, and 16 m), while the red horizontal line indicates the VS*‐all* model, and the blue horizontal line indicates the VS*‐fwd* model. All models include elevation at 0.5 m resolution. Note that gray dashed lines between points are indicative only and each model is independent.

GEA models were sensitive to predictor variable spatial resolutions, particularly at homogeneous terrain sites (ESS and PIE) (Figures S5 and S6). The sensitivity of RDA models to spatial resolution was assessed for the eight variable sets (Table 2) at each site using two metrics: (i) the model's adjusted‐*R* ^2^ as an indicator for genetic variance captured by the predictor variables, and (ii) the proportion of the high‐impact SNPs that were detected as outlier loci among the investigated high‐impact SNPs (p_S_) to represent the signatures of selection detected by the model. The low adjusted‐*R* ^2^ values of Figure 1 reflect that raw *R* ^2^ values were divided by the number of input variables used, which ranged between seven to 31 (see Table 2 for number of input variables and Figure S11 for raw values). VS*‐single* model adjusted‐*R* ^2^ values reflected variable selection order (Table 3): explained genetic variance was maximized with coarser resolutions (VS*‐16m*) at homogeneous sites and with intermediate resolutions (VS*‐2m* to VS*‐8m*) at heterogeneous sites (Figure 1a). Meanwhile, the proportion of the high‐impact SNPs detected as outlier loci (p_S_) varied amongst variable sets and local sites (Figure 1b). At PAR and PIE, p_S_ increased with coarser resolutions in VS*‐single* models, whereas ps was very low for all VS*‐single* models at ESS except for a peak with the VS*‐2m* model. At MAR, p_S_ peaked at VS*‐0.5m* and VS*‐8m*. At the regional level, RDA models had much lower adjusted‐*R* ^2^ values (Figure 1a) and detected extremely low numbers of high‐impact SNPs as outliers (low p_S_; Figure 1b) when compared to the local analyses, regardless of variable spatial resolutions or number of predictor variables.

In support of using a forward selection procedure to incorporate multi‐resolution variables, the VS*‐fwd* models for all sites had similar metrics to the highest‐performing VS*‐single* models, except at MAR where VS*‐fwd* performed relatively poorly (Figure 1). Although the VS*‐all* models had the highest raw *R* ^2^ at each site (Figure S11A), this was attributable to the large number of explanatory variables used. Indeed, VS*‐all* had the lowest adjusted‐*R* ^2^ at all sites when corrected for the number of input variables (Figure 1a) and detected relatively low proportions of high‐impact SNPs as outliers (Figure 1b; Figure S11B). Because of overfitting in the VS*‐all* model, we focus subsequent GEA interpretations only on VS*‐single* and VS*‐fwd* models, where comparisons with VS*‐all* can be found in the relevant Appendix S2.

A deeper understanding of RDA model sensitivity to differences in spatial resolution was obtained by comparing the identity of the high‐impact SNPs detected as outliers between variable sets at each site (Table 4; visualized using UpSet plots in Figure S12). Overall, most RDA models at a given site detected common outlier loci, with few models detecting >10% unique SNPs. Almost all outlier loci at PAR and PIE were identified by at least two variable sets, regardless of how many high‐impact SNPs were detected. In contrast, four VS*‐single* models at ESS and two at MAR detected 20%–80% unique SNPs, which was unrelated to the absolute number of high‐impact SNPs detected.

**TABLE 4:** Summary of GEA results performed using RDAs for the four sites (ESS, MAR, PAR, and PIE) separately (local; A–D) and grouped (regional; E). Values indicate the number of high‐impact SNPs, genes and GO terms detected as significant outliers in GEA analyses. *GO terms* are separated into BPs and MFs.

	Candidate SNPs	Candidate genes	GO terms: BP	GO terms: MF
(A) ESS				
VS*‐0.5m*	57	53		2
VS*‐1m*	38	29		3
VS*‐2m*	832	712	3	2
VS*‐4m*	70	59		1
VS*‐8m*	51	47	2	1
VS*‐16m*	115	96	2	2
VS*‐fwd*	642	546	8	5
(B) MAR				
VS*‐0.5m*	431	359	3	
VS*‐1m*	91	74	1	
VS*‐2m*	328	281	1	1
VS*‐4m*	261	233	1	
VS*‐8m*	491	402	1	
VS*‐16m*	403	335	1	
VS*‐fwd*	145	131	2	
(C) PAR				
VS*‐0.5m*	865	721	2	1
VS*‐1m*	1203	989	1	3
VS*‐2m*	1304	1050	1	5
VS*‐4m*	1762	1389	1	6
VS*‐8m*	1924	1495	1	4
VS*‐16m*	1963	1508	1	5
VS*‐fwd*	1837	1450		4
(D) PIE				
VS*‐0.5m*	53	54	2	1
VS*‐1m*	249	196	6	5
VS*‐2m*	309	246	1	
VS*‐4m*	522	403	7	7
VS*‐8m*	995	757	10	8
VS*‐16m*	1078	822	5	7
VS*‐fwd*	1064	823	6	8
(E) Regional				
VS*‐0.5m*	59	41		
VS*‐1m*	66	47		
VS*‐2m*	73	56		
VS*‐4m*	64	49		
VS*‐8m*	55	45		
VS*‐16m*	78	59		
VS*‐fwd*	448	227	3	2

### 3.4 Genotype–environment associations

Genotype–environment interactions were investigated by associating each high‐impact outlier SNP to a DEM‐derived variable based on projections in the RDA space (Table S8), where the locus distributions were generally insensitive to variable sets (Figure S13). The high‐impact candidate SNPs were then used to allocate the corresponding genes (hereafter “high‐impact genes”; Table 4) to an environmental variable and, rarely, to multiple variables (generally ≤10% per local model and <20% for the regional model; Tables S7 and S9).

**FIGURE 2:**
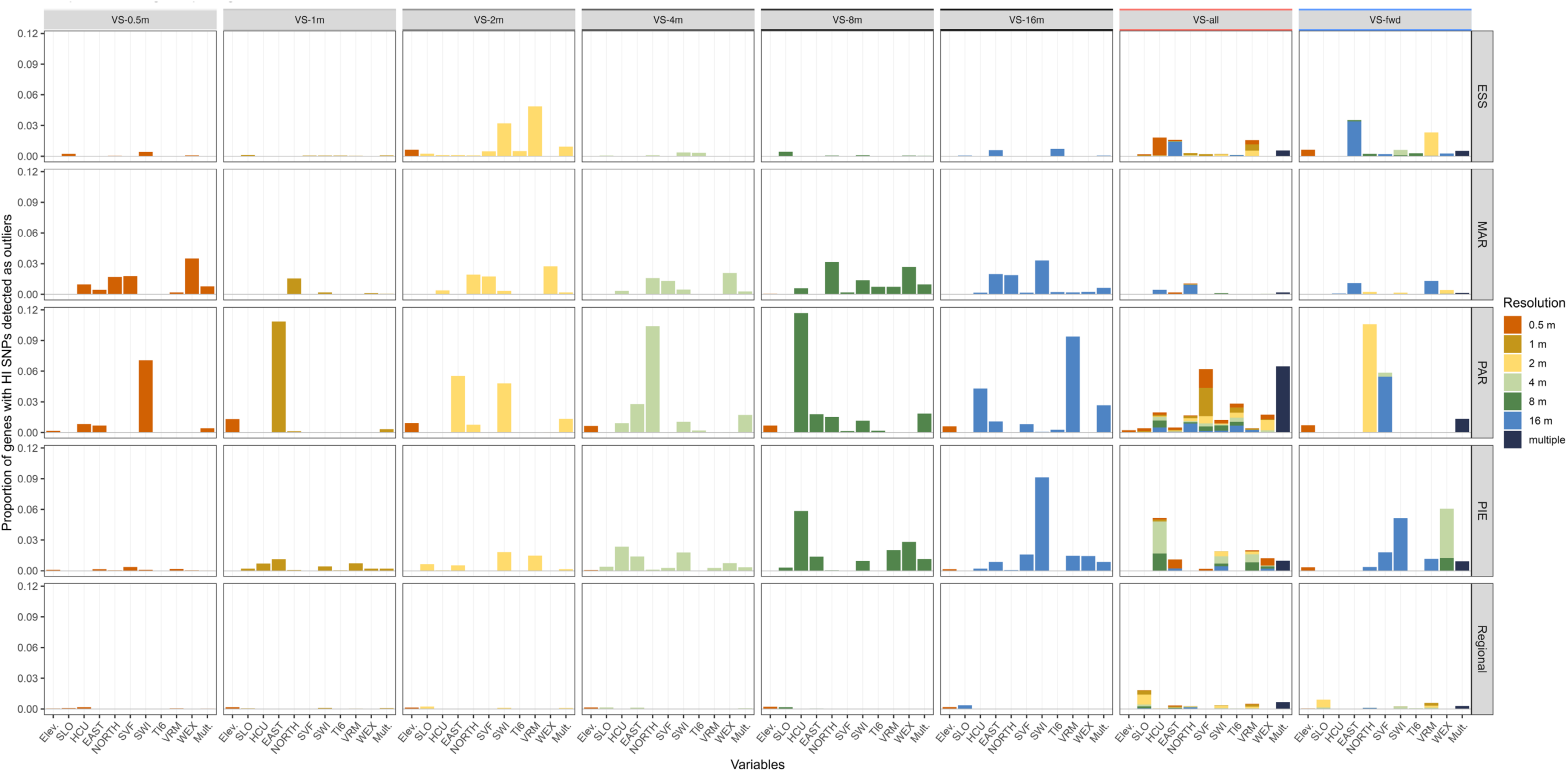
The proportion of high‐impact genes with outlier SNPs for the four sites (ESS, MAR, PAR, PIE) separately (local) and grouped (regional), grouped by the variable sets used as explanatory variables in the RDA models (columns). The *x*‐axis shows the associated variables, colored by the spatial resolution (grain size) for the variable. In cases where a gene had multiple SNP–variable associations, this was listed as “multiple”. Descriptions of variable sets found in Table S1. See Table S9 for raw counts of high‐impact SNP–variable associations in each gene.

The spatial resolutions of associated environmental variables reflected the selection order for VS*‐fwd*, where associated variables varied greatly across the genome, among sites and between variable sets (Figure 2; Table S9; Figure S14). Associations at homogeneous sites were dominated by coarser resolution variables (≥2 m), where top associations at ESS were with EAST, VRM, SWI, and HCU, and at PAR with SWI, HCU, and WEX. Associations at heterogeneous sites were with variables across a greater range of spatial resolutions, where top associations at MAR were with NORTH, WEX, and SWI, and at PIE with HCU, EAST, NORTH, and VRM. Surprisingly, despite being one of the first forward‐selected variables to explain genetic variation, elevation was rarely associated with genes at the local sites (generally <4%). At the regional level, elevation was a top‐associated variable with high‐impact genes (up to 45% in some VS*‐single* models, but only 2% in VS*‐fwd*), alongside SLO, EAST, and VRM. The variables NORTH, SWI, WEX, TI6 and HCU were rarely associated with high‐impact genes at the regional level.

The functional significance of high‐impact genes was investigated for each variable using GO enrichment analyses (Table 4). Significantly associated functions were largely unique to each site and detected by different variables (Table S2). Overall, only two molecular functions (MFs) and two biological processes (BPs) were detected across multiple sites, with one of each detected at ESS and in the regional analysis, which predominantly related to oxidation or cellular stress responses (Table S2). Cellular response to cold (GO:0070417), for example, was detected at PIE with VRM 16m and at MAR with WEX 4m. The variables associated with significant gene functions followed patterns from the top gene–variable associations. Plants at homogeneous sites generally presented more candidate genes with putatively adaptive functions, where the variable type exerting a potential selection pressure was relatively consistent amongst variable sets. Plants at heterogeneous sites contrastingly presented fewer candidate genes with adaptive functions that tended to be associated with different variable types and spatial resolutions. VS*‐fwd* models captured many of the same significant functions and putative processes under selection as the VS*‐single* models, though not always with the same variable type. Analyses at the regional level detected relatively few significant GO terms and only with the VS*‐fwd* model (Table 4), consistent with previous results highlighting a lack of power to detect local adaptation with these spatial resolutions.

**FIGURE 3:**
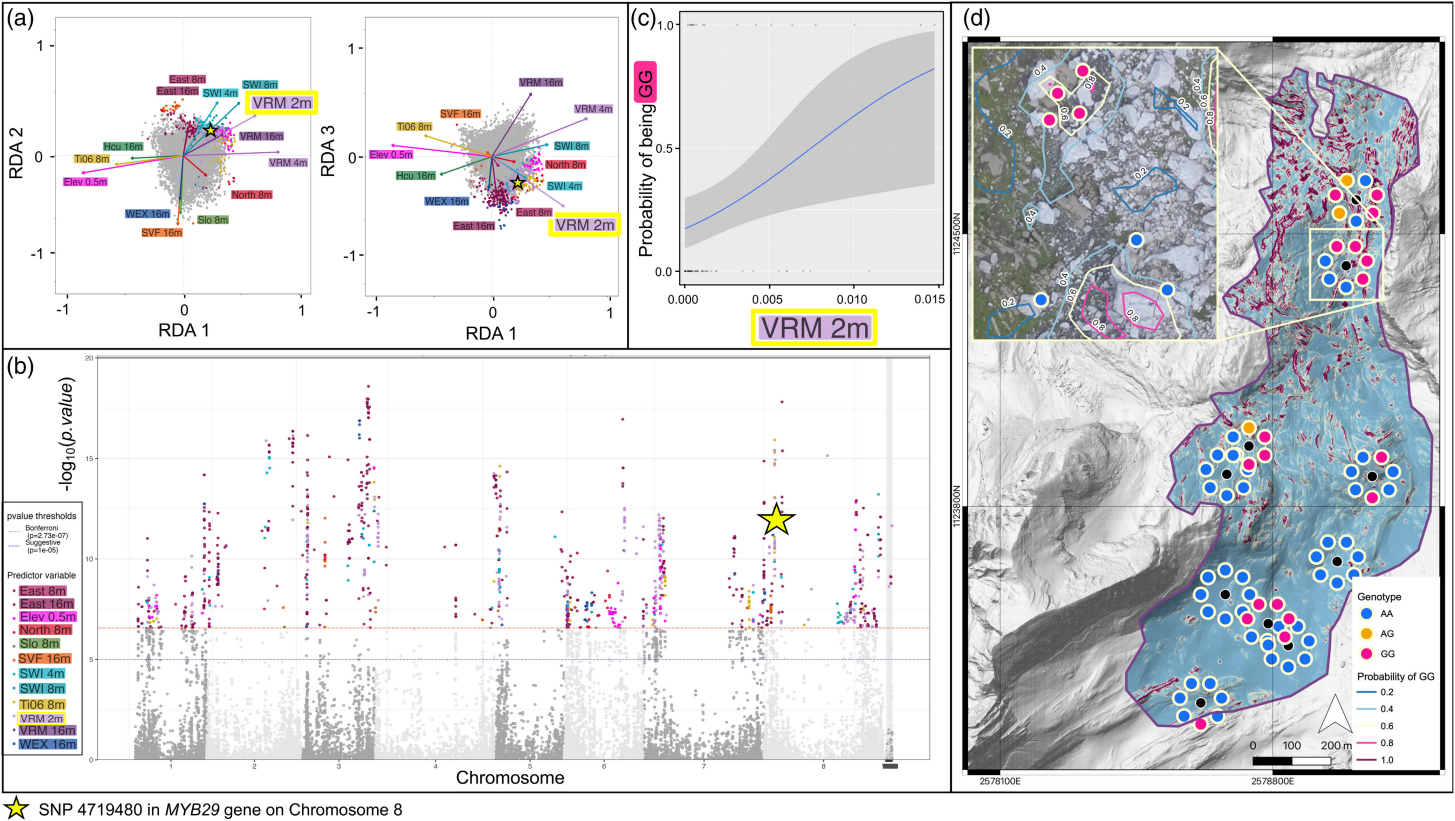
An example at study site Essets (ESS) to illustrate how a GEA model built with forward‐selected variables (VS*‐fwd*) can be used to detect a candidate SNP under selection and associate it with a given environmental variable (VRM at 2 m). Biplots (a) show the loading distribution of SNPs (points; multiplied by 20 to ease visualization) and environmental variables (arrows) across the first three RDA axes, where the projections in the RDA space were used to assign each outlier locus to the predictor variable with the largest absolute scalar value. The outlier loci are color‐coded to the most associated predictor variable of the same colored label. The location and significance of SNPs across the genome was visualized with a Manhattan plot (b), where outlier SNPs are color‐coded by their associated variable. In this example, locus 4719480 in the Aa_G76360.h1 gene of the MYB29 complex on chromosome 8 (indicated by yellow star in (a) and (b)) was most strongly influenced by VRM at 2 m. The logistic regression between the GG genotype at this locus with VRM at 2 m (c) was used to calculate the probability of finding the GG genotype across the ESS (d).

Interestingly, using multiscale DEM‐derived variables captured evidence of biotic pressures on plant local adaptation. Using the VS*‐fwd* RDA model at ESS, a significant association was found between VRM 2m and the Aa_G76360.h1 gene of the MYB29 complex, which is involved in *A. alpina* adaptive response to insect herbivory (GO:0009625). The RDA biplots highlight that the associated high‐impact SNP is strongly associated with VRM 2m, and slightly less strongly with SWI at coarse resolutions (star in Figure 3a). This SNP is also found in a genomic region that appears to have many genes potentially under selection on Chromosome 8 (Figure 3b). The positive correlation between the homozygous recessive genotype (GG) for this SNP given the values of VRM 2m was modelled using linear regression (Figure 3c), which was used to produce a probability map of finding the GG genotype across the extent of ESS (Figure 3d). This map highlights that the GG genotype has a higher probability of being found in rougher terrain areas with less soil build‐up, which could reduce insect herbivory pressure.

## 4 DISCUSSION

By coupling DEM‐derived h‐topographic environmental variables at multiple grain sizes with whole genome sequence data, we highlighted the sensitivity of GEA models to spatial resolutions. These findings illustrate the importance of incorporating multiscale variables into studies of local adaptation. Furthermore, we emphasise that the paradigm of using the finest resolution variables possible for modelling local adaptation in sessile organisms may not always hold, and indeed could introduce noise to models (Guillaume et al., 2021; Pradervand et al., 2014). Here, we discuss how spatial scale affects the relevance of DEM‐derived variables in GEA models with regards to study extent and variable type, and provide suggestions for integrating multiscale variables into GEA models.

### 4.1 Spatial scale matters

Systematic comparisons of GEA models highlighted that the spatial resolution of environmental variables matters. Variables with grain sizes between 2 and 16 m generally improved GEA model performance for the alpine plant investigated, where optimal resolution depended on variable type, terrain characteristics, and study extent. Furthermore, the same variable type was often selected at multiple spatial resolutions. This lack of specificity for an optimal spatial resolution reflects findings from multiscale species distribution models (Guillaume et al., 2021; Guisan et al., 2007), as well as GEA analyses based on low‐resolution genetic markers (Leempoel et al., 2018). While strong correlations were found when the same variable type was generalized to similar spatial resolutions, this trend weakened between more different grain sizes. Indeed, the same environmental variable at different spatial resolutions can capture distinct ecological processes and climatic conditions (Keitt & Urban, 2005; Lassueur et al., 2006; Leempoel et al., 2015) with subsequent impacts on model results, downstream analyses, and interpretations (Dungan et al., 2002). It is extremely difficult to determine exactly which spatial resolution is optimal for a given context, supporting the inclusion of the same variable type generalized to multiple spatial resolutions simultaneously in evolutionary ecology modelling.

Contrary to initial expectations (Gottfried et al., 1998; Lassueur et al., 2006), no single spatial resolution was identified as the most appropriate for any variable type. Generally, primary terrain variables (i.e., SLO, HCU and EAST/NORTH) were selected at coarser grain sizes (8–16 m), whereas secondary terrain variables were often relevant across a range of resolutions. Primary terrain attributes may be favoured at coarser resolutions due to smoothing over of the higher details present at finer resolutions (Kalbermatten et al., 2012), resulting in variables that better represent landscape processes relevant to the organism (Pain, 2005). In contrast, the relevance of secondary terrain attributes at finer grain sizes may be because they are specifically designed to model ecologically relevant hydrological, geomorphological, and BPs (Wilson & Gallant, 2000). Secondary terrain attributes representing solar radiation and sky availability (TI6, SVF) were selected at resolutions between 4 and 16 m, while variables of rugosity (VRM), soil wetness (SWI) and wind exposure (WEX) were selected across a broader range of resolutions between 1 and 16 m. That topographic effects on light are optimized at coarser resolutions than those on rockiness, water drainage and exposure might be due to their respective interactions with topographic features. For instance, primary attributes of eastness and northness proxy for sunlight availability, impacting near‐surface temperatures and photosynthetic rates (Bennie et al., 2008; Gottfried et al., 1998; Moore et al., 1991). As light and shade are influenced by larger‐scale topographic features (e.g., mountain crests, boulders) with strong seasonal fluctuations, these proxies may have coarser scale effects on vegetation and adaptive responses than more complex secondary attributes independent of large topographic features and seasonal variations (Keitt & Urban, 2005). However, without directly measuring an association between topographic variables and in‐field conditions (e.g., climate, soil chemistry, etc.), it remains difficult to assess the actual selection pressure that these variables exert on organisms. Indeed, selective pressures in natural environments are rarely known with certainty, and the hypothesized associations detected between genotypes and topographic variables require further validation via in‐field observations or experiments (Lasky et al., 2023).

General landscape topography is also important in dictating the appropriate grain size for a variable (Pain, 2005). In the present study, GEA models were optimized with variables selected at mid to coarse (4–16 m) resolutions for homogeneous terrain sites (ESS and PIE), and with a combination of variables at fine to coarse (1–16 m) resolutions for heterogeneous terrain sites (MAR and PAR). Differences in terrain heterogeneity likely reflects the scale at which local adaptation is occurring. Indeed, models of abiotic (e.g., Thompson et al., 2001) and biotic (e.g., Guillaume et al., 2021) factors have highlighted that finer details in variables are required at heterogeneous terrains, while natural processes at homogeneous terrains are smoothed over and require coarser resolution variables to represent environmental processes.

The spatial resolution of variables must appropriately reflect landscape processes likely affecting the study organism to ensure that signatures of local adaptation are detected (Anderson et al., 2010; Cushman & Landguth, 2010). Here, the finest spatial resolution of 0.5 m typically resulted in the lowest model performances at local sites, while model performances increased toward the coarser resolutions between 4 and 16 m. Despite the possible influence of pseudo‐replication arising from the inclusion of up to 10 samples in a grid upon generalization, the conservative thresholds used to detect outlier loci, and the fact that RDA performance metrics (adjusted‐*R* ^2^ and ps) were maximized with variables at any of the tested grain sizes, indicates that the observed patterns remain valid.

These results call for a need to reassess the general paradigm of increased landscape genetic model accuracy with finer grain variables (Cushman & Landguth, 2010), particularly when investigating sessile organisms in highly heterogeneous environments (Gottfried et al., 1998). This is not to say that variables at 0.5 m resolution should be discounted, as they can be ecologically relevant depending on variable type. Indeed, 0.5 m variables improved RDA model performance at one site (MAR) in the present study. Additionally, common enriched GO terms and similar BPs were detected between the present study and a univariate GEA analysis using topographic variables only at 0.5 m spatial resolution, including GO terms relating to defence responses at ESS (Rogivue et al., 2023). However, it was only when using the mixed resolution VS*‐fwd* model that enriched GO terms were detected at the regional level, which were missed in the VS*‐single* models. Therefore, we emphasise that a spectrum of potential ecologically important processes be captured using variables at multiple levels of complexity (Anderson et al., 2010; Cushman & Landguth, 2010), which can be done by integrating predictor variables at multiple nested spatial resolutions.

### 4.2 Local adaptation is local

Signatures of local adaptation associated with multiscale variables were specific for each population. Differences in candidate loci and gene functions putatively under selection were found between sites despite shared genomic background due to common recent history (Rogivue et al., 2018), potential parallel adaptation due to similar environmental pressures (Wos et al., 2022), and increased GEA model power through sampling across a range of habitats (Selmoni et al., 2020). Moreover, while each model detected relatively high number of candidate genes under selection, only four MFs and BPs were shared amongst multiple sites across the variable set models (Table 2), with none shared amongst all local sites. Similarly, in a re‐assessment of previously identified candidate SNPs using univariate GEA models, only 11 (31%) putative genes originally detected in one set of study populations of *Arabidopsis halleri* were found in an independent set of 18 other populations of the same species across the Swiss Alps (Rellstab et al., 2017).

The paucity of common enriched genes associated with high‐resolution topographic variables between four local populations in the present study echoes conclusions from a continental‐extent genome‐wide association study (GWAS) in *Arabidopsis thaliana* (Lopez‐Arboleda et al., 2021) that emphasized the detection of molecular patterns are highly sensitive to sample design and population structure, with some phenotypic traits influenced by distinct genetic effects in each subpopulation. Such specification of local adaptation is likely exacerbated in the present study due to limited gene flow in highly selfing populations (Buehler et al., 2012; Zeitler et al., 2023) that experience high LD due to reduced recombination rates (Nordborg, 2000; Yant and Bomblies, 2017). Additionally, as complex phenotypic responses are generally controlled by many SNPs of small effect sizes (Höllinger et al., 2019), which has been shown in populations of this species (Zeitler et al., 2023), selection likely results in multiple molecular solutions converging on similar functional phenotypic responses in different populations (Lasky et al., 2023; Yeaman, 2015). Very few outlier loci were detected at the regional level when compared to the local RDAs. Despite the reduced false discovery rates and increased power of RDA analyses to detect multilocus adaptation compared to traditional univariate GEA methods (Capblancq et al., 2018; Forester et al., 2018), signals of parallel local adaptation due to polygenic traits may still be missed (Le Corre & Kremer, 2012; Lotterhos, 2023; Rellstab et al., 2017). This is exacerbated in selfing populations for which the combined effects of lower effective population sizes, increased homozygosity, reduced effective recombination, stronger drift, greater linkage of beneficial mutations to deleterious ones, and the higher rate of fixation of maladaptive alleles, makes it extremely difficult to interpret patterns of adaptation and strength of selection pressures (Yant and Bomblies, 2017). Additionally, as this study was intended to assess the effect of multi‐resolution environmental variables on GEAs, rather than to perform an exhaustive evaluation of local adaptation, analyses were limited to high‐impact intragenic SNPs. As such, other outlier loci due to LD in intergenic and non‐high impact intragenic SNP regions may have been overlooked, where it may be more informative to use whole‐genome SNP data than just putatively causal sites when an exhaustive search for loci under selection is required (Le Corre & Kremer, 2012; Lotterhos, 2023). Future studies might consider a polygenic framework, specifically investigating gene sets (e.g. polysel; Daub et al., 2013), which could help detect multiple small‐effect loci involved in local adaptation, and include intergenic regions within LD of intragenic SNPs into analyses.

### 4.3 Relevance of topographic variables

Topographic variables are powerful tools to investigate local adaptation in heterogeneous environments such as mountainous regions. Here, DEM‐derived variables across six spatial resolutions explained between 10% and 30% of intragenic variation at each study site based on the uncorrected *R* ^2^ model values, detecting high‐impact genes potentially under selection across the genome of *A. alpina*. Interestingly, these topographic variables captured evidence of BPs potentially involved in local adaptation, such as the association between VRM 2 m and molecular response to insect herbivory. This follows a suite of studies that have successfully used topographic variables to investigate plant distributions across heterogeneous alpine environments (e.g., Gottfried et al., 1998; Guillaume et al., 2021; Lassueur et al., 2006), as well as to detect genetic responses to environmental conditions (e.g., Leempoel et al., 2018). Their usefulness owes to the fact that topographic variables are specifically designed as proxies for ecologically relevant environmental variables, including solar radiation (Wilson & Gallant, 2000), terrain ruggedness (Sappington et al., 2007), soil wetness (Beven & Kdirkby, 1979), soil pH (Böhner & Selige, 2006) and climate (Gottfried et al., 1998).

It has been suggested that because DEM‐derived variables can capture small‐scale terrain variation that drives mosaics of microclimates across landscapes (Gottfried et al., 1998; Hörsch, 2003; Irl et al., 2015; Scherrer & Körner, 2011), they are more relevant for investigations of local adaptation at finer spatial scales than climatic variables (Fischer et al., 2013). This is not to say that climactic variables are irrelevant for investigating local adaptation. Indeed, because topographic‐induced micro‐climates can vary over short distances, using fine‐resolution climatic variables as predictors can improve the modelling of plant trait responses to alpine‐arctic conditions (e.g., Kemppinen & Niittynen, 2022; Scherrer & Körner, 2011). Rather, because these climatic variables must be interpolated from weather stations or relatively‐coarse‐grained remote sensed data (Gottfried et al., 1998), climatic variables may be more appropriate for regional‐level analyses (Irl et al., 2015). This may be particularly true for studies investigating long‐term, multigenerational processes relevant across larger geographical extents (Fischer et al., 2013).

Of the nine independent derived variables selected to proxy for ecologically relevant characteristics in the present study, the top‐associated variables were those representing hydrology (HCU, SWI), solar radiation (EAST/NORTH, SVF, TI6), climate (WEX) and terrain ruggedness (VRM), where associations with elevation and slope (SLO) were primarily only detected in the analysis at the regional level. The dependence of RDA models on grain size may be attributed to changes in interactions between variables with changes in spatial resolutions, where the ecological relevance of a given variable, and therefore its ability to detect genes under selection (Loke & Chisholm, 2022; Pradervand et al., 2014; Thompson et al., 2001), changes as it is generalized to different spatial resolutions (Dormann et al., 2013; Kalbermatten et al., 2012). Here, only the top variable associated with each SNP is described, yet it may be that another correlated variable is the one exerting a selection pressure on the detected loci. We found that up to 10% of high‐impact genes at local sites and 20% at the regional had outlier SNPs associated with different variables. Tight correlations between some variables across different spatial resolutions meant that the same GO terms were associated with different variables depending on the spatial resolution and the variable set used. These correlations amongst variables highlight that once SNP–environment associations are detected, the associations require further testing to validate the outlier loci and their drivers identified in the GEA (described in Lasky et al., 2023).

Elevation was included as a variable to act as a proxy for temperature and biotic factors in general (Ashcroft & Gollan, 2013; Hof et al., 2012). Despite being the first variable retained in the forward selection models, it was rarely associated with genes at local sites, though it was a top association in the regional VS*‐single* models. This is likely because elevation was highly confounded with other factors at local sites, including population structure, latitude, and some derived variables. The inclusion of elevation as a variable in species distribution modelling is debated (reviewed in Hof et al., 2012). The primary argument against its inclusion is that organisms do not respond to elevation per se, but rather to other variables that co‐vary. Even temperature, for which elevation proxies, has been shown to not contribute any substantial improvements in plant species distribution modelling (Pradervand et al., 2014), likely as temperature is dependent on topographic features including sky view and northness (de Villemereuil et al., 2018). Yet other studies across large spatial extents have found that elevation is amongst one of the most important factors for predicting forest distribution (Hörsch, 2003). As such, we urge caution when using elevation in GEA models, and suggest to instead include other topographical variables that may more specifically proxy for temperature and humidity at small scales (Buri et al., 2020), such as wetness indices, VRM, slope (Leempoel et al., 2015) and solar radiation levels during growing seasons (Körner, 2007).

### 4.4 Integrating multiscale variables into GEA models

The sensitivity of GEA models to grain size can make it challenging to incorporate variables at adequate spatial resolutions. This study supports a method to integrate variables at spatial resolutions optimized for a specific location by leveraging a multiscale approach (Woodcock & Strahler, 1987). Here, a fine‐grained DEM is first generalized to multiple spatial resolutions (Kalbermatten et al., 2012) from which topographic variables of interest can be derived. Then, a forward selection model (Blanchet et al., 2008) is used to retain only predictor variables at spatial resolutions most relevant for explaining genetic variation, specific to each site. The resulting VS*‐fwd* RDA models were generally just as good at explaining genetic variation as the top performing single resolution models (VS*‐single*). In addition to their high model performance, these VS*‐fwd* models detected the same signatures of selection as the VS*‐single* models, with very few uniquely identified outlier loci. Furthermore, only the VS*‐fwd* model detected enriched GO terms at the regional level, indicating that signatures of selection may be missed if using a single spatial resolution. These results support this method as an appropriate and convenient way to choose variables at multiple spatial resolutions for use in GEA models to detect candidate genomic regions under selection.

The forward selection model provides one predictive approach to maximise explained genetic variation without considering the ecological or mechanistic drivers of genetic variation (Mac Nally, 2000), where different subsets of predictor variables could be selected by the model due to small perturbations in the data (Araújo and Guisan, 2006). Consequently, such resulting associations between genotypes and the environmental variables need to be interpreted with caution. Indeed, correlations between selected variables may remain large when using the forward selection approach, such that it can be difficult to disentangle which variable is driving detected associations. Likewise, this approach may remove the variables involved in local adaptation, potentially impacting downstream GEA results and missing genomic regions under selection.

Alternative predictive or explanatory approaches to the forward selection model exist (Mac Nally, 2000). While PCAs can condense a large number of potentially collinear variables into fewer, synthetic variables, this approach should be reserved for when the ecological interpretation of variables to PCA loadings is straightforward (Rellstab et al., 2015). When raw variables are preferred, a pairwise correlation analysis can be used to retain only variables independent above a threshold of, e.g., |*r*| ≥ 0. 8 (Fischer et al., 2013), or using variation inflation factors (VIF) with a threshold of, e.g. <10, to iteratively remove the most highly correlated factors until only independent variables remain (Dormann et al., 2013). Alternatively, machine learning approaches, such as random forest algorithms (Genuer et al., 2010), can be used to select variables. The best selection method for a study will depend on the analytical goals or mechanistic understanding of the study system (Capblancq & Forester, 2021), and it would be interesting to test how these alternative variable selection methods compare to the VS*‐fwd* model in future research.

While the present study was focused on very high resolutions (i.e., ≤16 m), such fine resolutions below 30 m are not always available. The relevance of multi‐resolution variables between commonly‐acquired resolutions of 30 m to 1 km (e.g., Fick & Hijmans, 2017; NASA Shuttle Radar Topography Mission (SRTM), 2013) for detecting molecular signatures of adaptation would be interesting to investigate, particularly when researching mobile or highly dispersing organisms, or when conducting studies over large, regional study extents. Ideally, researchers would consider the necessary spatial scales that are hypothesized to be important for a particular context prior to sampling (Anderson et al., 2010; Capblancq & Forester, 2021), such that both environmental and genomic data are collected at appropriate spatial scales (Dauphin et al., 2023) to allow for the integration of multiscale approaches prior to commencing sampling. The finest grain size should be at least slightly smaller than the average home range or dispersal distance of an organism (Dale and Fortin, 2014), while the maximum grain size should dictate the spacing of individuals sampled.

### 4.5 Conclusions

Ecologists and evolutionary biologists incorporating environmental variables into their models must decide on the discrete spatial scale to use in each analysis, with consequences for model outputs and subsequent downstream analyses. Although guidelines to select the size and shape of sampling units have been available for over 25 years (e.g., Legendre & Legendre, 1998), researchers usually make arbitrary decisions with regards to selected spatial scale, often with little to no justification (Dauphin et al., 2023; Dungan et al., 2002). Here, systematic comparisons of GEA models highlighted model sensitivity to spatial resolutions of explanatory variables, where optimal model results depended on variable type, terrain characteristics, and study extent. A promising way to integrate optimal spatial resolutions into GEAs is using fine‐grain variables as the base of a multiscale generalization to produce variables at multiple spatial resolutions, before retaining only those that are most relevant for a particular context. It is becoming increasingly important to develop methods to effectively select explanatory variables at spatial resolutions appropriate for specific ecological questions, especially as high‐resolution environmental and genetic datasets become ever‐more readily available. The adoption of multiscale variables in applied conservation frameworks means that model outputs will have direct impacts on natural resource management decisions.

Only the multivariate RDA method was investigated here. This method was selected due to the more realistic representation of genotype–environment interactions (Lasky et al., 2023), while maintaining lower false‐positive and higher true‐positive rates than commonly used univariate GEA methods (Forester et al., 2018). Landscape genomics would benefit from further investigations into the sensitivity to spatial resolutions of univariate GEA models, including latent factor mixed modelling (LFMM; Frichot et al., 2013) and SamBada (Duruz et al., 2019; Stucki et al., 2017). Furthermore, investigating the multiscale variable concept into landscape genomic analyses in other environments, such as seascapes and riverscapes, would be interesting as environmental variables are becoming available at ever finer resolutions. A novel complementary approach that could be used to select relevant spatial resolutions and validate multiscale GEA findings involves using wavelets to decompose the spatial patterns of genotypes observed across landscapes (Lasky et al., 2022). We emphasise that while GEA models are most useful for generating hypotheses, the function of candidate loci must still be validated with field or laboratory studies. Going forward, the effect of spatial scale in evolutionary ecology models must be carefully considered, where studies will need to be designed taking multiscale variables into account. We encourage continued investigation into how to best incorporate multiple spatial scales into models and stress the importance of justifying choice of spatial resolutions.

## CONFLICT OF INTEREST STATEMENT

The authors have no conflicts of interest to declare.

## Supporting information


Appendix S1.

Appendix S2.

## Data Availability

All genomic and environmental datasets are publicly available through respective data repositories:
Raw reads are available on the NCBI (BioProject ID: PRJNA489364). SNP genotypes are available on DRYAD (https://doi.org/10.5061/dryad.58g217k). Very‐High Resolution Digital Elevation Models and delimitation of study areas are available on ZENODO (https://zenodo.org/records/11500754). Custom scripts are available upon request.
