# Supplementary material for: Integrating very high resolution environmental proxies in genotype–environment association studies
Source: Evol Appl. 2024 Jun 28;17(7):e13737. doi: 10.1111/eva.13737 (PMC11212006; doi:10.1111/eva.13737)
Supplement: Supplementary file 1 — Appendix S1. [file EVA-17-e13737-s001.zip › eva13737-sup-0001-Supinfo1.pdf]

Supplementary Figures

Supp Figure S1: Schema of methods.

Flow chart of the methods, where 304 *Arabis alpina* individuals were sampled across four valleys in the western Swiss Alps (sites indicated in red on the map).

Environmental data (green boxes): The environment of these sites was characterised using topographic variables obtained from digital elevation models (DEMs) at a finest resolution of 0.5m from LiDAR technology. The DEMs were generalised to multiple nested resolutions (1m, 2m, 4m, 8m, 16m) from which nine topographic variables were derived.

Variable sets (‘*VS*’; green boxes): Seven variable sets were produced to compare the effects of topographic variables generalised to multiple spatial resolutions on detecting signatures of local adaptation for the Variation Partitioning and GEA.

i-vi: ***VS-single*** each topographic variable at a single spatial resolution
vii: ***VS-all*** all of the topographic variables at all of the spatial resolutions
viii: ***VS-fwd*** a forward variable selection procedure was used to retain only the variables at *VS-all* that best explained intragenic SNP variation.

Genomic data (red/orange boxes): Published (Rogivue et al 2019) whole genome sequence data was obtained from each individual, where the single nucleotide polymorphisms (SNPs) were filtered and categorised into intergenic and intragenic SNPs. The intergenic SNPs (orange boxes) were pruned for linkage disequilibrium and used to estimate population structure. The intragenic SNPs (pink box) were used as response variables in redundancy analyses (RDA).

Variation partitioning (blue boxes): A partial RDA (pRDA) was used to partition the variance of the intragenic SNPs into demographic (population structure), geographic (geographic distance from X, Y coordinates), and environmental influences.

Genotype-Environment Association (GEA; purple box): An RDA was used to detect candidate intragenic loci under topographic selection, where a pRDA was used in the Regional analysis to account for population structure (orange box).

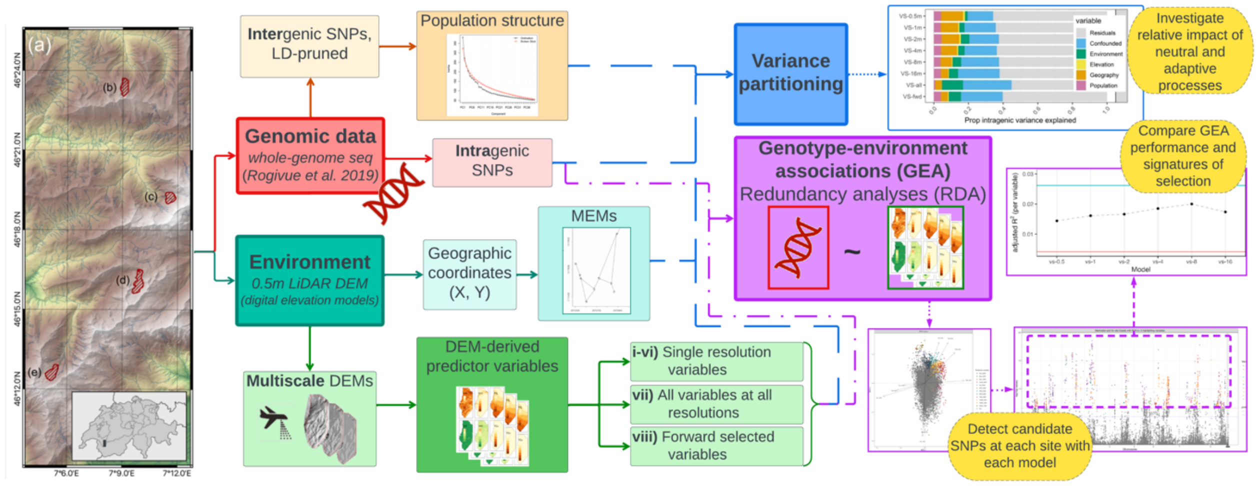

Supp Figure S2: Environmental variables.

Heatmaps of Spearman rank correlations (r_s) between the nine derived variables at 0.5m spatial resolution to test for independence. Values were obtained from the locations of the plant individuals across the four local sites **a)** Essets (n=70), **b)** Martinets (n=96), **c)** Para (n=69), and **d)** Pierredar (n=69), and at the **e)** regional level with all sites combined (n=304).

S2 a) Essets

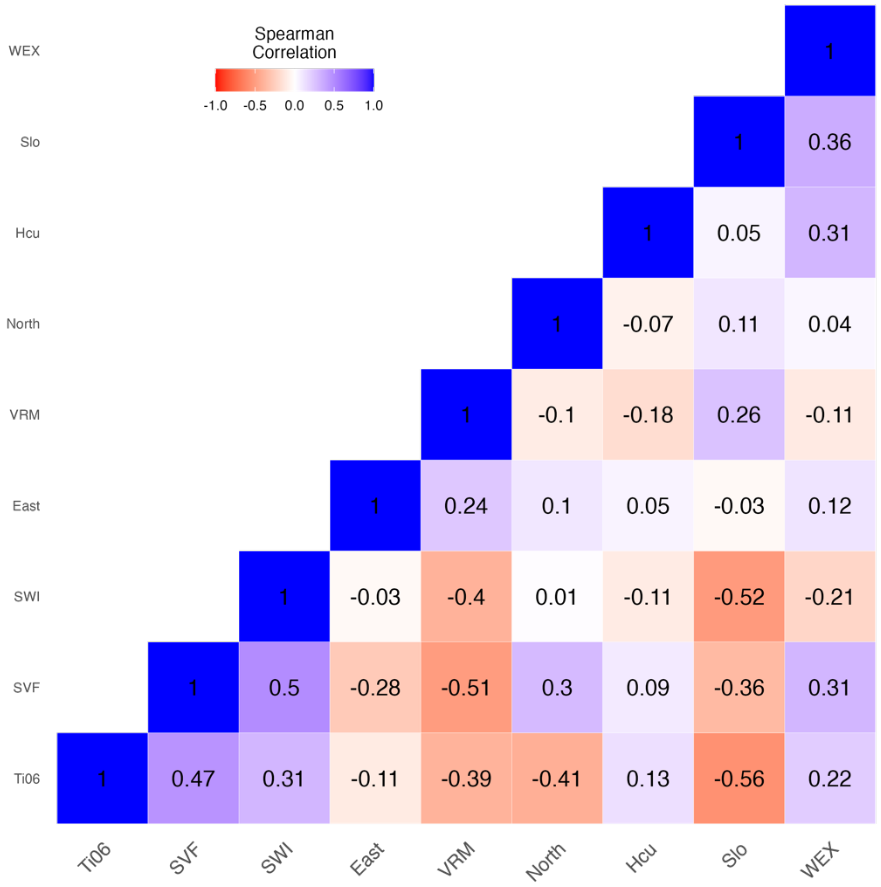

S2 b) Martinets

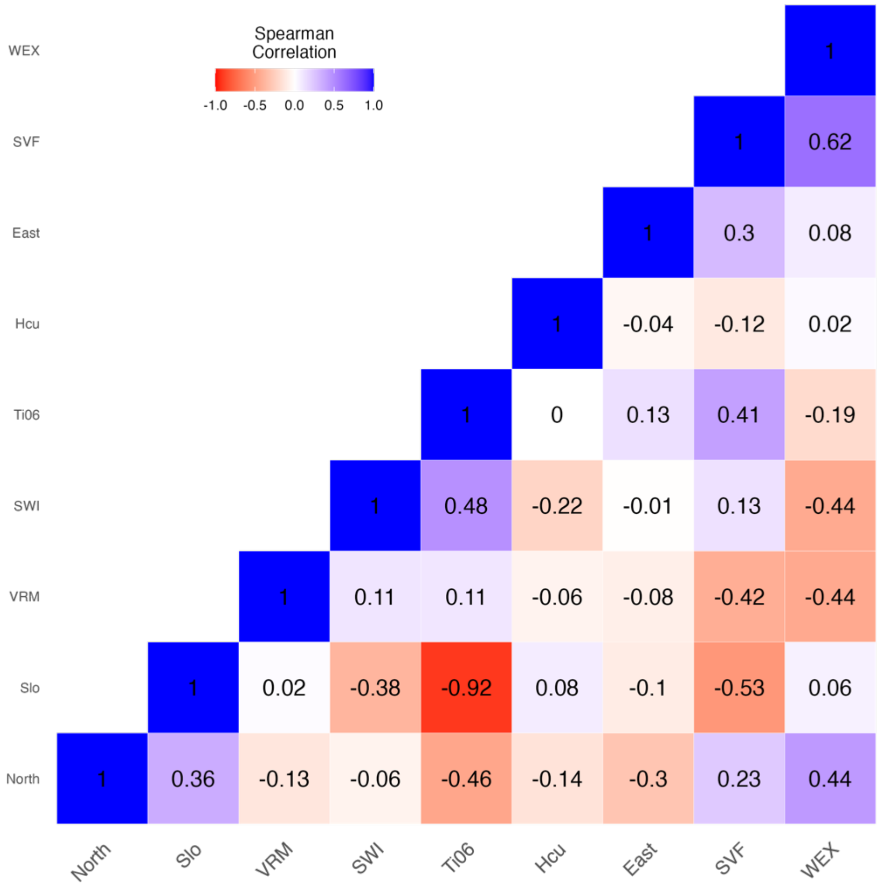

S2 c) Para

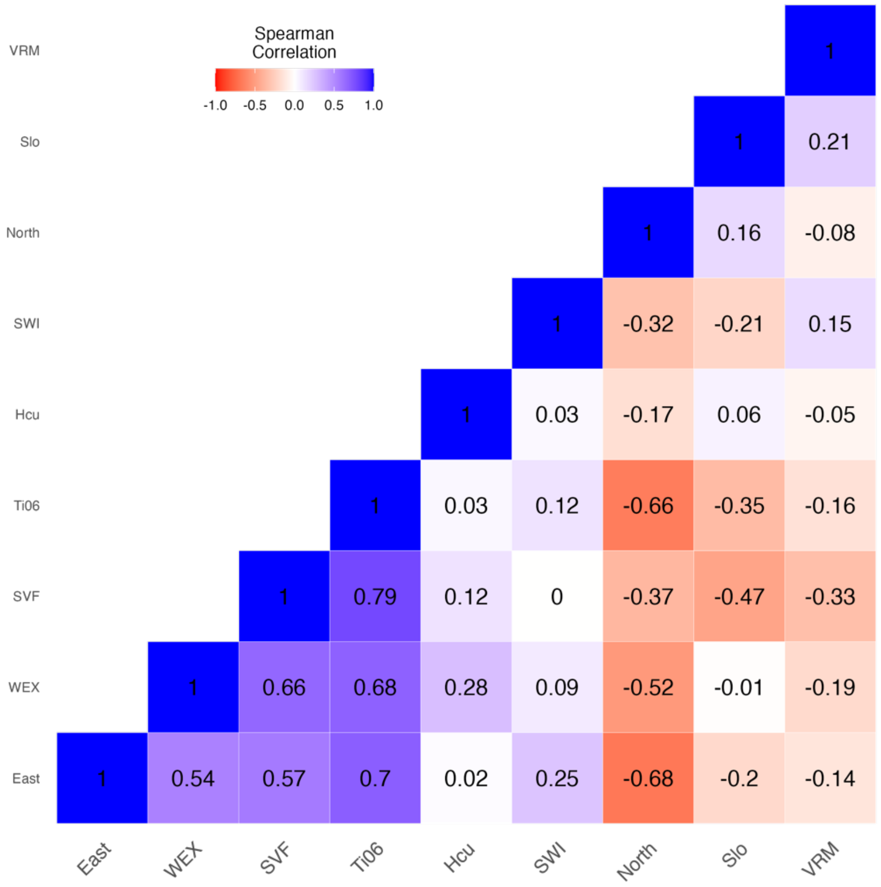

S2 d) Pierredar

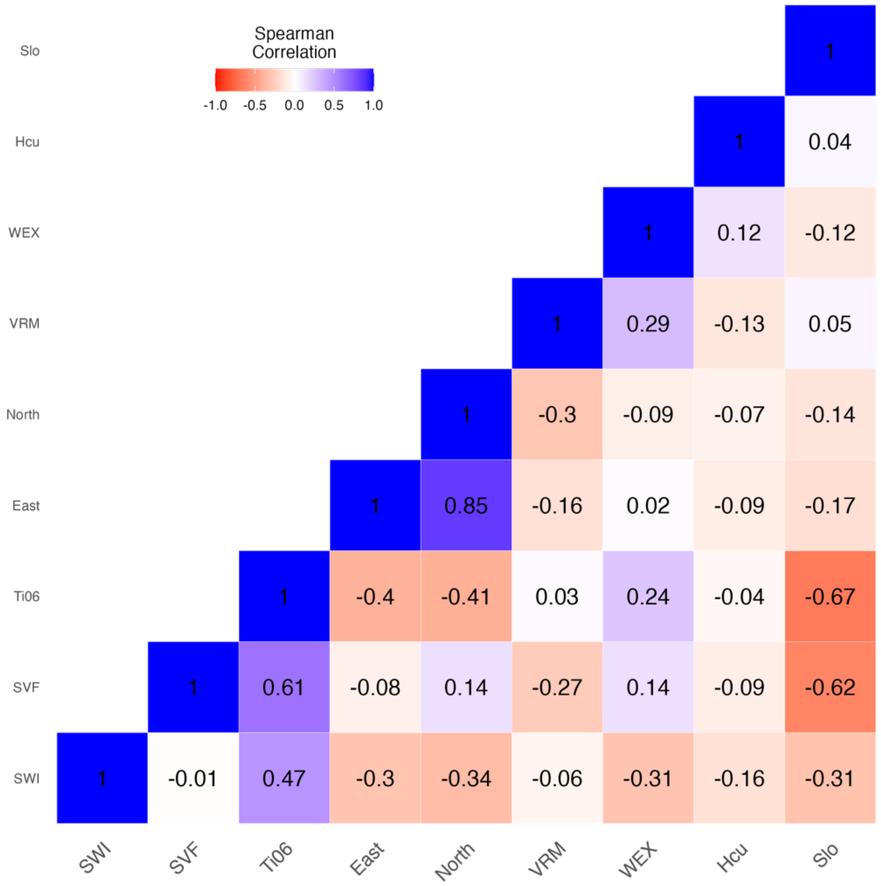

S2 e) Regional

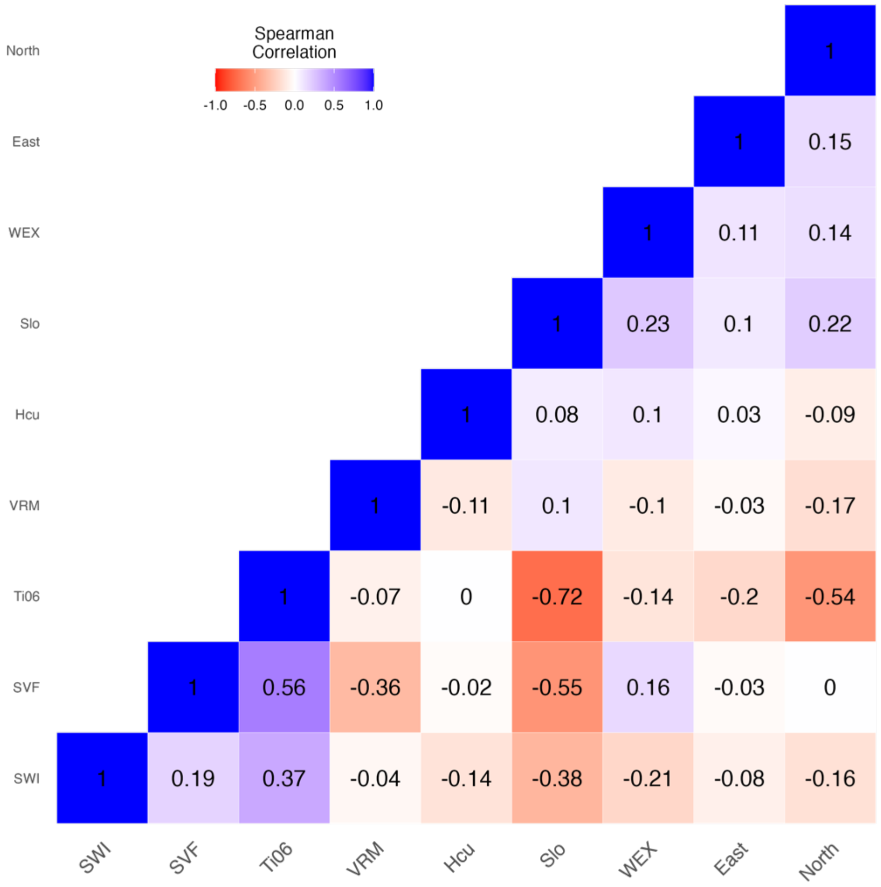

Supp Figure S3: Environmental variables.

Heatmaps of Spearman rank correlations (r_s) between the nine derived variables at all spatial resolutions (0.5, 1, 2, 4, 8, 16m), plus elevation at 0.5m spatial resolution. Values were obtained from the locations of the plant individuals across the four local sites **a)** Essets (n=70), **b)** Martinets (n=96), **c)** Para (n=69), and **d)** Pierredar (n=69), and at the **e)** regional level with all sites combined (n=304).

S3 a) Essets

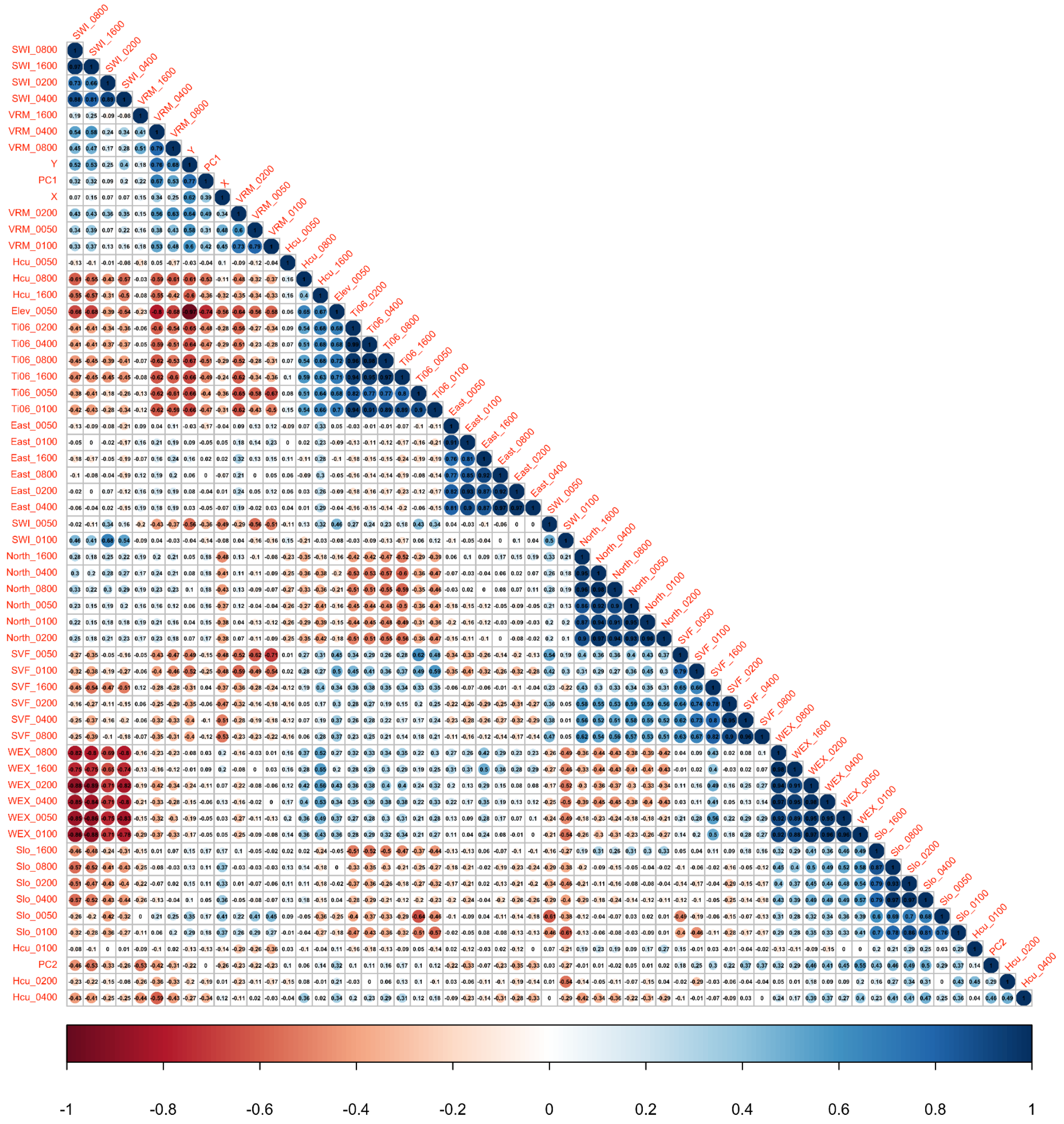

S3 b) Martinets

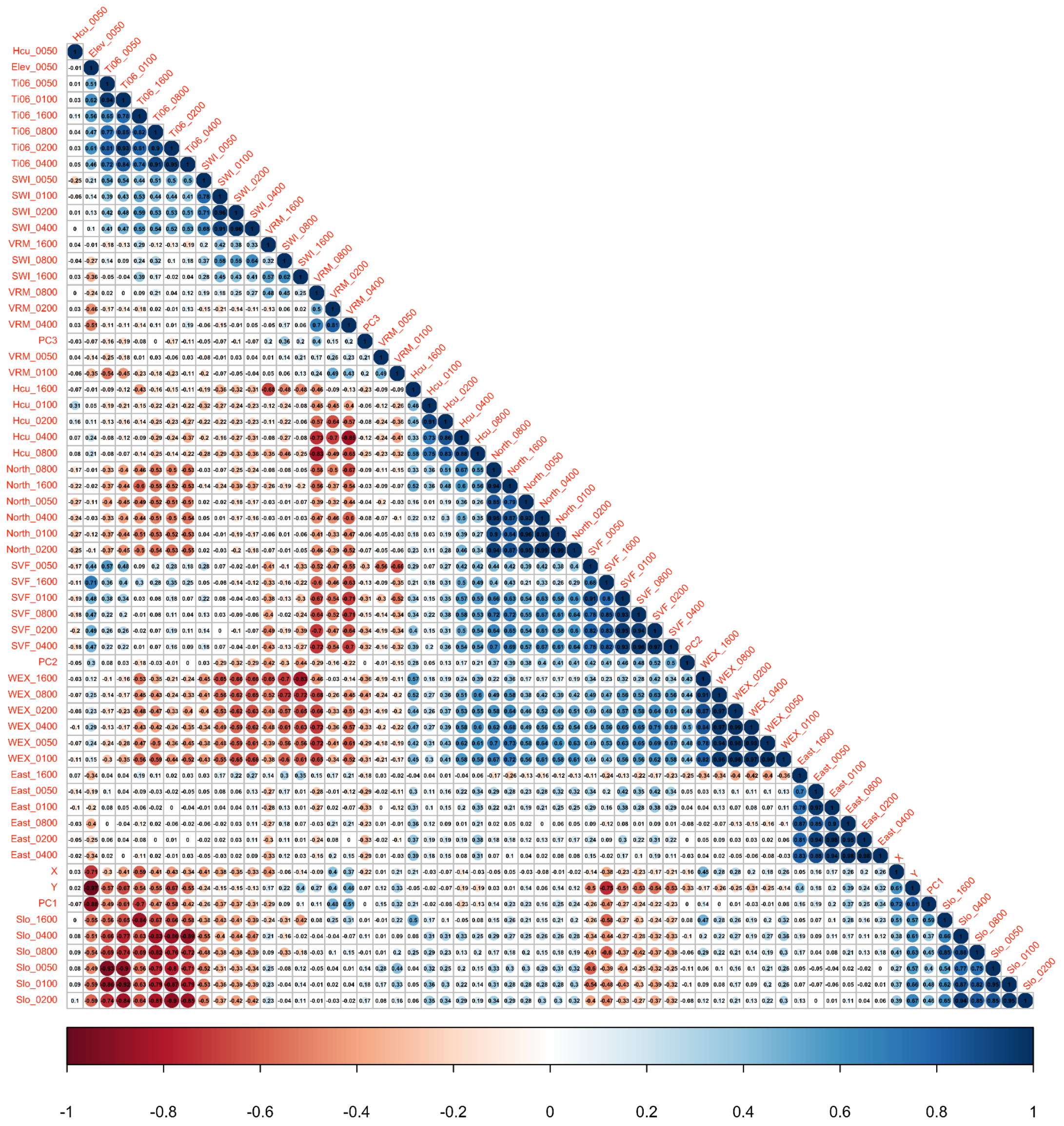

S3 c) Para

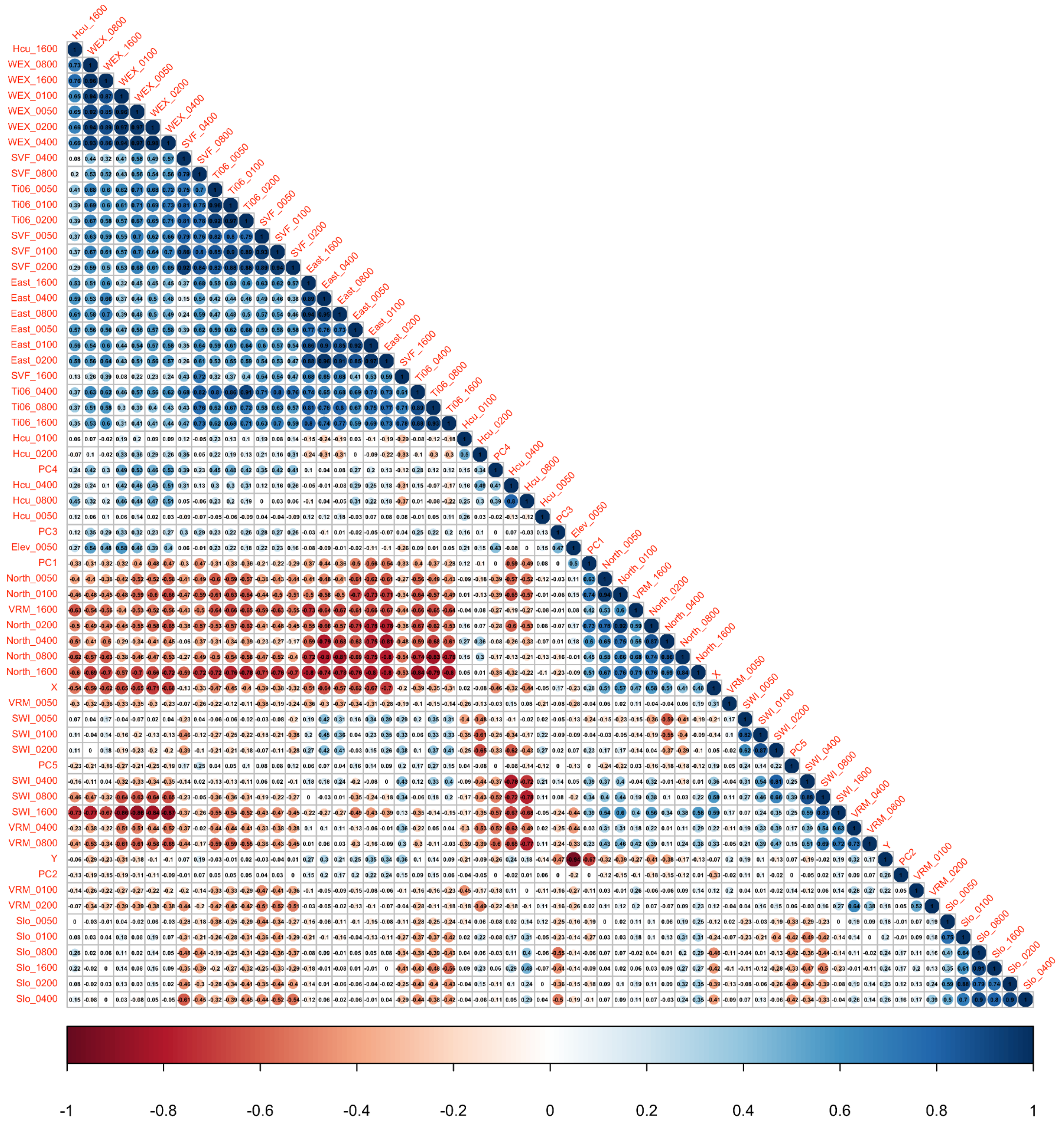
Hcu_1600
WEX_0800
WEX_1600
WEX_0100
WEX_0050
WEX_0200
WEX_0400
SVF_0400
SVF_0800
Ti06_0050
Ti06_0100
Ti06_0200
SVF_0050
SVF_0100
SVF_0200
East_1600
East_0400
East_0800
East_0050
East_0100
East_0200
SVF_1600
Ti06_0400
Ti06_0800
Ti06_1600
Hcu_0100
Hcu_0200
PC4
Hcu_0400
Hcu_0800
Hcu_0050
PC3
Elev_0050
PC1
North_0050
North_0100
VRM_1600
North_0200
North_0400
North_0800
North_1600
X
VRM_0050
SWI_0050
SWI_0100
SWI_0200
PC5
SWI_0400
SWI_0800
SWI_1600
VRM_0400
VRM_0800
Y
PC2
VRM_0100
VRM_0200
Slo_0050
Slo_0100
Slo_0800
Slo_1600
Slo_0200
Slo_0400
-1
-0.8
-0.6
-0.4
-0.2
0
0.2
0.4
0.6
0.8
1

S3 d) Pierredar

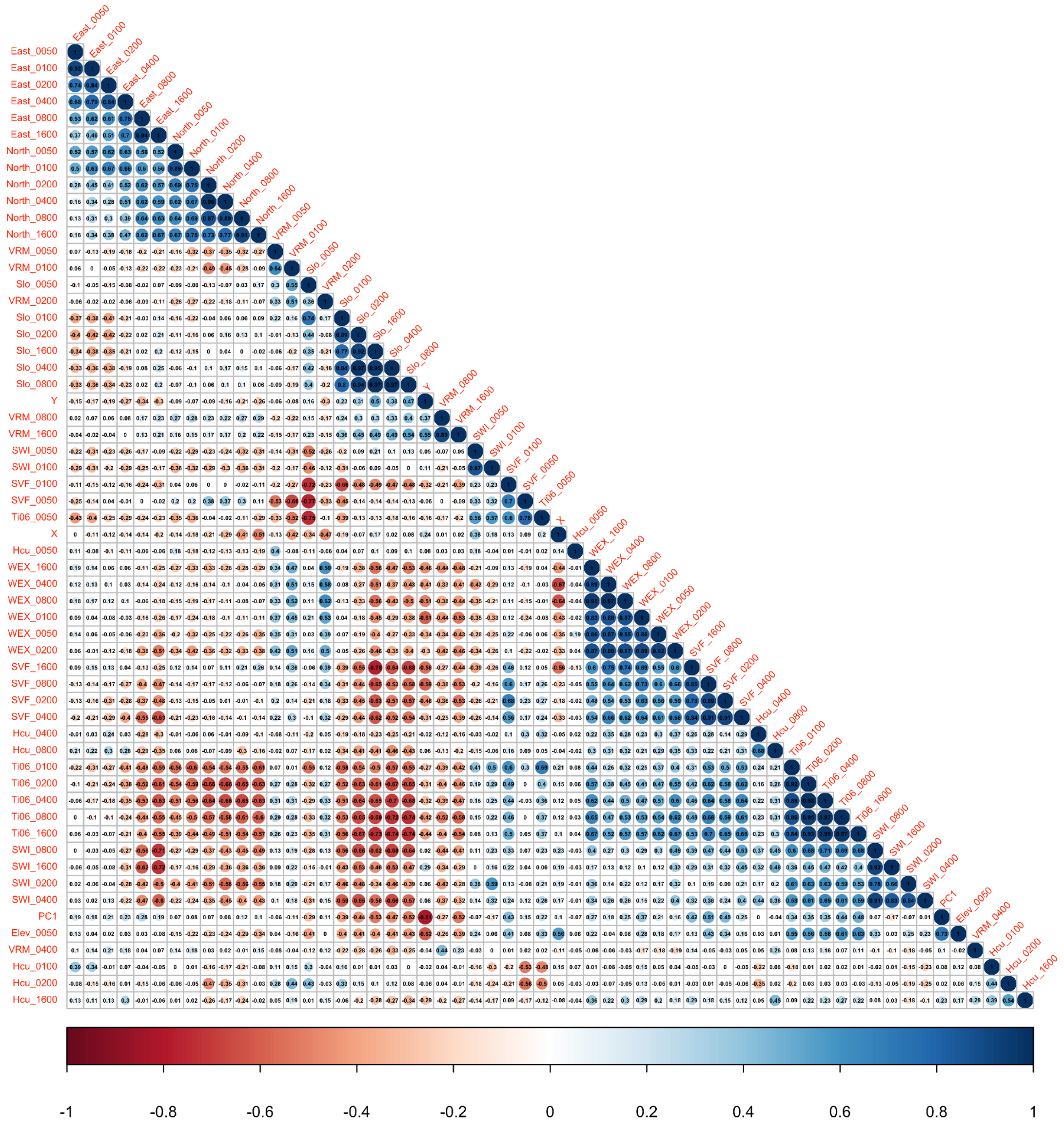

S3 e) Regional

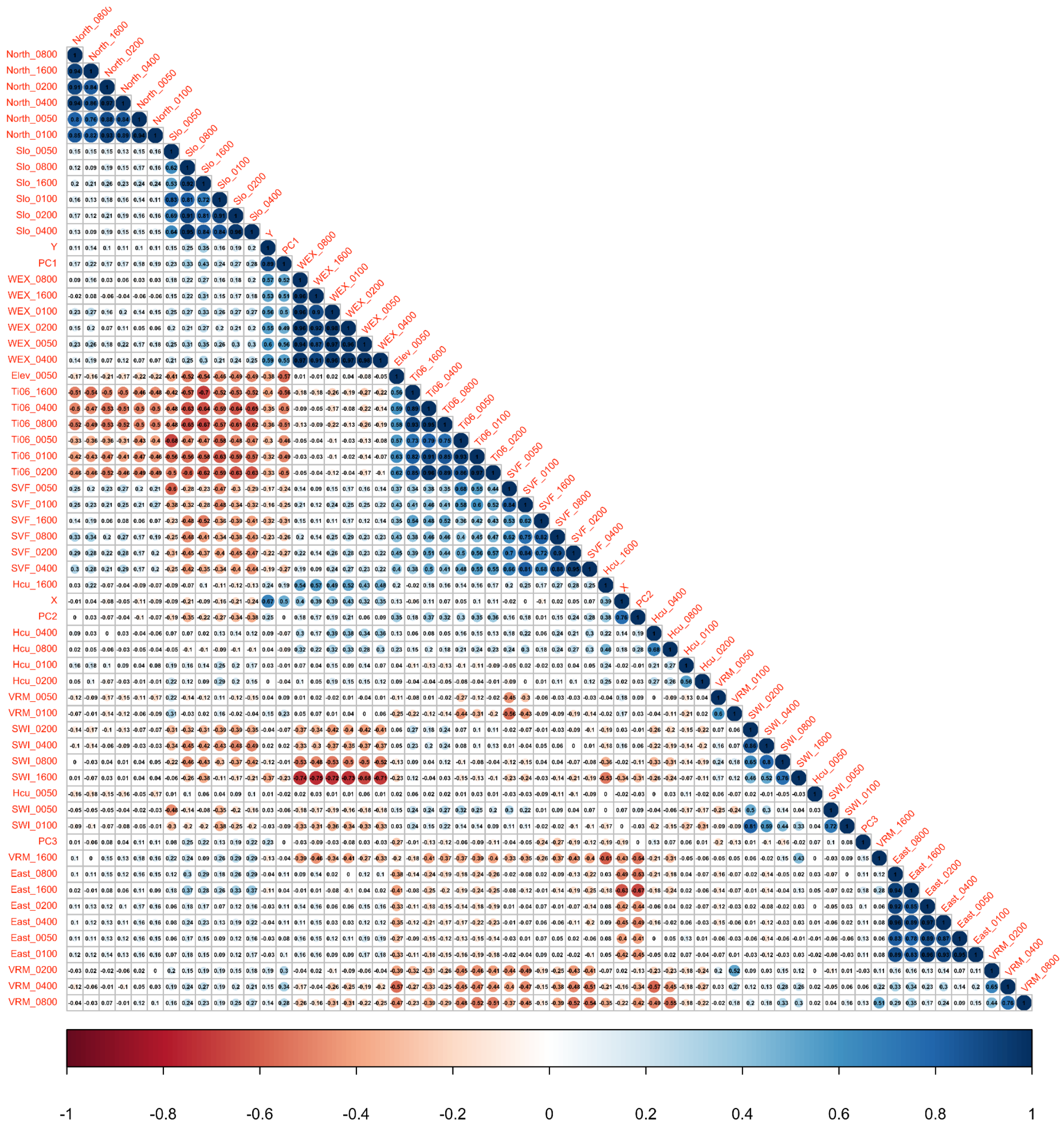

Supp Figure S4: Environmental variables.

Principal component analyses (PCA) of the nine derived variables at each spatial resolution (0.5, 1, 2, 4, 8, 16m) associated with each sampled individual, assessed across all four sites (n=304). Variables were centred and scaled prior to the PCA.

a) All variables at 0.5m spatial resolution

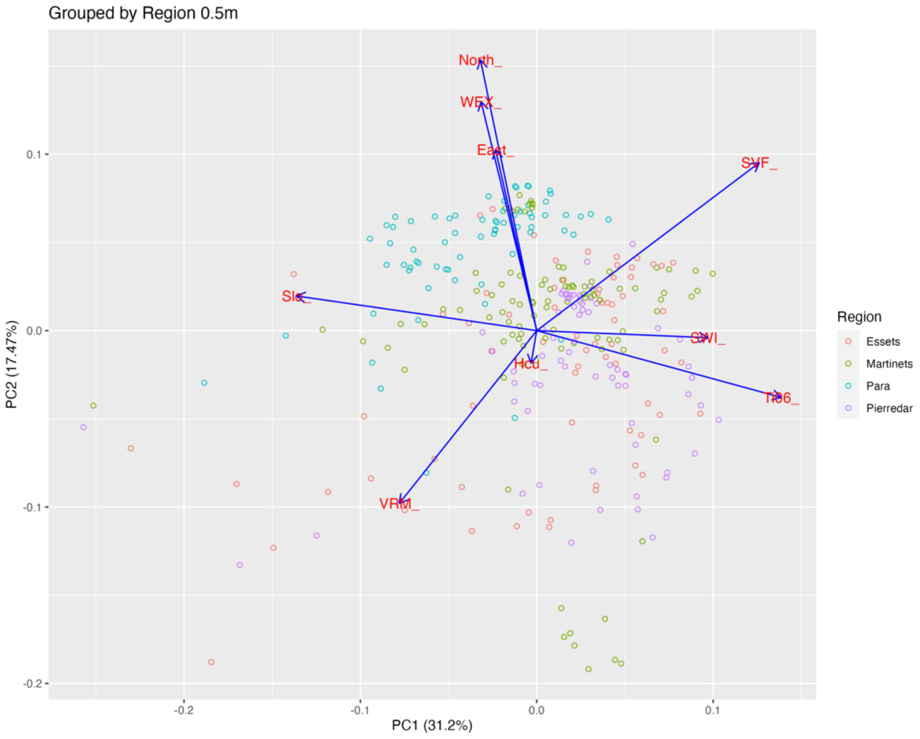

b) All variables at 1m spatial resolution

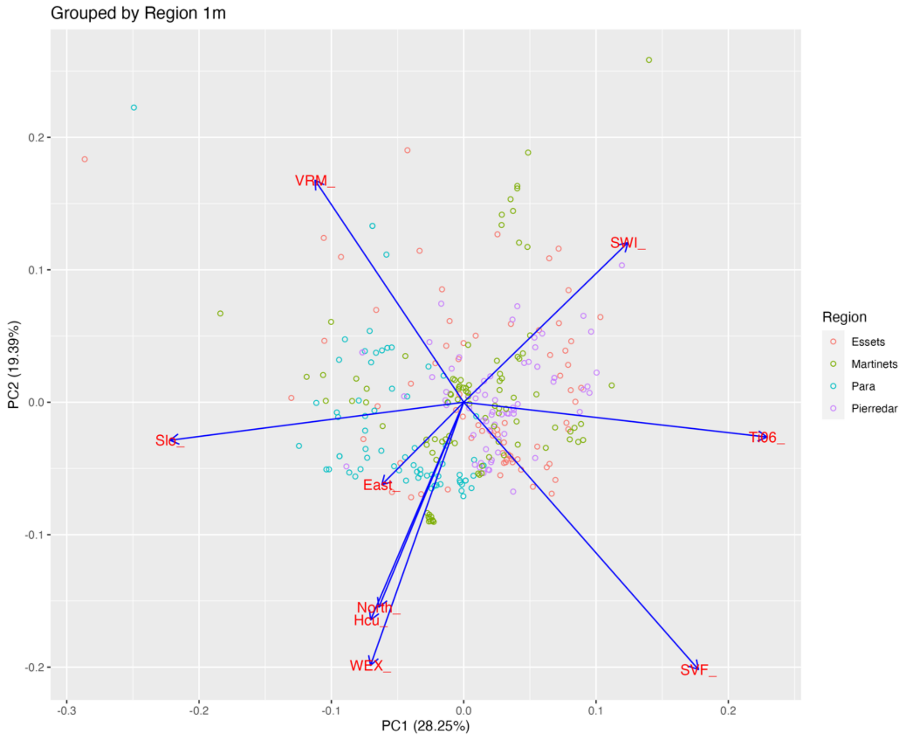

c) All variables at 2m spatial resolution

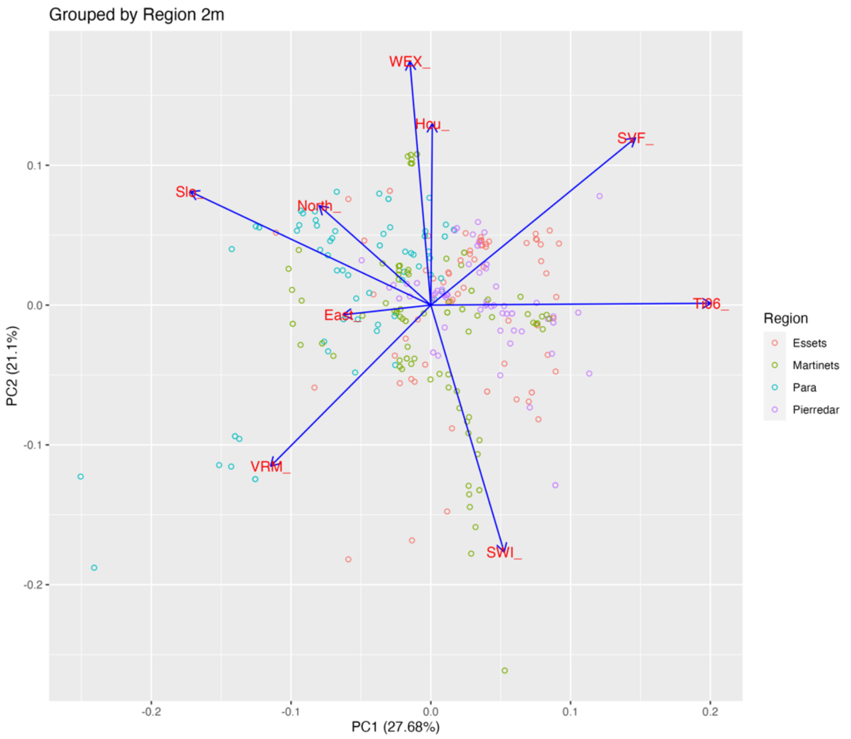

d) All variables at 4m spatial resolution

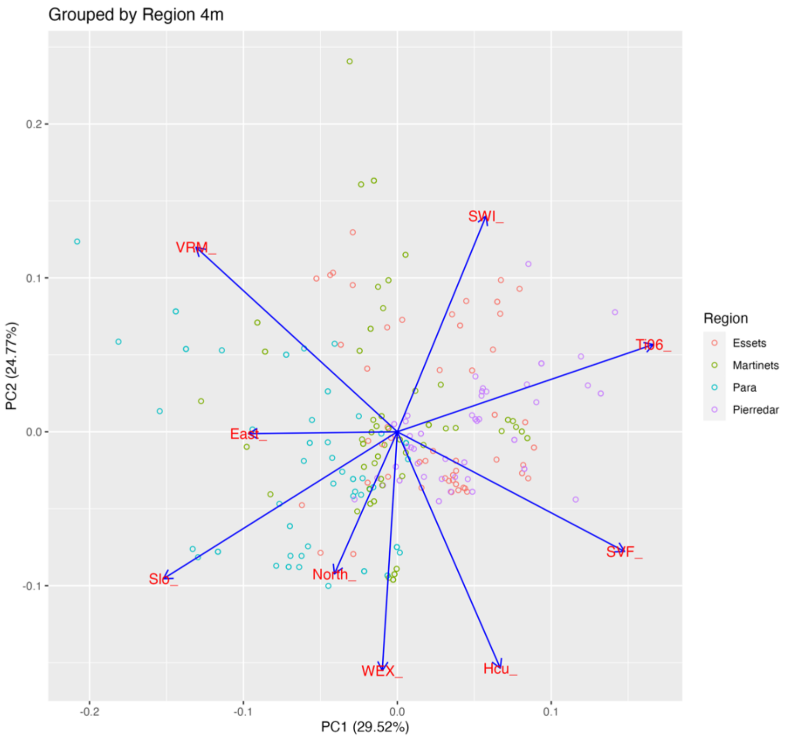

e) All variables at 8m spatial resolution

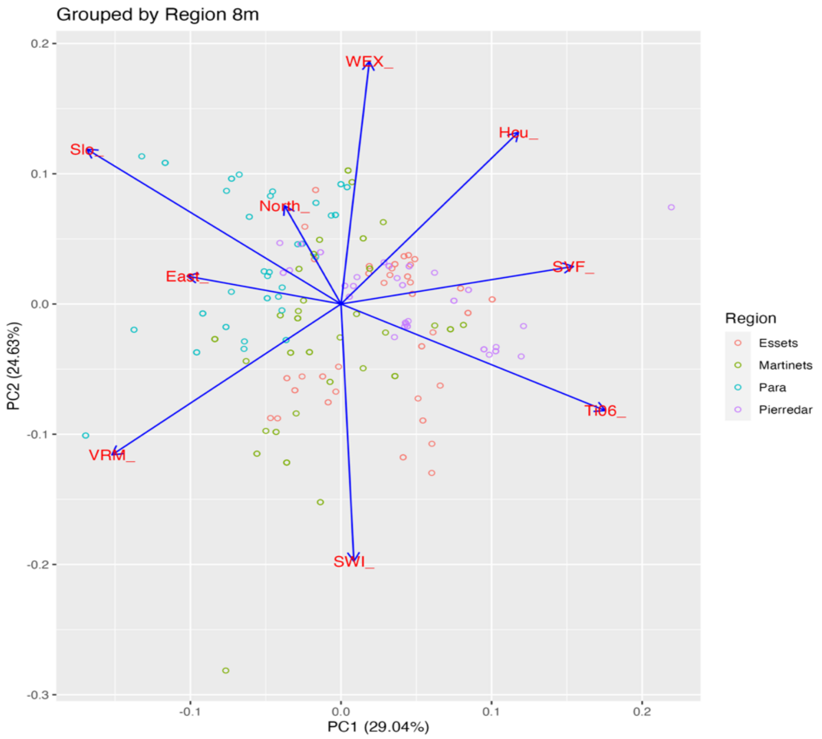

f) All variables at 16m spatial resolution

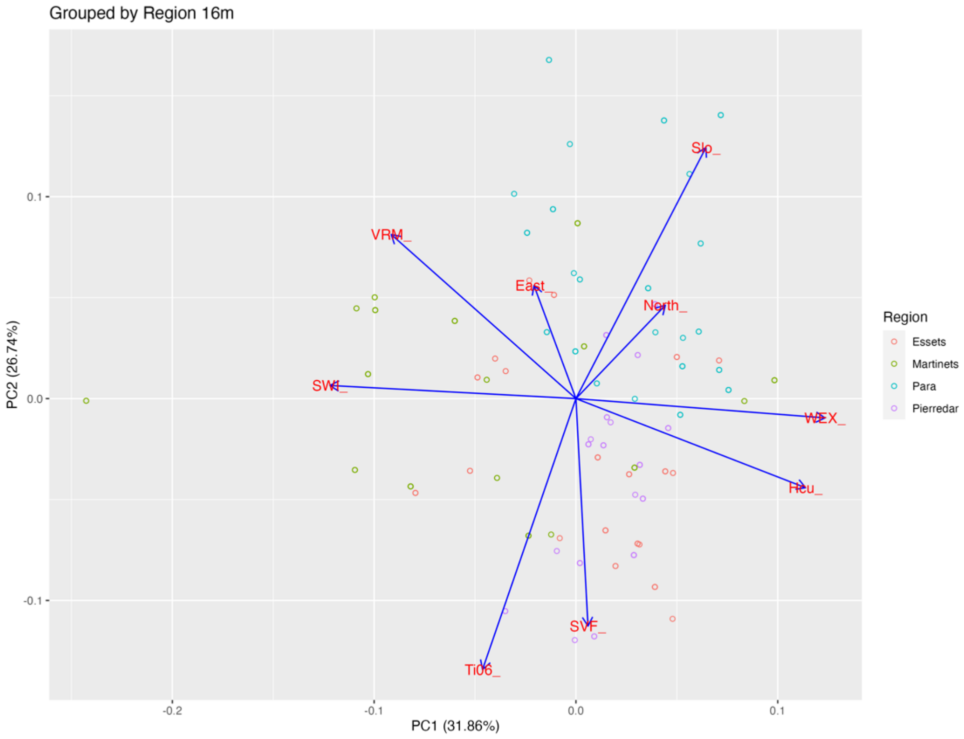

Supp Figure S5: Variation partitioning

The proportion of genetic variance at intragenic SNPs explained by partial redundancy analysis (pRDA) models, partitioned into the proportion explained by neutral processes due to demographic history (population) and spatial geographic structure (geography), and the proportion explained by the environment (elevation, environment) at the four sites (ESS, MAR, PAR, PIE) separately (local) and grouped (regional). The effect of the environment was assessed for each variable set using the six *VS-single* models, as well as *VS-all* and *VS-fwd* models, where elevation was evaluated separately. Raw values in **Table S5**.

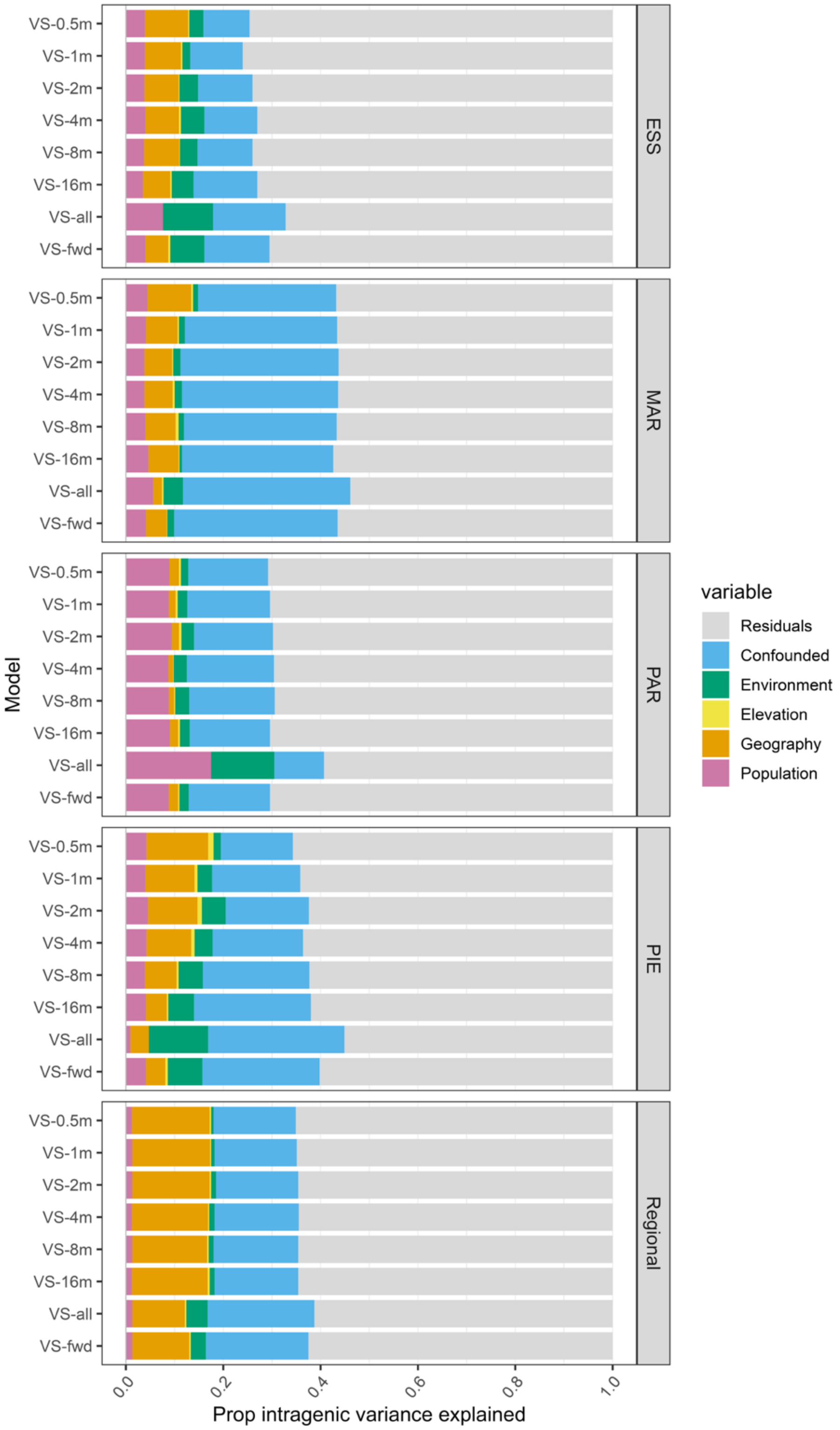

Supp Figure S6: Variance partitioning

Venn diagrams for the partitioning of variance (represented as adjusted-R^2) of intragenic SNP data into the effects of population structure ('Pop structure'; represented as PCs obtained from PCA of LD-pruned intergenic SNPs; **Table 1; Supp Figure S7**), spatial geographic structure (represented as forward-selected Moran's eigenvector maps (MEMs); **Supp Figure S8; Supp Table S6**), local adaptation (represented by environmental variables), and elevation ('Elevation' at 0.5m spatial resolution), at the four sites (ESS, MAR, PAR, PIE) separately (local, **a-d**) and grouped (regional, **e**). The effect of the environment was assessed for each variable set: i-vi) *VS-single* models, vii) *VS-all* models, and viii) *VS-fwd* models. Analyses were performed with the *varpart* function of the vegan (v2.5-7) R package. Values are summarised in **Supp Table S5**.

S6 a) Essets:
n(PC)=2; n(MEM)= 11

i) *VS-0.5m*

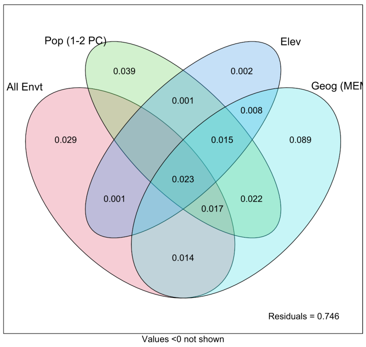

ii) *VS-1m*

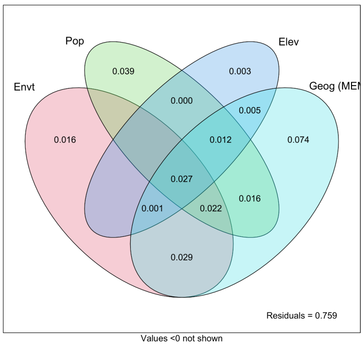

iii) *VS-2m*

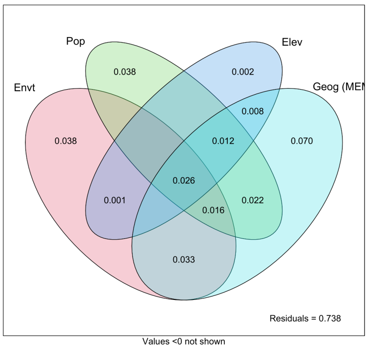

iv) *VS-4m*

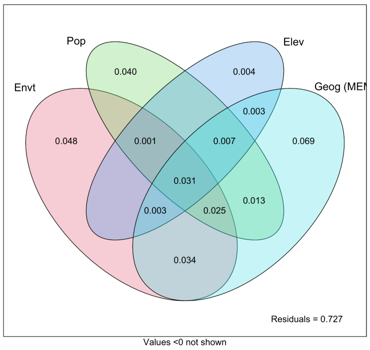

v) *VS-8m*

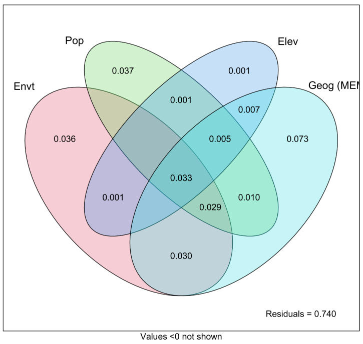

vi) *VS-16m*

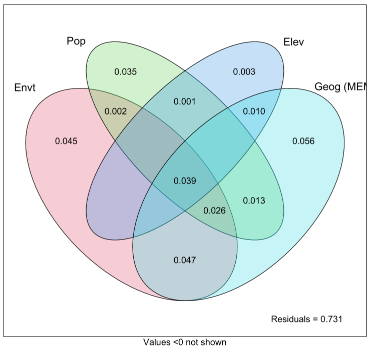

vii) *VS-all*

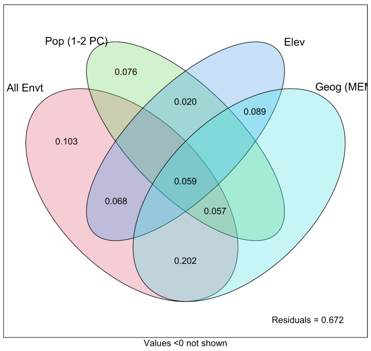

viii) *VS-fwd*

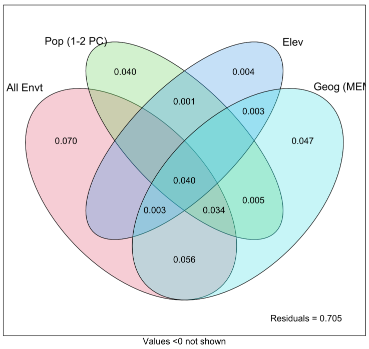

S6 b) Martinets:
n(PC)=3; n(MEM)= 16

i) VS-0.5m

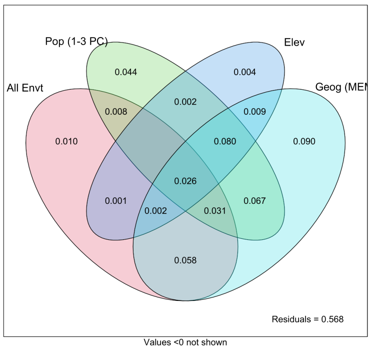

ii) VS-1m

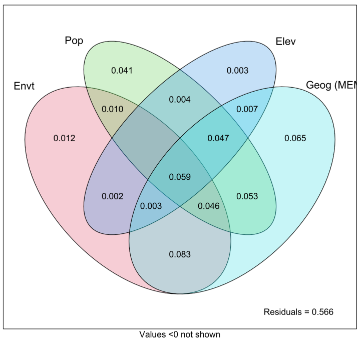

iii) VS-2m

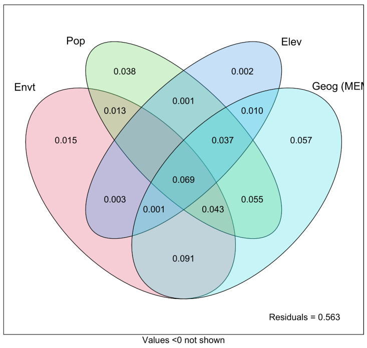

iv) VS-4m

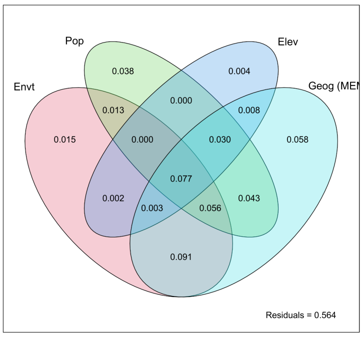

v) VS-8m

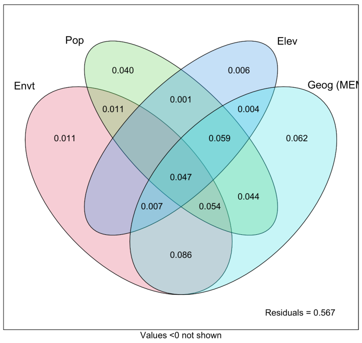

vi) VS-16m

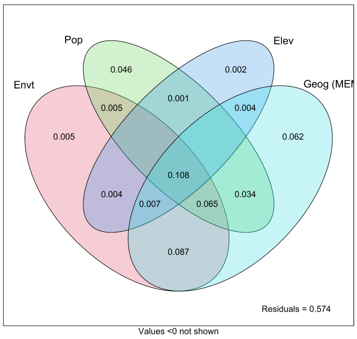

vii) VS-all

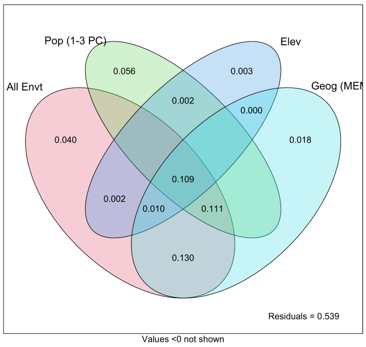

viii) VS-fwd

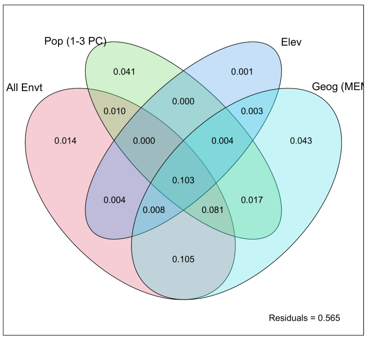

S6 c) Para:

n(PC)=5; n(MEM)= 6

i) *VS-0.5m*

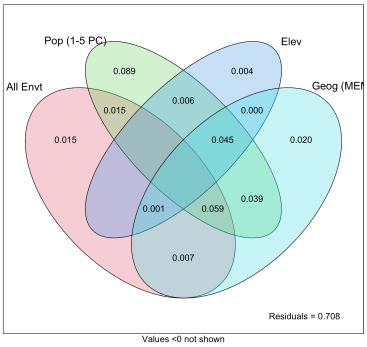

ii) *VS-1m*

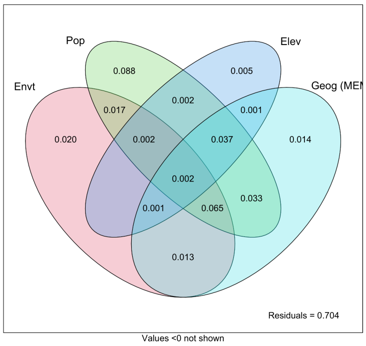

iii) *VS-2m*

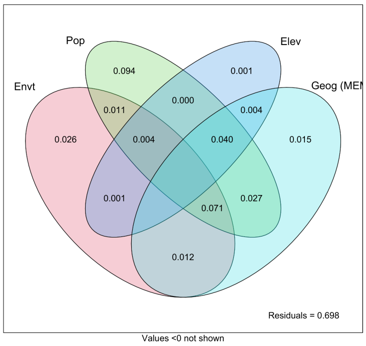

iv) *VS-4m*

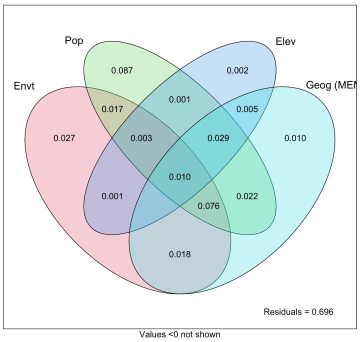

v) *VS-8m*

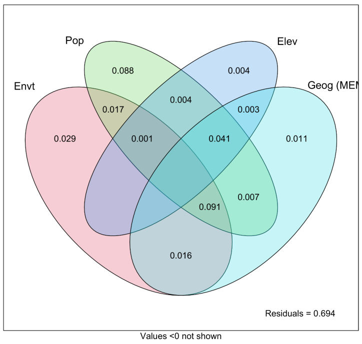

vi) *VS-16m*

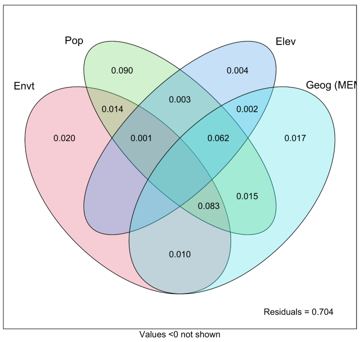

vii) *VS-all*

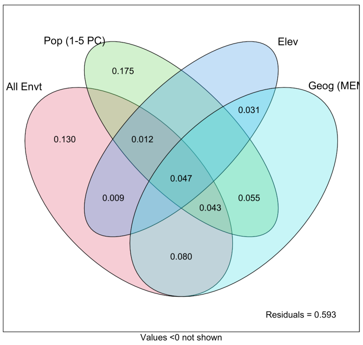

viii) *VS-fwd*

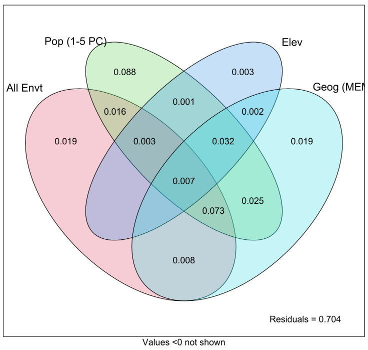

S6 d) Pierredar:

n(PC)=1; n(MEM)= 8

i) *VS-0.5m*

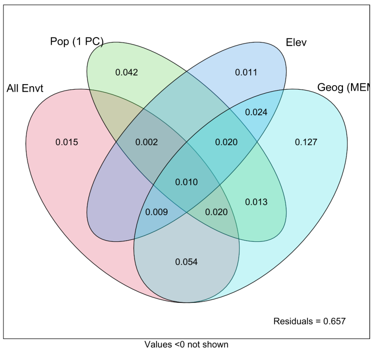

ii) *VS-1m*

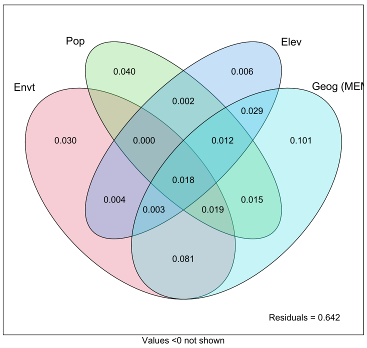

iii) *VS-2m*

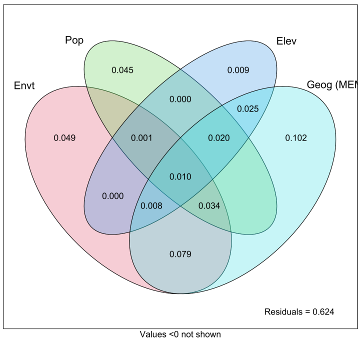

iv) *VS-4m*

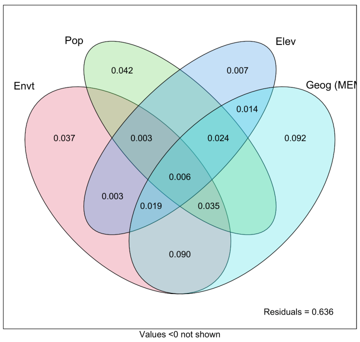

v) *VS-8m*

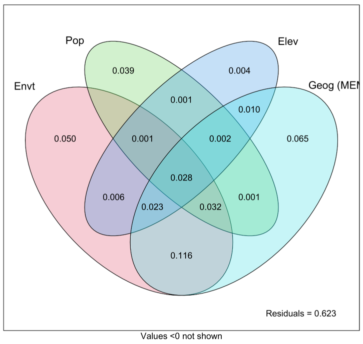

vi) *VS-16m*

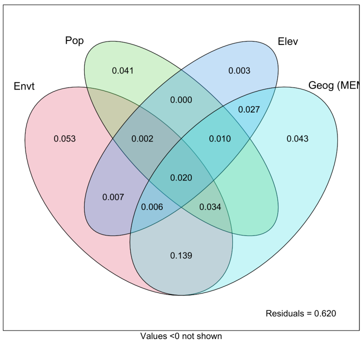

vii) *VS-all*

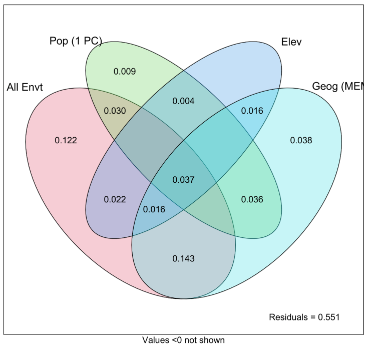

viii) *VS-fwd*

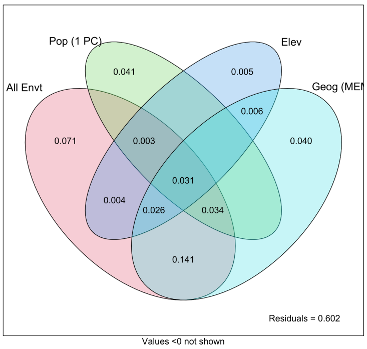

S6 e) Regional:

n(PC)=3; n(MEM)= 40

i) *VS-0.5m*

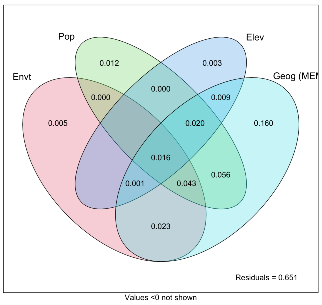

ii) *VS-1m*

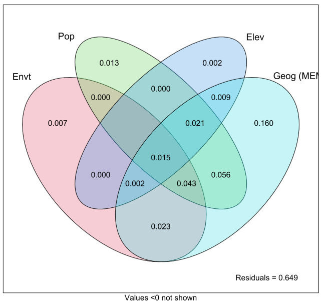

iii) *VS-2m*

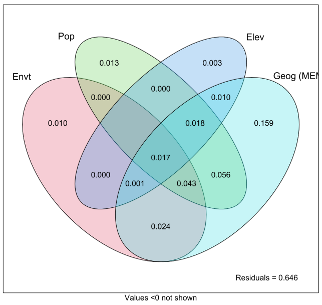

iv) *VS-4m*

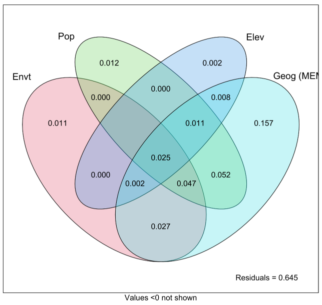

v) *VS-8m*

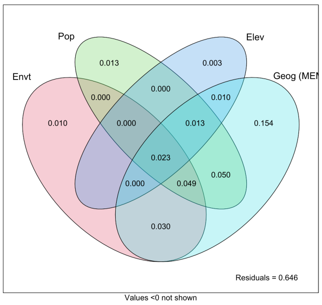

vi) *VS-16m*

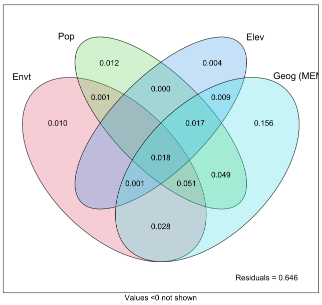

vii) *VS-all*

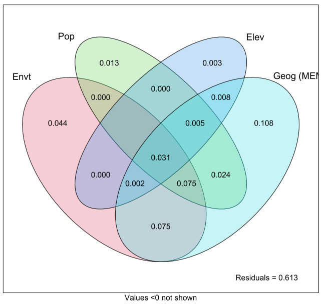

viii) *VS-fwd*

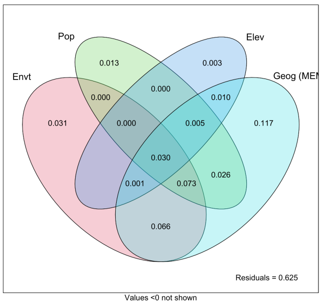

Supp Figure S7: Population structure.

Population structure for the sampled plants was assessed at the local **(a-d)** and regional (**e**) levels with principal component analyses (PCA), using the scaled neutral SNP data set (intergenic LD-pruned SNPs; **Table 1**). Scree plots with broken stick algorithms are provided (i), along with the biplots for the number of PCs retained (ii). Maps (iii) indicate the spatial distribution of neutral genetic structure, showing the eigenvector (EV) values attributed to each sampled individual for the number of PC retained at each site. At Martinets and the regional levels, the scree plot and broken stick algorithms indicated clear population structure that could be explained with the first 3 PCs at each. At Essets, Para and Pierredar, the scree plots showed no clear population structure, so the number of PCs retained was based on kinks in the scree plots. The PCs were used to represent population structure in the variance partitioning models. Analyses were performed with the *vegan* R package (v2.5-7).

S7 a) Essets: PC1-2 cumulatively explained 10% of the genetic variance.

i)

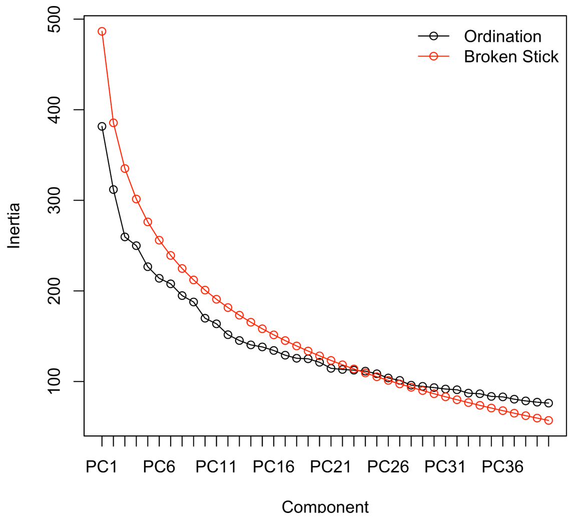

ii)

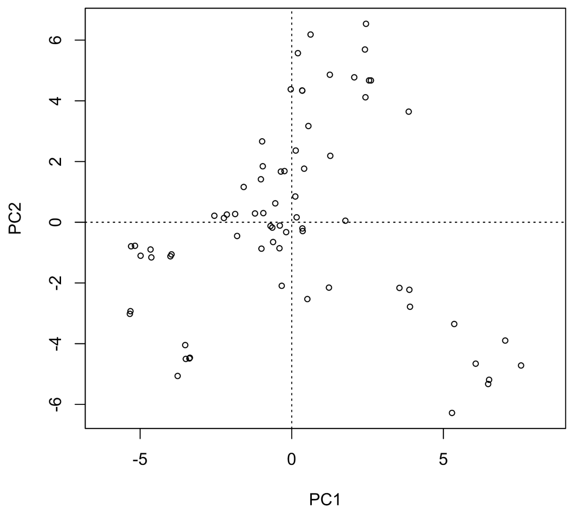

iii)

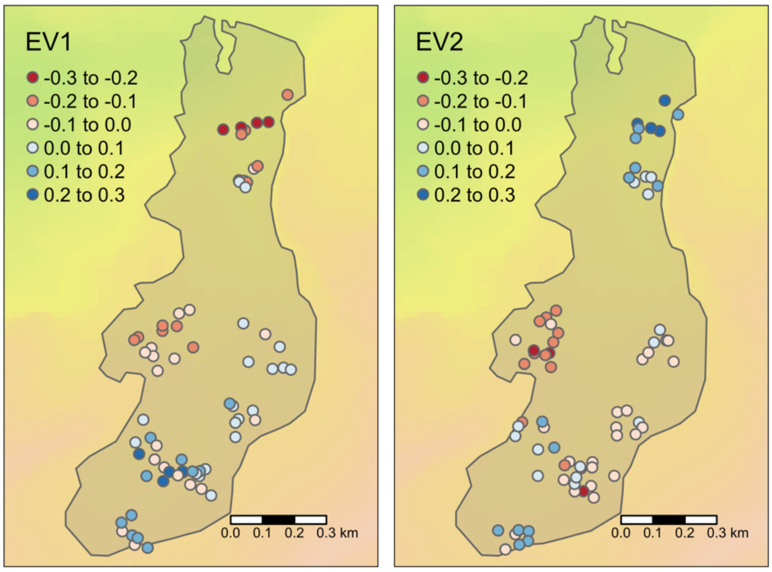

S7 b) Martinets: PC1-3 cumulatively explained 15% of the genetic variance.

i)

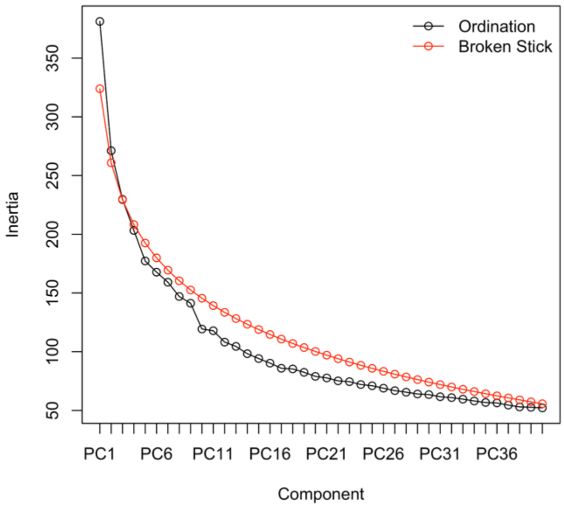

ii)

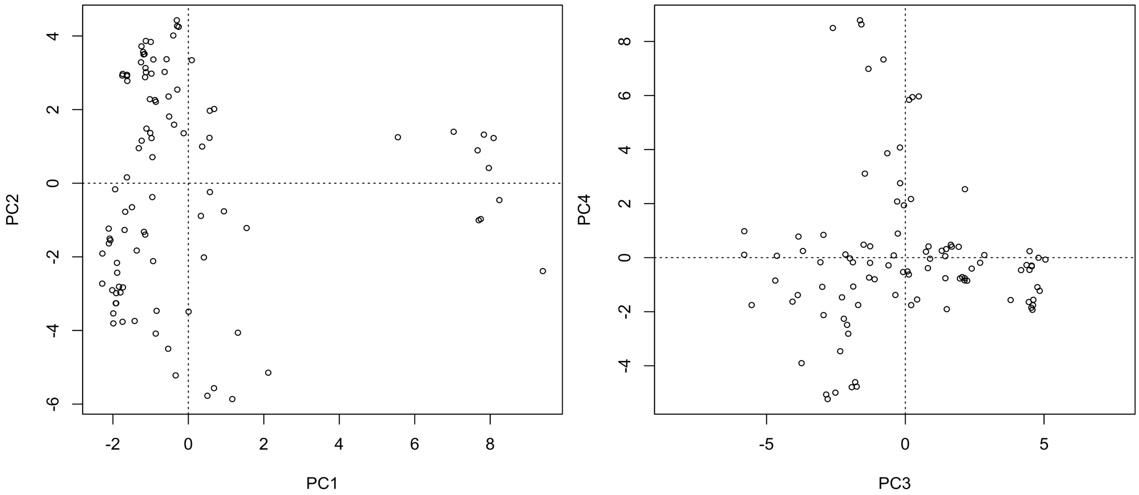

iii)

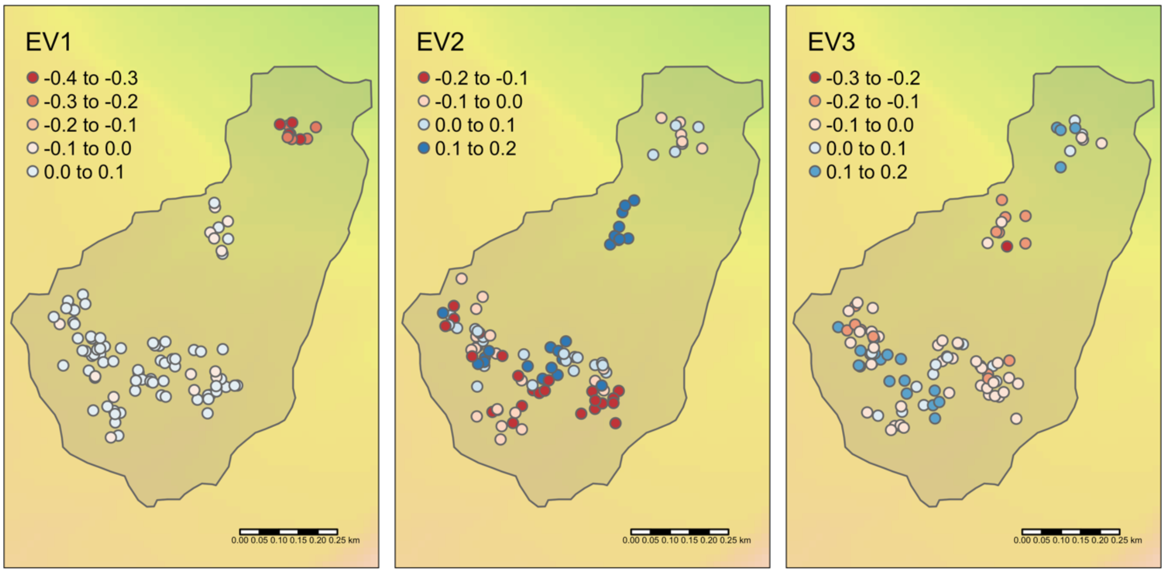

S7 c) Para: PC1-5 cumulatively explained 19% of the genetic variance.

i) **ii)**

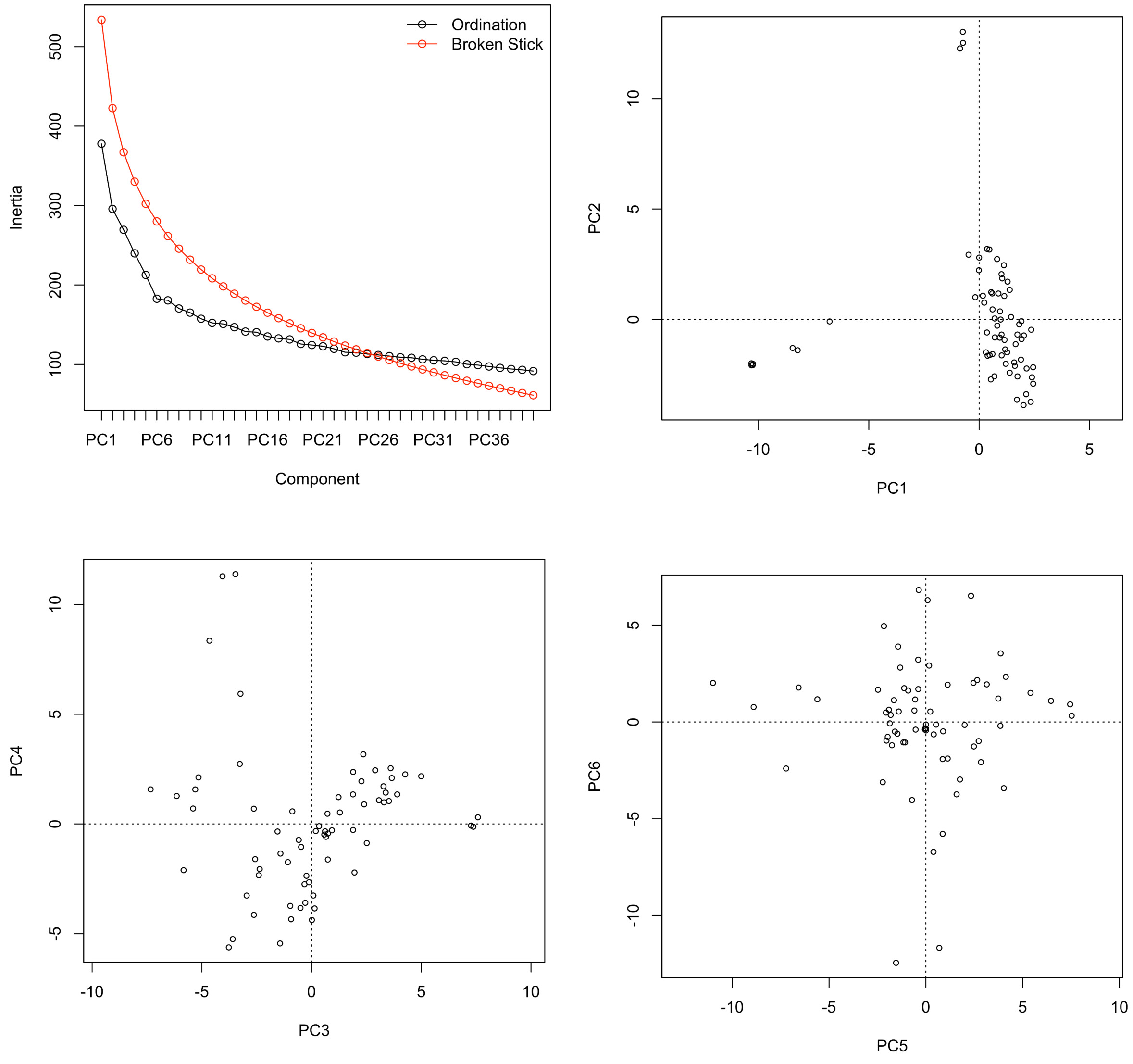

iii)

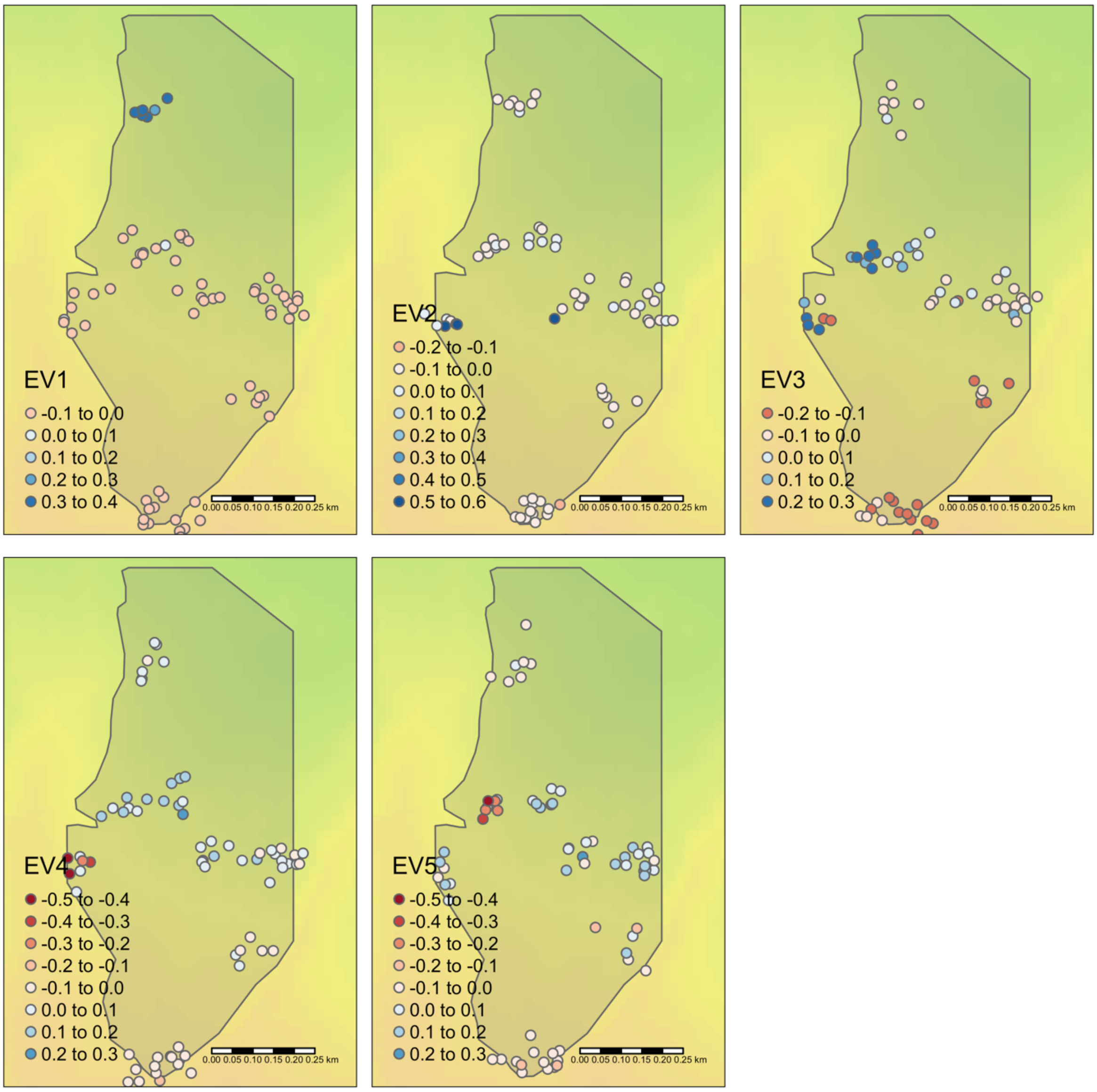
EV1
-0.1 to 0.0
0.0 to 0.1
0.1 to 0.2
0.2 to 0.3
0.3 to 0.4
0.00 0.05 0.10 0.15 0.20 0.25 km
EV2
-0.2 to -0.1
-0.1 to 0.0
0.0 to 0.1
0.1 to 0.2
0.2 to 0.3
0.3 to 0.4
0.4 to 0.5
0.5 to 0.6
0.00 0.05 0.10 0.15 0.20 0.25 km
EV3
-0.2 to -0.1
-0.1 to 0.0
0.0 to 0.1
0.1 to 0.2
0.2 to 0.3
0.00 0.05 0.10 0.15 0.20 0.25 km
EV4
-0.5 to -0.4
-0.4 to -0.3
-0.3 to -0.2
-0.2 to -0.1
-0.1 to 0.0
0.0 to 0.1
0.1 to 0.2
0.2 to 0.3
0.00 0.05 0.10 0.15 0.20 0.25 km
EV5
-0.5 to -0.4
-0.4 to -0.3
-0.3 to -0.2
-0.2 to -0.1
-0.1 to 0.0
0.0 to 0.1
0.1 to 0.2
0.2 to 0.3
0.00 0.05 0.10 0.15 0.20 0.25 km

S7 d) Pierredar: PC1 explained 7% of the genetic variance.

i)

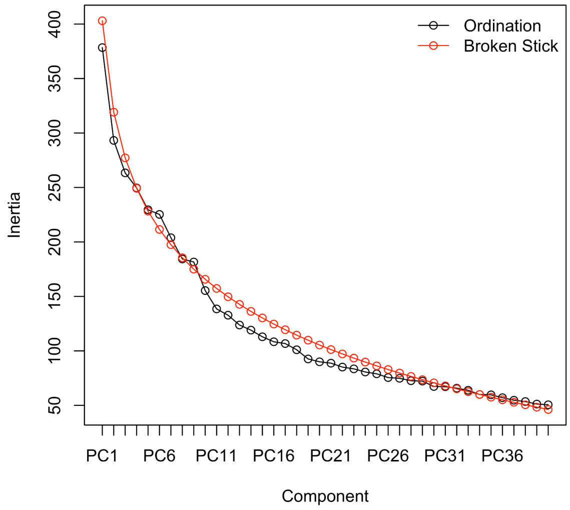

ii)

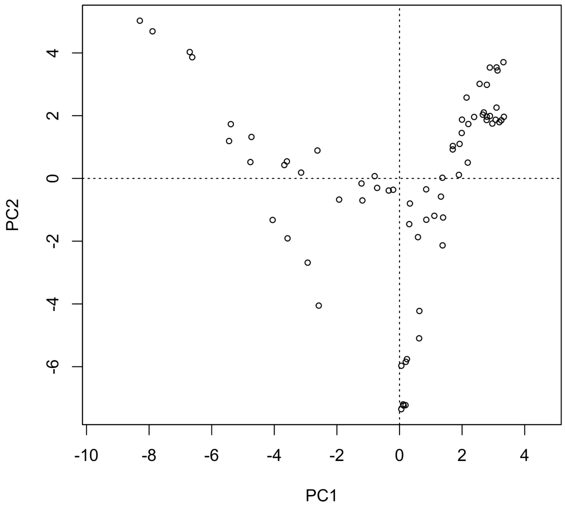

iii)

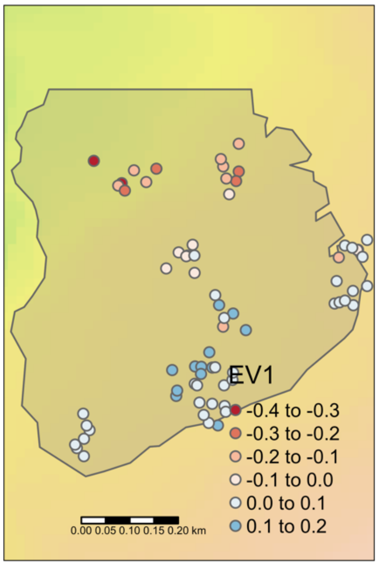

S7 e) Regional: PC1-3 cumulatively explained 12% of the genetic variance

i)

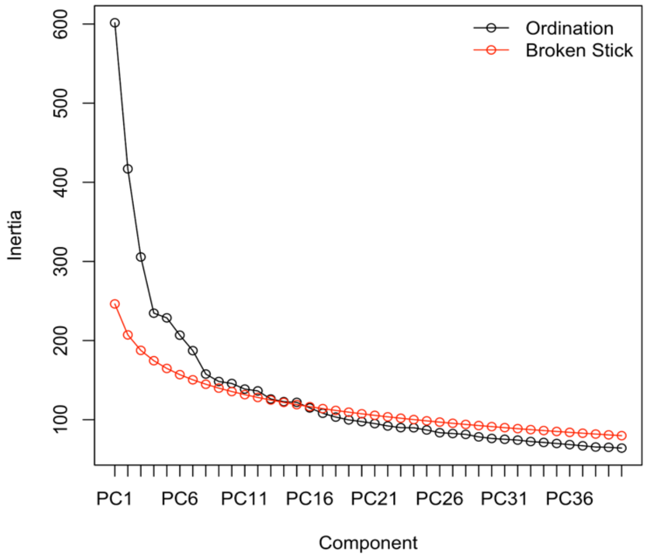

ii)

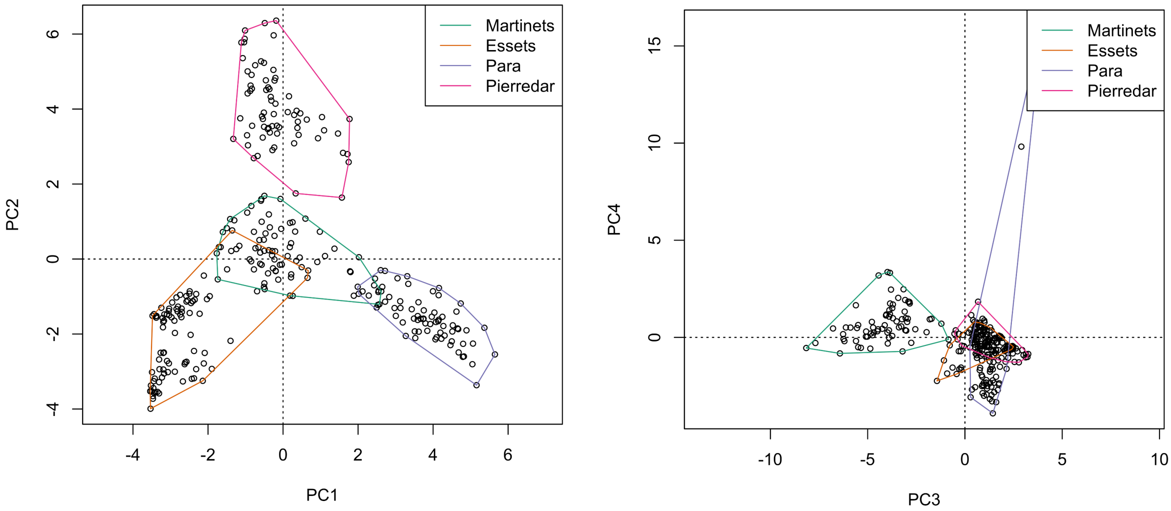

iii)

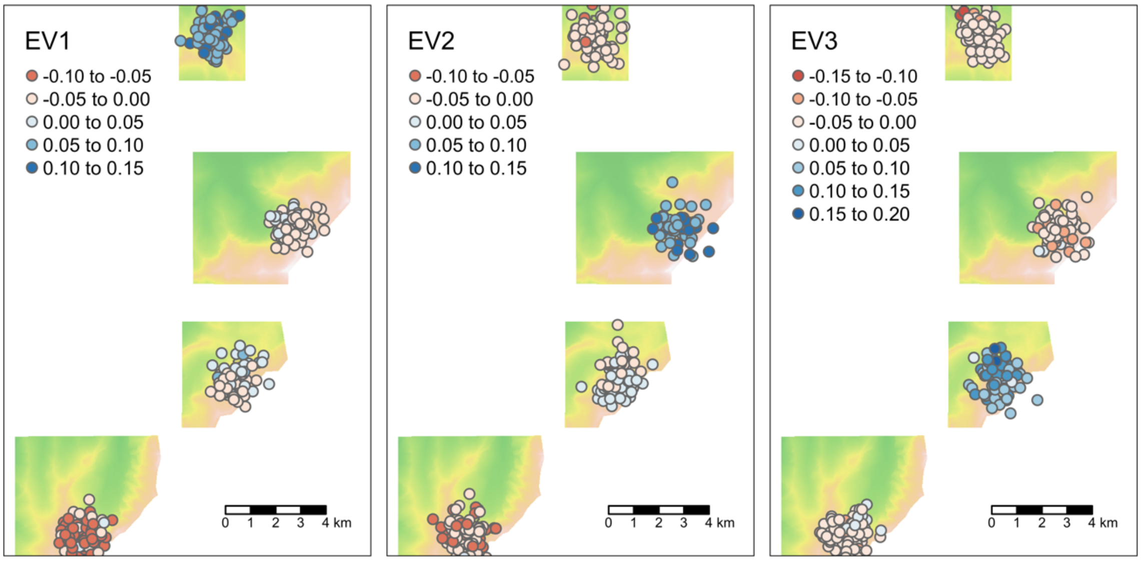

Supp Figure S8: Geography and MEMs.

Nearest neighbour networks triangulated from plant individual geographic coordinates (X, Y) used to calculate Moran's Eigenvector Maps (MEMs) to assess spatial connectivity among individuals. Connectivity maps are shown at the local study site level (**a-d**) and at the regional level (**e**). Order of selection for forward selected MEMs used in the variation partitioning of the intragenic SNP data set can be found in **Supp Table S6**.

S8 a) Essets

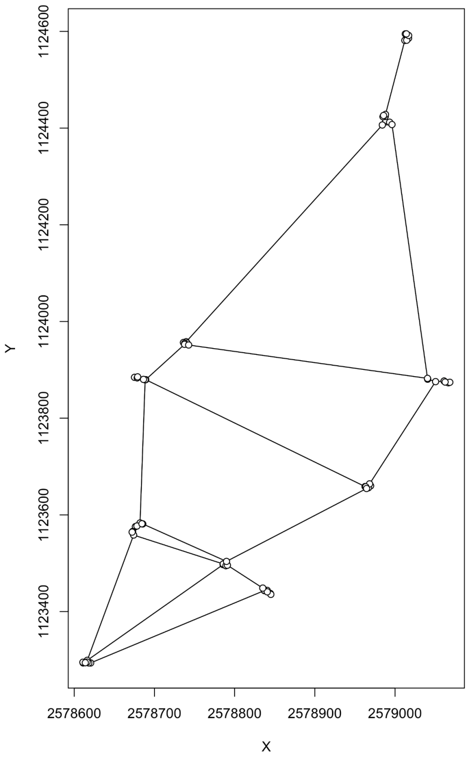

S8 b) Martinets

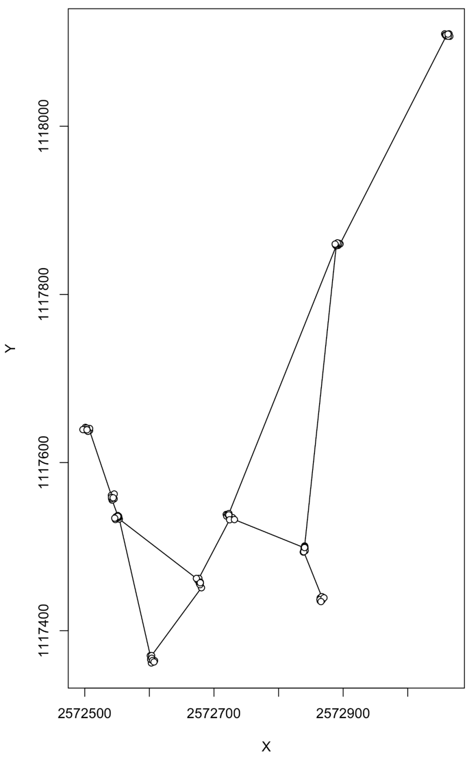

S8 c) Para

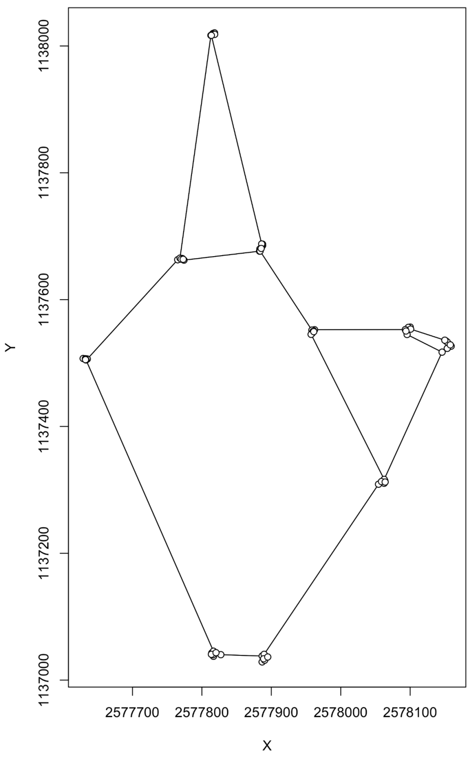

S8 d) Pierredar

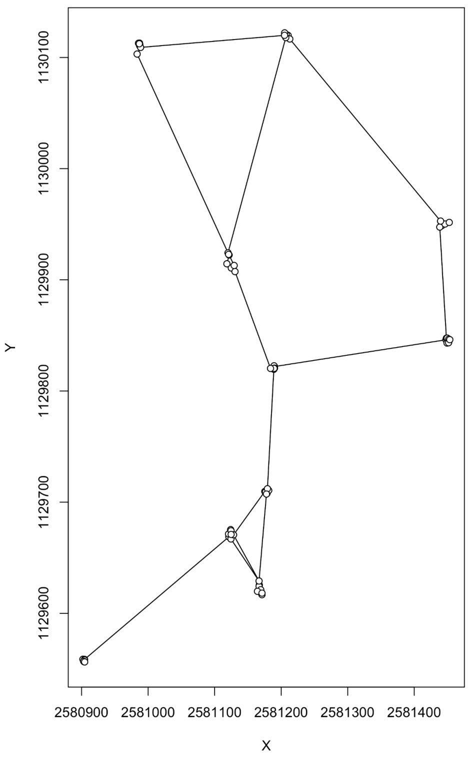

S8 e) Regional

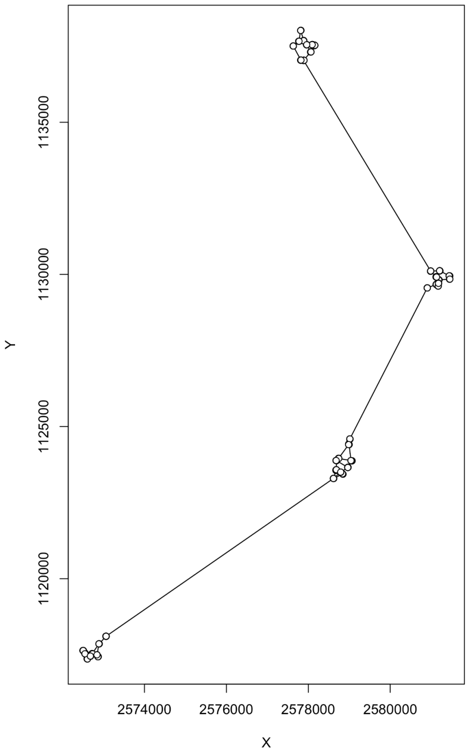

Supp Figure S9: ***GEA model***

Scree plots of ordination axis eigenvalues for each redundancy analysis (RDA) model, used to determine the number of constrained axes to retain for calculating outlier loci. The RDA models were performed at the local **(a-d)** and regional **(e)** levels. The models were built with one of eight variable sets: i-vi) *VS-single* models with variables at a single spatial resolution (0.5, 1, 2, 4, 8, 16m), vii) *VS-all* models built with all variables at all spatial resolutions and viii) *VS-fwd* models built with variables selected using forward selection methods. All models included elevation at 0.5m spatial resolution. Analyses were performed with the *rda* function of the vegan (v2.5-7) R package.

S9 a) Essets:
K=3 retained RDA axes

i) *VS-0.5m*

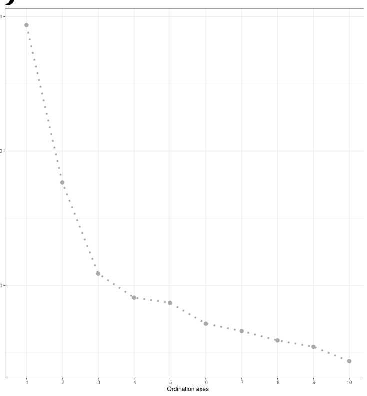

ii) *VS-1m*

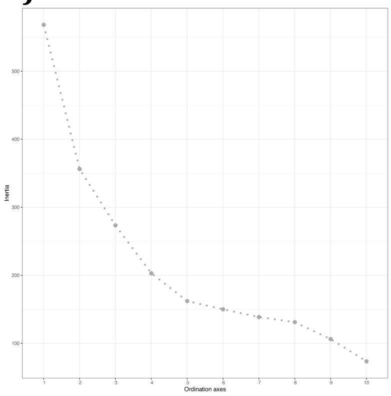

iii) *VS-2m*

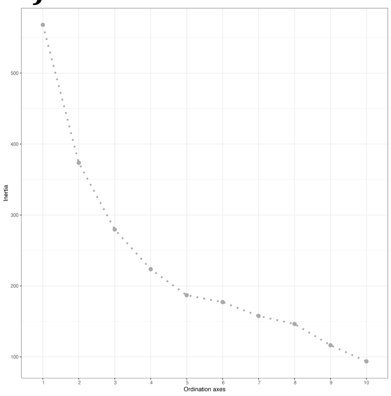

iv) *VS-4m*

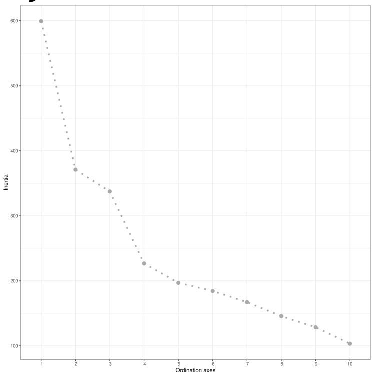

v) *VS-8m*

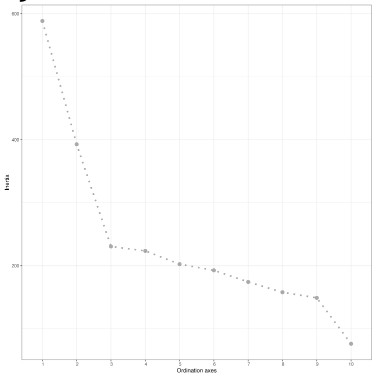

vi) *VS-16m*

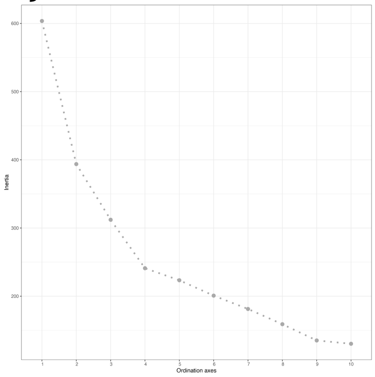

vii) *VS-all*

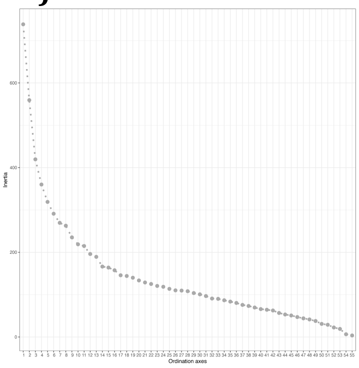

viii) *VS-fwd*

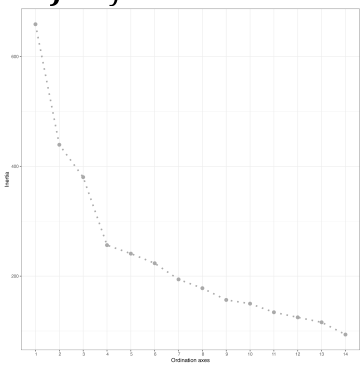

S9 b) Martinets
K=2 retained RDA axes

i) *VS-0.5m*

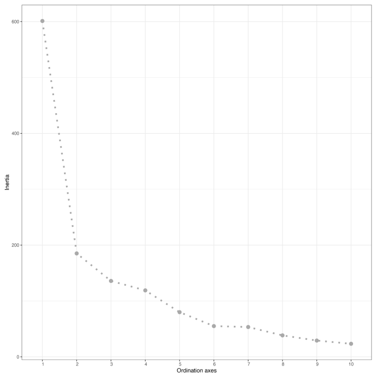

ii) *VS-1m*

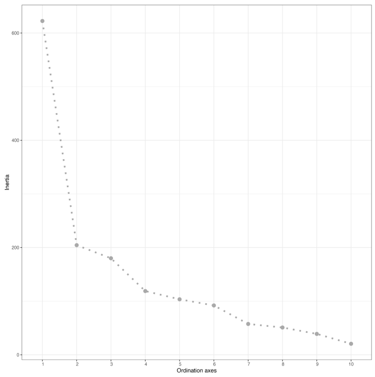

iii) *VS-2m*

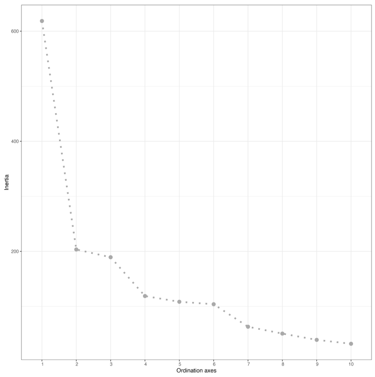

iv) *VS-4m*

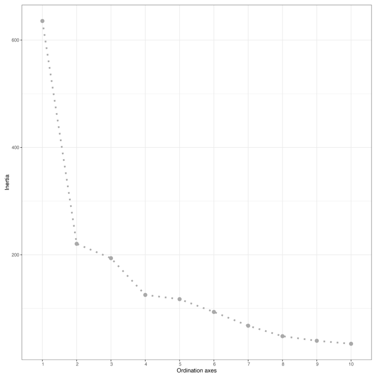

v) *VS-8m*

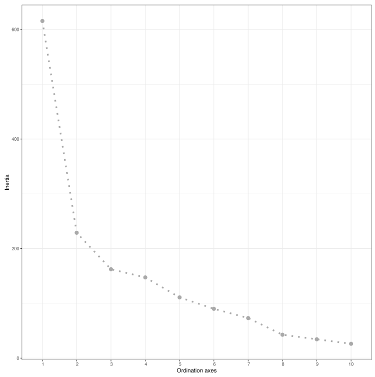

vi) *VS-16m*

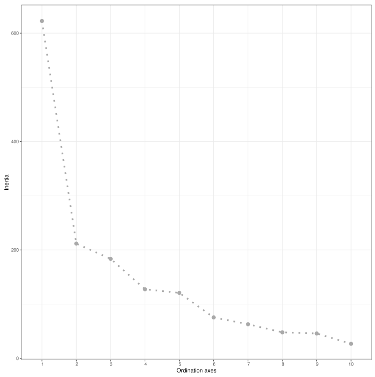

vii) *VS-all*

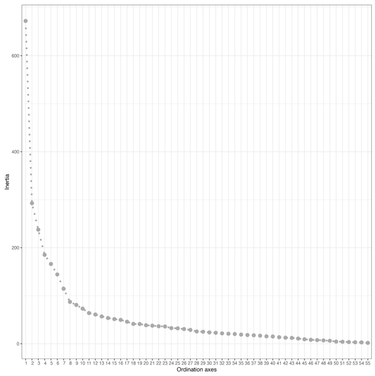

viii) *VS-fwd*

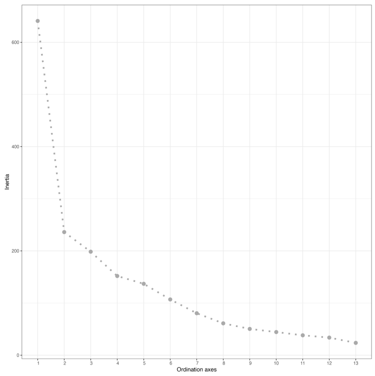

S9 c) Para:
K=2 retained RDA axes

i) *VS-0.5m*

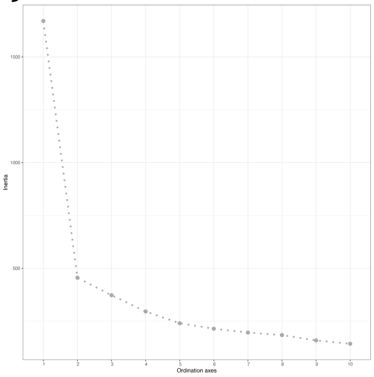

ii) *VS-1m*

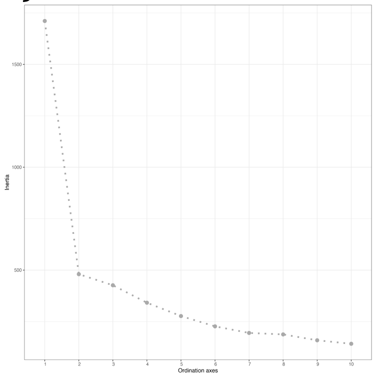

iii) *VS-2m*

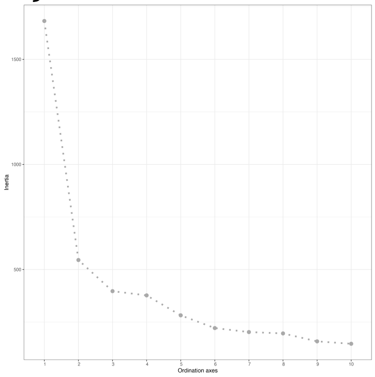

iv) *VS-4m*

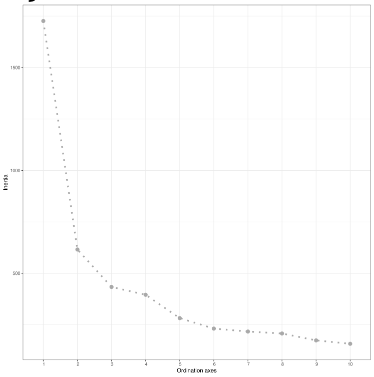

v) *VS-8m*

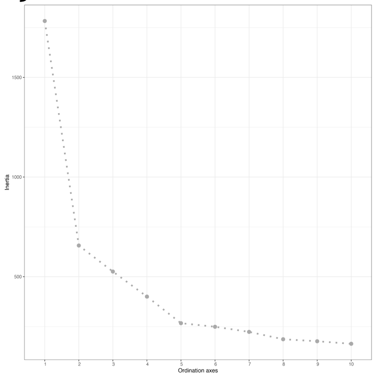

vi) *VS-16m*

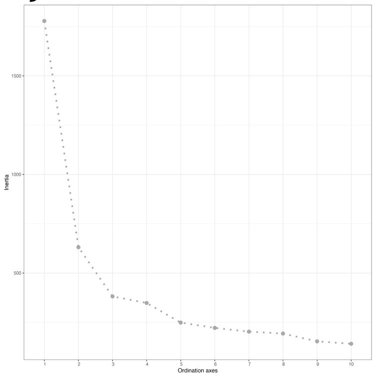

vii) *VS-all*

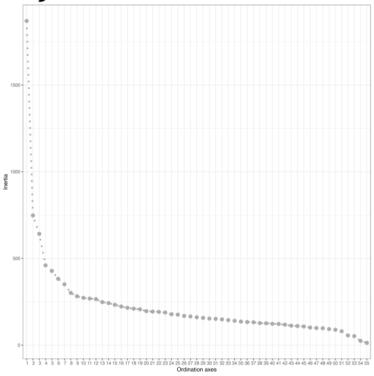

viii) *VS-fwd*

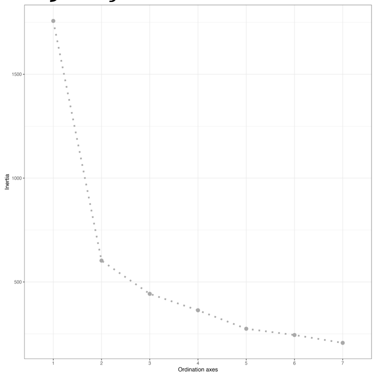

S9 d) Pierredar:
K=3 retained RDA axes

i) *VS-0.5m*

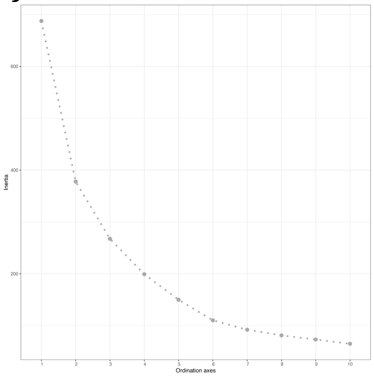

ii) *VS-1m*

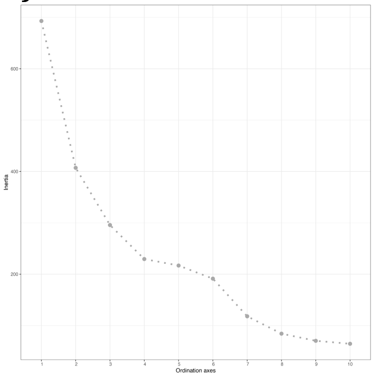

iii) *VS-2m*

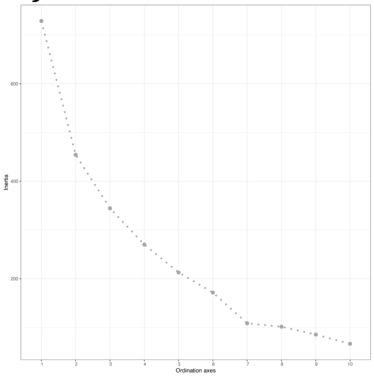

iv) *VS-4m*

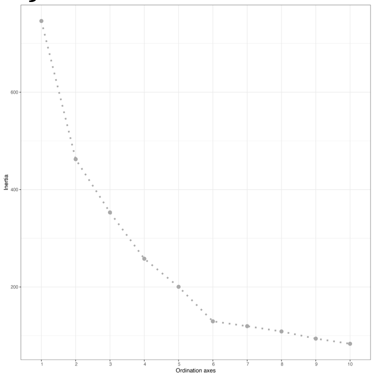

v) *VS-8m*

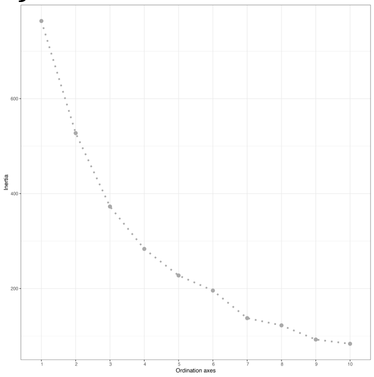

vi) *VS-16m*

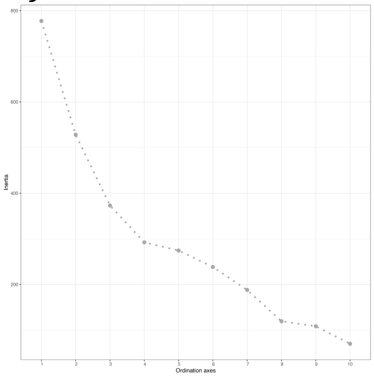

vii) *VS-all*

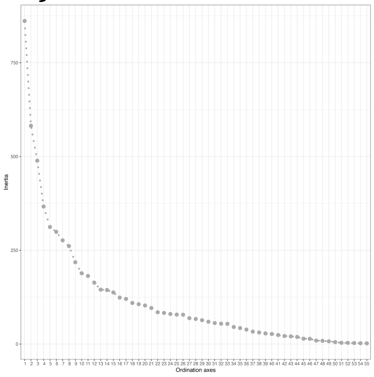

viii) *VS-fwd*

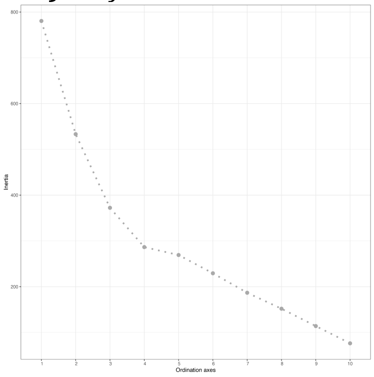

S9 e) Regional:
K=2 retained RDA axes

i) *VS-0.5m*

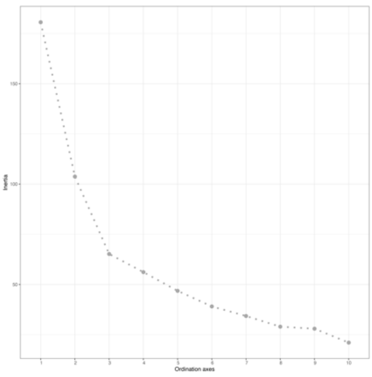

ii) *VS-1m*

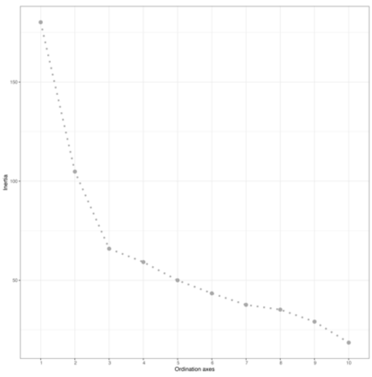

iii) *VS-2m*

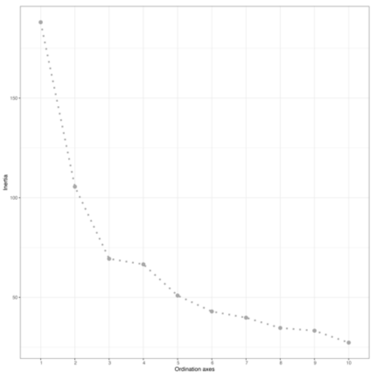

iv) *VS-4m*

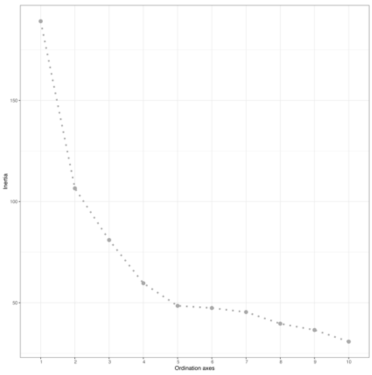

v) *VS-8m*

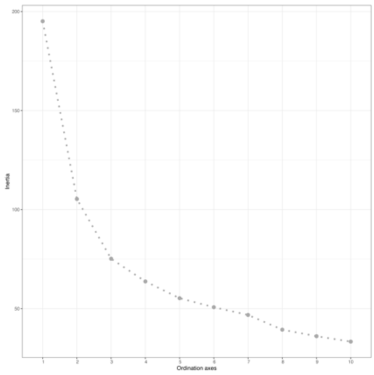

vi) *VS-16m*

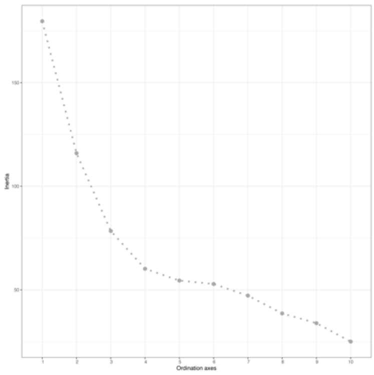

vii) *VS-all*

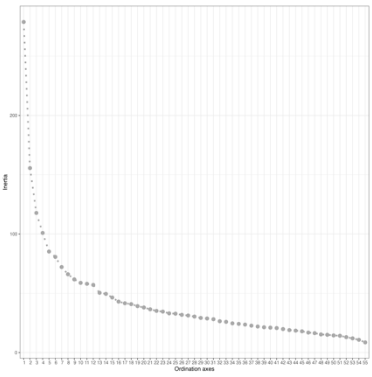

viii) *VS-fwd*

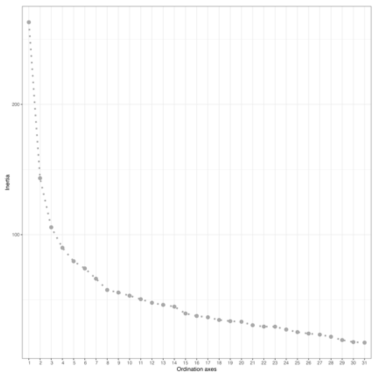

Supp Figure S10: ***GEA model.***

Histograms of SNP p-values assigned based on extremeness along a Mahalanobis distance, which was estimated between the distance of the SNP from the center of the redundancy analysis (RDA) space along retained axes. The RDA models were performed at the local **(a-d)** and regional **(e)** levels. The models were built with one of eight variable sets: **i-vi)** *VS-single* models with variables at a single spatial resolution (0.5, 1, 2, 4, 8, 16m), **vii)** *VS-all* models built with all variables at all spatial resolutions and **viii)** *VS-fwd* models built with variables selected using forward selection methods. All models included elevation at 0.5m spatial resolution. Analyses were performed with *rda* function of the vegan (v2.5-7) R package.

S10 a) Essets: *K=3 retained RDA axes*

i) *VS-0.5m*

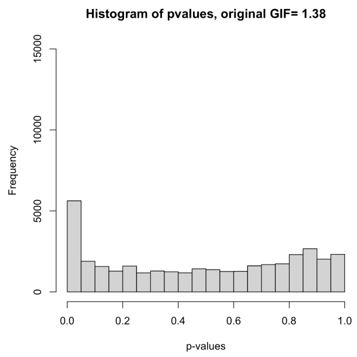

ii) *VS-1m*

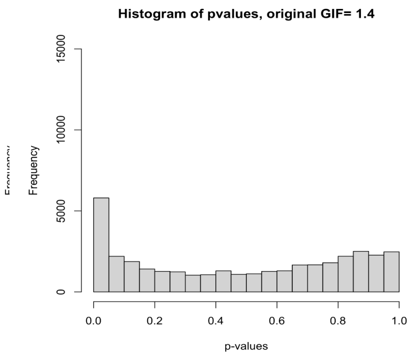

iii) *VS-2m*

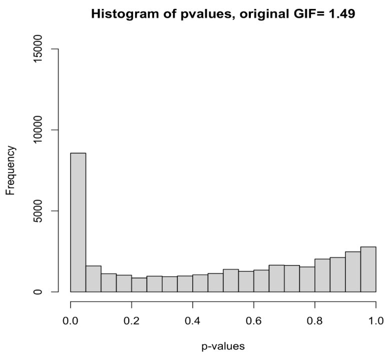

iv) *VS-4m*

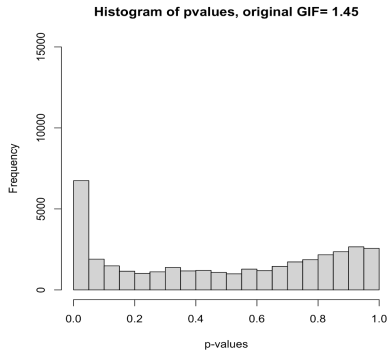

v) *VS-8m*

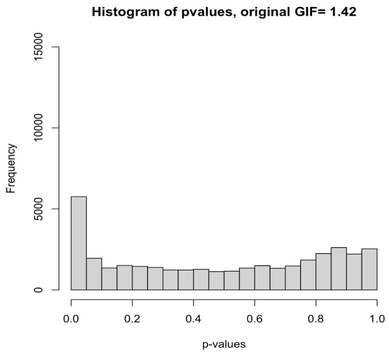

vi) *VS-16m*

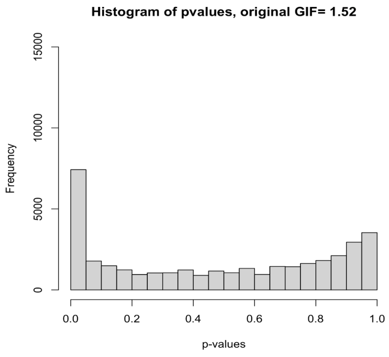

vii) *VS-all*

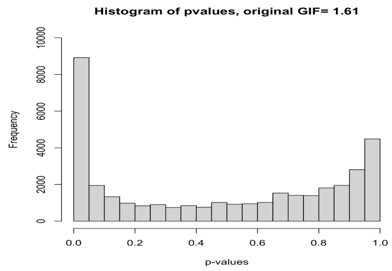

viii) *VS-fwd*

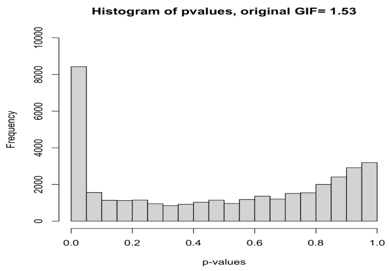

S10 b) Martinets:
K=2 retained RDA axes

i) *VS-0.5m*

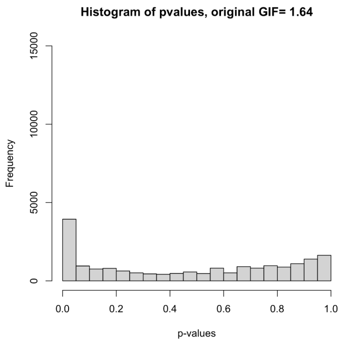

ii) *VS-1m*

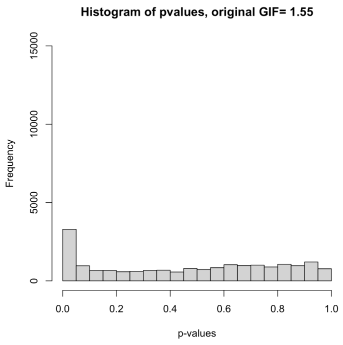

iii) *VS-2m*

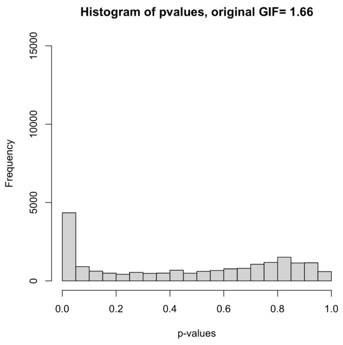

iv) *VS-4m*

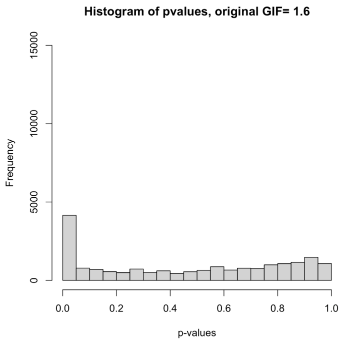

v) *VS-8m*

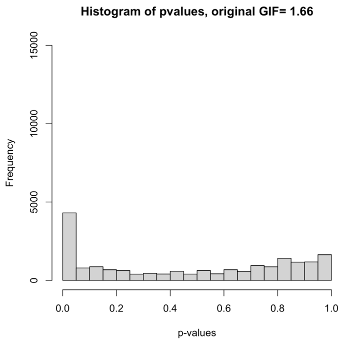

vi) *VS-16m*

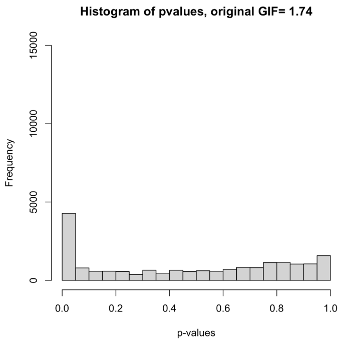

vii) *VS-all*

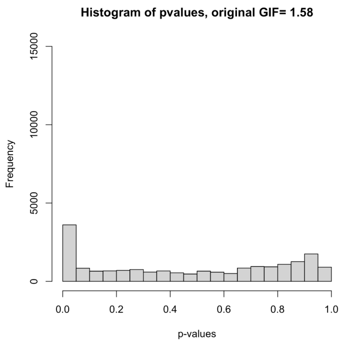

viii) *VS-fwd*

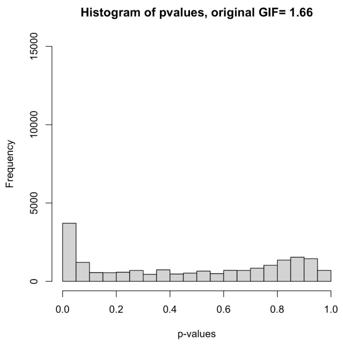

S10 c) Para:
K=2 retained RDA axes

i) *VS-0.5m*

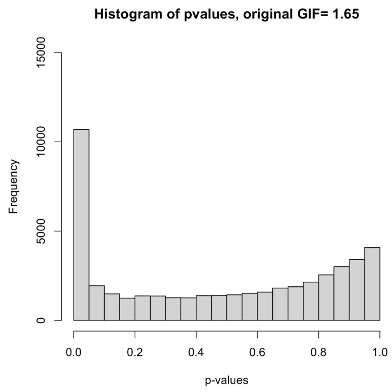

ii) *VS-1m*

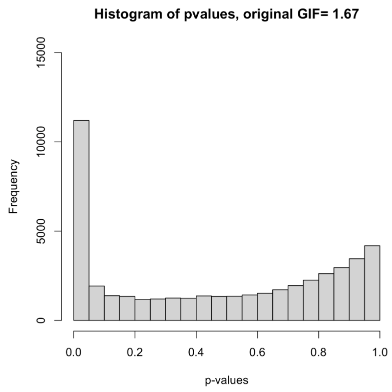

iii) *VS-2m*

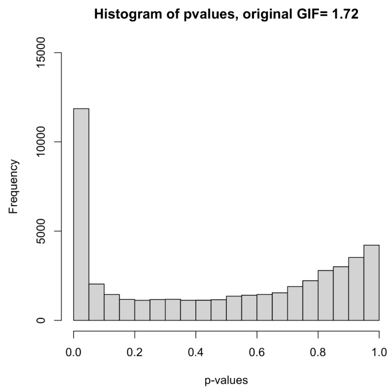

iv) *VS-4m*

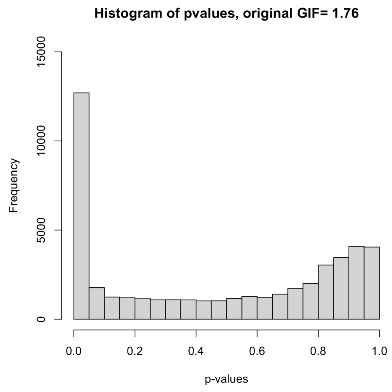

v) *VS-8m*

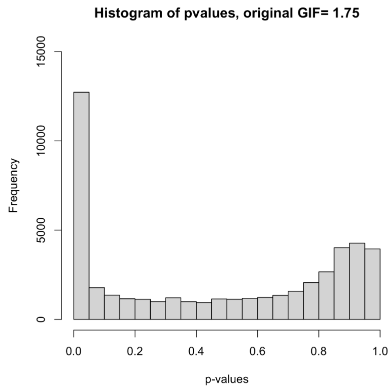

vi) *VS-16m*

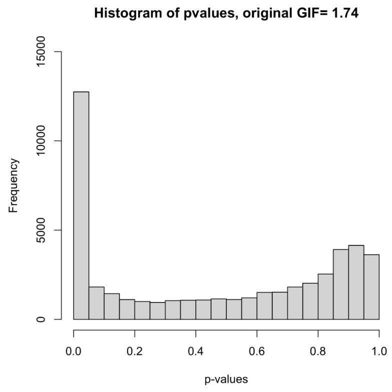

vii) *VS-all*

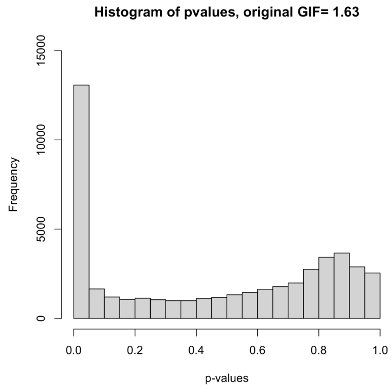

viii) *VS-fwd*

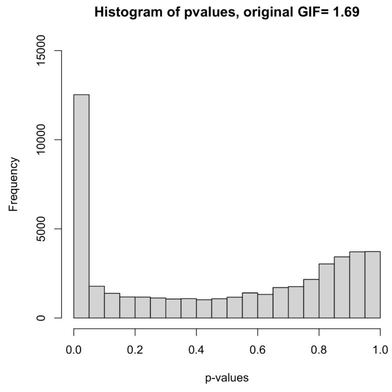

S10 d) Pierredar:
K=3 retained RDA axes

i) *VS-0.5m*

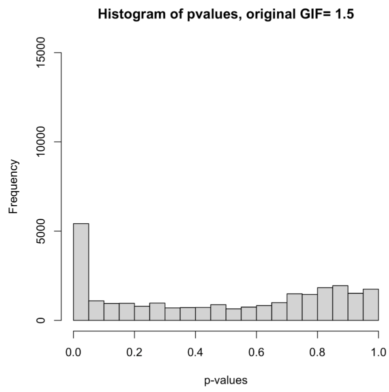

ii) *VS-1m*

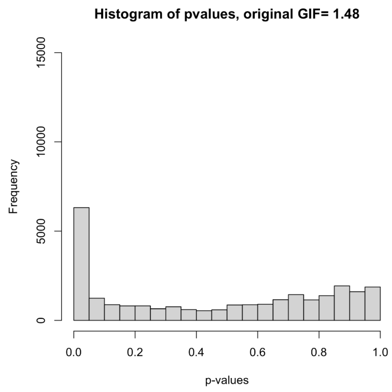

iii) *VS-2m*

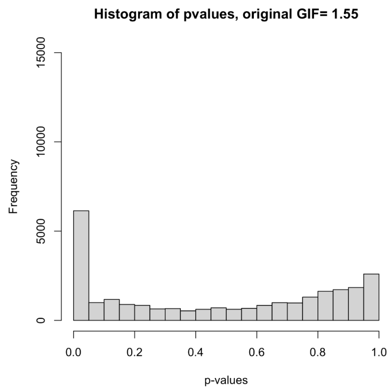

iv) *VS-4m*

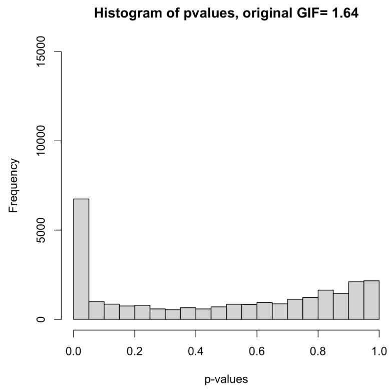

v) *VS-8m*

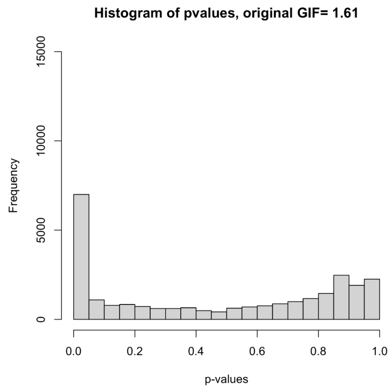

vi) *VS-16m*

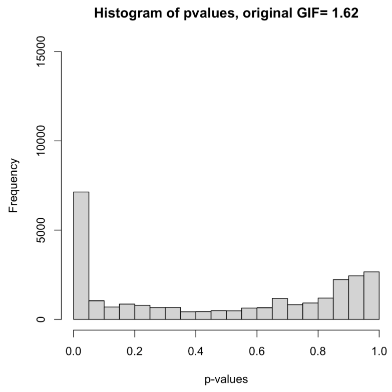

vii) *VS-all*

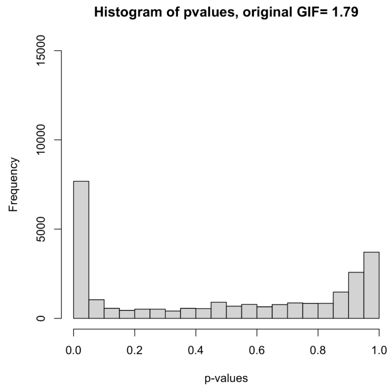

viii) *VS-fwd*

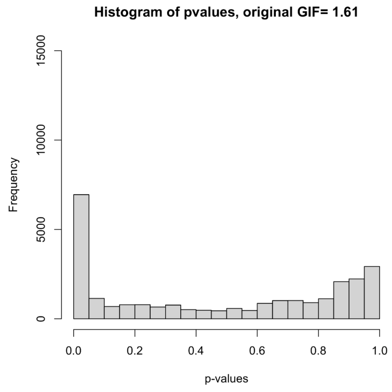

S10 e) Regional:
K=2 retained RDA axes

i) *VS-0.5m*

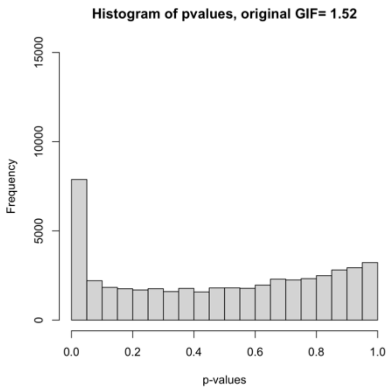

ii) *VS-1m*

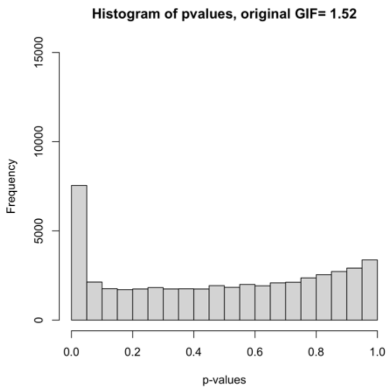

iii) *VS-2m*

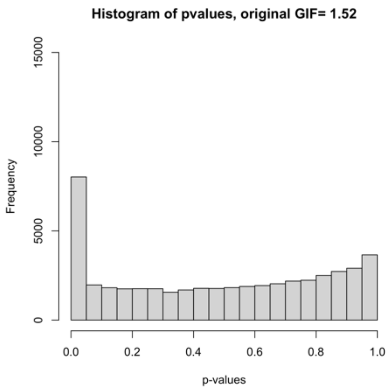

iv) *VS-4m*

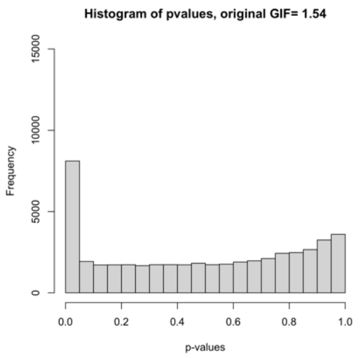

v) *VS-8m*

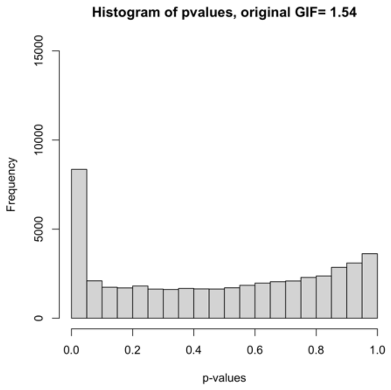

vi) *VS-16m*

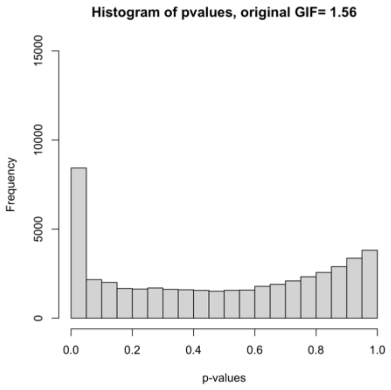

vii) *VS-all*

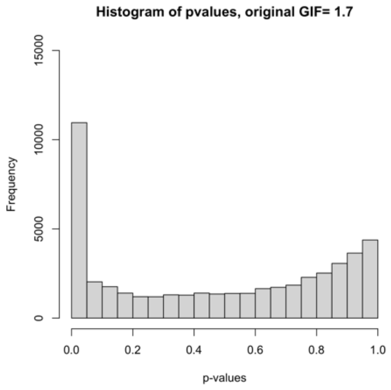

viii) *VS-fwd*

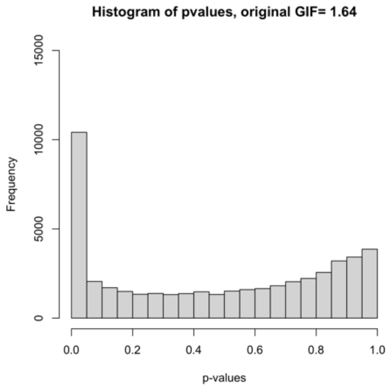

Supp Figure S11: *GEA model.*

Assessment of redundancy analysis (RDA) model performance based on two metrics: **a)** RDA model's adjusted-R^2 values, and **b)** the proportion of high-impact SNPs (p_S) that were detected as candidates by RDA models. Models were built with one of eight variable sets: *VS-single* models with variables at a single spatial resolution (0.5, 1, 2, 4, 8, 16m) are on the x-axis, *VS-all* models built with all variables at all spatial resolutions (red line) and the *VS-fwd* models built with variables selected using forward selection methods (blue line). All models included elevation at 0.5m spatial resolution. The RDA models were performed at the local and regional levels. Note: grey dashed lines between points are indicative only and each model is independent.

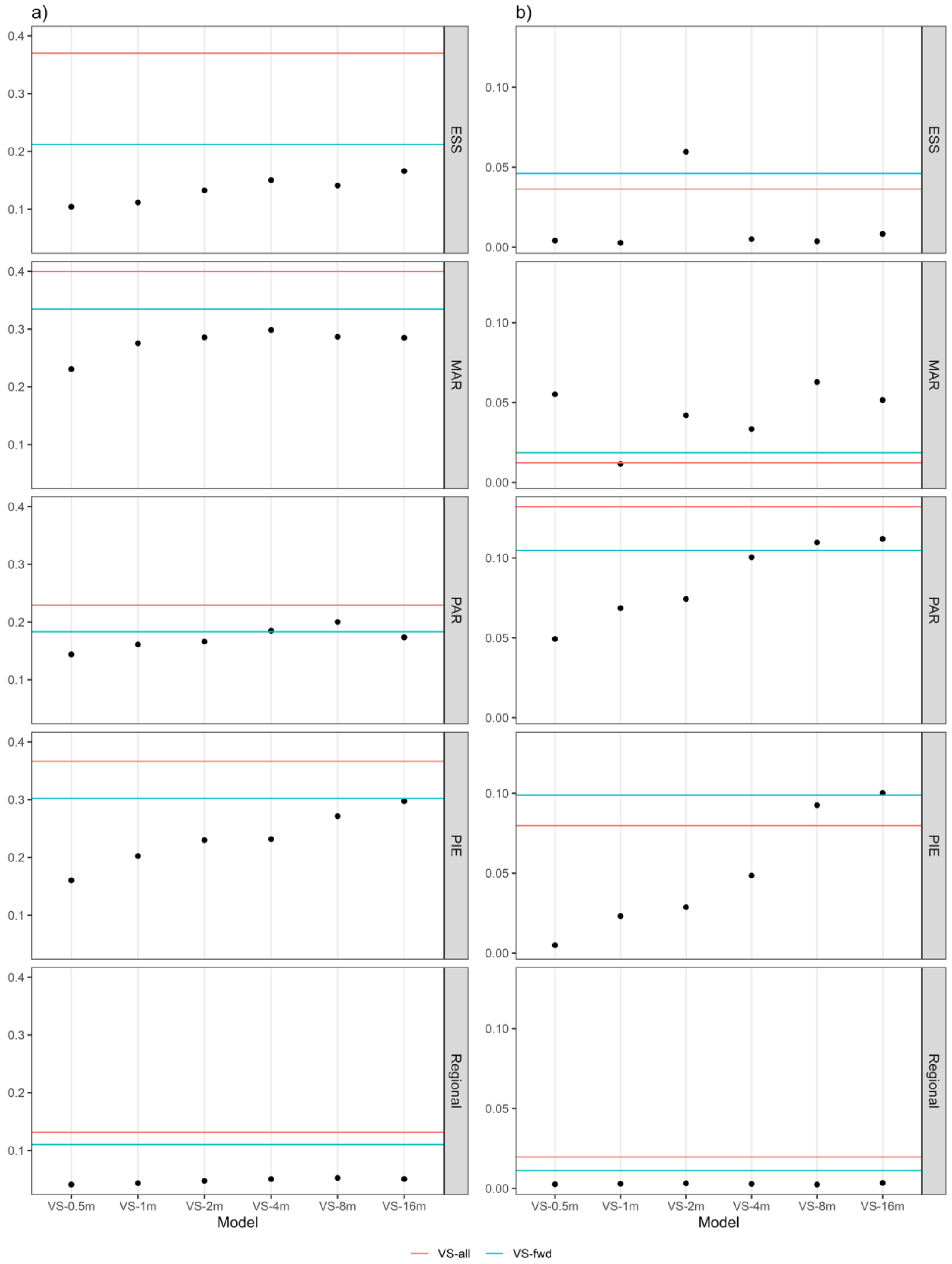

Supp Figure S12: ***GEA model*.**

Upset plots comparing the number of (**i**) high-impact SNPs and (**ii**) all SNPs detected as outliers between redundancy analysis (RDA) models. For each local site (**a-d**) and at the regional level (**e**), we compared RDA models built with one of eight variable sets, where the first six variable sets were based on the nine variable types at one of the following spatial resolutions: 0.5, 1, 2, 4, 8, or 16m, along with elevation at 0.5m spatial resolution (*res0050* to *res1600*). The seventh variable set included all nine variable types at all six spatial resolutions, plus elevation at 0.5m (*All*). The eighth variable set was created using a forward stepwise selection procedure of the *'All'* variable set, such that the variable set was unique to the study site (*Forward*).

In these upset plots, the models built with different variable sets are the rows, where the horizontal bar chart to the left shows the number of SNPs detected. The vertical bar chart shows the number of SNPs that were detected by multiple models, where the comparison is indicated as the dots with a line below each bar; single dots indicate the number of SNPs detected uniquely by the model on the left.

S12 a) Essets

i) High-impact candidate SNPs

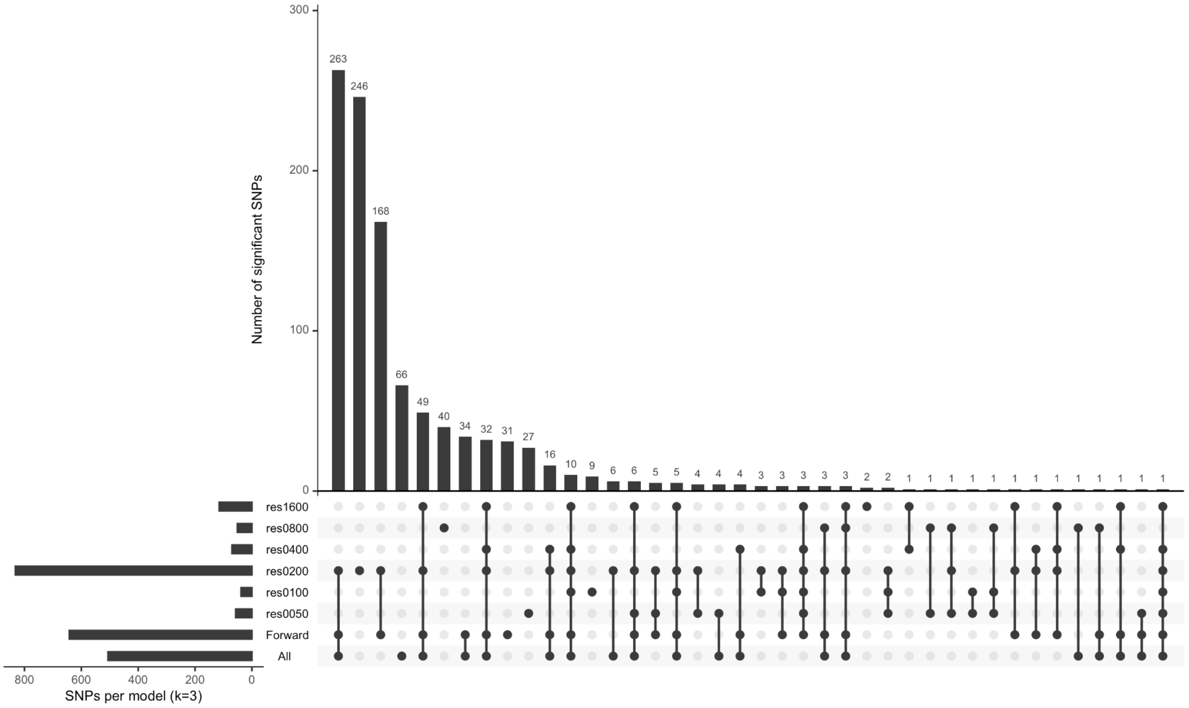

ii) All candidate SNPs

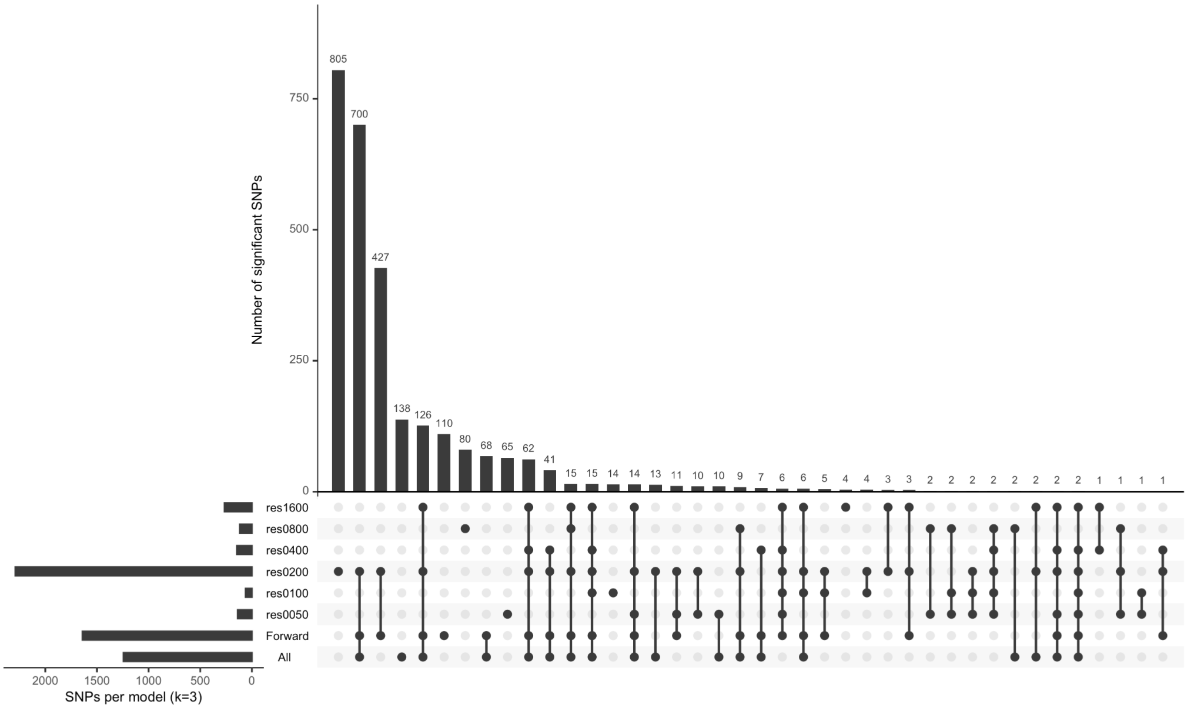

S12 b) Martinets

i) High-impact candidate SNPs

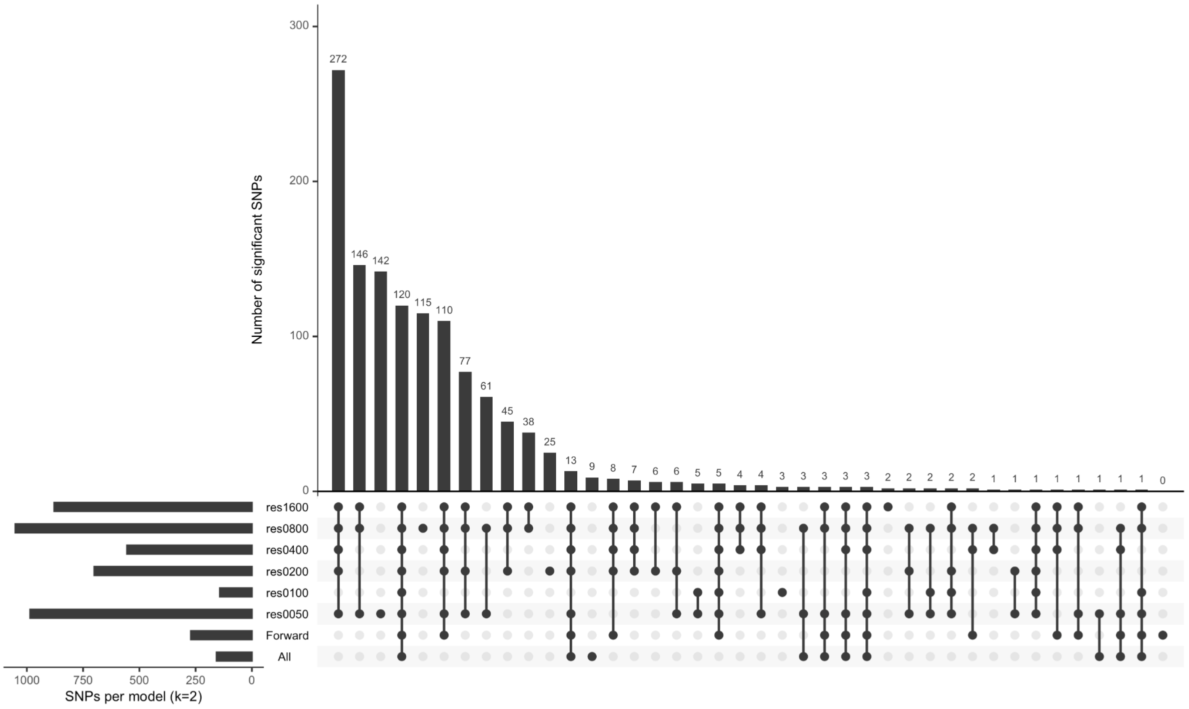

ii) All candidate SNPs

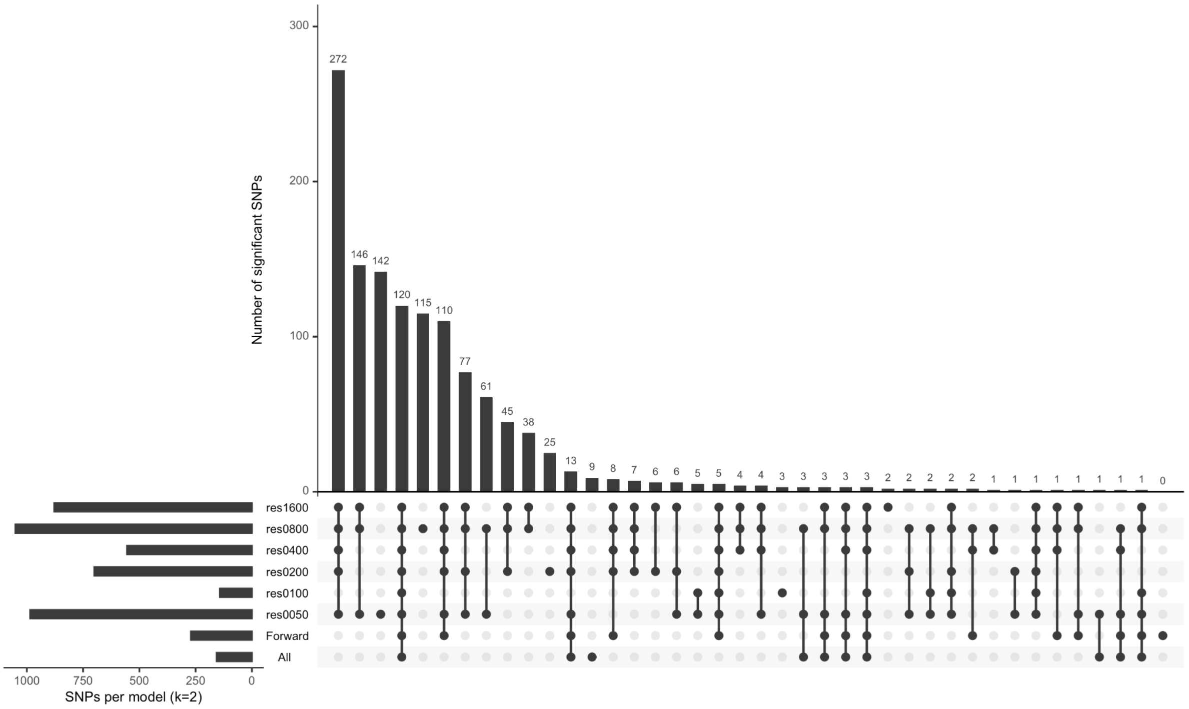

S12 c) Para

i) High-impact candidate SNPs

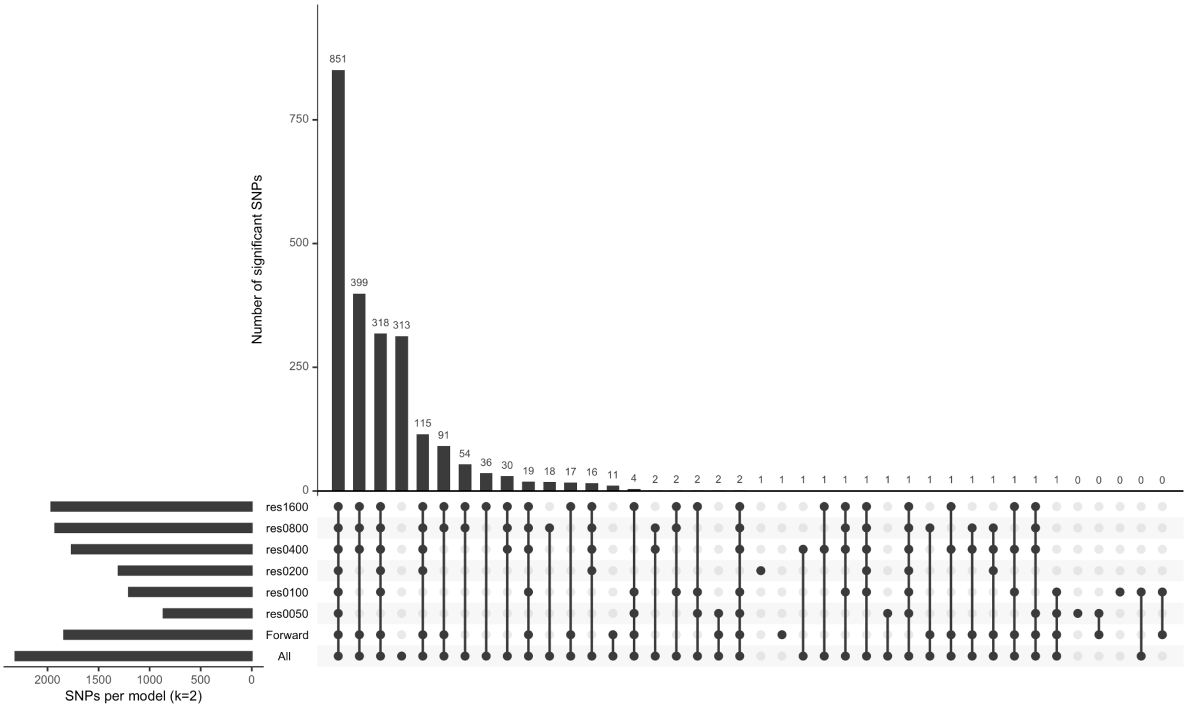

ii) All candidate SNPs

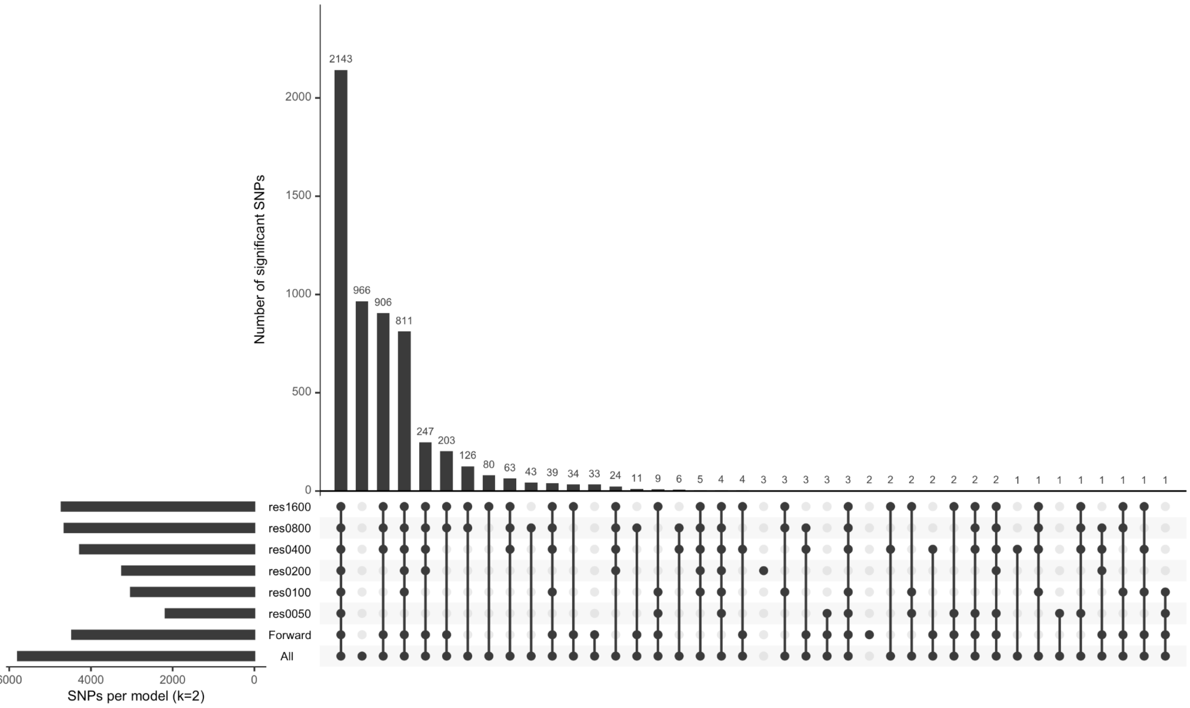

S12 d) Pierredar

i) High-impact candidate SNPs

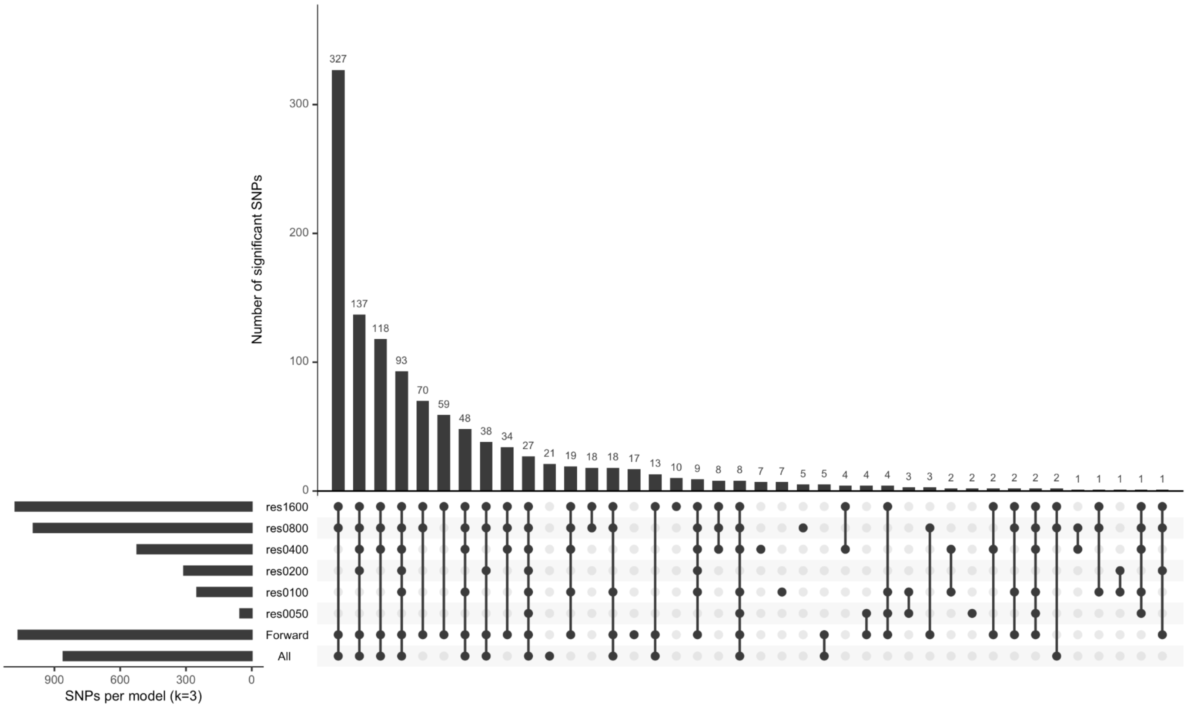

ii) All candidate SNPs

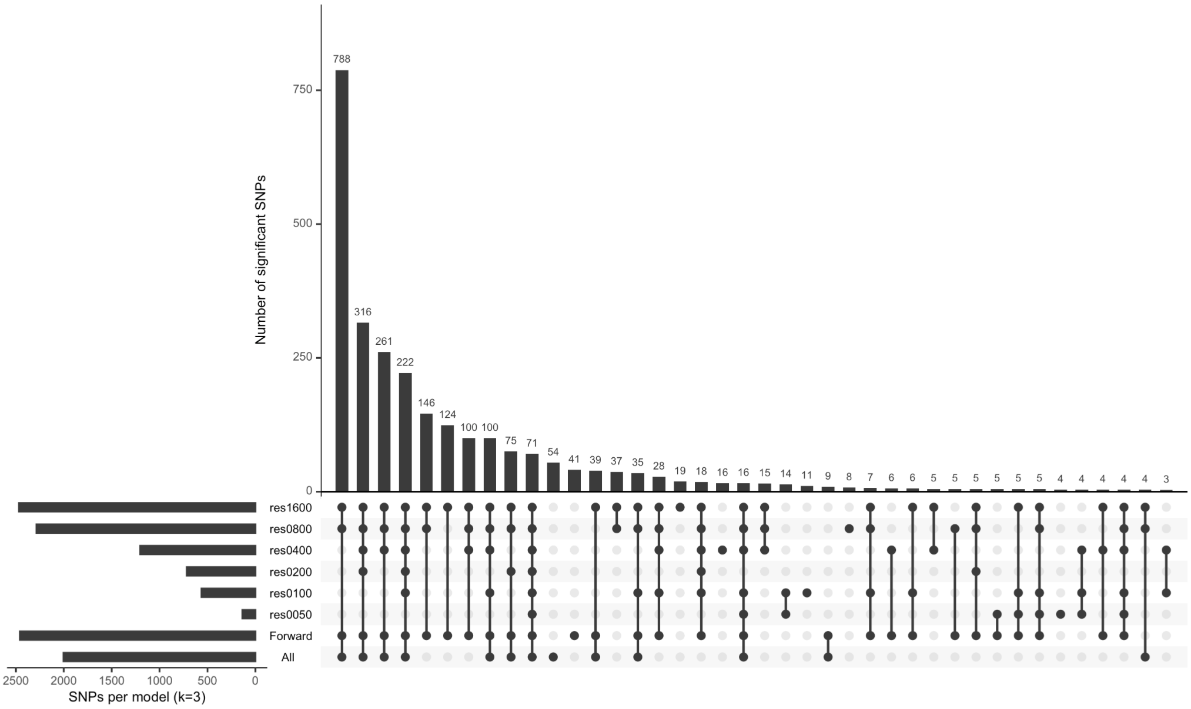

S12 e) Regional

i) High-impact candidate SNPs

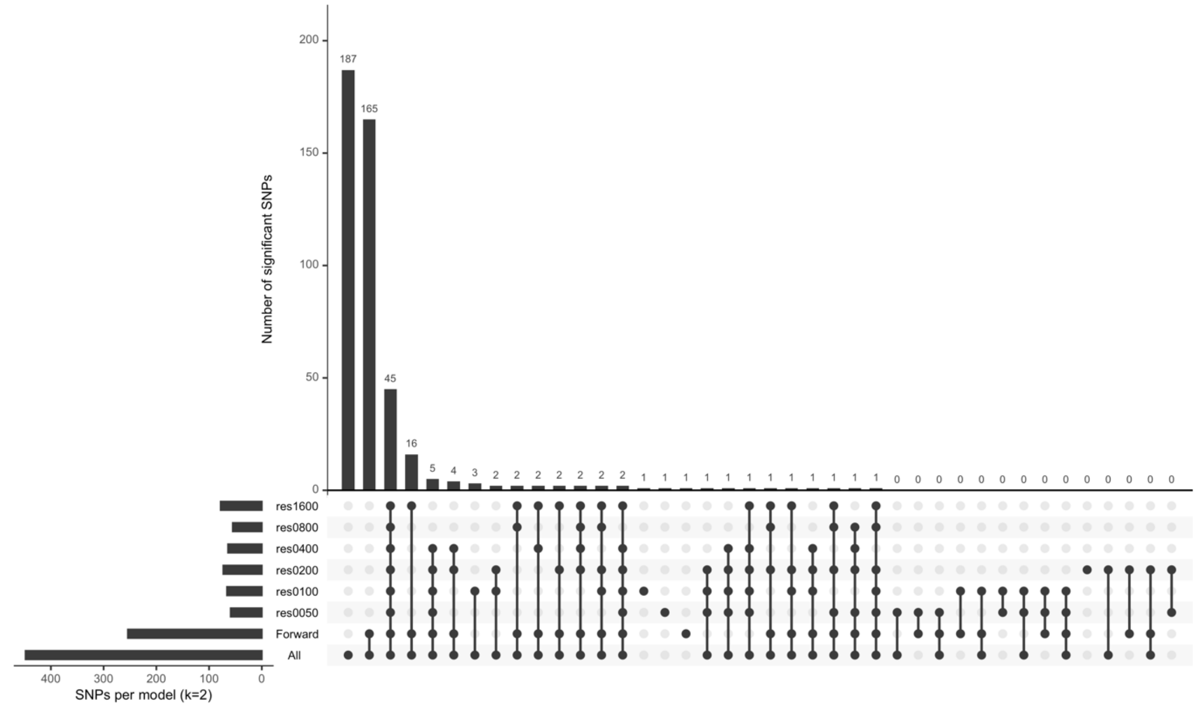

ii) All candidate SNPs

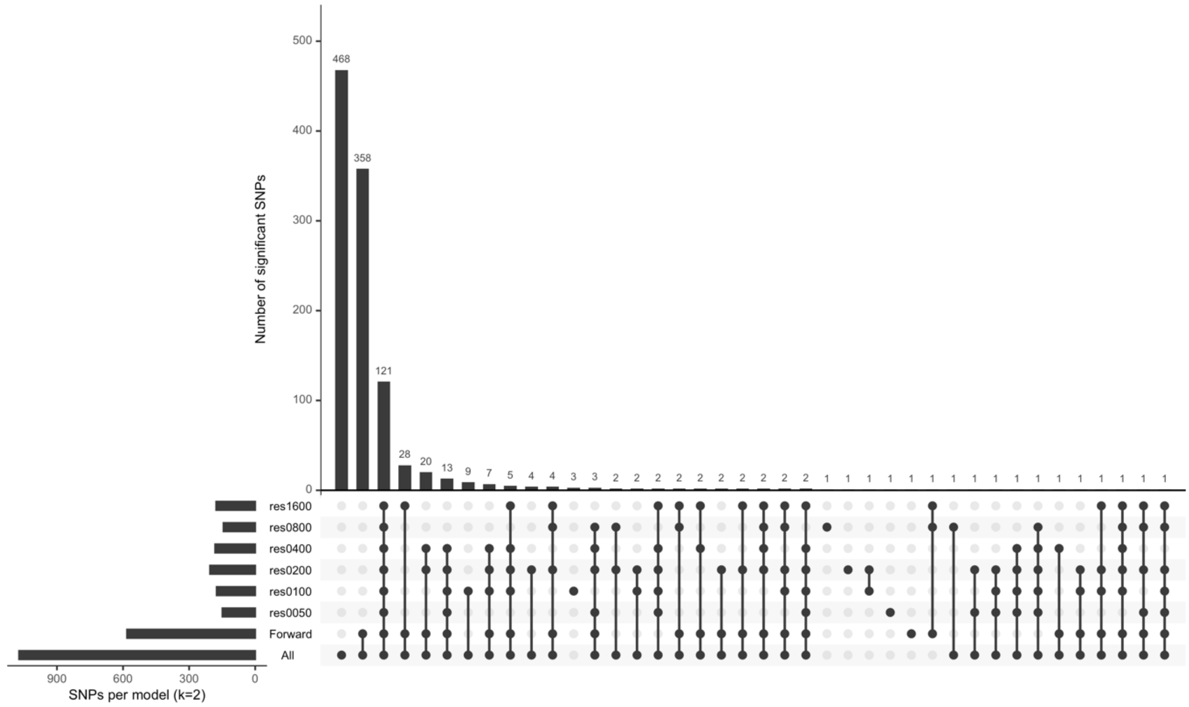

Supp Figure S13: ***GEA model*.**

Biplots that show the projection of loci and environmental variables across the first *K*-retained axes for each redundancy analysis (RDA), where the RDA1 axis is always on the x-axis. Points represent loci that were rescaled by a factor of 20 to aid in readability. Outlier loci are indicated by dots coloured by the environmental variable that they have the greatest association with, while non-outlier loci are dots coloured in dark grey. The environmental variables are given by the arrow vectors and are colour coded by variable type and spatial resolution. The RDA models were performed at the local (a-d) and regional (e) levels. The models were built with one of eight variable sets: i-vi) *VS-single* models with variables at a single spatial resolution (0.5, 1, 2, 4, 8, 16m), vii) *VS-all* models built with all variables at all spatial resolutions and viii) *VS-fwd* models built with variables selected using forward selection methods. All models included elevation at 0.5m spatial resolution. Analyses were performed with the *rda* function of the *vegan* (v2.5-7) R package.

S13 a) Essets:
K=3 retained RDA axes

S13 a) Essets: i) *VS-0.5m*

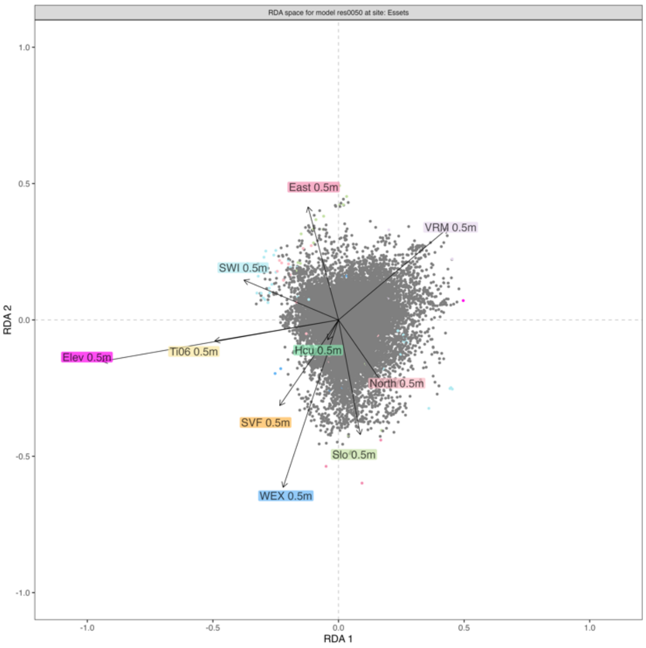

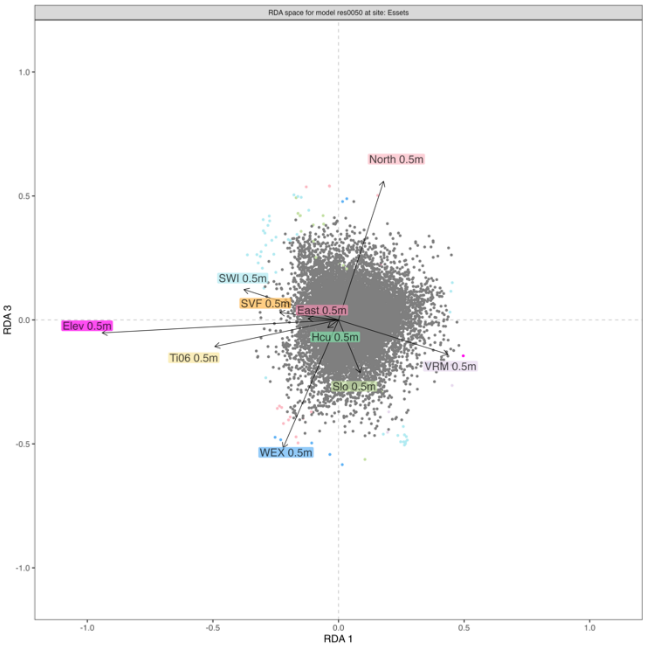

S13 a) Essets: ii) *VS-1m*

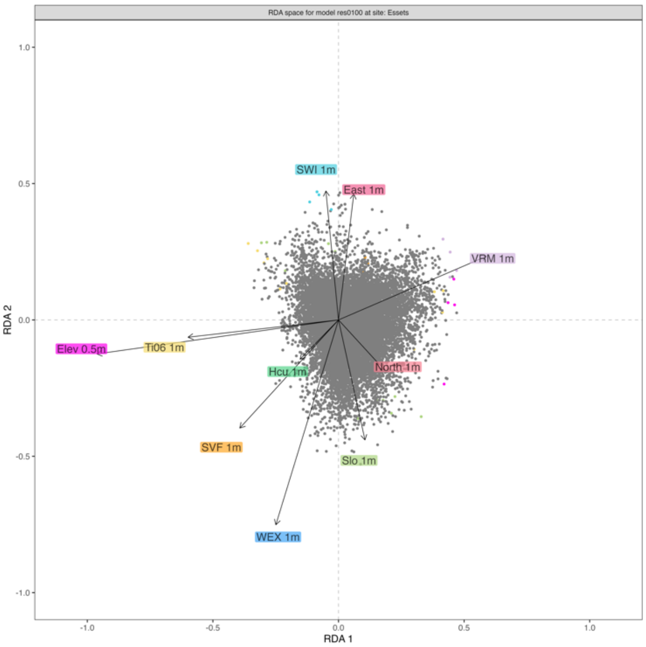

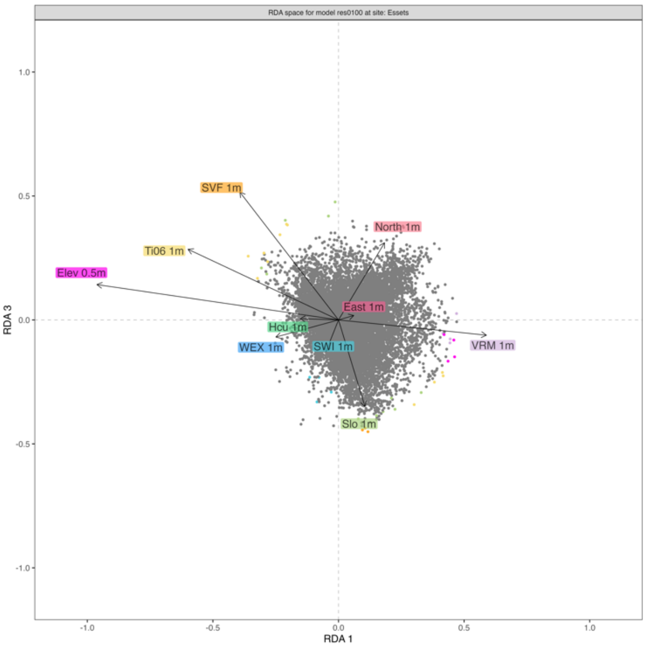

S13 a) Essets: iii) *VS-2m*

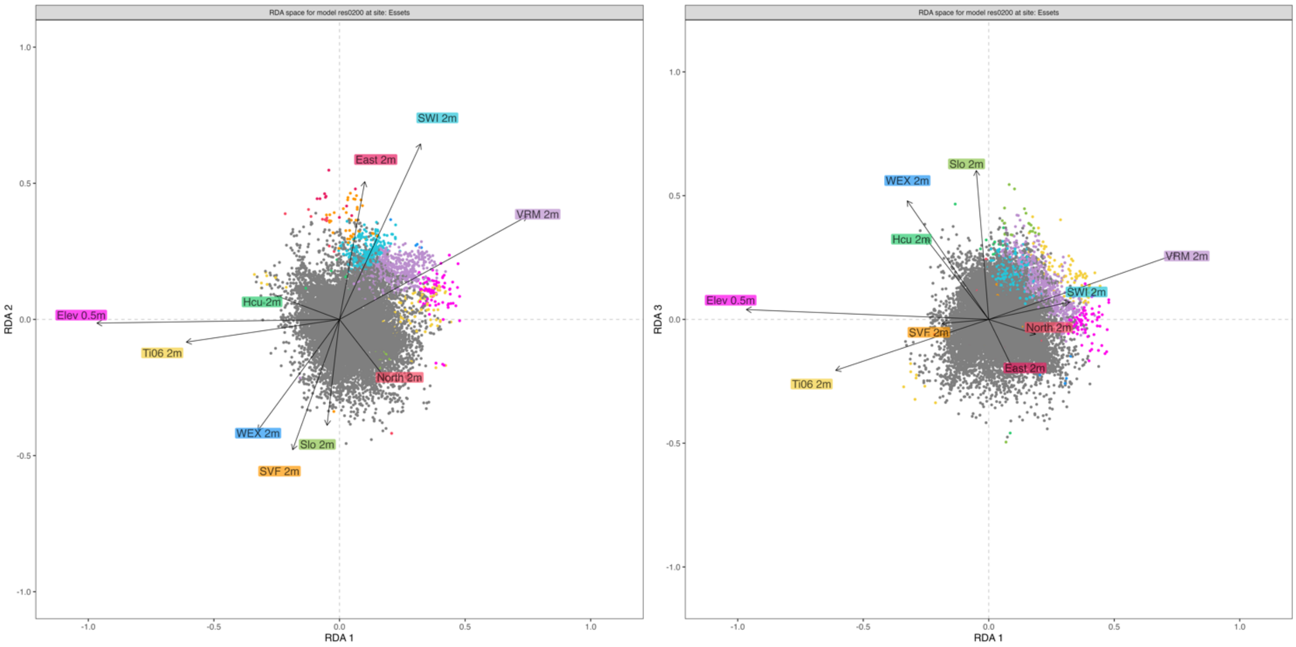

S13 a) Essets: iv) *VS-4m*

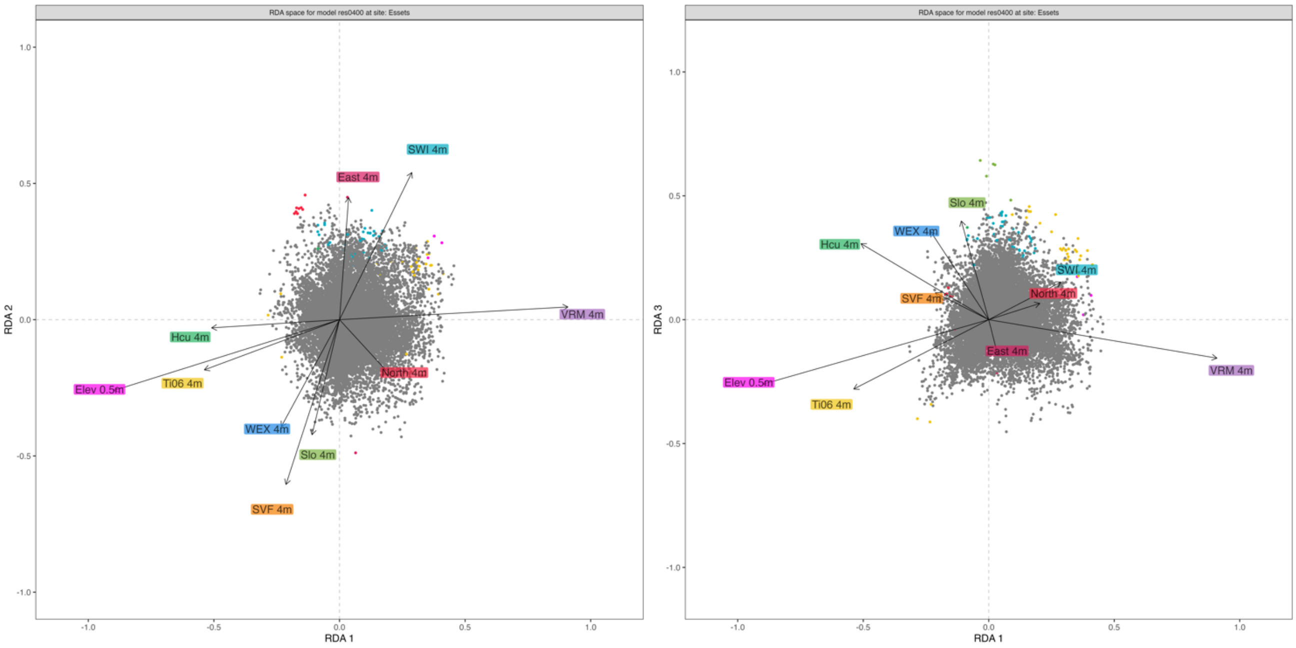

S13 a) Essets: v) *VS-8m*

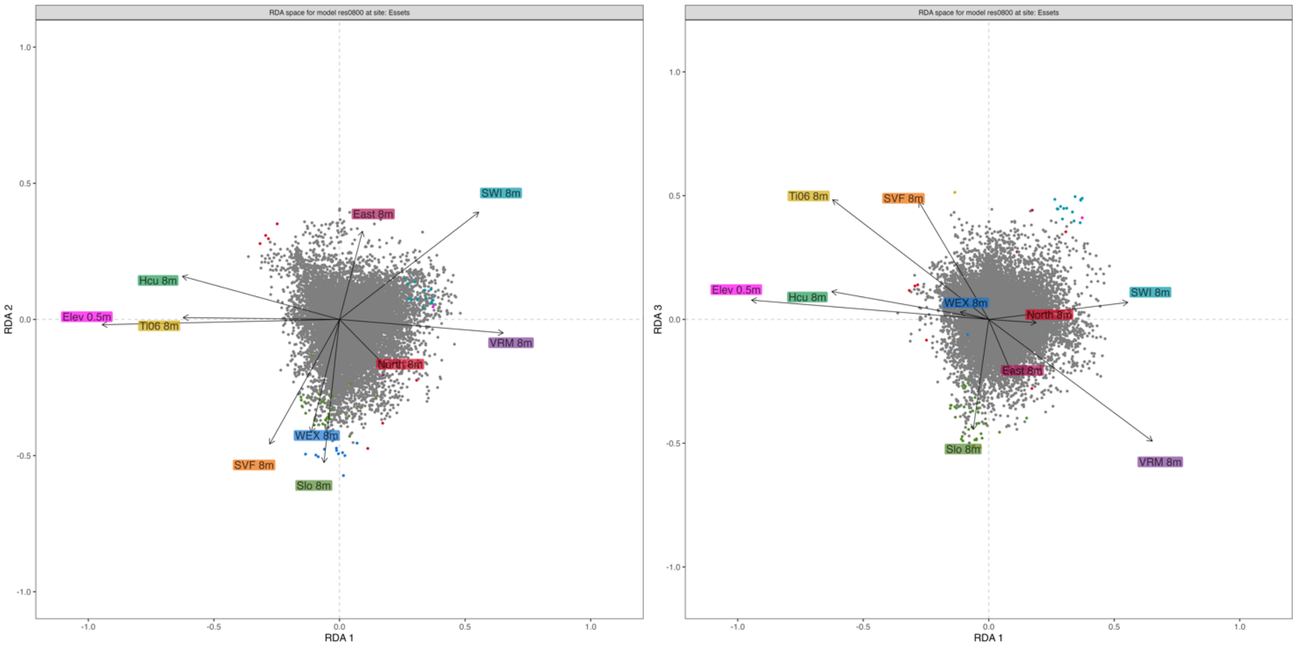

S13 a) Essets: vi) *VS-16m*

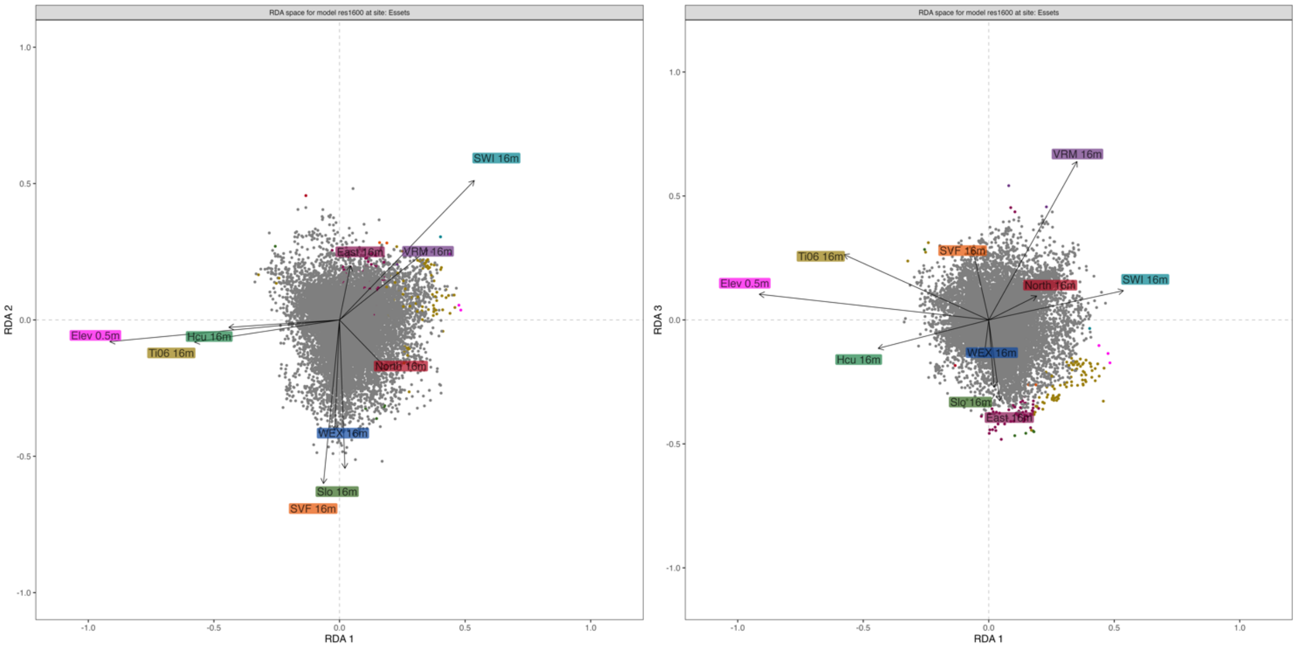

S13 a) Essets: vii) *VS-all*

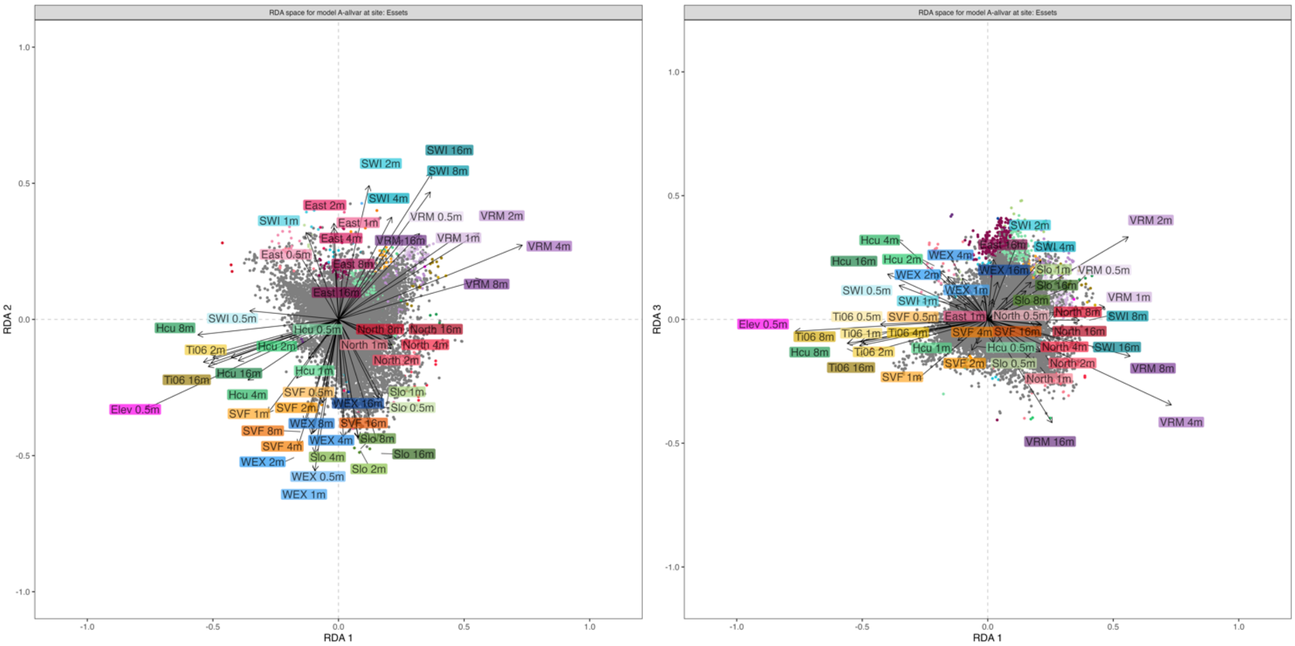

S13 a) Essets: viii) *VS-fwd*

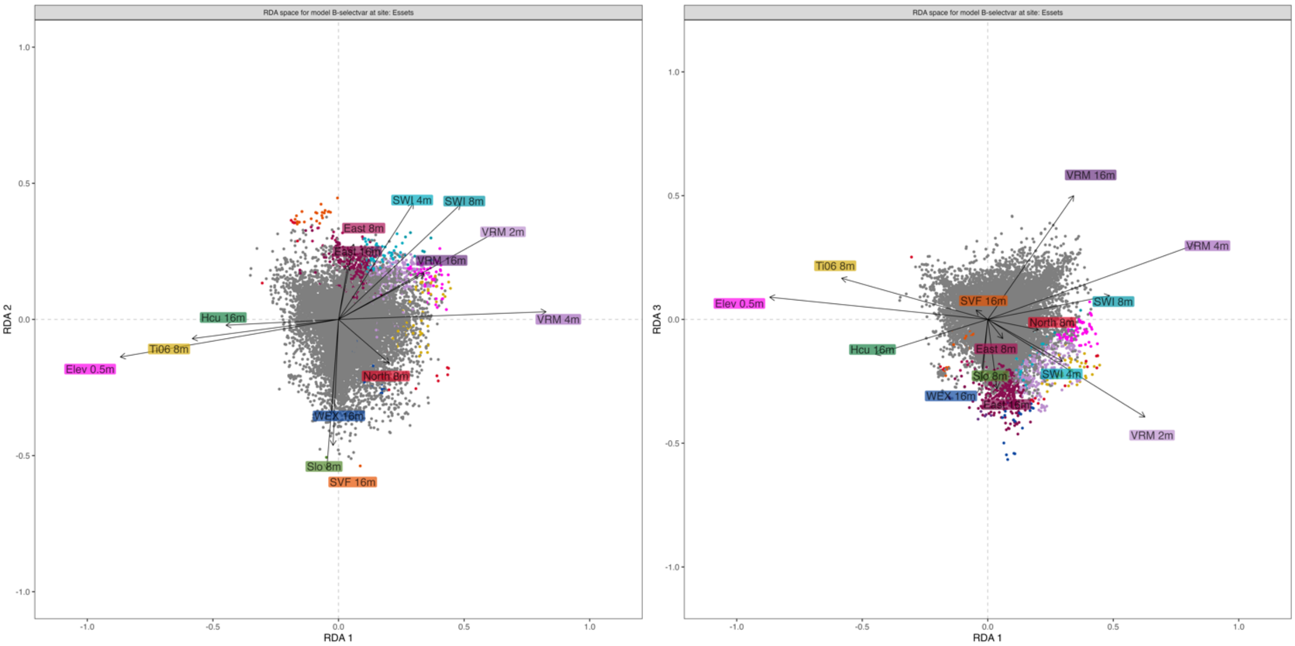

b) Martinets:
K=2 retained RDA axes

S13 b) Martinets: **i)** *VS-0.5m*

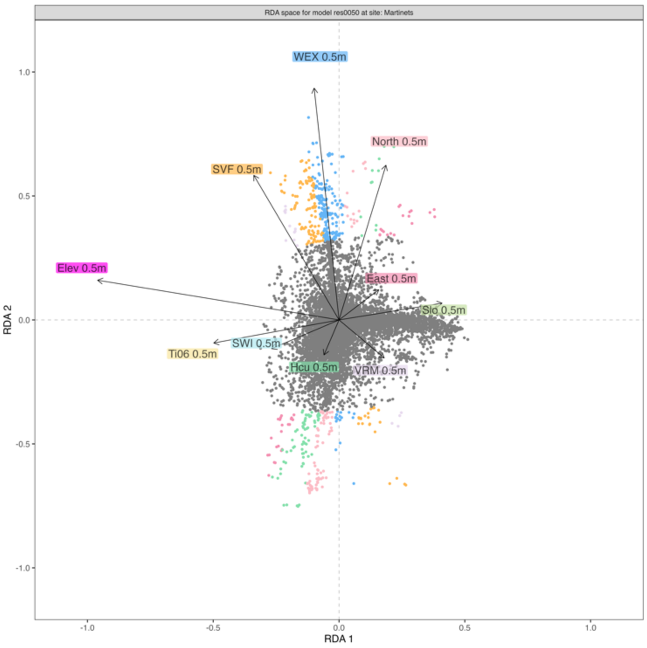

S13 b) Martinets: **ii)** *VS-1m*

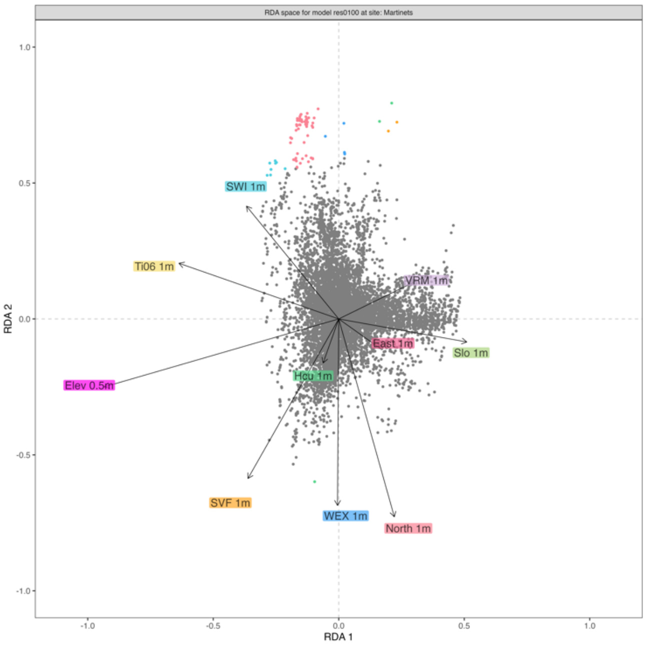

S13 b) Martinets: **iii)** *VS-2m*

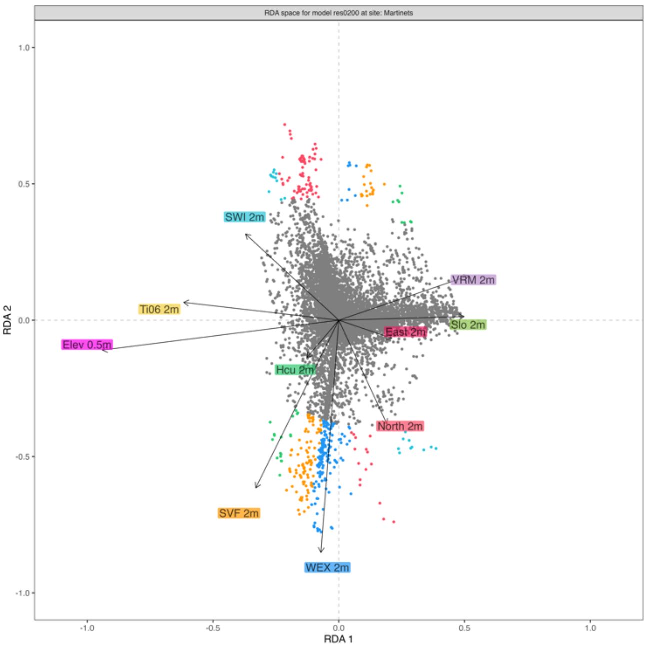

S13 b) Martinets: **iv)** *VS-4m*

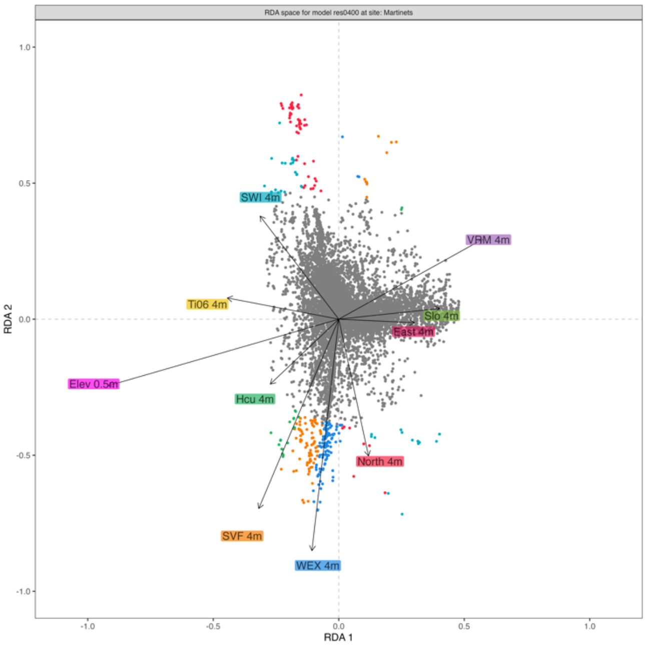

S13 b) Martinets: v) *VS-8m*

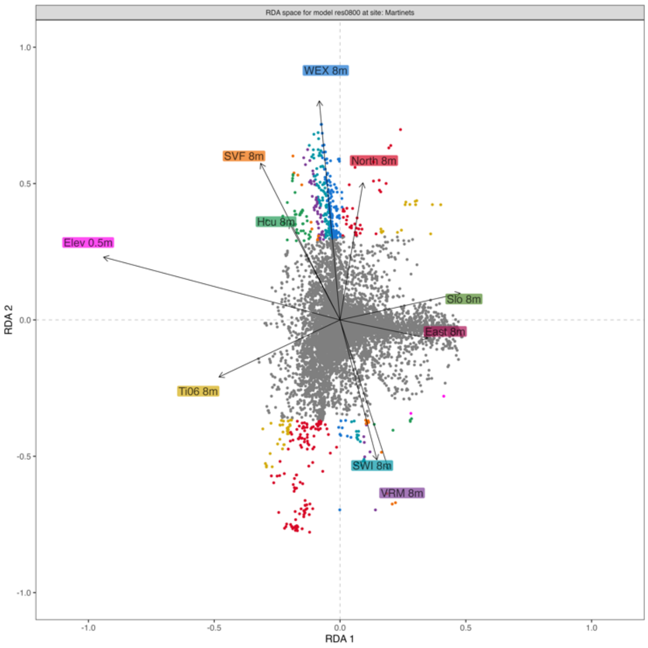

S13 b) Martinets: vi) *VS-16m*

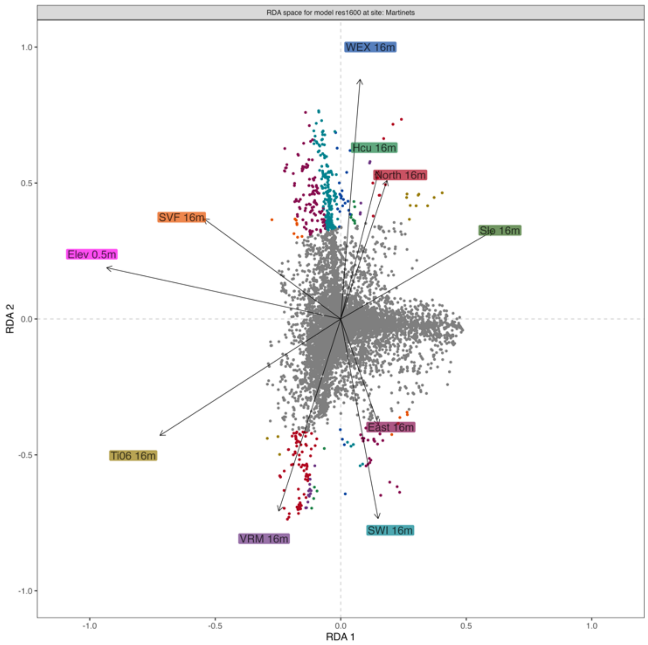

S13 b) Martinets: vii) *VS-all*

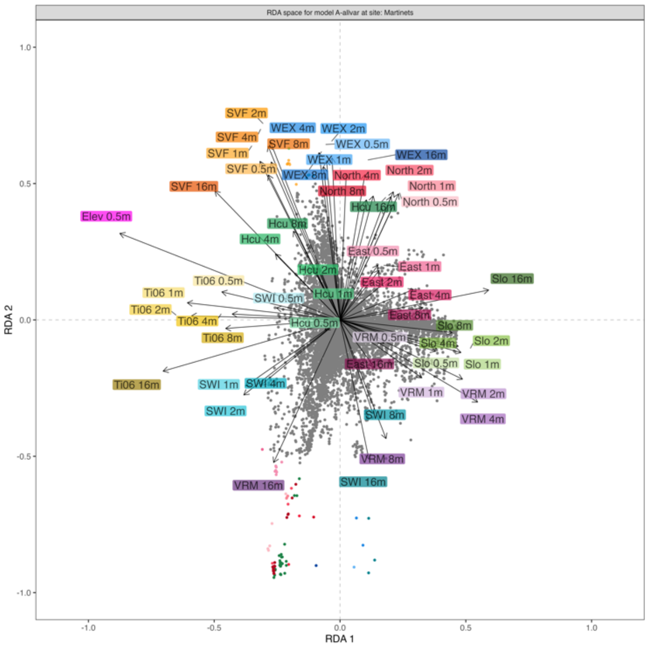

S13 b) Martinets: viii) *VS-fwd*

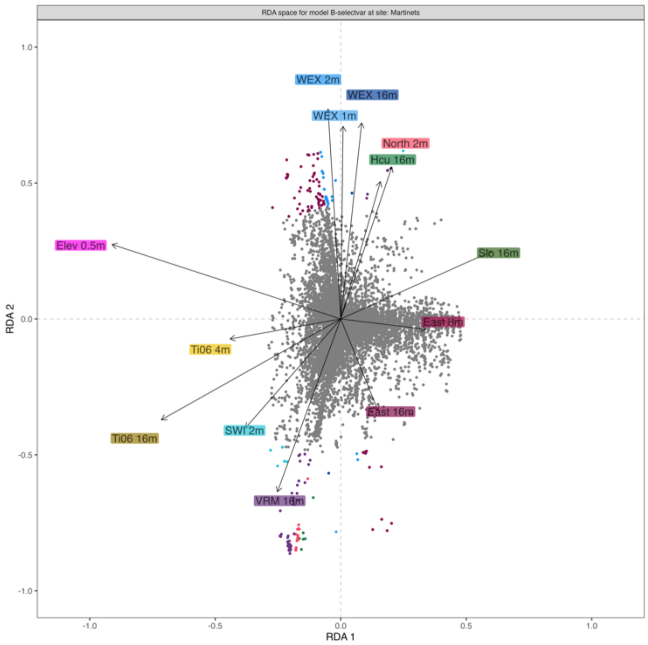

S13 c) Para:
K=2 retained RDA axes

S13 c) Para: **i)** *VS-0.5m*

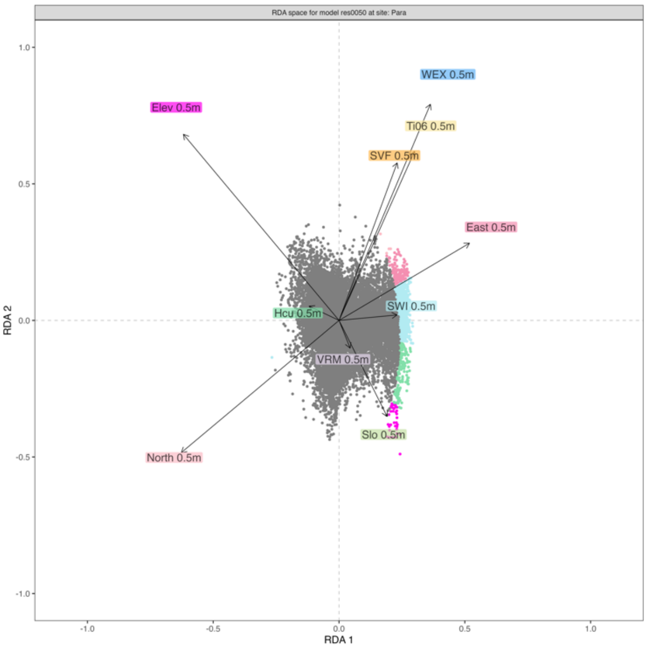

S13 c) Para: **ii)** *VS-1m*

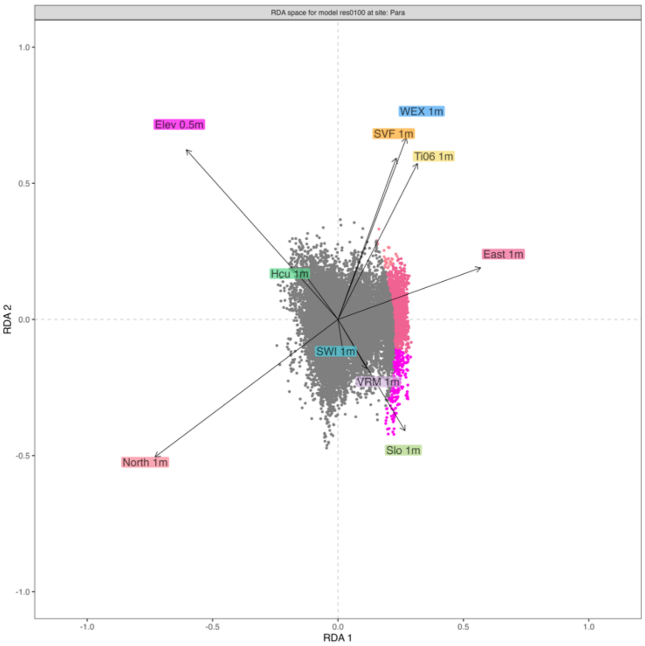

S13 c) Para: **iii)** *VS-2m*

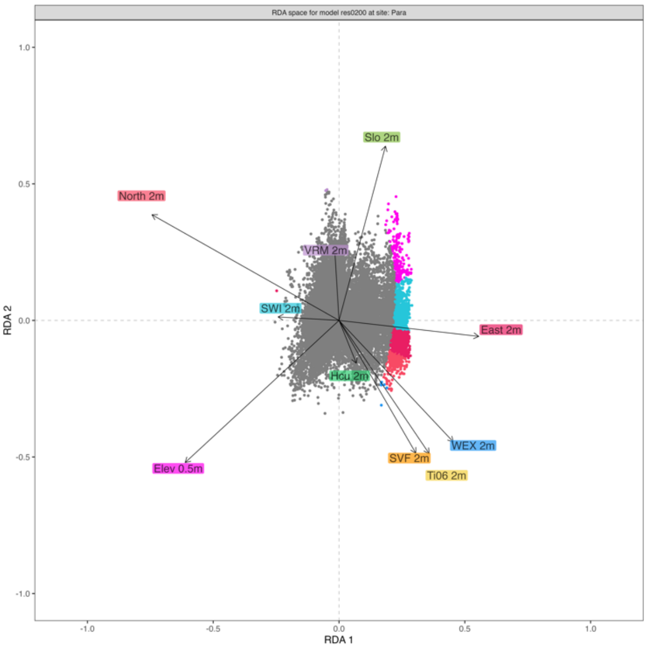

S13 c) Para: **iv)** *VS-4m*

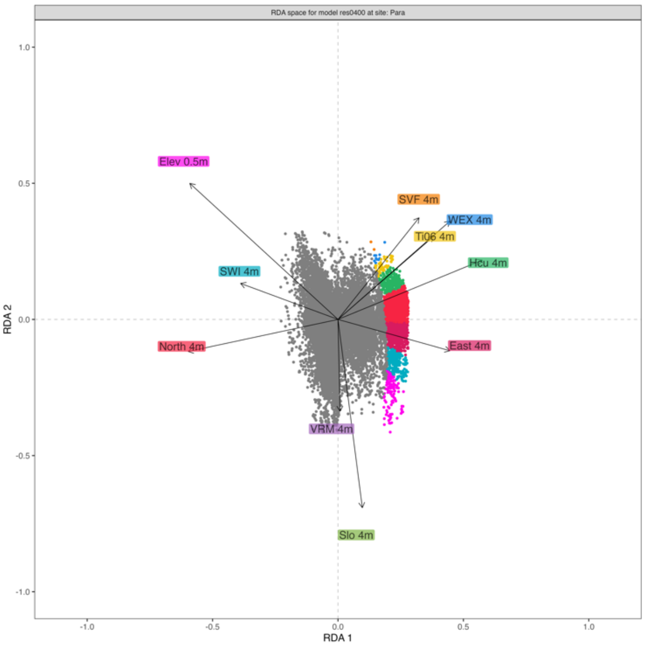

S13 c) Para: v) *VS-8m*

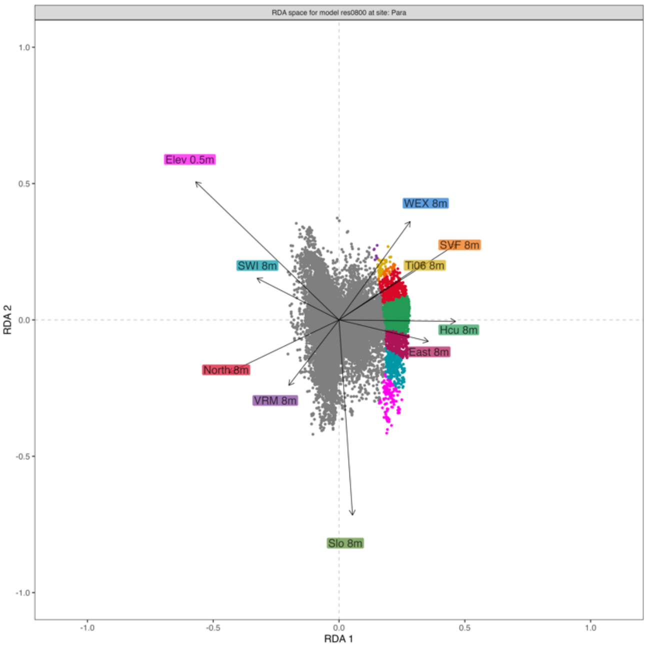

S13 c) Para: vi) *VS-16m*

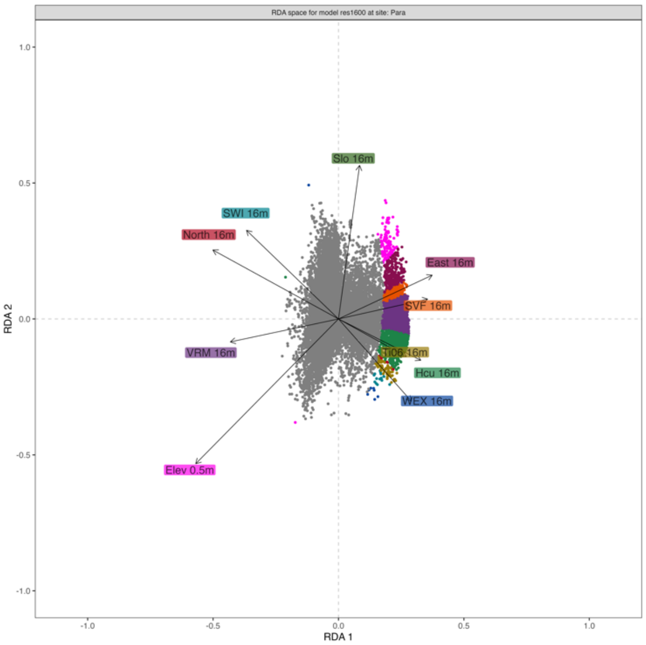

S13 c) Para: vii) *VS-all*

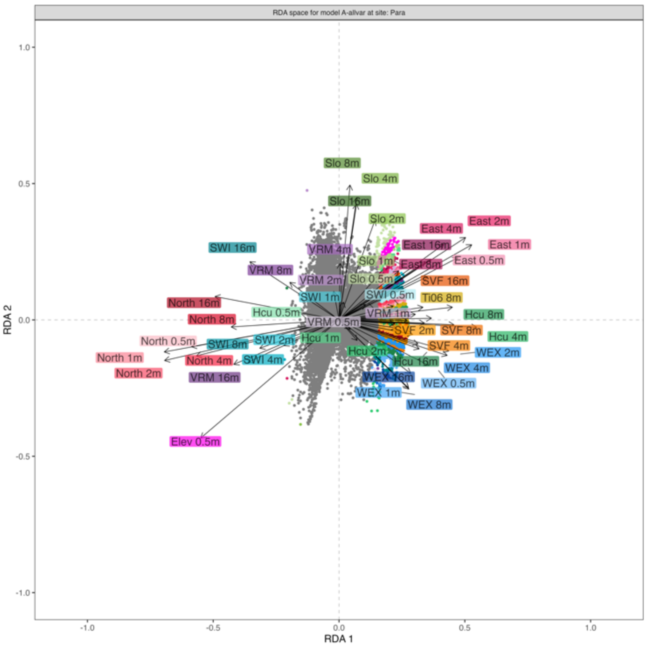

S13 c) Para: viii) *VS-fwd*

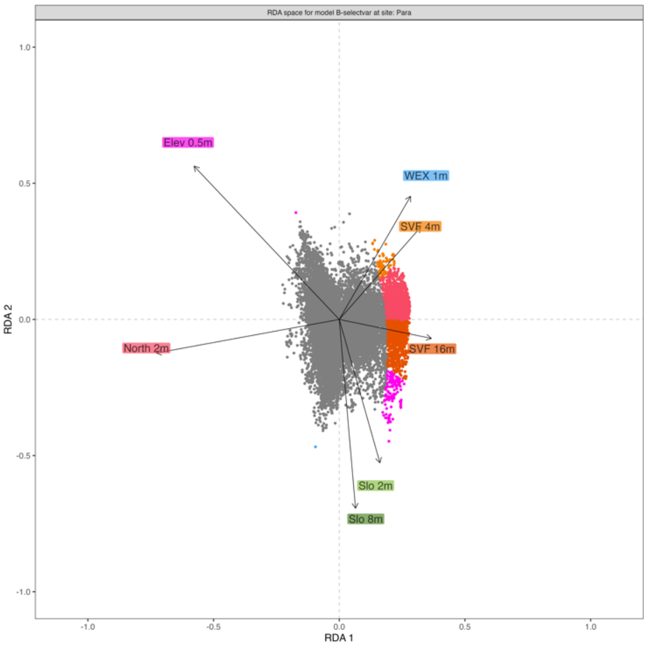

S13 d) Pierredar:

K=3 retained RDA axes

S13 d) Pierredar: i) *VS-0.5m*

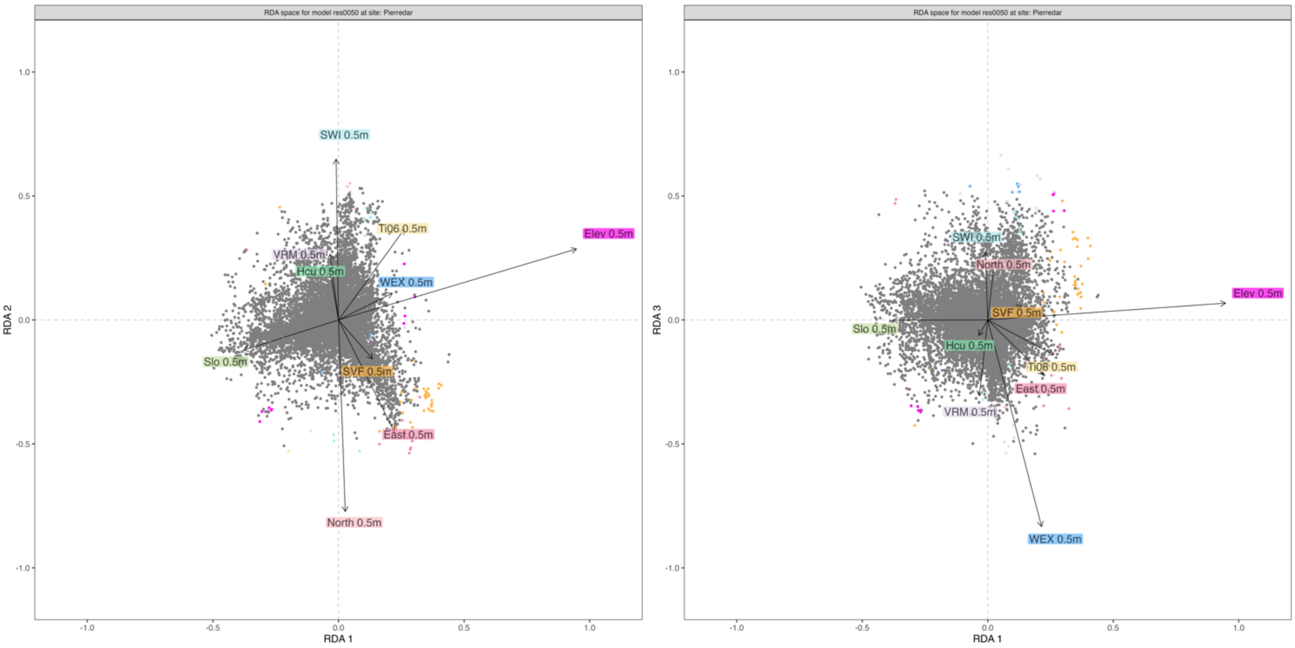

S13 d) Pierredar: ii) *VS-1m*

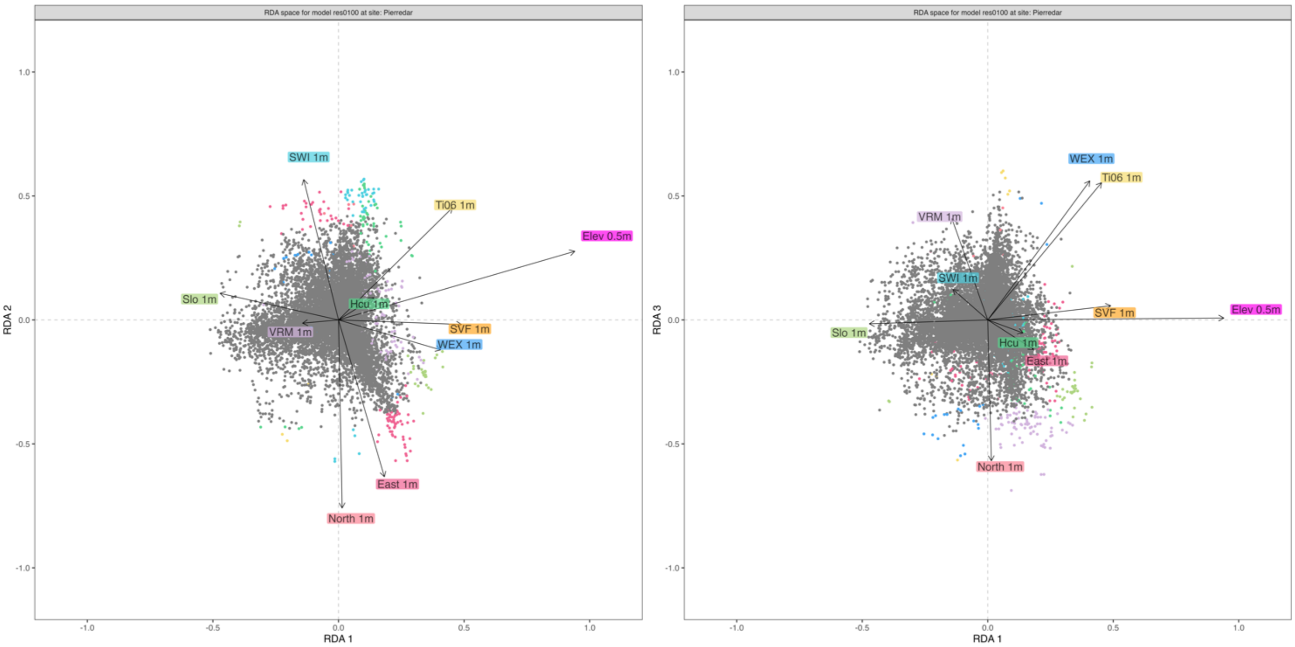

S13 d) Pierredar: iii) *VS-2m*

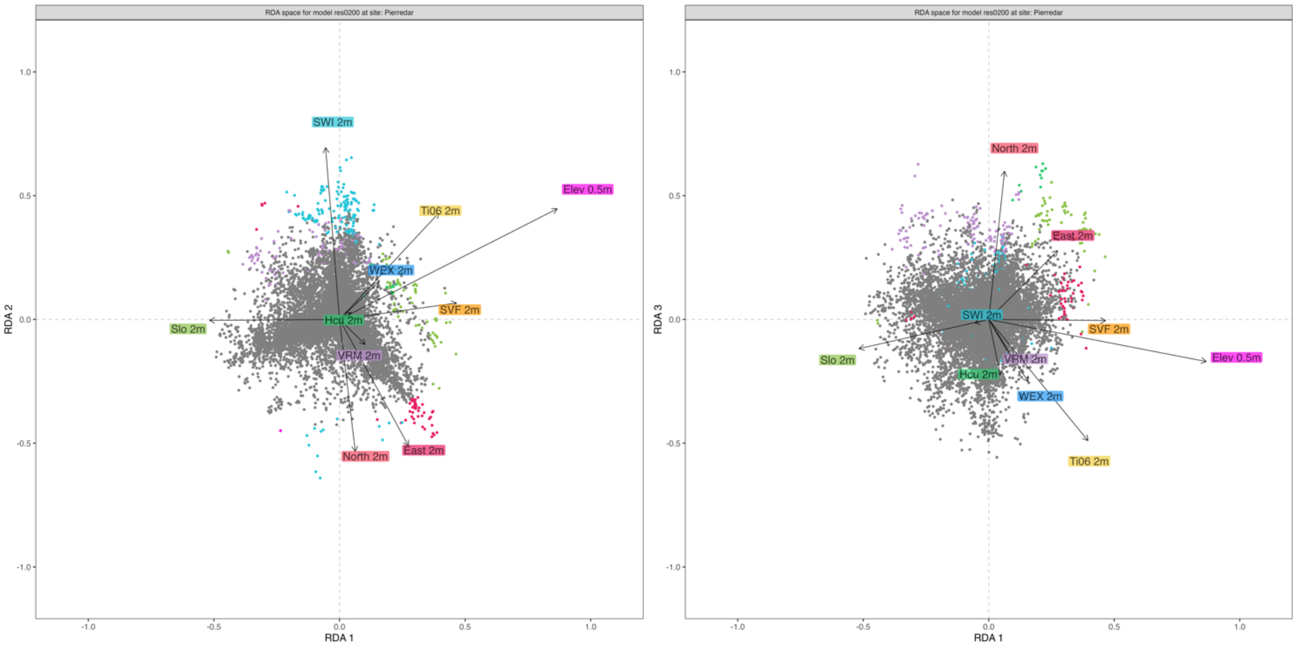

S13 d) Pierredar: iv) *VS-4m*

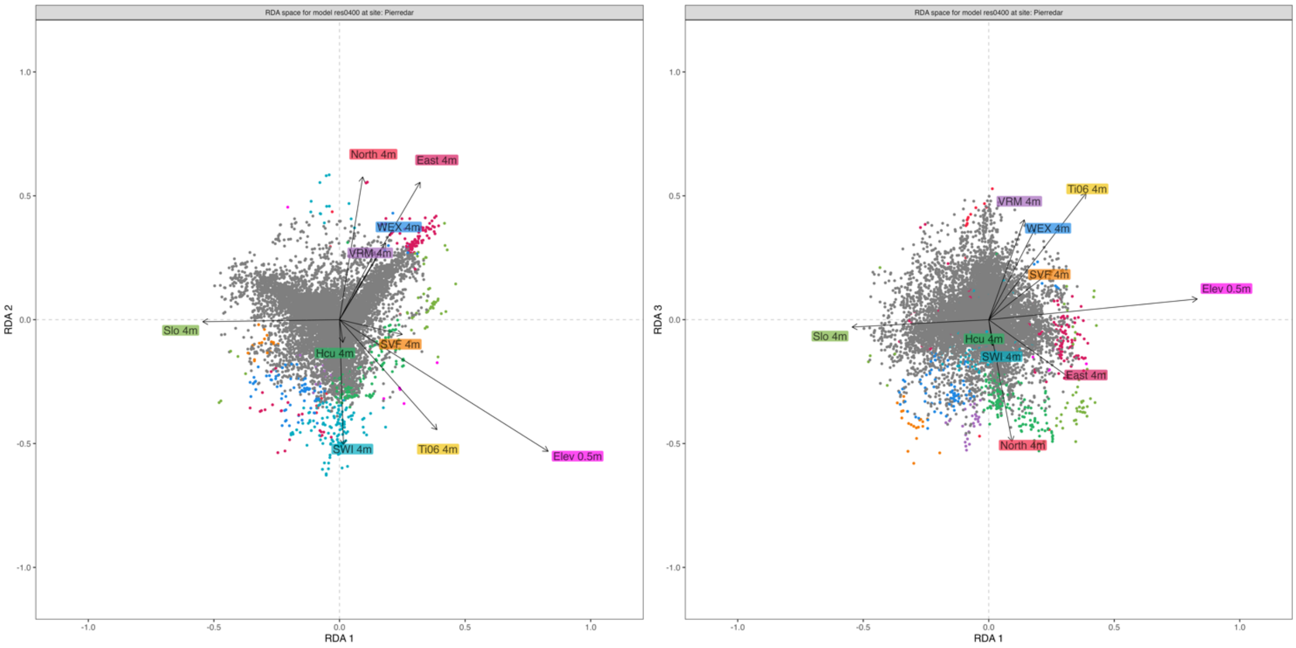

S13 d) Pierredar: v) *VS-8m*

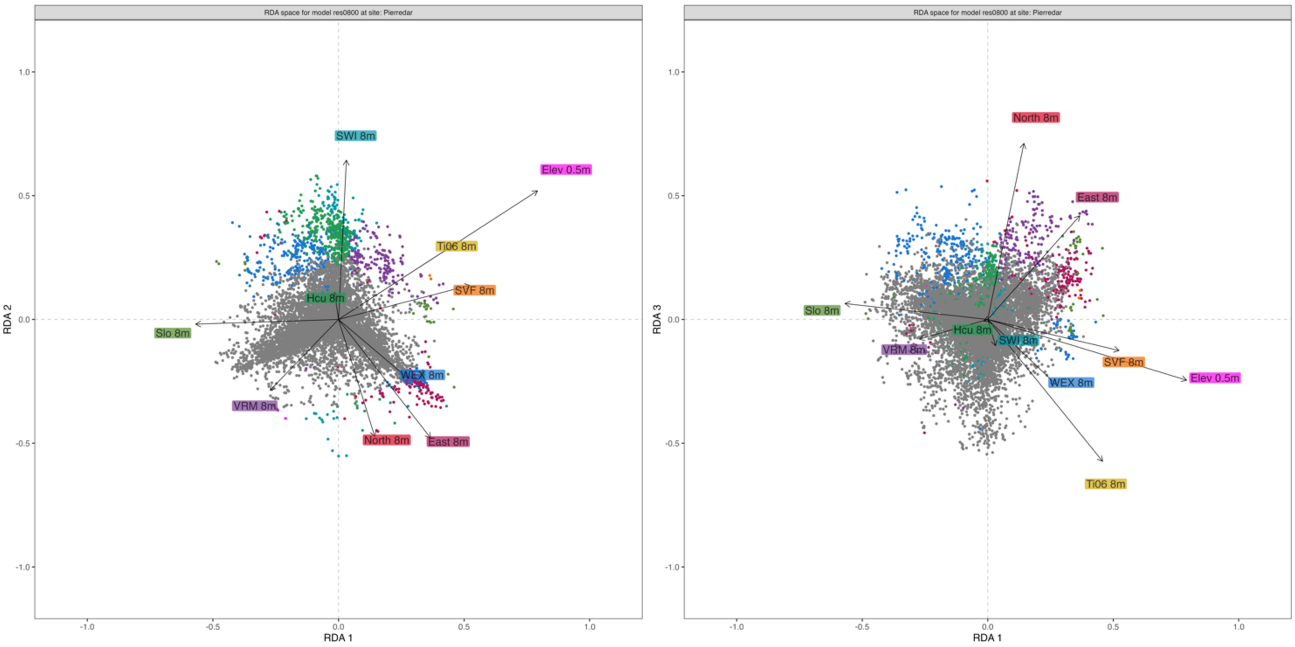

S13 d) Pierredar: vi) *VS-16m*

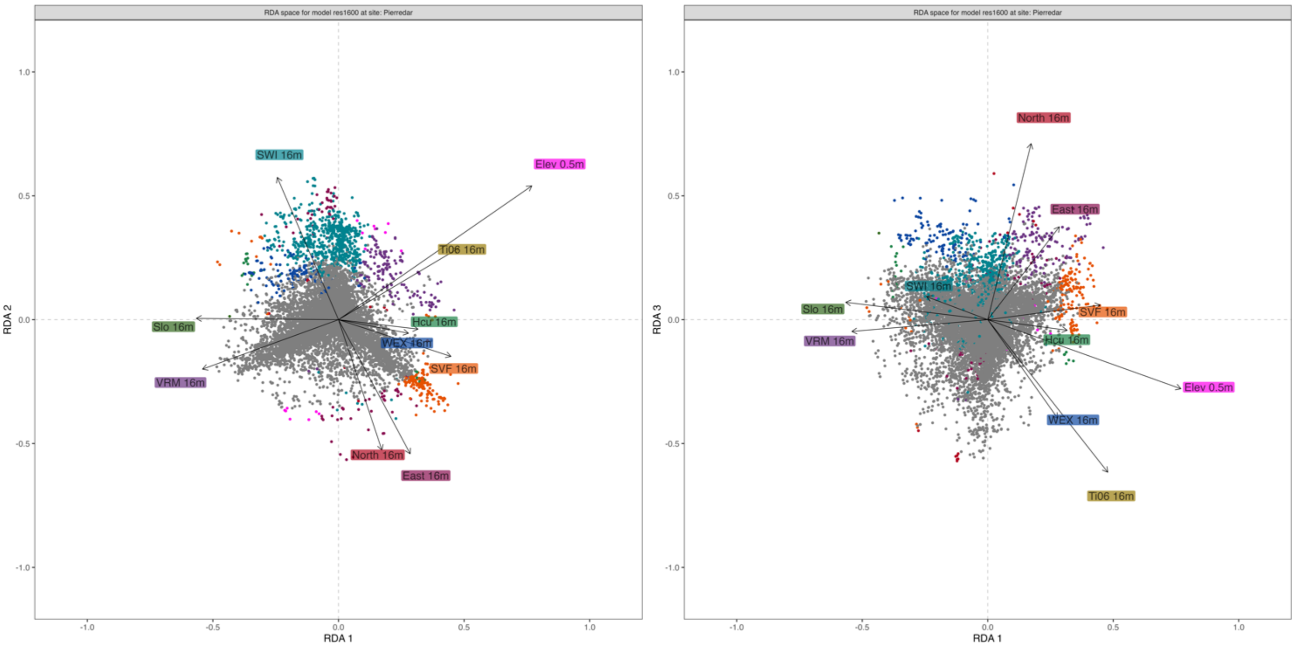

S13 d) Pierredar: vii) *VS-all*

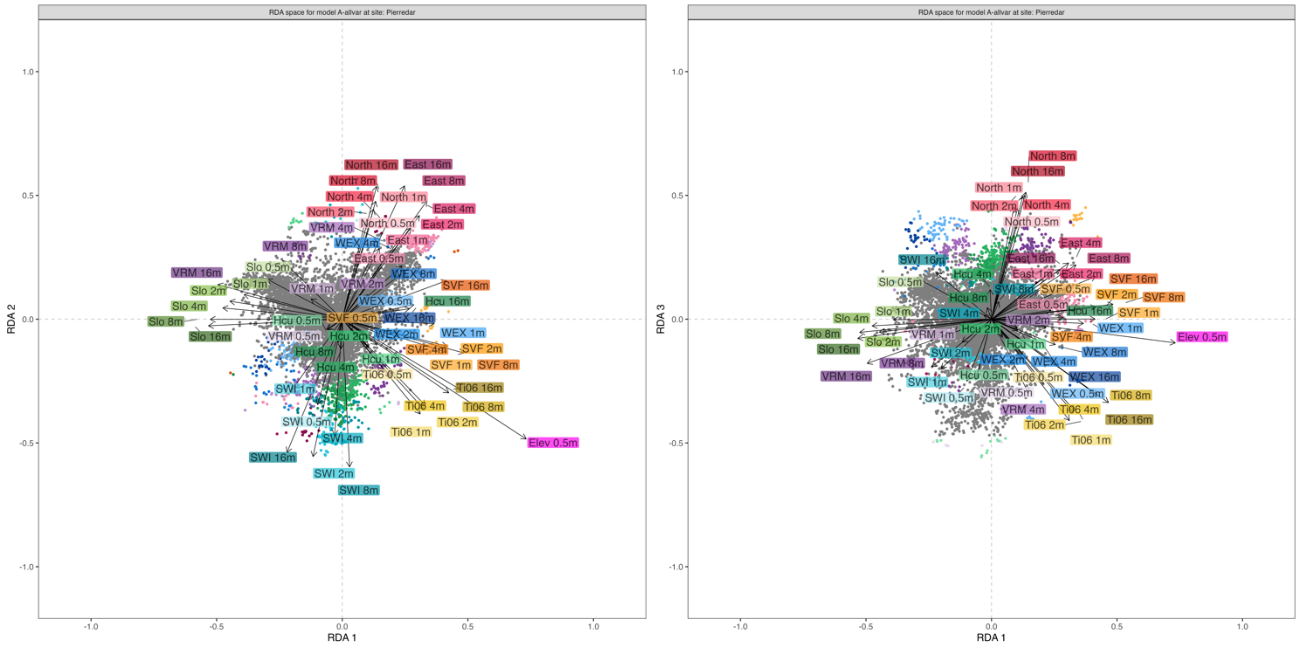

S13 d) Pierredar: viii) *VS-fwd*

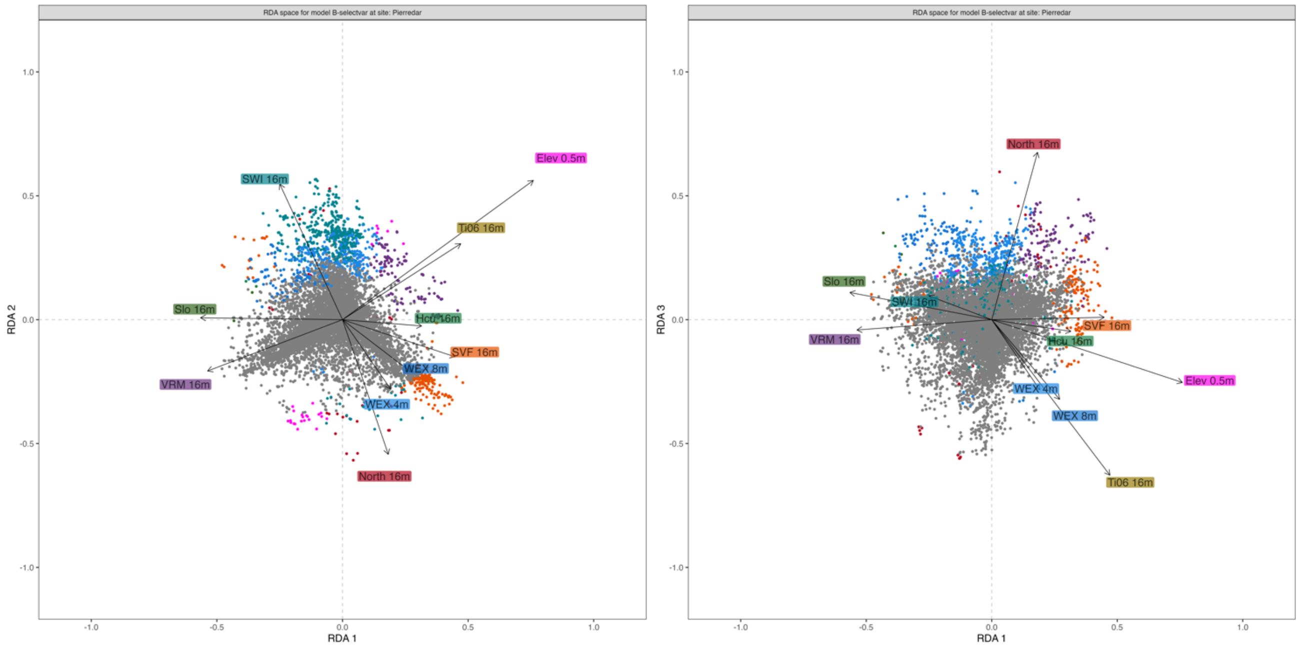

e) Regional:

K=2 retained RDA axes

S13 e) Regional: **i)** *VS-0.5m*

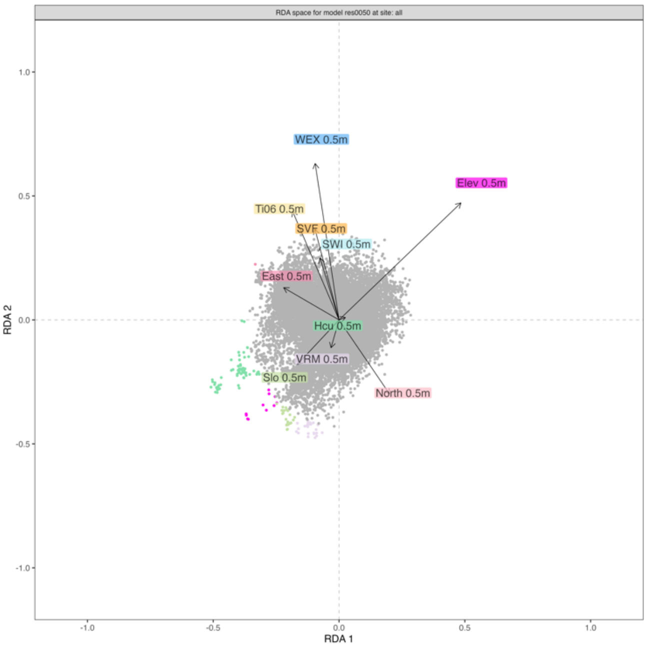

S13 e) Regional: **ii)** *VS-1m*

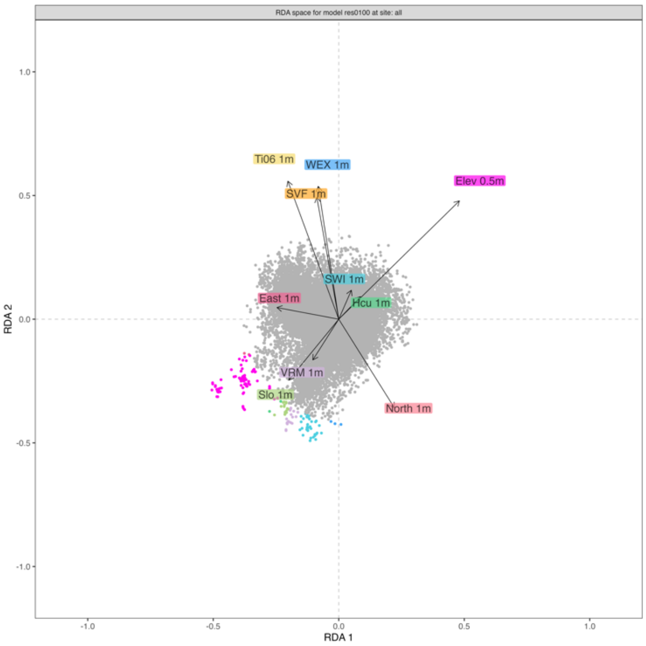

S13 e) Regional: **iii)** *VS-2m*

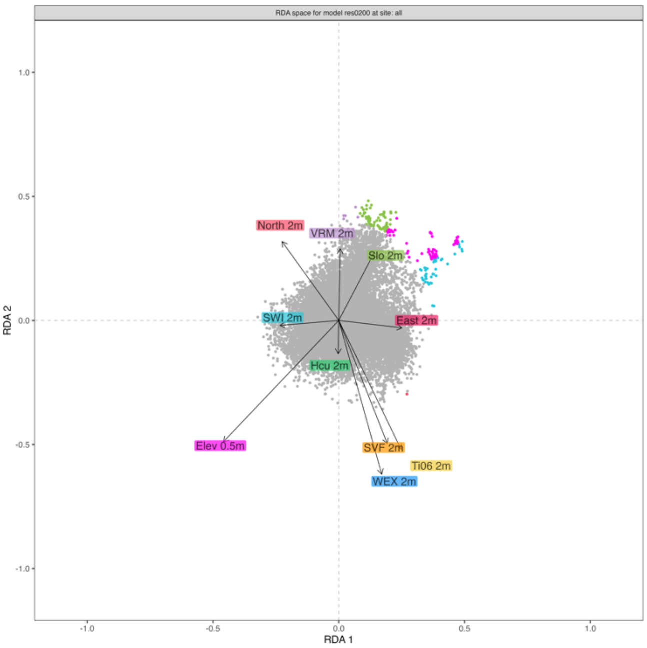

S13 e) Regional: **iv)** *VS-4m*

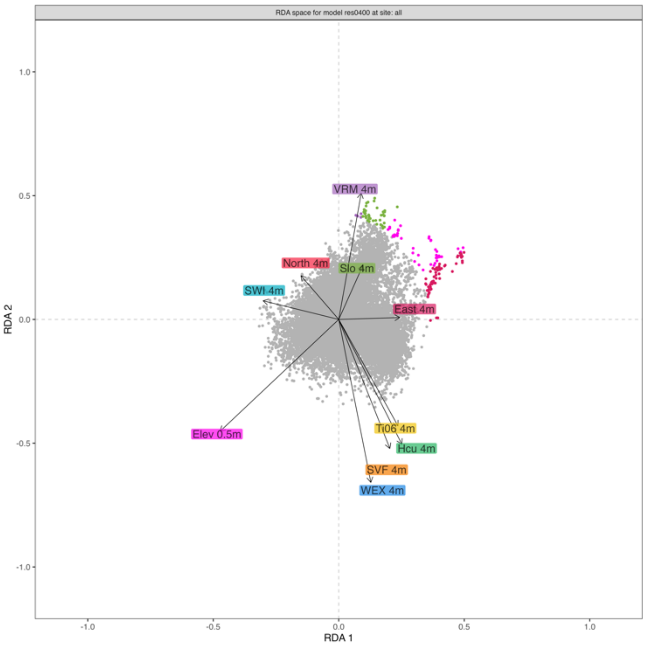

S13 e) Regional: v) *VS-8m*

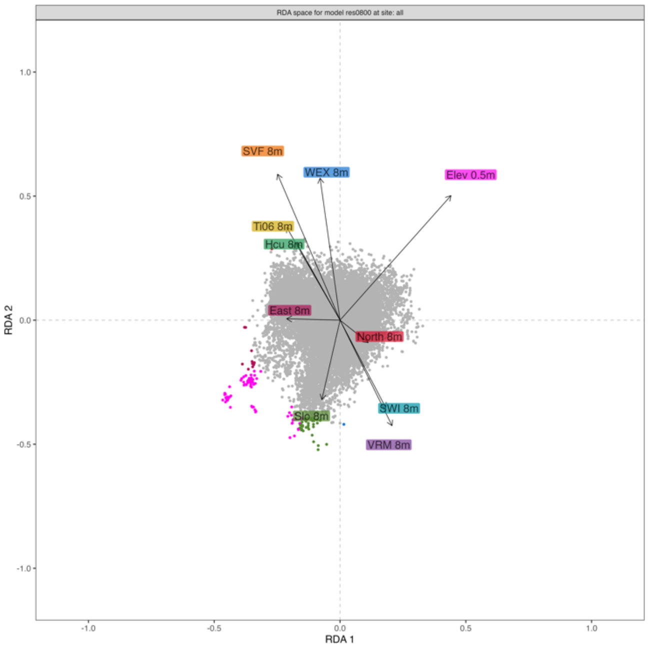

S13 e) Regional: vi) *VS-16m*

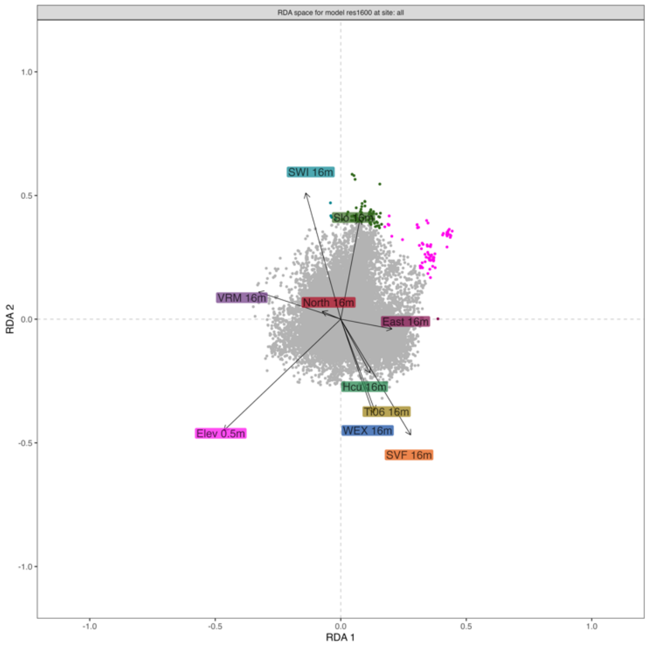

S13 e) Regional: vii) *VS-all*

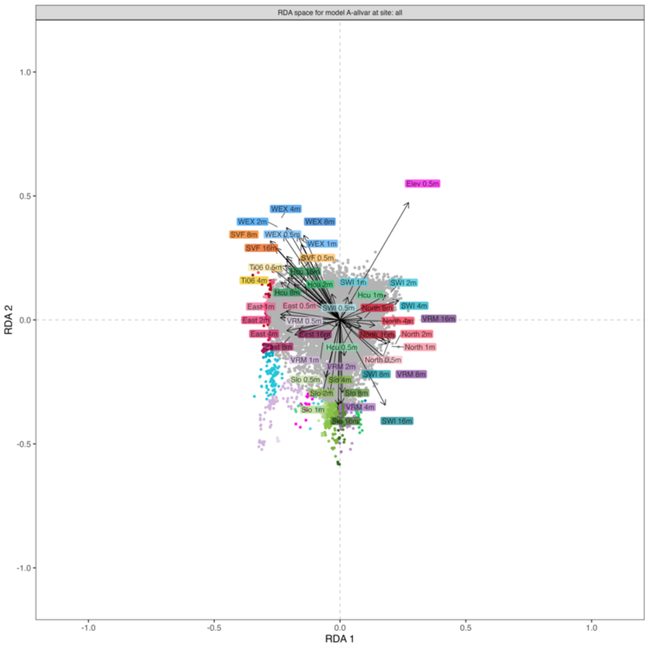

S13 e) Regional: viii) *VS-fwd*

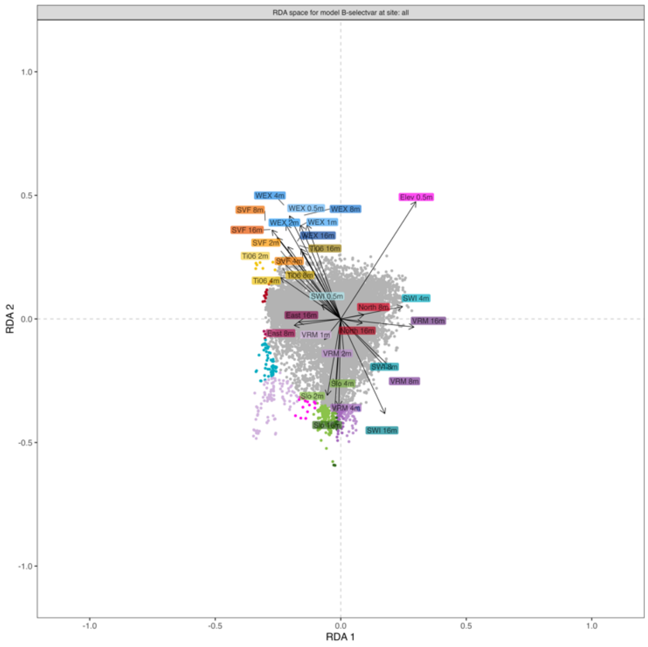

Supp Figure S14: *GEA model.*

Manhattan plots with the distribution of -$\log_{10}$(p-values) (y-axis) of loci along the eight chromosomes (x-axis), where p-values were allocated based on the loci's position in the *K*-retained redundancy analysis (RDA) axes. Loci are indicated in grey. Outlier loci are colour-coded by the environmental variable that resulted in the largest absolute scalar projection value based on the *K*-retained RDA axes. The Bonferroni threshold is indicated as the dashed red line (p=0.01/number of input SNPs), with the generic suggestive GWAS threshold of p=10^{-5} indicated as the dashed blue line. The RDA models were performed at the local **(a-d)** and regional **(e)** levels. The models were built with one of eight variable sets: i-vi) *VS-single* models with variables at a single spatial resolution (0.5, 1, 2, 4, 8, 16m), vii) *VS-all* models built with all variables at all spatial resolutions and viii) *VS-fwd* models built with variables selected using forward selection methods. All models included elevation at 0.5m spatial resolution. Analyses were performed with the *rda* function (*vegan* R package, v2.5-7).

S14 a) Essets:
K=3 retained RDA axes

S14 a) Essets: i) *VS-0.5m*

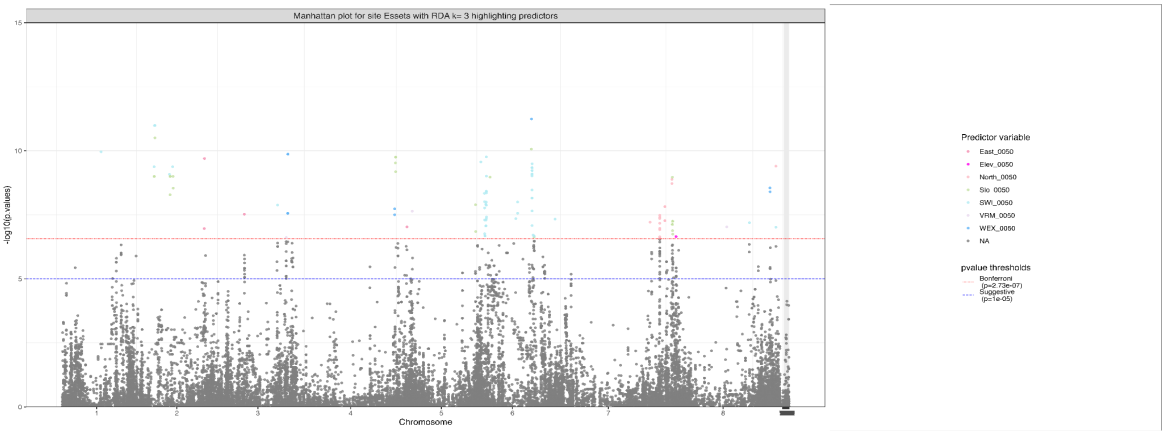

S14 a) Essets: ii) *VS-1m*

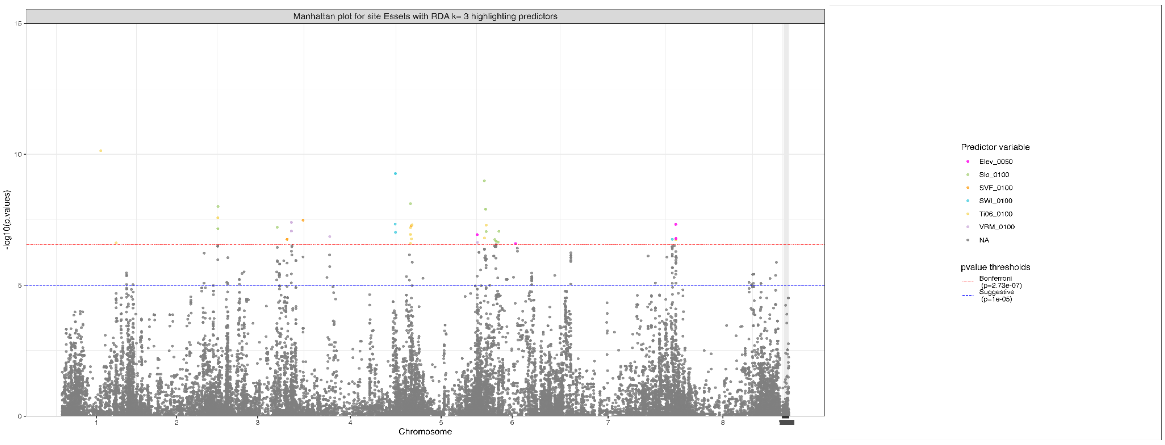

S14 a) Essets: iii) *VS-2m*

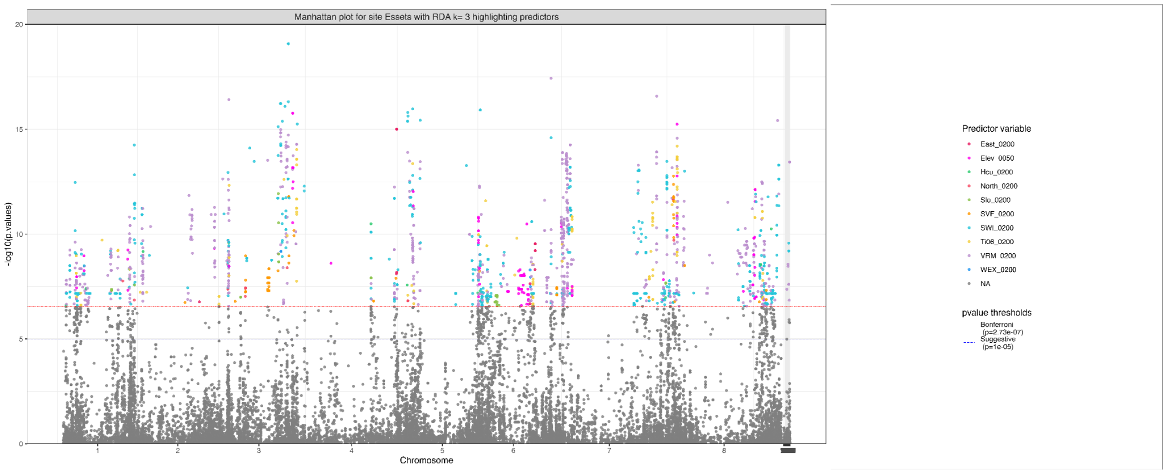

S14 a) Essets: iv) *VS-4m*

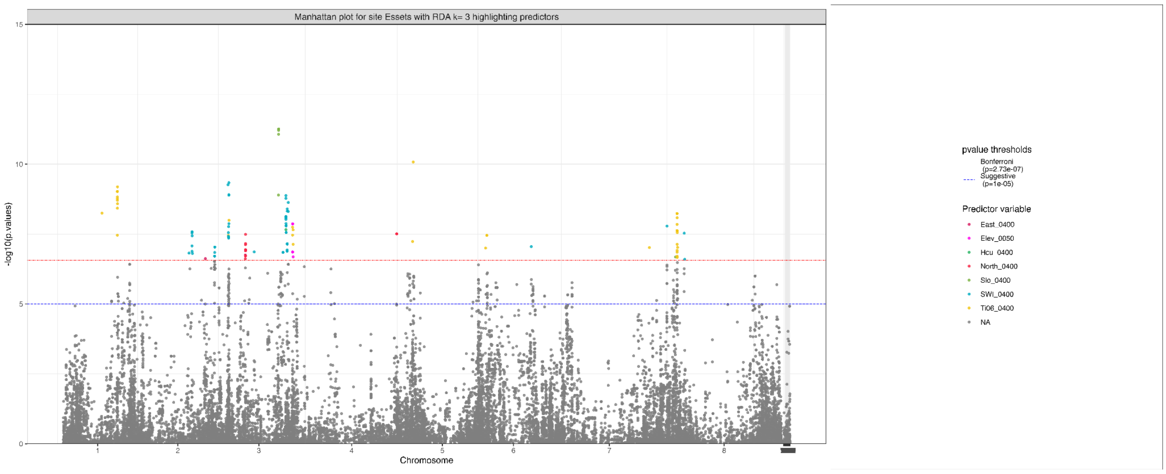

S14 a) Essets: v) *VS-8m*

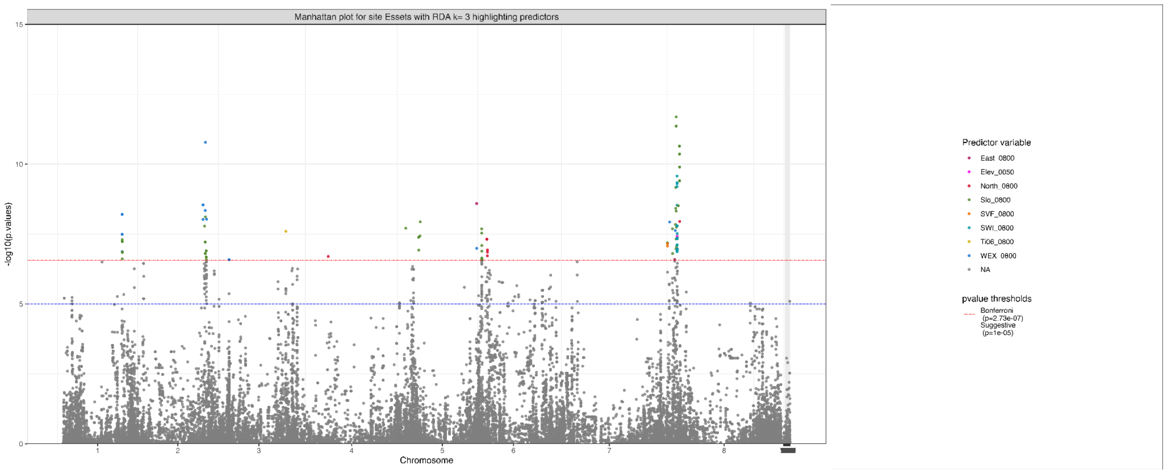

S14 a) Essets: vi) *VS-16m*

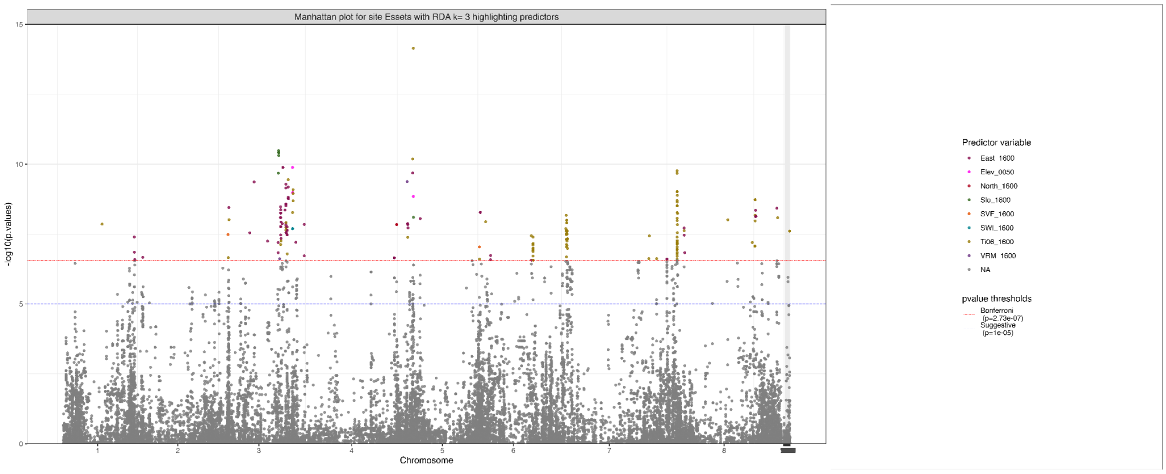

S14 a) Essets: vii) *VS-all*

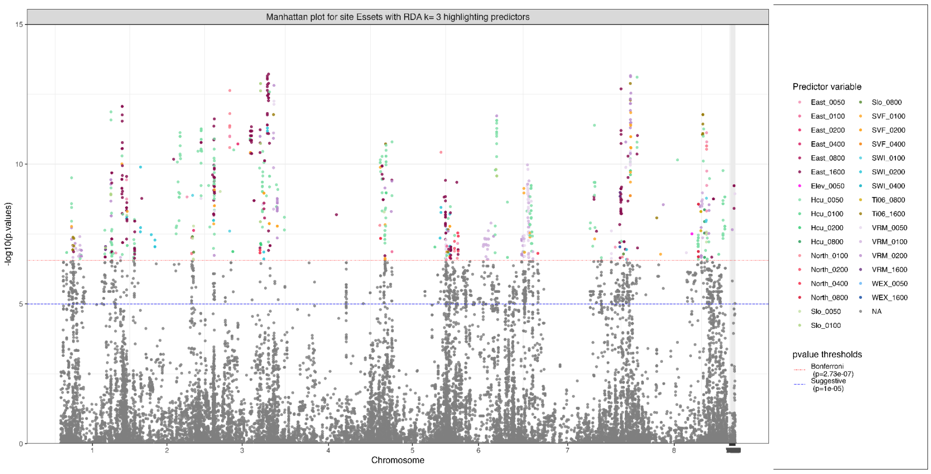

S14 a) Essets: viii) *VS-fwd*

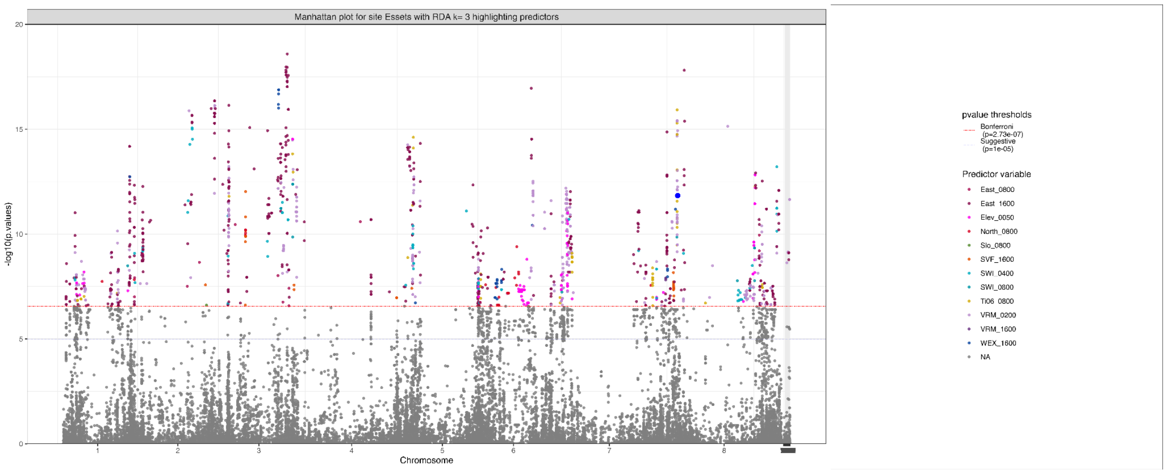

S14 b) Martinets:
K=2 retained RDA axes

S14 b) Martinets: i) *VS-0.5m*

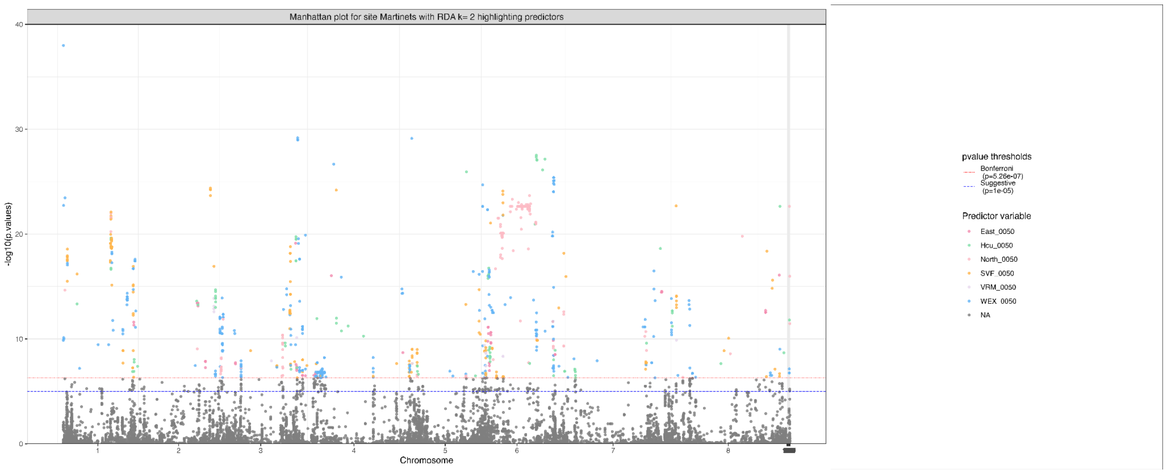

S14 b) Martinets: ii) *VS-1m*

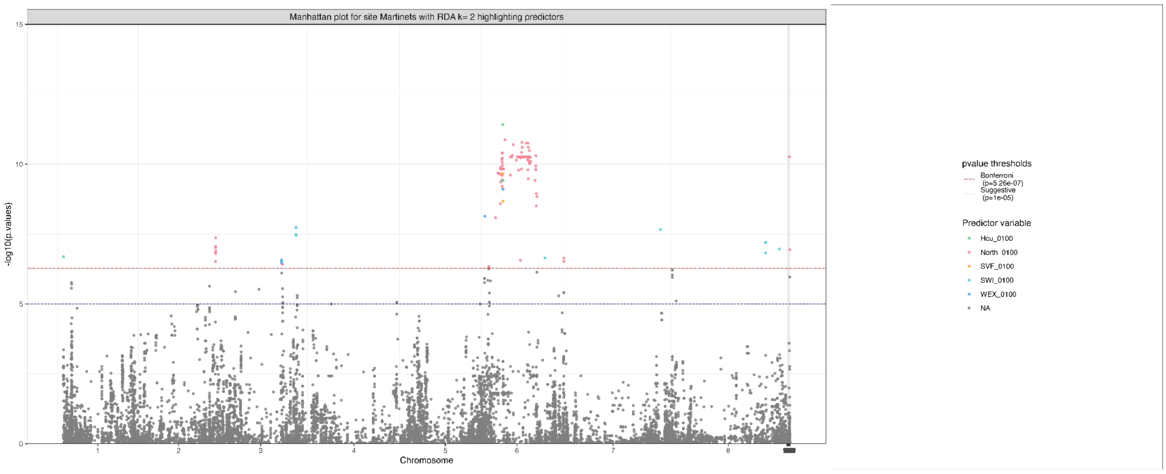

S14 b) Martinets: iii) *VS-2m*

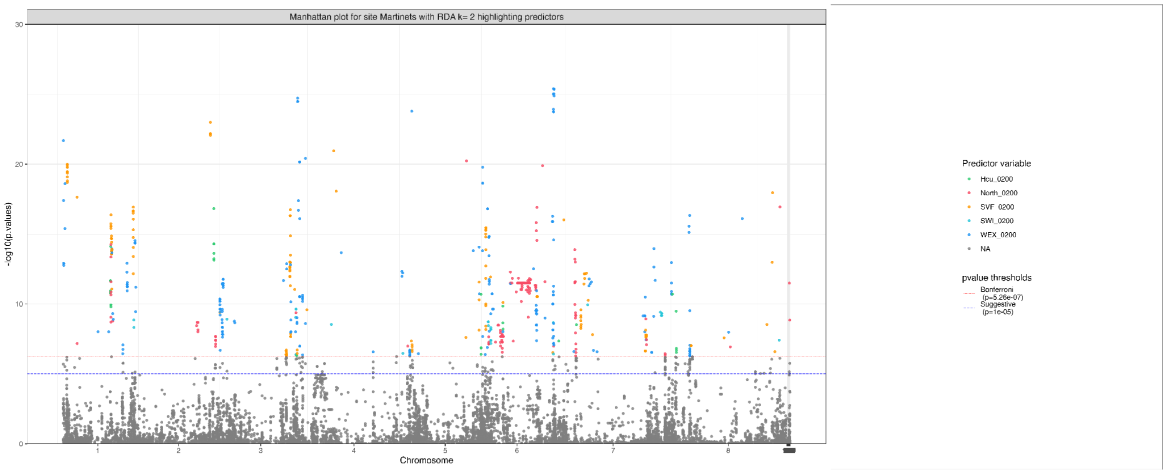

S14 b) Martinets: iv) *VS-4m*

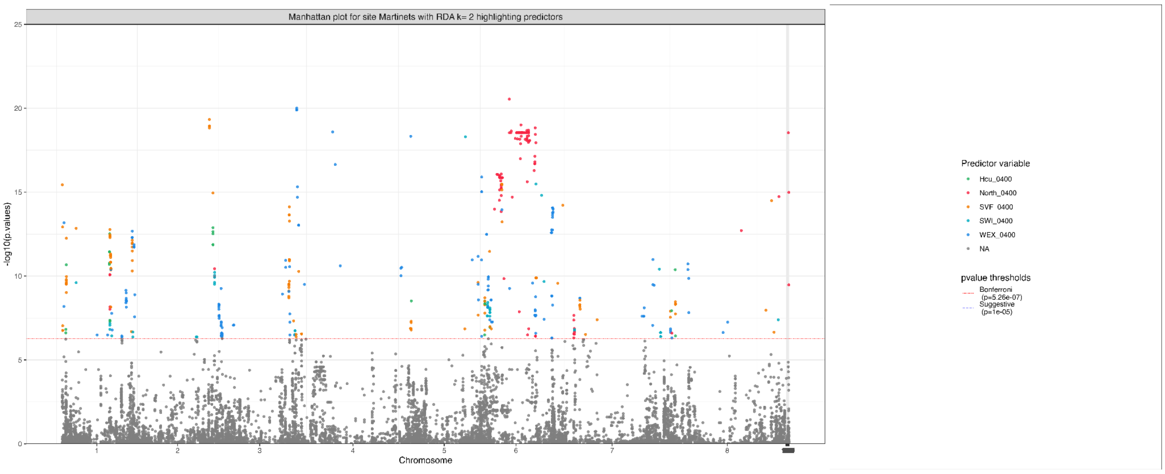

S14 b) Martinets: v) *VS-8m*

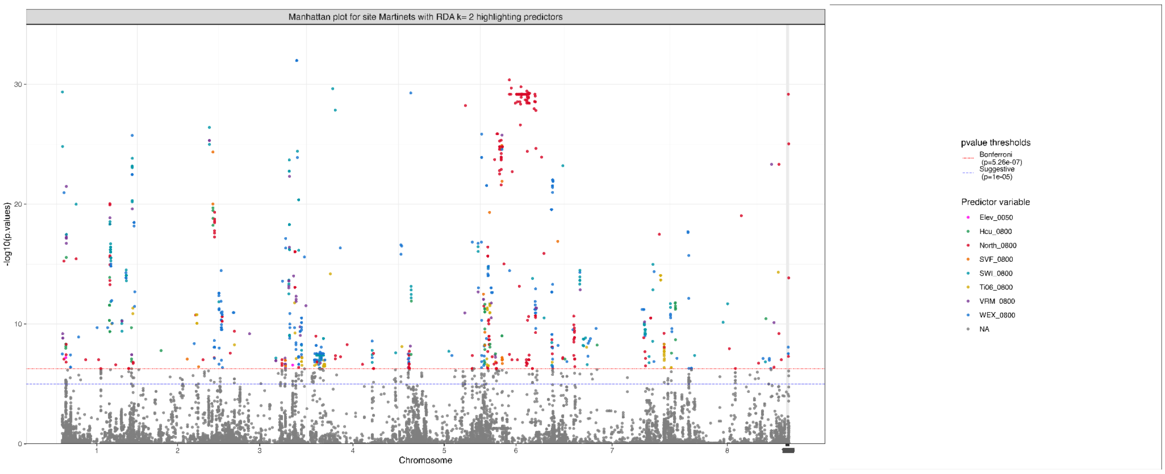

S14 b) Martinets: vi) *VS-16m*

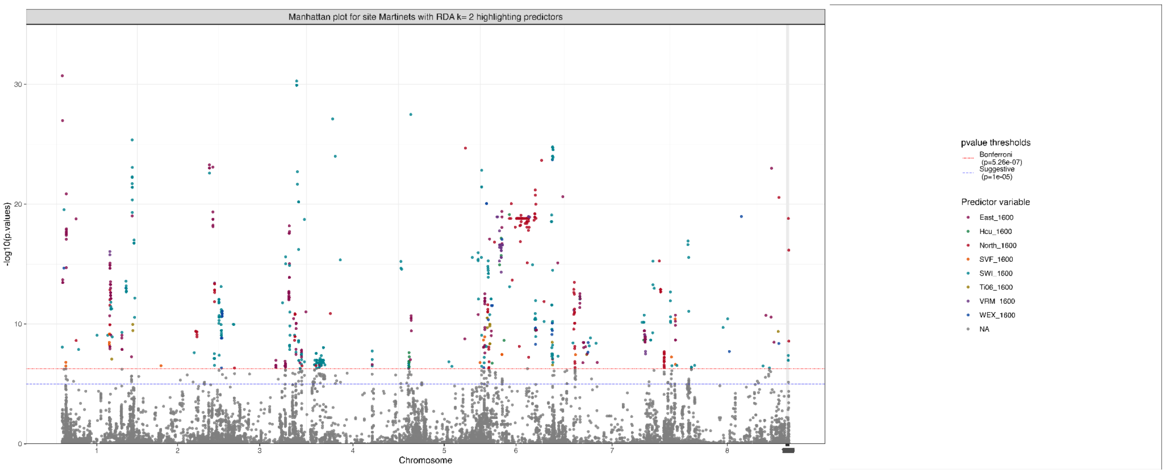

S14 b) Martinets: vii) *VS-all*

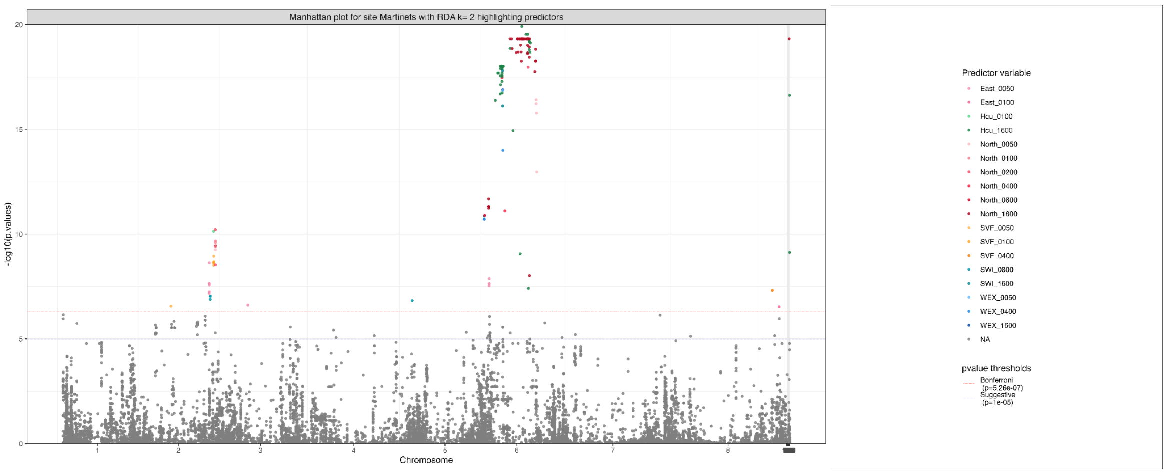

S14 b) Martinets: viii) *VS-fwd*

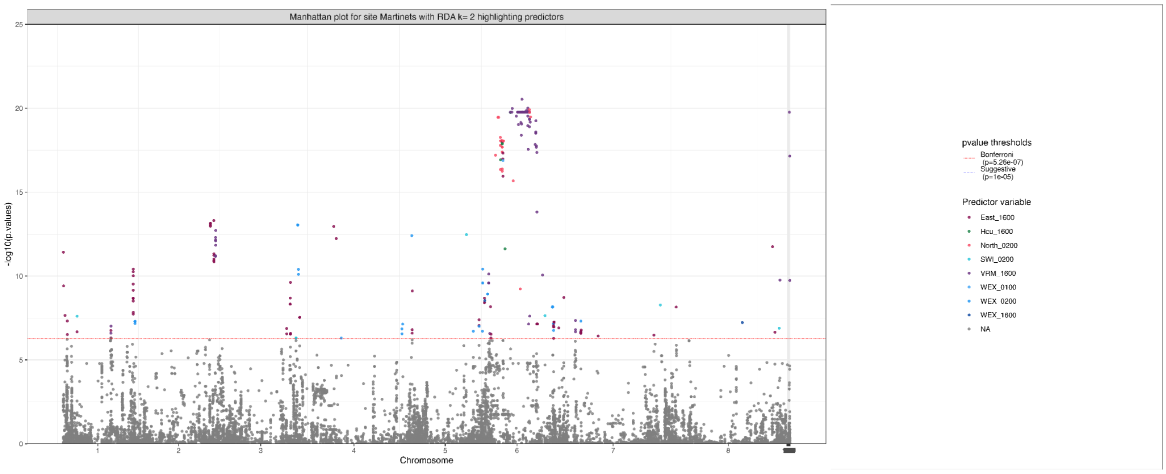

S14 c) Para:

K=2 retained RDA axes

S14 c) Para: i) *VS-0.5m*

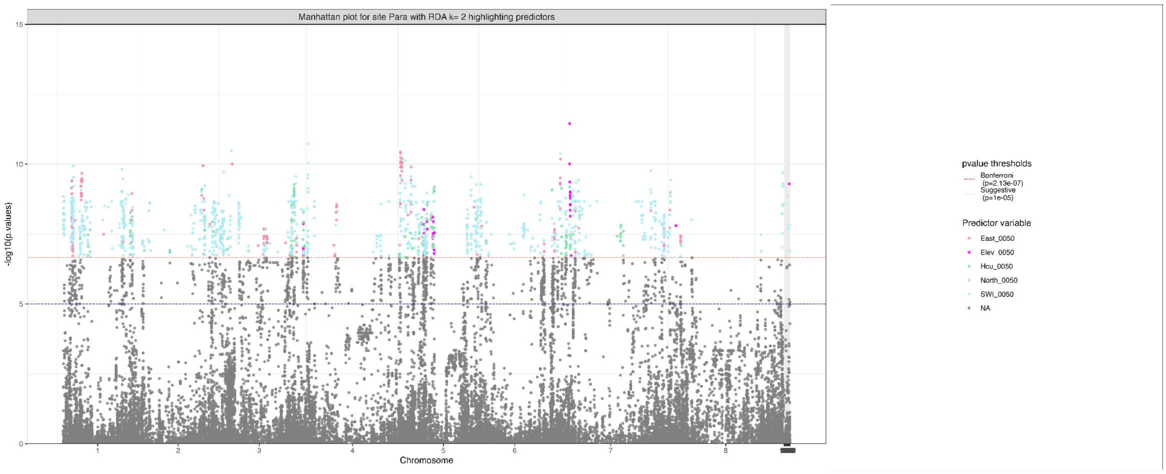

S14 c) Para: ii) *VS-1m*

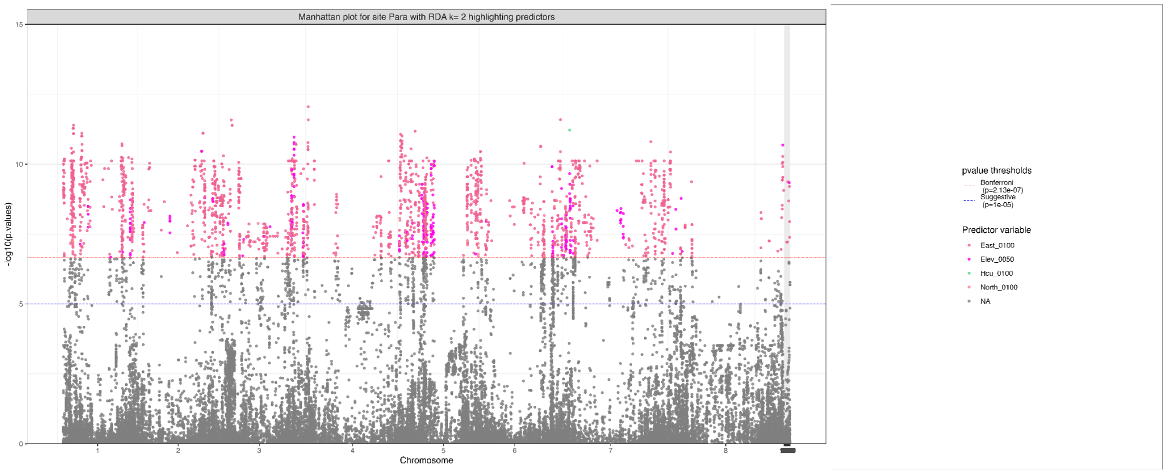

S14 c) Para: iii) *VS-2m*

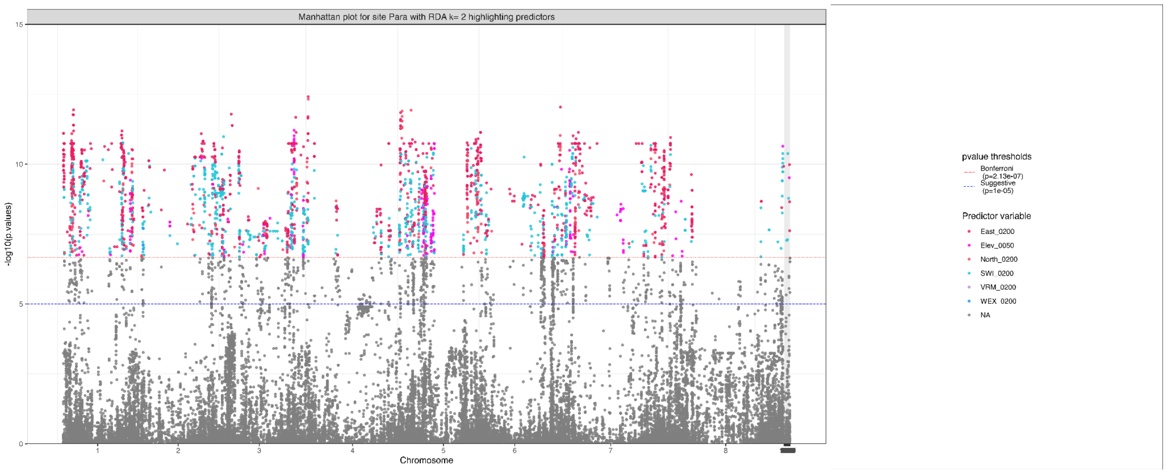

S14 c) Para: iv) *VS-4m*

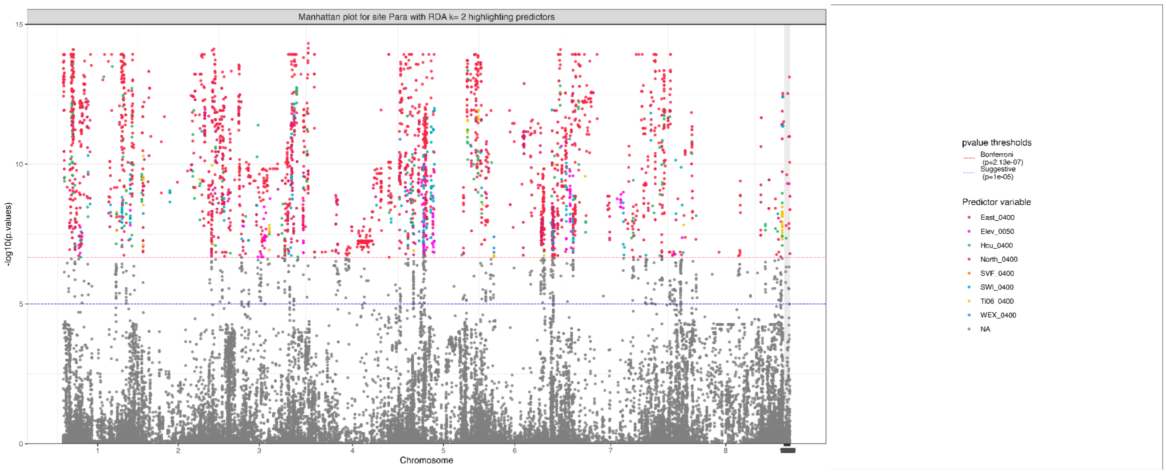

S14 c) Para: v) *VS-8m*

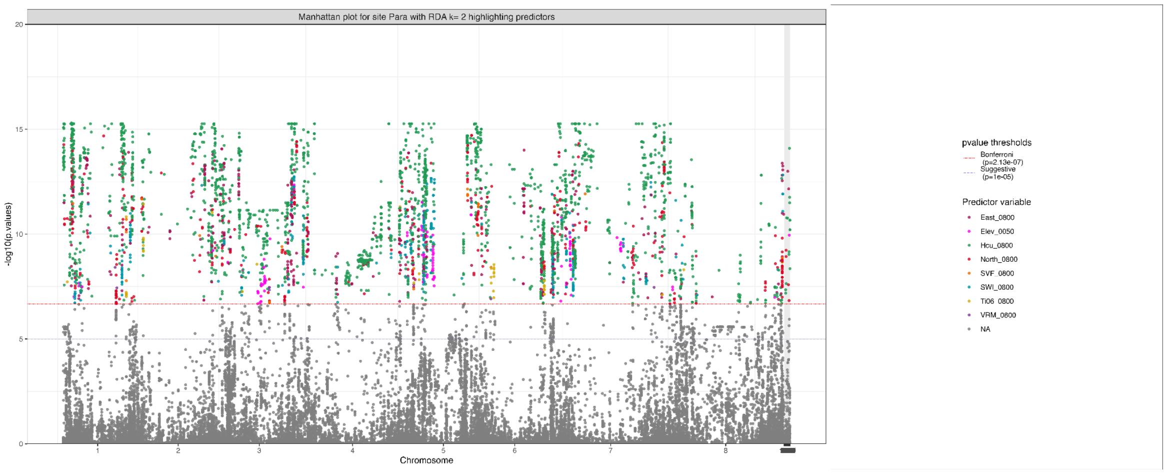

S14 c) Para: vi) *VS-16m*

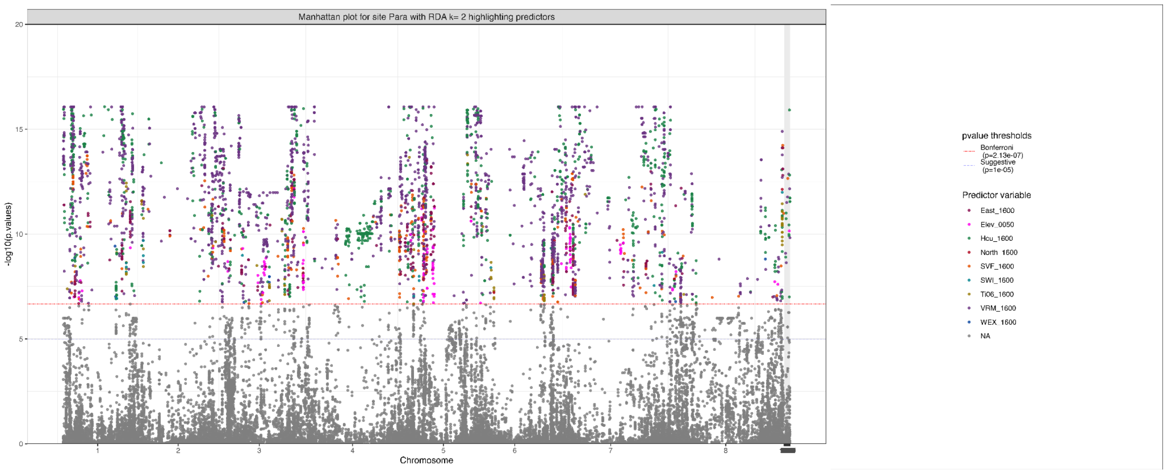

S14 c) Para: vii) *VS-all*

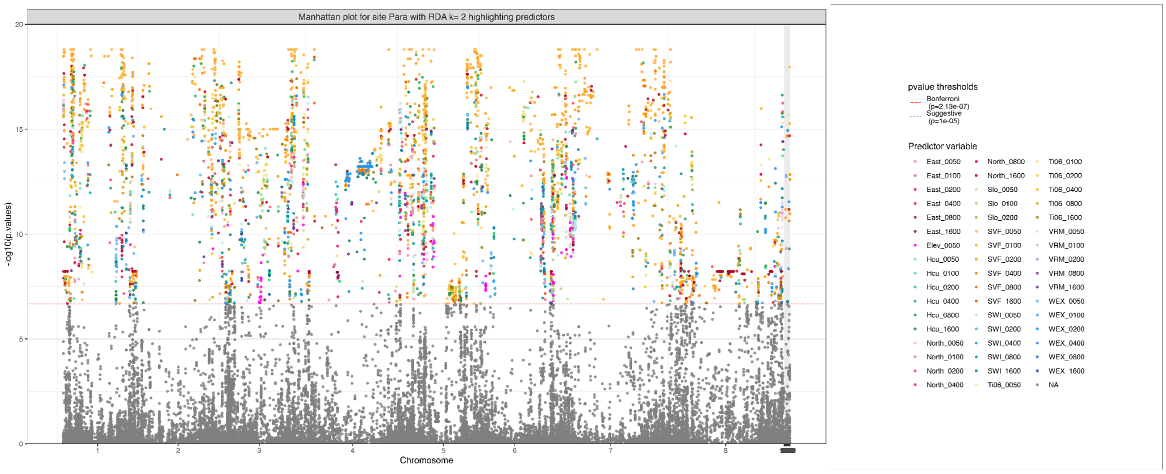

S14 c) Para: viii) *VS-fwd*

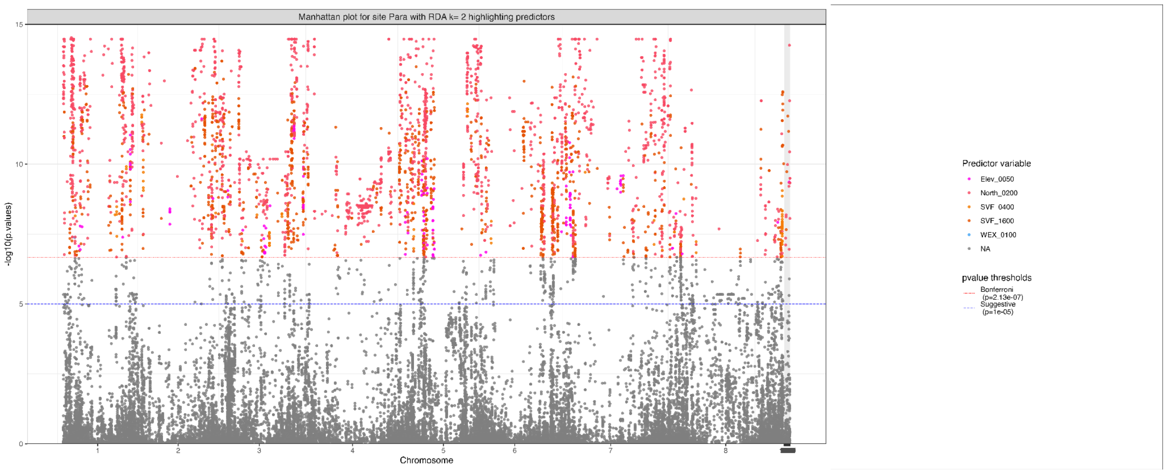

S14 d) Pierredar:

K=3 retained RDA axes

S14 d) Pierredar: i) *VS-0.5m*

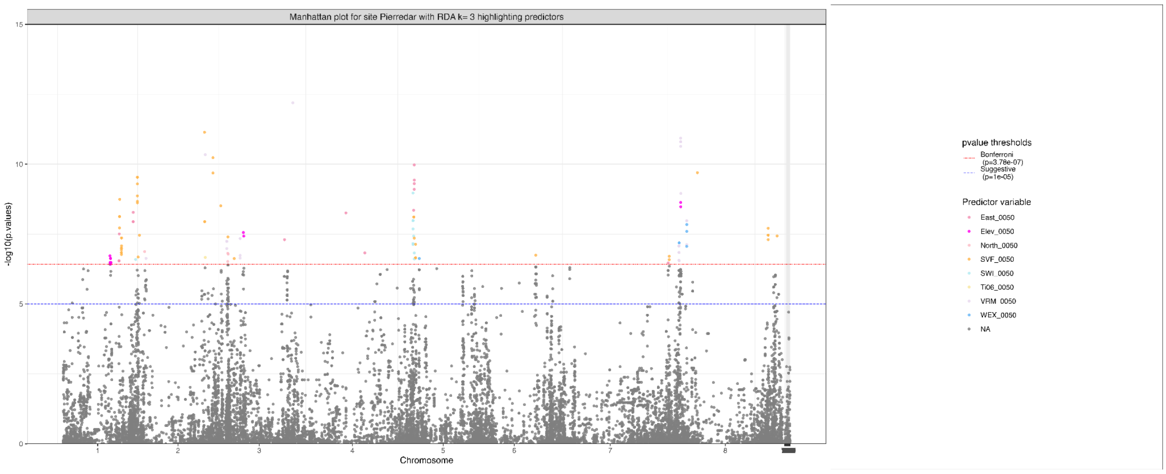

S14 d) Pierredar: ii) *VS-1m*

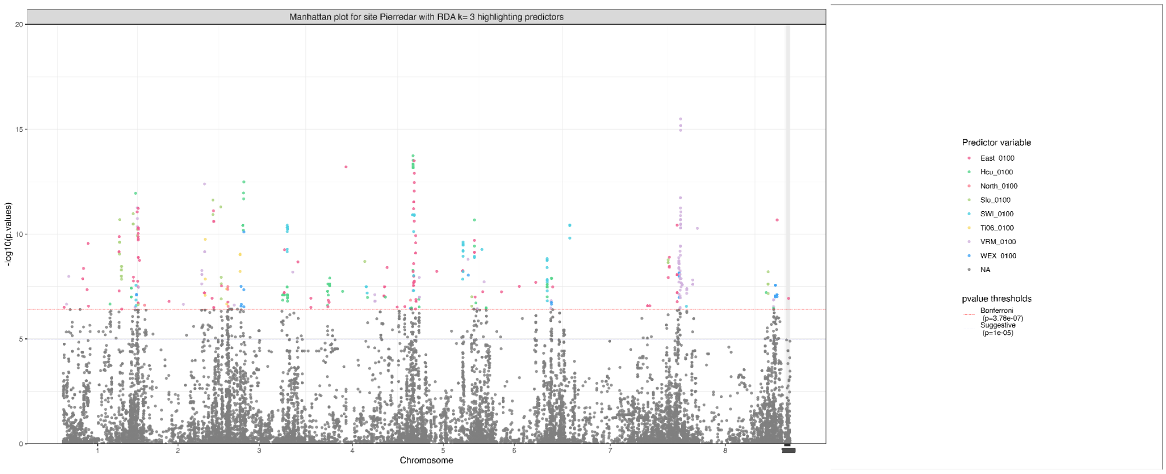

S14 d) Pierredar: iii) *VS-2m*

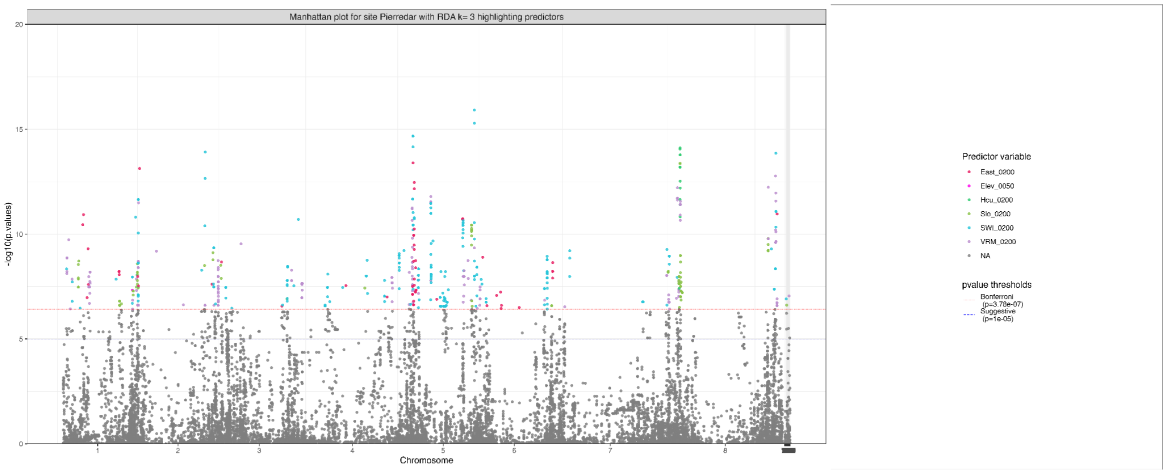

S14 d) Pierredar: iv) *VS-4m*

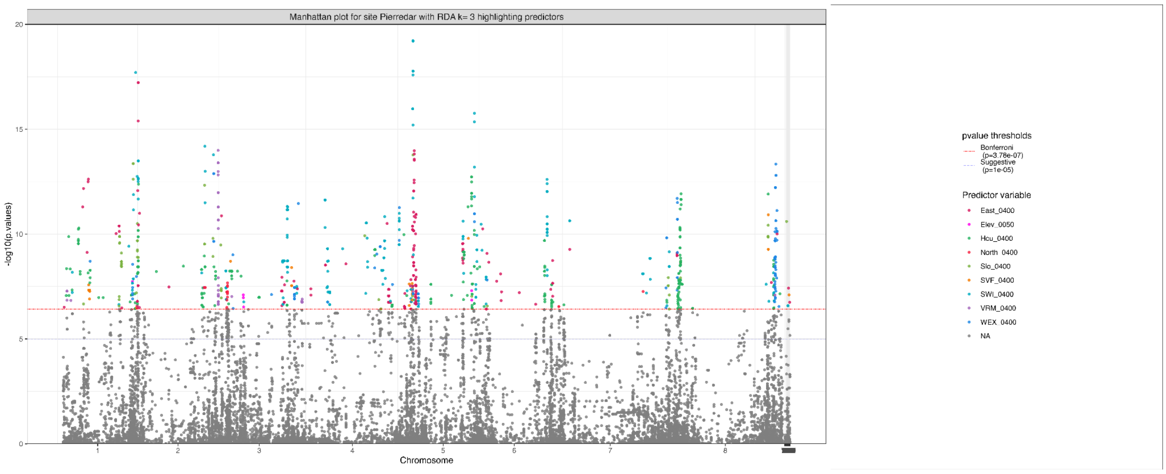

S14 d) Pierredar: v) *VS-8m*

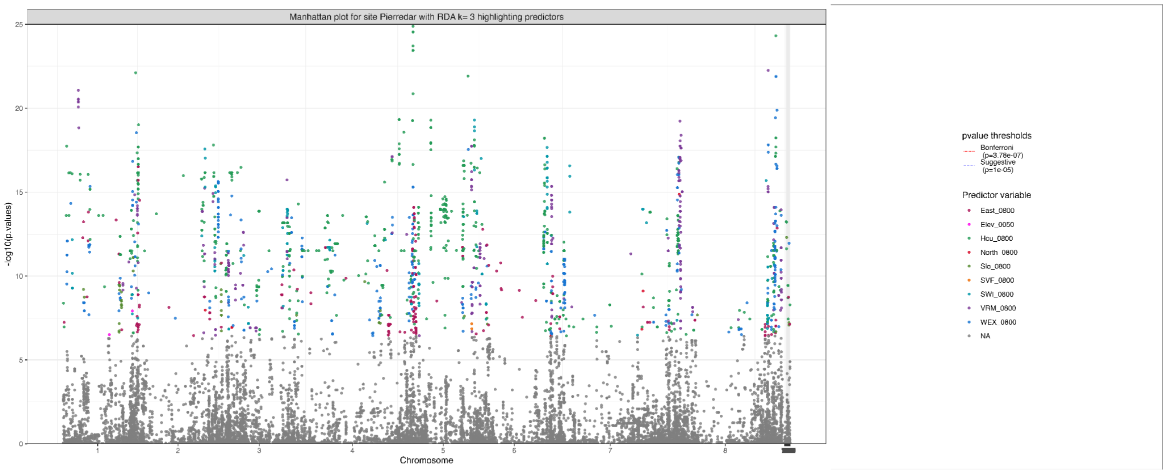

S14 d) Pierredar: vi) *VS-16m*

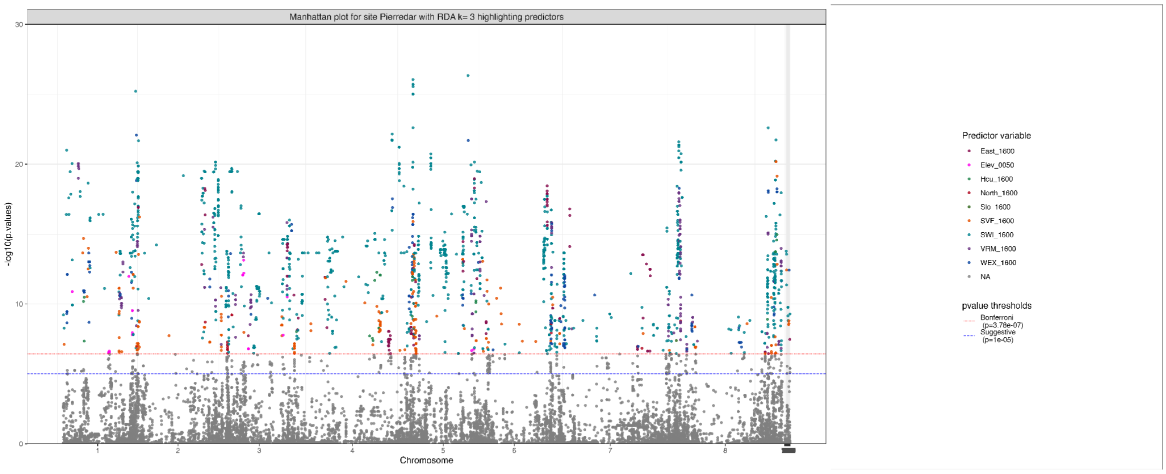

S14 d) Pierredar: vii) *VS-all*

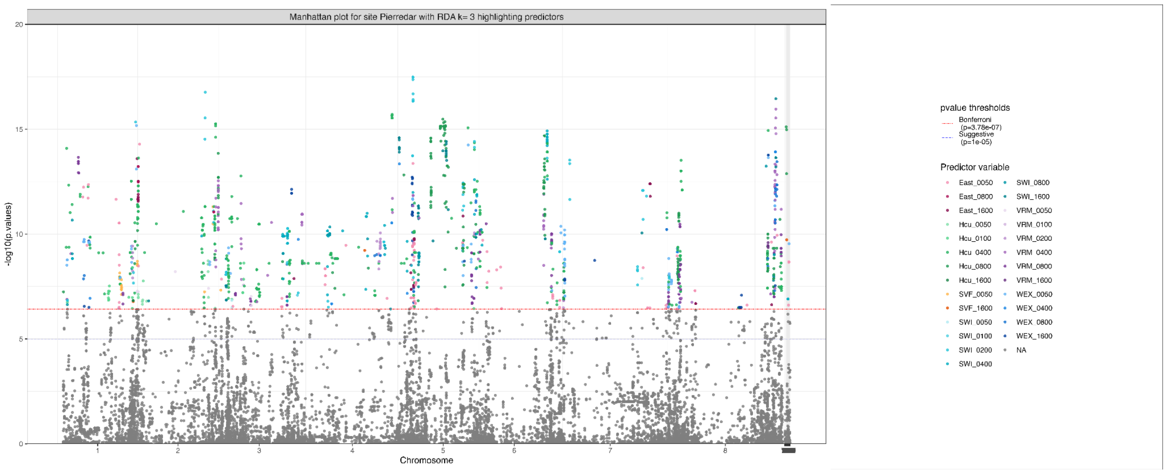

S14 d) Pierredar: viii) *VS-fwd*

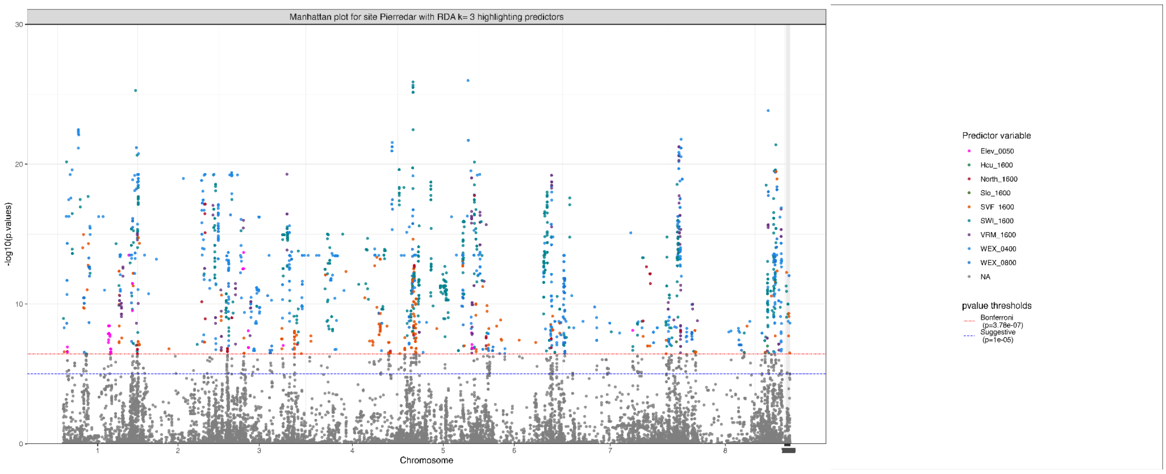

S14 e) Regional:
K=2 retained RDA axes

S14 e) Regional: i) *VS-0.5m*

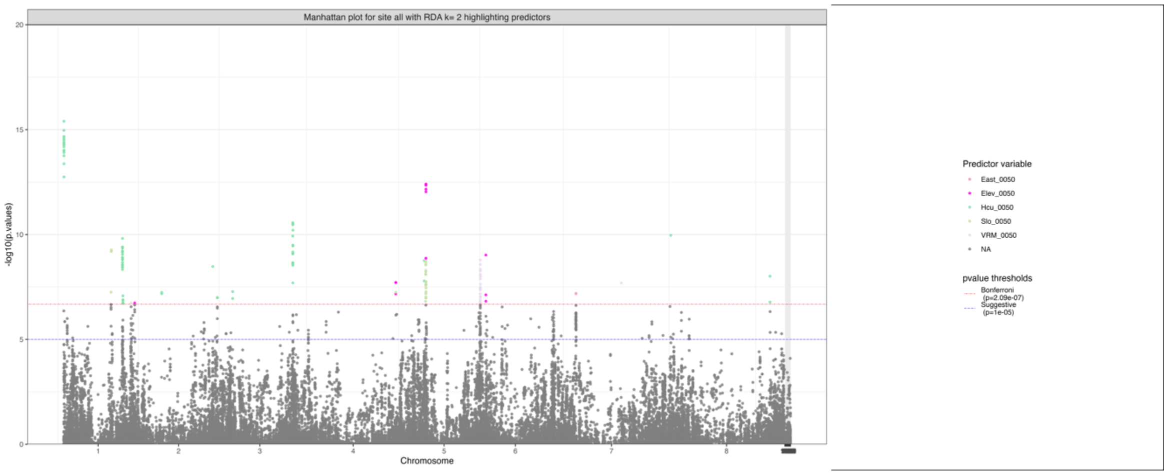

S14 e) Regional: ii) *VS-1m*

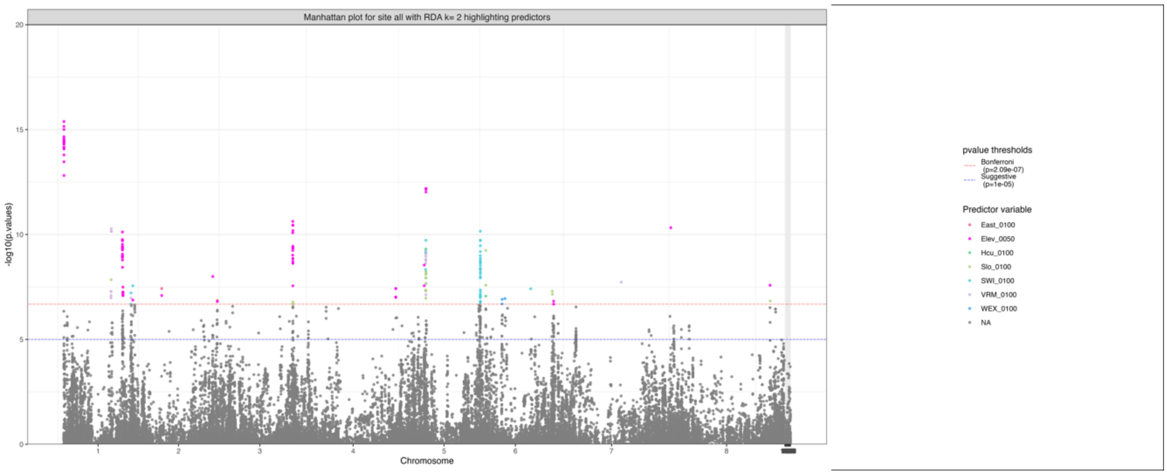

S14 e) Regional: iii) *VS-2m*

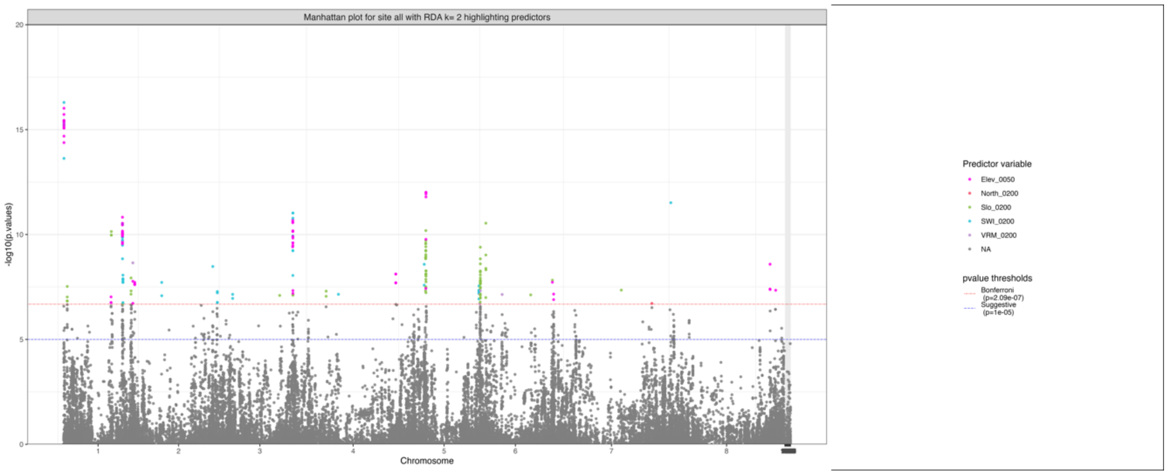

S14 e) Regional: iv) *VS-4m*

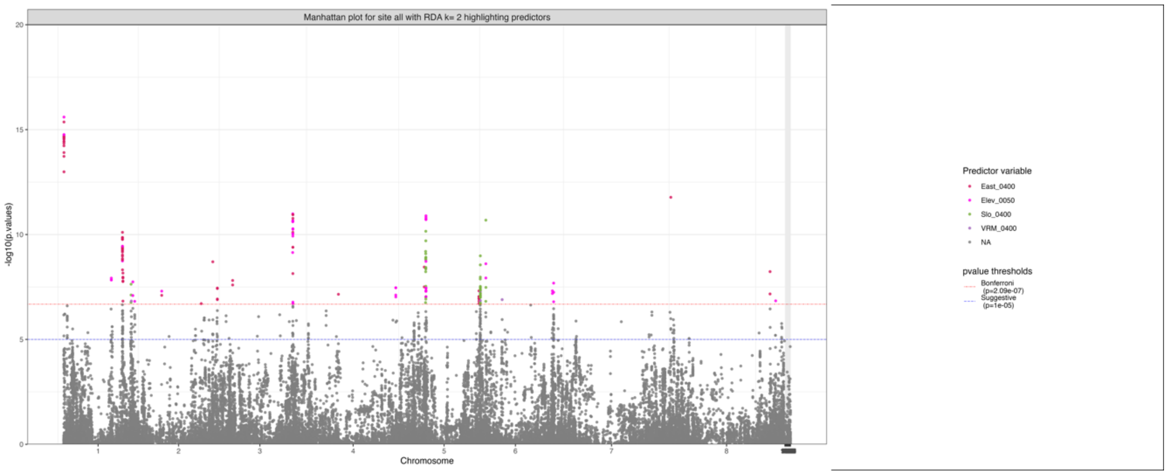

S14 e) Regional: v) *VS-8m*

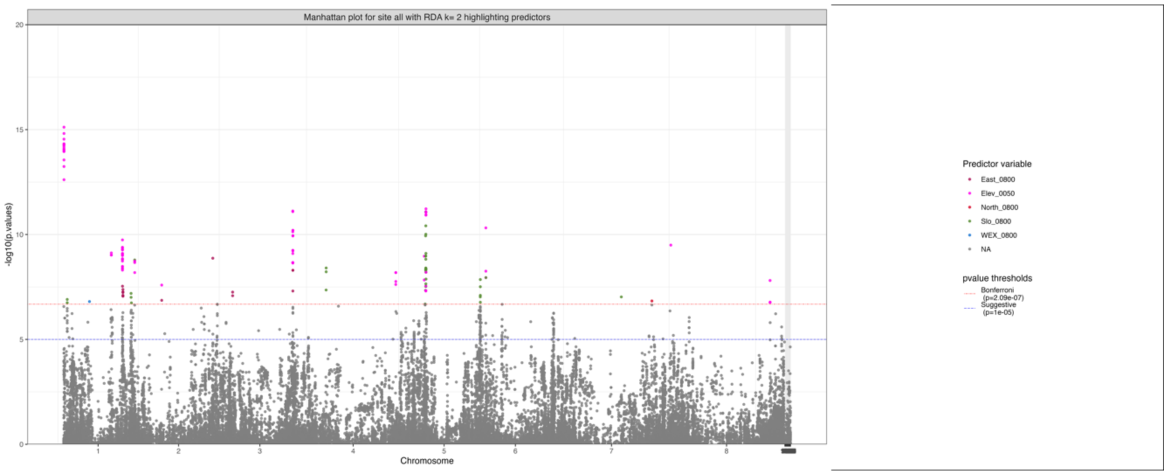

S14 e) Regional: vi) *VS-16m*

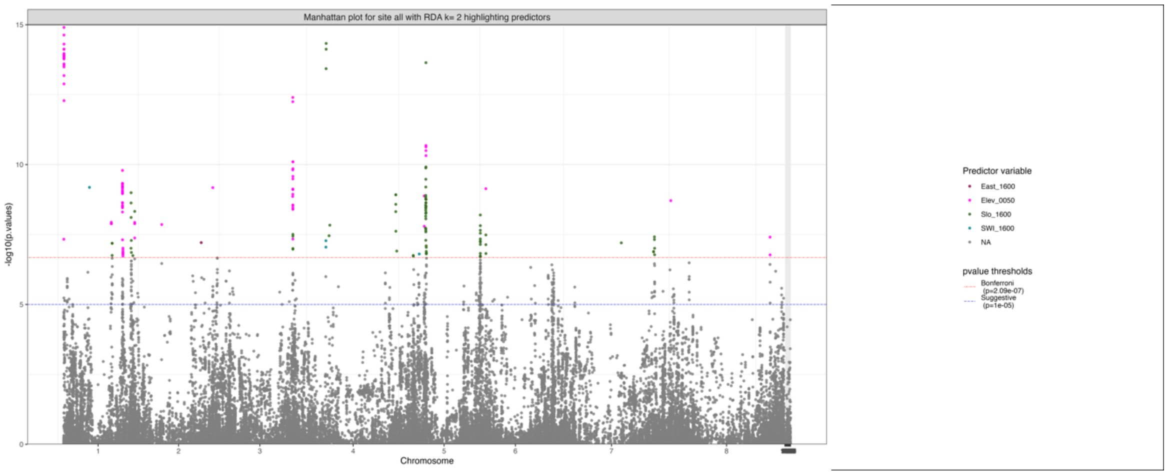

S14 e) Regional: vii) *VS-all*

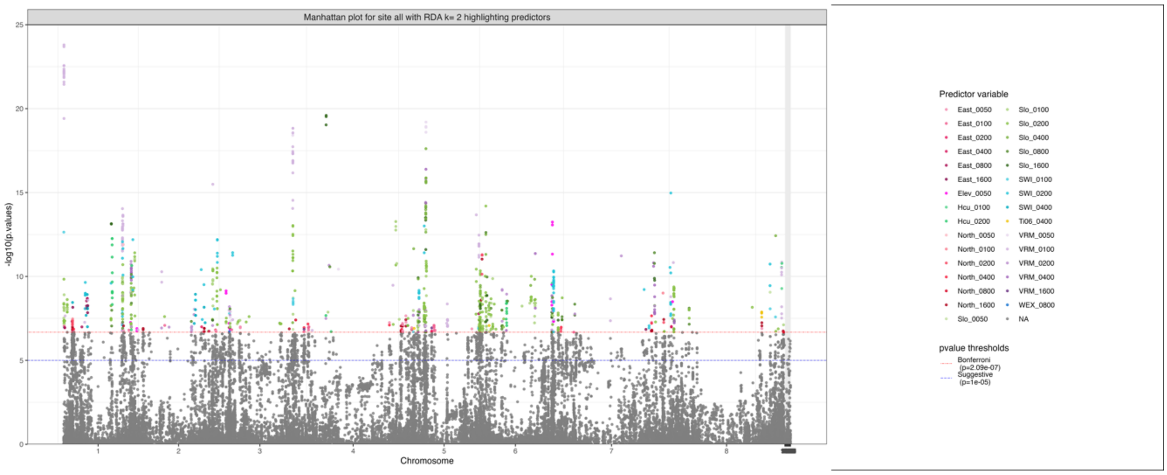

S14 e) Regional: viii) *VS-fwd*

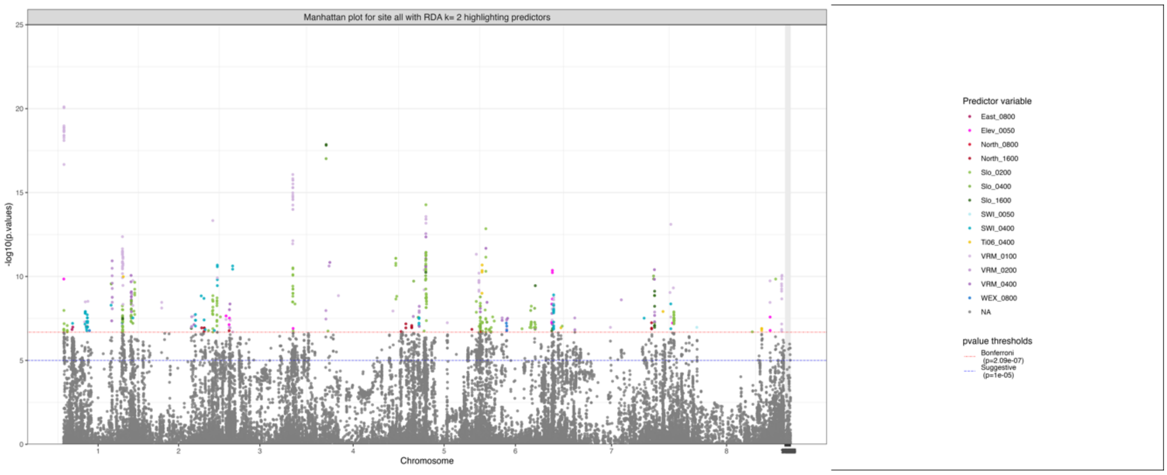

Supplementary Tables

Supp Table S1: Environmental variables.

Description and parameters for elevation and nine independent digital elevation model (DEM)-derived variables. Variables were selected due to their common usage in ecological studies. Aspect was converted into Eastness and Northness. Each variable was computed from LiDAR DEMs at spatial resolutions of 0.5, 1, 2, 4, 8 and 16m. Variables were uncorrelated at 0.5m spatial resolution with an $|r_s| \geq 0.8$ threshold (Spearman; **Supp Figure S3**).

	Variable	Abbv.	Description	Units	Parameters/Reference
**Primary attributes**	Elevation	Elev	Elevation obtained from DEMs.	m	Interpolated from a LiDAR point cloud at 0.5m resolution; generalized to multiple resolutions using B-spline wavelet transforms.
	Slope	SLO	*Morphometry.* Local morphometric terrain parameters; proxies for water flow, snow movements, erosion, solar radiation, etc. Eastness and Northness represent the sine and cosine of Aspect, respectively. Curvature is important for understanding variation in terrain.	Radian	Method = 9 parameter 2nd order polynomial; unit slope = radian; unit aspect = radian
	Eastness	EAST		Radian	
	Northness	NORTH		Radian	
	Plan curvature	HCU		*1/m*	

	Variable	Abbv.	Description	Units	Parameters/Reference
**Secondary attributes**	Vector ruggedness measure	VRM	*Morphometry.* Quantifies rugosity with less correlation to slope, indicating a combined variability in slope and aspect. Values 0= no terrain variation; 1= complete terrain variation	No unit	Radius = 1 cell
	SAGA Wetness Index	SWI	*Hydrology*. Modified version of Topographic Wetness Index (TWI): calculation of the slope and the modified catchment area. It predicts soil moisture for cells situated on the valley floor.	m^2/radian	Suction = 10; Type of Area = square root of TCa; Slope type = Catchment slope; Min Slope = 0; Offset Slope = 0.1; Slope Weighting = 1
**Secondary attributes**	Sky view factor	SVF	*Lighting*. Ratio of radiation received by a planar surface to the radiation emitted by the entire hemispheric environment	no unit	Max search radius = 10000; Method = sectors; Multi Scale Factor = 3; Number of sectors = 8
	Total solar radiation in June	TI6	*Lighting.* Sum of direct and diffuse insolation in summer	kWh/m^2	Solar Constant = 1367 W/m2; Shadow = flat; Location = constant lat; Latitude=46.407047°; Atmosphere method = Height of Atmosphere and Vapor Pressure; Atmosphere = 12000m; Time period = 30 days; Day step = 6 days; Hour range = 0-24h; Hour step = 1. Date range: 01-30 June 2015
	Wind Exposition Index	WEX	*Climate.* Average wind effect for all directions using an angular step. Values <1= shelter; >1= exposed.	No unit	Search distance = 300km; Angular step size=15.0deg; Acceleration = 1.5

Supp Table S2: Comparison of SNP set effects on GEA.

The effect of SNP datasets (i.e., intragenic, intergenic and whole genome) were compared in a genotype-environment association (GEA) using the forward selected Variable Sets (*VS-fwd*). The adjusted-R^2 for each GEA is provided to compare the performance of environmental factors for explaining variation in the SNP datasets. That intragenic SNPs have higher adjusted-R^2 values than intergenic and whole genome provides support that using only the intragenic SNP dataset as response variables in Variation Partitioning and GEAs

	**ESS**	**MAR**	**PAR**	**PIE**	**Regional**
Intragenic	0.212	0.335	0.183	0.302	0.110
Intergenic	0.207	0.315	0.178	0.296	0.105
Whole genome	0.208	0.319	0.179	0.297	0.105

Supp Table S3: Environmental variables.

Summary statistics (mean ±standard deviation) for the nine DEM-derived environmental variables at the middle resolution of 2m plus elevation at 0.5m. Values were calculated across the entirety of the four study sites (ESS, MAR, PAR, PIE) separately (local; **a-d**) and grouped (regional; **e**). Full details of parameters and descriptions of variables are provided in **Supplementary Table S1**.

	**a) ESS**	**b) MAR**	**c) PAR**	**d) PIE**	**e) Regional**
Environmental variables					
Elevation of study site (m a.s.l)	2265±103	2208±102	2055±130	2243±78	2198±133
Slope - SLO (radian)	0.41±0.2	0.46±0.2	0.51±0.2	0.41±0.2	0.44±0.2
Eastness - EAST (radian)	-0.08±0.6	0.32±0.6	0.43±0.5	-0.61±0.4	0.03±0.7
Northness - NORTH (radian)	0.53±0.5	0.47±0.6	0.62±0.4	0.46±0.5	0.52±0.5
Horizontal curvature - HCU (m^{-1})	-0.003±0.27	-0.001±0.31	-0.003±0.23	0±0.3	0±0.3
Vector Ruggedness Measure - VRM (no unit)	$3.3x10^{-3}$±$6x10^{-3}$	$4.3x10^{-3}$±$1x10^{-2}$	$4.6x10^{-3}$±9 $x10^{-3}$	$2.8x10^{-3}$±$6x10^{-3}$	$3.8x10^{-3}$±$8x10^{-3}$
SAGA Wetness Index - SWI (m^2 rad^{-1})	3.25±0.9	3.02±0.88	3.11±0.88	3.53±0.89	3.21±0.90
Sky View Factor - SVF (no unit)	0.9±0.1	0.89±0.1	0.89±0.1	0.89±0.1	0.89±0.6
Irradiance June - TI6 (kWh m^{-2})	224.7±30.4	224.8±29.7	213.3±35.6	232.9±34	223.7±33
Wind Exposure - WEX (no unit)	1.01±0.04	1.00±0.04	1.04±0.03	1.01±0.02	1.01±0.04

Supp Table S4: Forward selection.

Forward selected model results of environmental variables, from an original 55 variables (9 variables at 6 spatial resolutions, plus elevation at 0.5m) at the four sites (ESS, MAR, PAR, PIE) separately (local, **a-d)** and grouped (regional, **e).** In this predictive approach, variables were selected in a stepwise manner, first selecting those that optimise the maximum genetic variance observed in the intragenic data set (**Table 1**). The *ordi2Rstep* function of the vegan (v2.5-7) R package was used to build a forward model that maximises the adjusted-R^2 at each step, with stopping criteria of variable significance p<0.01 over 1000 permutations. For each site, variables are listed in the order of selection.

**a) ESS**	**Variable**	**Res. (m)**	**Adj-R^2**	**df**	**AIC**	***F*-value**	**Prob(>*F*)**
	Elev	0.5	0.05	1	635.9	4.36	0.002
	SVF	16	0.07	1	635.4	2.52	0.002
	VRM	4	0.09	1	634.8	2.43	0.002
	VRM	2	0.10	1	634.5	2.21	0.002
	SLO	8	0.12	1	634.1	2.23	0.002
	VRM	16	0.13	1	634.0	1.89	0.002
	NORTH	8	0.14	1	634.1	1.77	0.002
	TI6	8	0.15	1	633.9	1.88	0.002
	EAST	16	0.17	1	633.9	1.81	0.002
	WEX	16	0.18	1	633.7	1.89	0.002
	SWI	8	0.19	1	633.7	1.69	0.002
	SWI	4	0.20	1	633.7	1.63	0.006
	EAST	8	0.20	1	633.7	1.61	0.004
	HCU	16	0.21	1	633.7	1.56	0.006
	**All**		**0.37**				

**b) MAR**	**Variable**	**Res. (m)**	**Adj-R^2**	**df**	**AIC**	***F*-value**	**Prob(>*F*)**
	Elev	0.5	0.12	1	791.1	14.39	0.002
	WEX	16	0.16	1	787.8	5.27	0.002
	TI6	16	0.19	1	785.2	4.51	0.002
	VRM	16	0.21	1	783.4	3.64	0.002
	NORTH	2	0.24	1	781.6	3.63	0.002
	SWI	2	0.26	1	780.1	3.34	0.002
	WEX	2	0.27	1	778.5	3.31	0.002

	Variable	Res. (m)	Adj-R^2	df	AIC	*F*-value	Prob(>*F*)
	WEX	1	0.29	1	777.0	3.28	0.002
	EAST	16	0.31	1	776.1	2.66	0.002
	SLO	16	0.32	1	775.2	2.53	0.002
	EAST	8	0.32	1	775.3	1.73	0.002
	HCU	16	0.33	1	775.4	1.65	0.006
	TI6	4	0.33	1	775.5	1.63	0.01
	**All**		**0.40**				

c) PAR	Variable	Res. (m)	Adj-R^2	df	AIC	*F*-value	Prob(>*F*)
	NORTH	2	0.06	1	659.0	5.54	0.002
	Elev	0.5	0.10	1	657.1	3.77	0.002
	WEX	1	0.13	1	655.6	3.38	0.002
	SLO	8	0.15	1	655.0	2.46	0.002
	SVF	4	0.17	1	654.6	2.28	0.002
	SVF	16	0.18	1	654.6	1.77	0.002
	SLO	2	0.18	1	655.0	1.48	0.006
	**All**		**0.23**				

d) PIE	Variable	Res. (m)	Adj-R^2	df	AIC	*F*-value	Prob(>*F*)
	Elev	0.5	0.07	1	611.5	6.46	0.002
	NORTH	16	0.12	1	608.9	4.57	0.002
	SWI	16	0.15	1	607.2	3.55	0.002
	SLO	16	0.20	1	604.2	4.88	0.002
	VRM	16	0.23	1	603.0	2.99	0.002
	WEX	8	0.25	1	601.8	2.89	0.002
	TI6	16	0.26	1	601.1	2.42	0.002
	HCU	16	0.28	1	600.7	2.11	0.002
	SVF	16	0.29	1	600.6	1.89	0.002
	WEX	4	0.30	1	600.1	2.13	0.002
	**All**		**0.37**				

e) Regional	Variable	Res. (m)	Adj-R^2	df	AIC	*F*-value	Prob(>*F*)
	Elev	0.5	0.05	1	2753.3	16.45	0.002
	WEX	4	0.08	1	2744.4	10.97	0.002
	EAST	16	0.10	1	2739.9	6.49	0.002

TI6	2	0.11	1	2736.7	5.18	0.002
SWI	4	0.12	1	2734.5	4.17	0.002
SVF	16	0.12	1	2733.0	3.37	0.002
WEX	1	0.13	1	2731.7	3.23	0.002
WEX	0.5	0.14	1	2730.6	3.04	0.002
WEX	8	0.14	1	2729.6	2.93	0.002
EAST	8	0.15	1	2728.7	2.86	0.002
VRM	4	0.15	1	2727.7	2.84	0.002
TI6	16	0.16	1	2726.9	2.73	0.002
NORTH	16	0.16	1	2726.0	2.77	0.002
SWI	16	0.17	1	2725.3	2.58	0.002
SLO	16	0.17	1	2724.0	3.17	0.002
WEX	2	0.18	1	2723.4	2.40	0.002
SVF	4	0.18	1	2723.0	2.27	0.002
WEX	16	0.19	1	2722.6	2.29	0.002
SWI	0.5	0.19	1	2722.4	2.07	0.002
VRM	16	0.19	1	2722.3	1.97	0.002
VRM	8	0.19	1	2721.8	2.30	0.002
SVF	2	0.20	1	2721.7	1.94	0.002
SVF	8	0.20	1	2721.6	1.94	0.002
TI6	8	0.20	1	2721.4	2.10	0.002
SWI	8	0.21	1	2721.2	1.98	0.002
SLO	2	0.21	1	2721.2	1.82	0.002
NORTH	8	0.21	1	2721.2	1.83	0.002
SLO	4	0.21	1	2721.2	1.83	0.002
VRM	2	0.22	1	2721.2	1.80	0.002
TI6	4	0.22	1	2721.4	1.64	0.002
VRM	1	0.22	1	2721.4	1.74	0.002
**All**		**0.24**				

Supp Table S5: Variance partitioning.

Adjusted-R^2 values for the partitioning of variance (adjusted-R^2) of intragenic SNP data into the effects of population structure ('Pop structure'; represented as PCs obtained from PCA of LD-pruned intergenic SNPs; **Table 1; Supp Figure S7**), spatial geographic structure (represented as forward-selected Moran's eigenvector maps (MEMs); **Supp Figure S8; Supp Table S6**), local adaptation (represented by environmental variables), and elevation ('Elevation' at 0.5m spatial resolution), at the four sites (ESS, MAR, PAR, PIE) separately (local, **a-d**) and grouped (regional, **e**). The environment was assessed per variable set: i-vi) *VS-single* models, vii) *VS-all* models, and viii) *VS-fwd* models. Adjusted-R^2 values are provided for the full model as well as amount confounded ('Conf'd') and residuals ('Resid.'). Analyses were performed with the *varpart* function of the vegan (v2.5-7) R package. Venn diagrams in **Supp Figure S6**.

	**Full model**	**Pop structure**	**IBD**	**Elevation**	**IBE**	**Conf'd**	**Resid.**
**a) ESS**							
i) VS-0.5m	**0.254**	0.039	0.089	0.002	0.029	0.095	0.746
ii) VS-1m	**0.240**	0.039	0.074	0.003	0.016	0.108	0.760
iii) VS-2m	**0.260**	0.038	0.070	0.002	0.038	0.112	0.740
iv) VS-4m	**0.270**	0.040	0.069	0.004	0.048	0.109	0.730
v) VS-8m	**0.260**	0.037	0.073	0.001	0.036	0.113	0.740
vi) VS-16m	**0.270**	0.035	0.056	0.003	0.045	0.131	0.730
vii) VS-all	**0.328**	0.076	0.000	0.000	0.103	0.149	0.672
viii) VS-fwd	**0.295**	0.040	0.047	0.004	0.070	0.134	0.705
**b) MAR**							
i) VS-0.5m	**0.432**	0.044	0.090	0.004	0.010	0.284	0.568
ii) VS-1m	**0.434**	0.041	0.065	0.003	0.012	0.313	0.566
iii) VS-2m	**0.437**	0.038	0.057	0.002	0.015	0.325	0.563
iv) VS-4m	**0.436**	0.038	0.058	0.004	0.015	0.321	0.564
v) VS-8m	**0.433**	0.040	0.062	0.006	0.011	0.314	0.567
vi) VS-16m	**0.426**	0.046	0.062	0.002	0.005	0.311	0.574
vii) VS-all	**0.461**	0.056	0.018	0.003	0.040	0.344	0.539
viii) VS-fwd	**0.435**	0.041	0.043	0.001	0.014	0.336	0.565

**c) PAR**							
i) VS-0.5m	**0.292**	0.089	0.020	0.004	0.015	0.164	0.708
ii) VS-1m	**0.296**	0.088	0.014	0.004	0.020	0.170	0.704
iii) VS-2m	**0.302**	0.094	0.015	0.005	0.026	0.162	0.698
iv) VS-4m	**0.304**	0.087	0.010	0.001	0.027	0.179	0.696
v) VS-8m	**0.306**	0.088	0.011	0.002	0.029	0.176	0.694
vi) VS-16m	**0.296**	0.090	0.017	0.004	0.020	0.165	0.704
vii) VS-all	**0.407**	0.175	0.000	0.000	0.130	0.102	0.593
viii) VS-fwd	**0.296**	0.088	0.019	0.003	0.019	0.167	0.704
**d) PIE**							
i) VS-0.5m	**0.343**	0.042	0.127	0.011	0.015	0.148	0.657
ii) VS-1m	**0.358**	0.040	0.101	0.006	0.030	0.181	0.642
iii) VS-2m	**0.376**	0.045	0.102	0.009	0.049	0.171	0.624
iv) VS-4m	**0.364**	0.042	0.092	0.007	0.037	0.186	0.636
v) VS-8m	**0.377**	0.039	0.065	0.004	0.050	0.219	0.623
vi) VS-16m	**0.380**	0.041	0.043	0.003	0.053	0.240	0.620
vii) VS-all	**0.449**	0.009	0.038	0.000	0.122	0.280	0.551
viii) VS-fwd	**0.398**	0.041	0.040	0.005	0.071	0.241	0.602
**e) Regional**							
i) VS-0.5m	**0.349**	0.012	0.160	0.003	0.005	0.169	0.651
ii) VS-1m	**0.351**	0.013	0.160	0.002	0.007	0.169	0.649
iii) VS-2m	**0.354**	0.013	0.159	0.003	0.010	0.169	0.646
iv) VS-4m	**0.355**	0.012	0.157	0.002	0.011	0.173	0.645
v) VS-8m	**0.354**	0.013	0.154	0.003	0.010	0.174	0.646
vi) VS-16m	**0.354**	0.012	0.156	0.004	0.010	0.172	0.646
vii) VS-all	**0.387**	0.013	0.108	0.003	0.044	0.219	0.613
viii) VS-fwd	**0.375**	0.013	0.117	0.003	0.031	0.211	0.625

Supp Table S6: ***Geography***.

Forward selection results of Moran's Eigenvector Maps (MEMs) calculated to assess plant spatial connectivity at the four sites (ESS, MAR, PAR, PIE) separately (local, **a-d**) and grouped (regional, **e**). The geographic (X,Y) coordinates of sampled plants were used to triangulate the neighbours of each plant, which was used to calculate weightings to make MEMs. Moran's *I* was calculated for each eigenvector of the weighting matrix, using 999 permutations to calculate a p-value, where MEMs with a p-value <0.01 were retained. A stepwise forward selection procedure was then used to select the MEMs that best explained the neutral genetic variance (intergenic SNPs pruned for linkage disequilibrium, **Table 1**), where the cut-off threshold was determined using a redundancy analysis (RDA). Note that only every second MEM was used in the forward selection procedure due to computational limitations. See Dray et al (2012) for full details. Analyses were performed with the spdep package (v1.2-1) to calculate nearest neighbours, the spacemakeR package (v0.0-5) to calculate MEMs, the vegan package (v2.5-7) to calculate the R_{adj} threshold for the forward selection, and the *forward.sel* function of the adespatial package (v0.3-14) to perform the stepwise forward selection. For each site, variables are listed in the order of selection.

MEM	R^2	Cumulative R^2	Cumulative adjusted-R^2	F	p-value
**a) ESS**					
MEM 1	0.04	0.04	0.02	2.58	0.001
MEM 2	0.03	0.07	0.04	2.31	0.001
MEM 6	0.03	0.10	0.06	2.11	0.001
MEM 5	0.03	0.13	0.07	2.13	0.001
MEM 3	0.03	0.15	0.09	2.01	0.001
MEM 7	0.02	0.18	0.10	1.82	0.001
MEM 4	0.02	0.20	0.11	1.75	0.001
MEM 8	0.02	0.22	0.12	1.71	0.001
MEM 14	0.02	0.24	0.13	1.48	0.001
MEM 10	0.02	0.26	0.13	1.44	0.005
MEM 11	0.02	0.28	0.14	1.40	0.002
**b) MAR**					
MEM 4	0.05	0.05	0.04	4.46	0.001
MEM 2	0.04	0.09	0.07	4.09	0.001

MEM	R^2	Cumulative R^2	Cumulative adjusted-R^2	F	p-value
MEM 3	0.03	0.12	0.09	3.34	0.001
MEM 1	0.03	0.15	0.11	3.24	0.001
MEM 7	0.03	0.17	0.13	2.92	0.001
MEM 5	0.02	0.20	0.15	2.73	0.001
MEM 8	0.02	0.22	0.16	2.50	0.001
MEM 9	0.02	0.24	0.17	2.49	0.001
MEM 6	0.02	0.26	0.19	2.37	0.001
MEM 32	0.01	0.28	0.19	1.44	0.003
MEM 13	0.01	0.29	0.19	1.44	0.002
MEM 78	0.01	0.30	0.20	1.43	0.002
MEM 85	0.01	0.31	0.20	1.38	0.009
MEM 16	0.01	0.32	0.21	1.37	0.003
MEM 30	0.01	0.33	0.21	1.33	0.008
MEM 10	0.01	0.35	0.21	1.33	0.027
**c) PAR**					
MEM 1	0.06	0.06	0.04	4.12	0.001
MEM 3	0.03	0.09	0.06	2.22	0.001
MEM 2	0.02	0.11	0.07	1.64	0.001
MEM 4	0.02	0.13	0.08	1.63	0.001
MEM 8	0.02	0.15	0.09	1.45	0.003
MEM 7	0.02	0.17	0.09	1.42	0.002
**d) PIE**					
MEM 2	0.05	0.05	0.04	3.62	0.001
MEM 1	0.05	0.10	0.07	3.72	0.001
MEM 3	0.04	0.14	0.10	3.04	0.001
MEM 6	0.04	0.18	0.13	2.80	0.001
MEM 5	0.03	0.21	0.15	2.80	0.001

MEM	R^2	Cumulative R^2	Cumulative adjusted-R^2	F	p-value
MEM 7	0.03	0.25	0.17	2.82	0.001
MEM 9	0.03	0.28	0.19	2.35	0.001
MEM 8	0.03	0.30	0.21	2.15	0.001
**e) Regional**					
MEM 2	0.03	0.03	0.02	8.43	0.001
MEM 1	0.02	0.05	0.05	7.70	0.001
MEM 4	0.02	0.07	0.06	5.74	0.001
MEM 5	0.02	0.09	0.07	5.70	0.001
MEM 7	0.02	0.10	0.09	5.31	0.001
MEM 6	0.02	0.12	0.10	5.36	0.001
MEM 3	0.02	0.13	0.11	5.15	0.001
MEM 9	0.02	0.15	0.13	5.21	0.001
MEM 10	0.01	0.16	0.14	4.35	0.001
MEM 11	0.01	0.17	0.14	3.48	0.001
MEM 8	0.01	0.18	0.15	3.46	0.001
MEM 13	0.01	0.19	0.16	3.27	0.001
MEM 15	0.01	0.20	0.16	2.97	0.001
MEM 12	0.01	0.21	0.17	2.99	0.001
MEM 30	0.01	0.21	0.17	2.40	0.001
MEM 38	0.01	0.22	0.18	2.38	0.001
MEM 25	0.01	0.23	0.18	2.30	0.001
MEM 27	0.01	0.23	0.18	2.30	0.001
MEM 29	0.01	0.24	0.19	2.24	0.001
MEM 17	0.01	0.24	0.19	2.25	0.001
MEM 18	0.01	0.25	0.19	2.24	0.001
MEM 14	0.01	0.26	0.20	2.13	0.001
MEM 19	0.01	0.26	0.20	2.12	0.001

MEM	R^2	Cumulative R^2	Cumulative adjusted-R^2	F	p-value
MEM 21	0.01	0.27	0.20	2.10	0.001
MEM 22	0.01	0.27	0.21	2.10	0.001
MEM 32	0.01	0.28	0.21	2.03	0.001
MEM 39	0.01	0.28	0.21	1.95	0.001
MEM 23	0.01	0.29	0.21	1.94	0.001
MEM 26	0.01	0.29	0.22	1.94	0.001
MEM 34	0.00	0.30	0.22	1.93	0.001
MEM 35	0.00	0.30	0.22	1.94	0.001
MEM 31	0.00	0.31	0.23	1.87	0.001
MEM 28	0.00	0.31	0.23	1.87	0.001
MEM 20	0.00	0.32	0.23	1.85	0.001
MEM 24	0.00	0.32	0.23	1.84	0.001
MEM 16	0.00	0.33	0.23	1.73	0.001
MEM 49	0.00	0.33	0.24	1.62	0.001
MEM 37	0.00	0.33	0.24	1.62	0.001
MEM 40	0.00	0.34	0.24	1.59	0.001
MEM 53	0.00	0.34	0.24	1.59	0.001

Supp Table S7: ***GEA parameters and results*.**

Summary of genotype–environment association (GEA) model parameters and all SNP results performed using redundancy analyses (RDA) for the four sites (ESS, MAR, PAR, PIE) separately (local; **a-d**) and grouped (regional; **e**). *Model parameters* summarise the proportion of intragenic SNP variance explained by the *K*-retained RDA axes in the GEA analyses, as well as the genomic inflation factors (GIF) used to adjust the distribution of p-values attributed to loci. Columns for *Candidate SNPs, Genes,* and *Enriched GO terms* summarise the number of all and high-impact (H-I) SNPs, genes and GO terms that were detected as significant outliers in GEA analyses, respectively. The number of multiple variable associations are included for *Genes*. *GO terms* are separated into biological processes (BP) and molecular functions (MF).

	**Model parameters**		**Candidate SNPs**			**Genes**				**Enriched GO terms**			
	Prop explained by K axes	GIF	All SNPs	H-I SNPs	Prop unique H-I SNPs	All SNPs	All SNPs: multiple assoc	H-I SNPs	H-I SNPs: multiple assoc	all SNPs: BP	all SNPs: MF	H-I SNPs: BP	H-I SNPs: MF
**A) ESS**													
i) *VS-0.5m*	0.55	1.38	137	57	0.47	85	2	53	0	1			2
ii) *VS-1m*	0.55	1.40	61	38	0.24	33	5	29	5		3		3
iii) *VS-2m*	0.53	1.49	2 290	832	0.30	1 153	75	712	59	3	4	3	2
iv) *VS-4m*	0.53	1.45	145	70	0.00	85	2	59	1	1	2		1
v) *VS-8m*	0.51	1.42	116	51	0.78	65	s	47	3	2	2	2	1
vi) *VS-16m*	0.51	1.52	265	115	0.02	141	6	96	4	2	2	2	2
vii) *VS-all*	0.22	1.61	1 243	506	0.13	628	44	419	36	4	10	5	3
viii) *VS-fwd*	0.44	1.53	1 640	642	0.05	839	44	546	33	6	8	8	5

**B) MAR**													
i) *VS-0.5m*	0.60	1.64	985	431	0.33	559	36	359	30		1	3	
ii) *VS-1m*	0.56	1.55	142	91	0.03	99	2	74	2	1		1	
iii) *VS-2m*	0.56	1.66	700	328	0.08	417	7	281	7	1	1	1	1
iv) *VS-4m*	0.54	1.60	555	261	0.00	345	12	233	11		1	1	
v) *VS-8m*	0.55	1.66	1 051	491	0.23	613	40	402	37	1	1	1	
vi) *VS-16m*	0.55	1.74	878	403	0.00	513	29	335	24	1	1	1	
vii) *VS-all*	0.30	1.58	157	96	0.09	109	9	77	7	5	3	1	3
viii) *VS-fwd*	0.49	1.66	271	145	0.00	186	6	131	5	2		2	
**C) PAR**													
i) *VS-0.5m*	0.54	1.65	2 173	865	0.00	1 052	42	721	33	3	5	2	1
ii) *VS-1m*	0.53	1.67	3 023	1203	0.00	1 440	31	989	26	3	4	1	3
iii) *VS-2m*	0.53	1.72	3 240	1304	0.00	1 525	123	1050	105	3	10	1	5
iv) *VS-4m*	0.53	1.76	4 271	1762	0.00	1 996	155	1389	134	1	5	1	6
v) *VS-8m*	0.53	1.75	4 648	1924	0.00	2 147	175	1495	146	3	7	1	4
vi) *VS-16m*	0.56	1.74	4 718	1963	0.00	2 159	255	1508	209	1	4	1	5
vii) *VS-all*	0.21	1.63	5 789	2315	0.14	2 737	637	1859	507	14	22	6	8
viii) *VS-fwd*	0.61	1.69	4 463	1837	0.00	2 082	128	1450	105	2	9		4

**D) PIE**													
i) *VS-0.5m*	0.63	1.50	135	53	0.04	83	2	54	2	1	1	2	1
ii) *VS-1m*	0.59	1.48	563	249	0.03	283	12	196	11	5	6	6	5
iii) *VS-2m*	0.60	1.55	716	309	0.01	368	11	246	8	3	3	1	
iv) *VS-4m*	0.61	1.64	1 203	522	0.01	607	26	403	18	3	9	7	7
v) *VS-8m*	0.59	1.61	2 286	995	0.01	1 162	66	757	60	6	7	10	8
vi) *VS-16m*	0.57	1.62	2 469	1078	0.01	1 252	53	822	45	4	7	5	7
vii) *VS-all*	0.30	1.79	2 004	859	0.02	1 001	61	654	51	9	9	9	10
viii) *VS-fwd*	0.56	1.61	2 458	1064	0.02	1 254	56	823	48	4	7	6	8
**E) Regional**													
i) *VS-0.5m*	0.47	1.52	149	59	0.00	55	4	41	4				
ii) *VS-1m*	0.46	1.52	175	66	0.00	64	8	47	8				
iii) *VS-2m*	0.45	1.52	205	73	0.00	80	10	56	9				
iv) *VS-4m*	0.43	1.54	182	64	0.00	66	7	49	7				
v) *VS-8m*	0.43	1.54	144	55	0.00	62	3	45	3				
vi) *VS-16m*	0.43	1.56	177	78	0.00	79	1	59	1				
vii) *VS-all*	0.19	1.70	1072	448	0.02	534	74	396	65	10	8	6	8
viii) *VS-fwd*	0.25	1.64	582	254	0.01	303	29	227	28	5	4	3	2

Supp Table S7: ***GEA candidate SNP tally.***

Tally of the high-impact candidate SNPs detected as outliers in genotype–environment associations (GEA), performed with redundancy analyses (RDA). The derived variables (**Supp Table S1**) at each spatial resolution (0.5, 1, 2, 4, 8, 16m) that was most strongly associated with the outlier SNP is indicated by rows. This is done for the four sites (ESS, MAR, PAR, PIE) separately (local, **a-d**) and grouped (regional, **e**). For each site, three variations of the redundancy analysis (RDA) model were performed (as columns), with either all variables at all spatial resolutions (*VS-all*), with variables selected using forward selection methods (*VS-fwd*), or with all variables at a single spatial resolution (*VS-sin*) of either 0.5, 1, 2, 4, 8, 16m spatial resolutions. Note that elevation at 0.5m spatial resolution was included in all variable sets. Variables not used in an RDA model are shaded out in grey.

	**a) ESS**			**b) MAR**			**c) PAR**			**d) PIE**			**e) Regional**		
**Var.**	*VS-all*	*VS-fwd*	*VS-sing*	*VS-all*	*VS-fwd*	*VS-sing*	*VS-all*	*VS-fwd*	*VS-sing*	*VS-all*	*VS-fwd*	*VS-sing*	*VS-all*	*VS-fwd*	*VS-sing*
**0.5m resolution variables**															
Elev		68	1				30	69	13		17	8	4	2	5
EAST	10			7		19	18		74	51		4	10		1
HCU	131					46	52		80	5					27
NORTH			10	3		96	14		1			1	1		
SLO	8		14				32						4		15
SVF				2		83	236			13		25			
SWI			26				73		697	2		3		1	
TI6							49					1			
VRM	44		1			10	16			6		10	2		11
WEX	1		5	1		177	64			61		1			
**Total *VS-0.5m***			**57**			**431**			**865**			**53**			**59**

	a) ESS			b) MAR			c) PAR			d) PIE			e) Regional		
**Var.**	*VS-all*	*VS-fwd*	*VS-sing*	*VS-all*	*VS-fwd*	*VS-sing*	*VS-all*	*VS-fwd*	*VS-sing*	*VS-all*	*VS-fwd*	*VS-sing*	*VS-all*	*VS-fwd*	*VS-sing*
**1m resolution variables**															
Elev			3						128						28
EAST	1			1			10		1058			61	12		1
HCU	1					2	21			8		53	2		1
NORTH	15			2		75	29		17			1	2		
SLO	7		11				12					19	56		9
SVF	17		5	1		2	323								
SWI	1		3			9				1		32	2		12
TI6			8				68					3			
VRM	48		8				27			2		63	40	42	14
WEX						3	4					17			1
**Total *VS-1m***			**38**			**91**			**1203**			**249**			**66**
**2m resolution variables**															
Elev			56						94						26
EAST	4		7				27		609			30	2		
HCU	2		6			16	11					3			
NORTH	2		12	1	19	91	29	1071	99				1		
SLO			14				1					37	130	100	29
SVF	2		40			83	91						37		
SWI	10		247		5	12	10		498	39		118			16
TI6			60				69								
VRM	43	168	387							25		121	22	22	2
WEX			3		15	126	107		4						
**Total *VS-2m***			**832**			**328**			**1304**			**309**			**73**

	a) ESS			b) MAR			c) PAR			d) PIE			e) Regional		
**Var.**	*VS-all*	*VS-fwd*	*VS-sing*	*VS-all*	*VS-fwd*	*VS-sing*	*VS-all*	*VS-fwd*	*VS-sing*	*VS-all*	*VS-fwd*	*VS-sing*	*VS-all*	*VS-fwd*	*VS-sing*
**4m resolution variables**															
Elev			7						72			3			14
EAST	1						10		305			88	2		27
HCU						14	50		104	201		149	11		
NORTH	3		9	1		79	11		1105			9	3		
SLO			4									33	25	15	20
SVF	1					57	46	56	2			16			
SWI	1	40	22			20	20		132	54		122	8	31	
TI6			28				49		38				4	6	
VRM										80		40	13	19	3
WEX				2		91	22		4	10	332	62			
**Total *VS-4m***			**70**			**261**			**1762**			**522**			**64**
**8m resolution variables**															
Elev						1			60						31
EAST	2	7	1				13		214	1		101	4		3
HCU	3					23	89		1235	139		388			
NORTH	2	22	4			162	21		180			3	1	1	
SLO	1	1	34									25	24		21
SVF			1			14	50		24						
SWI		12	6	3		91	94		186	21		82			
TI6	3	26	1			34	47		21						
VRM						35			4	55		144			
WEX			4			131				18	98	252	1	2	
**Total *VS-8m***			**51**			**491**			**1924**			**995**			**55**

	a) ESS			b) MAR			c) PAR			d) PIE			e) Regional		
**Var.**	*VS-all*	*VS-fwd*	*VS-sing*	*VS-all*	*VS-fwd*	*VS-sing*	*VS-all*	*VS-fwd*	*VS-sing*	*VS-all*	*VS-fwd*	*VS-sing*	*VS-all*	*VS-fwd*	*VS-sing*
**16m resolution variables**															
Elev			2						55			8			27
EAST	126	264	40		45	96	9		180	14		72	2		1
HCU				29	4	9	57		505	1	2	11			
NORTH			1	40		89	78		4		21	8	11	5	
SLO			4								1	1	11	8	48
SVF		15				9	30	641	117	1	122	116			
SWI			5	3		163	30		7	30	375	640			2
TI6	10		62			8	108		52						
VRM	5	4	1		56	17	51		1038	5	96	110	1		
WEX	1	15			1	12	7		5	16		112			
**Total *VS-16m***			**115**			**403**			**1963**			**1078**			**78**
**Total *VS-all* *VS-fwd***	**506**	**642**		**96**	**145**		**2315**	**1837**		**859**	**1064**		**448**	**254**	

Supp Table S9: ***GEA candidate gene tally.***

Tally of the genes associated with high-impact candidate SNP outliers, detected through genotype–environment associations (GEA), performed with redundancy analyses (RDA). The derived variable (**Supp Table S1**) at each spatial resolution (0.5, 1, 2, 4, 8, 16m) that was most strongly associated with the outlier SNP is indicated by rows. In cases where a gene had multiple SNP-variable associations, this was listed as 'multiple'. This is done for the four sites (ESS, MAR, PAR, PIE) separately (local, **a-d**) and grouped (regional, **e**). For each site, three variations of the redundancy analysis (RDA) model were performed (as columns), with either all variables at all spatial resolutions (*VS-all*), with variables selected using forward selection methods (*VS-fwd*), or with all variables at a single spatial resolution (*VS-sin*) of either 0.5, 1, 2, 4, 8, 16m spatial resolutions. Note that elevation at 0.5m spatial resolution was included in all variable sets. Variables not used in an RDA model are shaded out in grey.

	**a) ESS**			**b) MAR**			**c) PAR**			**d) PIE**			**e) Regional**		
**Var.**	*VS-all*	*VS-fwd*	*VS-sing*	*VS-all*	*VS-fwd*	*VS-sing*	*VS-all*	*VS-fwd*	*VS-sing*	*VS-all*	*VS-fwd*	*VS-sing*	*VS-all*	*VS-fwd*	*VS-sing*
**0.5m resolution variables**															
Elev		40	1				18	57	13		18	5	2	5	5
EAST	8		1	6		17	18		54	46		8	9		1
HCU	110					37	20		66	8					17
NORTH			3	3		65	6		1			3	1		
SLO	6		15				22						3		8
SVF						68	146			9		19			
SWI			27				28		554			5		1	
TI6							30								
VRM	26		1			7	3			5		9	5		6
WEX	1		5			135	37			37		3			
Multi.						**30**			**33**			**2**			**4**
**Total *VS-0.5m***			**53**			**359**			**721**			**54**			**41**

	a) ESS			b) MAR			c) PAR			d) PIE			e) Regional		
**Var.**	*VS-all*	*VS-fwd*	*VS-sing*	*VS-all*	*VS-fwd*	*VS-sing*	*VS-all*	*VS-fwd*	*VS-sing*	*VS-all*	*VS-fwd*	*VS-sing*	*VS-all*	*VS-fwd*	*VS-sing*
**1m resolution variables**															
Elev			1						103						17
EAST	1			1			4		850			59	9		
HCU							11			10		37	1		
NORTH	12					60	16		10			4	2		
SLO	5		8				10					11	38		6
SVF	11		4			1	216								
SWI	3		4			7				1		22	5		9
TI6			4				39					3			
VRM	38		3				10			2		38	23	28	4
WEX						4	5					11			3
Multi.			**5**			**2**			**26**			**11**			**8**
**Total *VS-1m***			**29**			**74**			**989**			**196**			**47**
**2m resolution variables**															
Elev			40						73			1			13
EAST	1		7				7		433			28	2		
HCU	2		7			15	6					3	4		
NORTH	3		5		9	74	21	830	60				3		
SLO			16				1					34	92	75	22
SVF	1		31			67	55								
SWI	10		204		6	13	8		376	24		95	22		10
TI6			32				39								
VRM	33	144	309							13		77	15	18	2
WEX			2		14	105	79		3						
Multi.			**59**			**7**			**105**			**8**			**9**
**Total *VS-2m***			**712**			**281**			**1050**			**246**			**56**

	a) ESS			b) MAR			c) PAR			d) PIE			e) Regional		
**Var.**	*VS-all*	*VS-fwd*	*VS-sing*	*VS-all*	*VS-fwd*	*VS-sing*	*VS-all*	*VS-fwd*	*VS-sing*	*VS-all*	*VS-fwd*	*VS-sing*	*VS-all*	*VS-fwd*	*VS-sing*
**4m resolution variables**															
Elev			2						50			4			14
EAST	1		1				3		217			73	3		12
HCU						13	25		72	162		122			
NORTH	2		6	1		61	9		814			6	5		
SLO			4									20	19	10	14
SVF	1					50	24	30				14			
SWI	1	33	24			18	6		83	37		93	8	24	
TI6			21				31		16				5	7	
VRM										41		14	4	12	2
WEX				2		80	15		3	5	250	39			
Multi.			**1**			**11**			**134**			**18**			**7**
**Total *VS-4m***			**59**			**233**			**1389**			**403**			**49**
**8m resolution variables**															
Elev						2			54			1			20
EAST	2	7	1				1		142			72	5	2	4
HCU	3					23	55		916	88		304			
NORTH	2	15	4	1		121	10		119			3	2	1	
SLO	1	1	28									16	17		17
SVF						7	34		11						
SWI		6	6	3		53	41		91	14		50			
TI6	1	18	1			28	30		14						
VRM						28			2	42		105			
WEX			4			103				11	65	146		3	1
Multi.			**3**			**37**			**146**			**60**			**3**
**Total *VS-8m***			**47**			**402**			**1495**			**757**			**45**

	a) ESS			b) MAR			c) PAR			d) PIE			e) Regional		
**Var.**	*VS-all*	*VS-fwd*	*VS-sing*	*VS-all*	*VS-fwd*	*VS-sing*	*VS-all*	*VS-fwd*	*VS-sing*	*VS-all*	*VS-fwd*	*VS-sing*	*VS-all*	*VS-fwd*	*VS-sing*
**16m resolution variables**															
Elev			2						47			8			18
EAST	88	216	38		42	76	5		85	12		45	4		1
HCU				17	3	6	38		337			11			
NORTH			1	35		72	69		2		19	4	14	9	
SLO			4								1	1	7	4	36
SVF		13				6	12	428	65	1	94	83			
SWI			1	1		127	14		5	23	267	474			3
TI6	7		45			8	53		21						
VRM	2	3	1		50	7	20		735	1	61	76	2		
WEX	1	17			2	9	2		2	11		75			
Multi.			**4**			**24**			**209**			**45**			1
**Total *VS-16m***			**96**			**335**			**1508**			**822**			**59**
**Total multi.**	**36**	**33**		**7**	**5**		**507**	**105**		**51**	**48**		**65**	**28**	
**Total vs-all vs-fwd**	**419**	**546**		**77**	**131**		**1859**	**1450**		**654**	**823**		**396**	**227**	
