# Supplementary material for: Integrating very high resolution environmental proxies in genotype–environment association studies
Source: Evol Appl. 2024 Jun 28;17(7):e13737. doi: 10.1111/eva.13737 (PMC11212006; doi:10.1111/eva.13737)
Supplement: Supplementary file 2 — Appendix S2. [file EVA-17-e13737-s002.pdf]

Variation partitioning

Methods

A partial redundancy analysis (pRDA) variance partitioning method was used to investigate patterns of spatial genetic variation at local (ESS, MAR, PAR, PIE) and regional (aggregated across all local sites) levels. Specifically, the relative effects of neutral (population structure and geographic spatial structuring) and adaptive processes (local adaptation) on explaining intragenic SNP variation were disentangled. In a pRDA, the model looks to estimate the proportion of variance explained by a group of explanatory variables while controlling for other variables of interest (Legendre and Legendre, 1998), such that the independent contributions of each variable group can be separated from confounding effects due to collinearity amongst variable group (Peres-Neto et al., 2006). Here, the estimated independent effects of i) population structure, ii) spatial geographic structure (geography), and iii) local adaptation (environmental drivers) on observed intragenic SNP variation at each site were investigated, using the *varpart* function in the vegan R package.

Neutral genetic structure resulting from demographic history was accounted for using PCAs of the LD-pruned intergenic SNP datasets, performed with the *rda* function in the vegan R package. The number of principal components (PCs) retained to represent population structure was determined visually using scree plots and biplots. Similarly, geography was accounted for using Moran Eigenvector Maps (MEMs), following Dray et al. (2006). Briefly, plant neighbours were triangulated using geographic coordinates (X, Y) to estimate weightings of MEMs using the *graph2nb* and *nb2listw* functions of the spdep R package (v.1.2.3; Bivand, 2022). Moran's *I* was calculated for each MEM eigenvector of the weighting matrix, using 999 permutations using the *scores.listw* and *test.scores* functions of the spacemakeR R package (v.0.0-5/r113; Dray, 2013), where only MEMs with a p-value<0.01 were retained. The number of MEMs were further reduced using a forward selection procedure, retaining MEMs that best explained variance in the neutral LD-pruned intergenic SNP dataset, with the full RDA model's adjusted-R^2 value as the stopping criteria, which was performed using the *forward.sel* function of the adespatial R package (v.0.3-16; Dray et al., 2022).

The contribution of elevation and DEM-derived variables in shaping genetic variation and supporting a pattern of local adaptation was assessed using variables at a range of spatial resolutions. The effect of each of the variable sets (**Table 2** in the main

text) was systematically evaluated for the local and regional analyses. As elevation is known to be correlated with environmental variables such as temperature and humidity (Ashcroft and Gollan, 2013; Hof et al., 2012), it was removed from each of the variable sets and evaluated separately.

Results

Up to half of genomic variation across each local site was explained by either neutral processes, including demographic history (population structure) and spatial geographic structure (geography), or by adaptive processes yielding patterns of local adaptation (**Suppl. Fig. S5; Suppl. Table S5**), where most of explained variation was confounded between neutral processes (**Suppl. Fig. S6**). Total explained genetic variation was predominantly influenced by the number of input environmental variables used in analyses, rather than the spatial resolution of the variable set (**Table 2**). At local sites, population structure, accounted for using PCAs of the neutral intergenic dataset (**Suppl. Fig. S7a–d**), explained relatively limited variance alone, as it was highly confounded with geography such as latitudinal coordinates (**Suppl. Fig. S3**). Geography (accounted for using MEMs; **Suppl. Fig. S8; Suppl. Table S6**) explained more intragenic variance at sites with homogeneous rather than heterogeneous terrains, where geography was stronger when modelled with finer-resolution *VS-single* models (**Suppl. Fig. S5**). After accounting for neutral spatial genetic structuring, elevation explained very little genetic variation on its own (generally <1%) at the local sites. Likewise, the environmental partition explained little genetic variation alone, where patterns of local adaptation were stronger at homogeneous than at heterogeneous terrain sites (**Suppl. Fig. S5**).

At the regional level, almost half of the total intragenic variation was explained by neutral or adaptive processes, half of which was confounded (**Suppl. Fig. S3e; Suppl. Fig. S5**). In contrast to the local sites, more than half of this unconfounded variance was shaped purely by geography and, to a much lesser extent, also population structure (**Suppl. Fig. S7e**). Patterns of local adaptation however remained weak at the regional level, with elevation accounting for <1% of explained genetic variation, and the remaining environmental variables accounting for <3% with *VS-single* and <15% with *VS-fwd* and *VS-all.*

References

Ashcroft, M.B., Gollan, J.R., 2013. Moisture, thermal inertia, and the spatial distributions of near-surface soil and air temperatures: Understanding factors that promote microrefugia. Agric. For. Meteorol. 176, 77–89. https://doi.org/10.1016/j.agrformet.2013.03.008

Bivand, R., 2022. R packages for analyzing spatial data: a comparative case study with areal data. Geogr. Anal. 54, 488–518. https://doi.org/10.1111/gean.12319

Dray, S., 2013. spacemakeR: Spatial modelling. R package version 0.0-5/r113.

Dray, S., Bauman, D., Blanchet, G., Borcard, D., Clappe, S., Guenard, G., Jombart, T., Larocque, G., Legendre, P., Madi, N., Wagner, H.H., 2022. adespatial: Multivariate Multiscale Spatial Analysis. R package version 0.3-16.

Dray, S., Legendre, P., Peres-Neto, P.R., 2006. Spatial modelling: a comprehensive framework for principal coordinate analysis of neighbour matrices (PCNM). Ecol. Modell. 196, 483–493. https://doi.org/10.1016/j.ecolmodel.2006.02.015

Hof, A.R., Jansson, R., Nilsson, C., 2012. The usefulness of elevation as a predictor variable in species distribution modelling. Ecol. Modell. 246, 86–90. https://doi.org/10.1016/j.ecolmodel.2012.07.028

Legendre, P., Legendre, L., 1998. Numerical ecology. Elsevier, Amsterdam, The Netherlands.

Peres-Neto, P.R., Legendre, P., Dray, S., Borcard, D., 2006. Variation partitioning of species data matrices: Estimation and comparison of fractions. Ecology 87, 2614–2625. https://doi.org/10.1890/0012-9658(2006)87[2614:VPOSDM]2.0.CO;2
